# Fenebrutinib in H_1_ antihistamine-refractory chronic spontaneous urticaria: a randomized phase 2 trial

**DOI:** 10.1038/s41591-021-01537-w

**Published:** 2021-11-08

**Authors:** Martin Metz, Gordon Sussman, Rémi Gagnon, Petra Staubach, Tonny Tanus, William H. Yang, Jeremy J. Lim, Holly J. Clarke, Joshua Galanter, Leslie W. Chinn, Tom Chu, Anastasia Teterina, Tracy Burgess, D. James Haddon, Timothy T. Lu, Marcus Maurer

**Affiliations:** 1 Dermatological Allergology, Allergie-Centrum-Charité, Department of Dermatology and Allergy, Charité-Universitätsmedizin Berlin, Berlin, Germany; 2 Allergology, Fraunhofer Institute for Translational Medicine and Pharmacology ITMP, Berlin, Germany; 3 Division of Allergy and Clinical Immunology, St. Michael’s Hospital and University of Toronto, Toronto, Ontario Canada; 4 Service d’Allergie et Immunologie, Département de Médecine, Centre Hospitalier Universitaire de Québec, Quebec City, Québec Canada; 5 Department of Dermatology, University Medical Center Mainz, Mainz, Germany; 6 Kern Allergy Medical Clinic Inc., Bakersfield, CA USA; 7 Ottawa Allergy Research Corporation, Department of Medicine, University of Ottawa, Ottawa, Ontario Canada; 8 Genentech, Inc., South San Francisco, CA USA; 9 Hoffman-LaRoche Limited, Missisauga, Ontario Canada

**Keywords:** Immunological disorders, Mast cells, Randomized controlled trials, Skin manifestations

## Abstract

Bruton’s tyrosine kinase (BTK) is crucial for FcεRI-mediated mast cell activation and essential for autoantibody production by B cells in chronic spontaneous urticaria (CSU). Fenebrutinib, an orally administered, potent, highly selective, reversible BTK inhibitor, may be effective in CSU. This double-blind, placebo-controlled, phase 2 trial (EudraCT ID 2016-004624-35) randomized 93 adults with antihistamine-refractory CSU to 50 mg daily, 150 mg daily and 200 mg twice daily of fenebrutinib or placebo for 8 weeks. The primary end point was change from baseline in urticaria activity score over 7 d (UAS7) at week 8. Secondary end points were the change from baseline in UAS7 at week 4 and the proportion of patients well-controlled (UAS7 ≤ 6) at week 8. Fenebrutinib efficacy in patients with type IIb autoimmunity and effects on IgG-anti-FcεRI were exploratory end points. Safety was also evaluated. The primary end point was met, with dose-dependent improvements in UAS7 at week 8 occurring at 200 mg twice daily and 150 mg daily, but not at 50 mg daily of fenebrutinib versus placebo. Asymptomatic, reversible grade 2 and 3 liver transaminase elevations occurred in the fenebrutinib 150 mg daily and 200 mg twice daily groups (2 patients each). Fenebrutinib diminished disease activity in patients with antihistamine-refractory CSU, including more patients with refractory type IIb autoimmunity. These results support the potential use of BTK inhibition in antihistamine-refractory CSU.

## Abstract (web summary)

Fenebrutinib, an oral Bruton’s tyrosine kinase inhibitor, reduces disease activity in patients with chronic spontaneous urticaria refractory to antihistamines, suggesting that this treatment type could be an alternative to standard of care anti-IgE therapy.

## Main

CSU is characterized by the development of wheals, angioedema or both for more than six weeks without an obvious external cause. Approximately 1% of the population is affected (point prevalence = 0.7%; lifetime prevalence = 1.4%); in most patients, the disease considerably impairs quality of life^1,2^.

CSU pathogenesis is not yet fully understood but autoallergic and autoimmune mechanisms are believed to be involved^3–6^. A large proportion of patients have autoallergic CSU, also known as type I autoimmune CSU, where IgE autoantibodies target various autoantigens, for example, thyroid peroxidase^7,8^ and interleukin-24^5,9^. Other patients have type IIb autoimmune CSU, where IgG autoantibodies target IgE or the high-affinity IgE receptor, FcεRI^3,10,11^. These autoantibodies activate mast cells and basophils via FcεRI receptors^4,11–15^. Type IIb autoantibodies fix complement^16^, which augments the secretion of mast cell mediators^17^.

While on current standard of care (that is, anti-IgE therapy), only 40% of patients with CSU in clinical trials achieve complete symptom control^18^, the guideline-supported goal of urticaria treatment^1^. Omalizumab, a humanized anti-IgE antibody, is recommended by international guidelines as third-line treatment for patients with CSU refractory to H_1_ antihistamines^1^. After treatment with omalizumab (300 mg), 56–66% of patients with CSU were well controlled (UAS7 ≤ 6), compared to 17–25% in the placebo group^19^. More recently, ligelizumab, a high-affinity anti-IgE therapy, demonstrated efficacy in CSU^20^ and is currently in phase 3 clinical trials (ClinicalTrials.gov IDs: NCT03580369, NCT03580356). Despite these new treatments, some patients do not respond and still need effective alternative therapy. This seems especially true for patients with type IIb autoimmunity, who tend to have increased disease activity^21^ and severity^22^ and may be more refractory to current therapies^23–25^. A positive basophil histamine release assay (BHRA^+^) indicates the presence of functional anti-IgE and/or anti-FcεRI autoantibodies and is a marker of type IIb autoimmunity^3,26^. Low serum total IgE is also associated with type IIb autoimmunity^27,28^.

FcεRI cross-linking rapidly activates BTK in both mast cells and basophils, the key cell types in CSU pathogenesis. BTK-null mice and patients deficient in BTK have impaired FcεRI signaling, which decreases histamine and inflammatory cytokine release by mast cells^29^ and basophils^30^. In addition, BTK inhibition with ibrutinib demonstrated a reduction in mast cell and IgE-dependent basophil reactivity in allergic patients with chronic lymphocytic leukemia and in patients with IgE-mediated allergy to peanut and/or tree nuts^31,32^.

Fenebrutinib, an orally administered, potent, highly selective, reversible BTK inhibitor, blocks IgE-mediated histamine release from mast cells *i*n vitro. In a recent phase 1 study, fenebrutinib inhibited IgE-mediated basophil activation in healthy volunteers^33^. Furthermore, BTK is critical in B cells for B cell receptor (BCR) signaling, proliferation, and survival. In patients with rheumatoid arthritis and lupus, fenebrutinib caused dose-dependent reductions in rheumatoid factor and anti-double-stranded DNA autoantibodies, respectively^34,35^. Thus, BTK inhibition by fenebrutinib may also disrupt production of FcεRI-activating autoantibodies in CSU. This phase 2 study was originally designed as a pilot study to assess initial clinical efficacy of 200 mg twice daily of fenebrutinib compared to placebo in adult patients with CSU (cohort 1). Based on results from an interim analysis, the study was amended to include a dose-ranging cohort (cohort 2) to characterize the dose– and exposure–response relationships for safety and efficacy to select the optimal dose for potential further studies.

## Results

### Study design and participants

**Extended Data Fig. 1:**
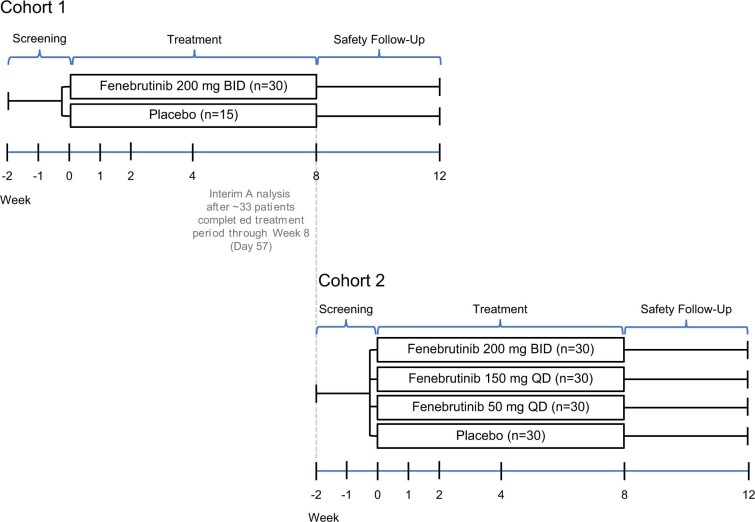
Study design. Study design.

This phase 2, multicenter, randomized, double-blind, placebo-controlled, pilot (cohort 1) and dose-ranging (cohort 2) study (EudraCT ID 2016-004624-35; ClinicalTrials.gov ID NCT03137069) examined fenebrutinib efficacy and safety compared to placebo in adult patients who had CSU for more than six months and were symptomatic despite treatment with H_1_ antihistamines (up to fourfold the approved dose). Both cohorts had a two-week screening period, an eight-week treatment period and a four-week follow-up period (Extended Data Fig. 1). Cohort 2 was initiated after a prespecified interim analysis of cohort 1 (Extended Data Fig. 1). An adaptive study design was used because the study was originally designed as a pilot study and the extent of target engagement was unknown (Methods). This report describes the cohort 2 results with cohort 1 data in the Supplementary Information. Patients in cohort 2 were randomly allocated to placebo or fenebrutinib (50 mg or 150 mg daily or 200 mg twice daily). During the treatment period, patients maintained stable doses of H_1_ antihistamines. The primary end point was the change from baseline in the UAS7 at week 8. Secondary end points were the change from baseline in UAS7 at week 4 and the proportion of patients well-controlled (UAS7 ≤ 6) at week 8. Additionally, we explored fenebrutinib efficacy in patients with type IIb autoimmunity and fenebrutinib effects on IgG-anti-FcεRI levels. We also monitored safety events in this patient population.

**Fig. 1:**
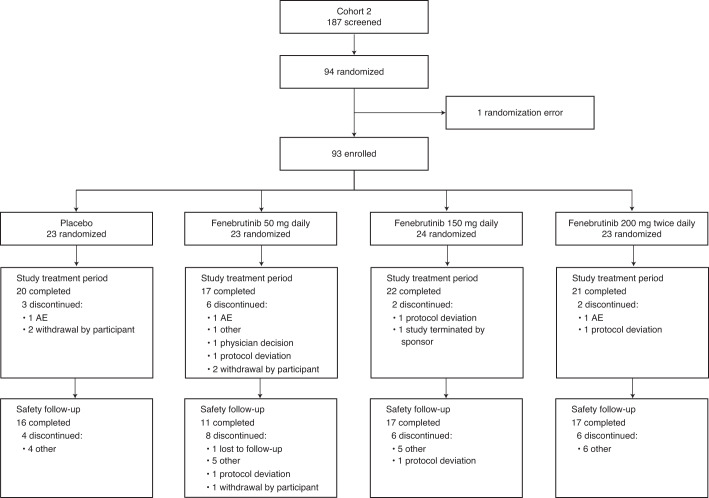
**CONSORT diagram for cohort 2.**

**Extended Data Fig. 2:**
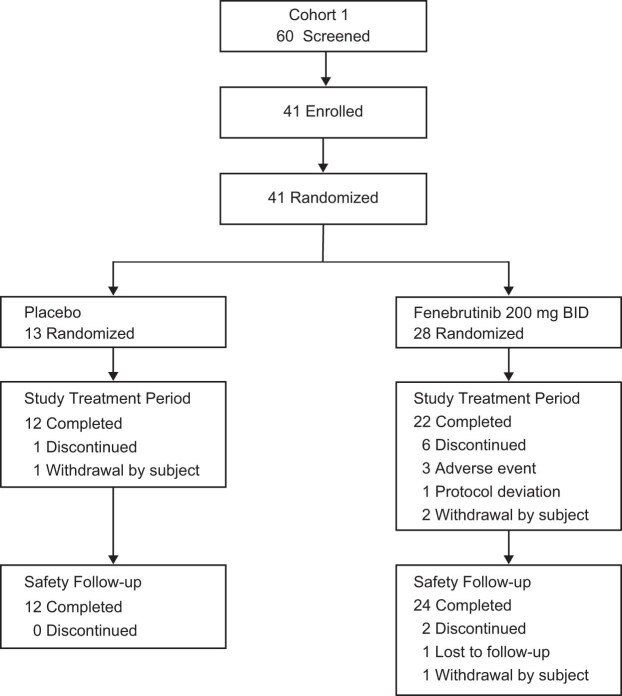
Study flow in cohort 1. Study flow in cohort 1.

Enrollment for Cohort 2 occurred between 28 May 2018 and 1 August 2019. Ninety-three patients were randomly assigned to treatment groups; 80 patients (86%) completed the study treatment period (Fig. 1 and Extended Data Fig. 2, cohort 1). There were no substantial imbalances between treatment groups with respect to baseline CSU symptoms (Table 1 and Supplementary Table 1, cohort 1). We stopped recruitment after an interim analysis of cohort 2 because the study objectives for characterizing fenebrutinib’s efficacy, exposure and safety in patients with CSU were satisfied. We observed a trend in the exposure–efficacy relationship; a biologically relevant subpopulation demonstrated differential efficacy and we observed transient transaminase elevations in a limited number of patients.

**Table 1:** Patient baseline characteristics (modified intention-to-treat population)^a^ in cohort 2

Characteristic	Placebo (*n* = 23)	Fenebrutinib			All patients (*n* = 93)
		50 mg daily (*n* = 23)	150 mg daily (*n* = 24)	200 mg twice daily (*n* = 23)	
Demographics					
Age, years, mean (s.d.)	40.2 (14.7)	45.0 (13.1)	43.3 (16.7)	44.3 (13.0)	43.2 (14.4)
Age group, *n* (%)					
18–40 years	11 (48)	8 (35)	12 (50)	10 (44)	41 (44)
41–64 years	10 (44)	14 (61)	8 (33)	12 (52)	44 (47)
≥65 years	2 (9)	1 (4)	4 (17)	1 (4)	8 (9)
Female, *n* (%)	17 (74)	18 (78)	20 (83)	16 (70)	71 (76)
Ethnicity					
White, *n* (%)	18 (78)	19 (83)	23 (96)	16 (70)	76 (82)
Non-white, *n* (%)	5 (22)	4 (17)	1 (4)	7 (30)	17 (18)
Weight, kg, mean (s.d.)	81.9 (20.0)	83.5 (24.0)	82.4 (20.7)	77.2 (16.7)	81.3 (20.3)
Body mass index, kg m^−^^2^, mean (s.d.)	28.3 (5.9)	30.2 (8.3)	30.6 (7.5)	28.5 (5.6)	29.4 (6.9)
Clinical					
Time since CSU diagnosis, years, median (range)	1.2 (0.0–14.4)	2.1 (0.0–40.0)	2.6 (0.5–36.6)	3.9 (0.6–45.3)	2.2 (0.0–45.3)
UAS7, mean (s.d.)	25.5 (4.7)	29.2 (8.2)	26.6 (7.1)	28.6 (8.5)	27.5 (7.3)
Weekly itch score, mean (s.d.)	12.4 (4.0)	12.8 (5.0)	12.0 (3.7)	12.8 (4.7)	12.5 (4.3)
Weekly hive score, mean (s.d.)	13.1 (4.2)	16.5 (4.6)	14.6 (4.6)	15.8 (5.0)	15.0 (4.7)
In-clinic UCT, mean (s.d.)^b^	4.7 (3.4)	4.7 (2.8)	4.1 (3.2)	4.7 (2.7)	4.5 (3.0)
Presence of angioedema, *n* (%)^b^	12 (54)	12 (52)	11 (46)	9 (39)	44 (48)
Rescue medication use, *n* (%)^b^	15 (68)	17 (74)	16 (67)	14 (61)	62 (67)
Proportion of rescue medication-free days, *n* (%)^b^	40.7 (45)	33.3 (46)	45.3 (46)	42.9 (48)	40.6 (46)
Total IgE, IU ml^−1^, mean (s.d.)	79.4 (127.7)	196.2 (345.6)	82.3 (109.2)	187.9 (246.1)	135.9 (230.4)
BHRA^+^ (≥10), *n* (%)^c^	9 (39)	9 (39)	10 (42)	10 (44)	38 (41)

^a^The modified intention-to-treat population included all patients who had undergone randomization and received at least one dose of a study drug. Percentages may not total 100 because of rounding.

^b^Data are for 22 patients in the placebo group.

^c^BHRA includes week 0, day 1 when the screening biomarker sample was unavailable. Data are for 23 patients in the fenebrutinib 150 mg daily group and for 21 patients in the 200 mg twice daily fenebrutinib group.

### BTK inhibition with fenebrutinib is effective in CSU

**Fig. 2:**
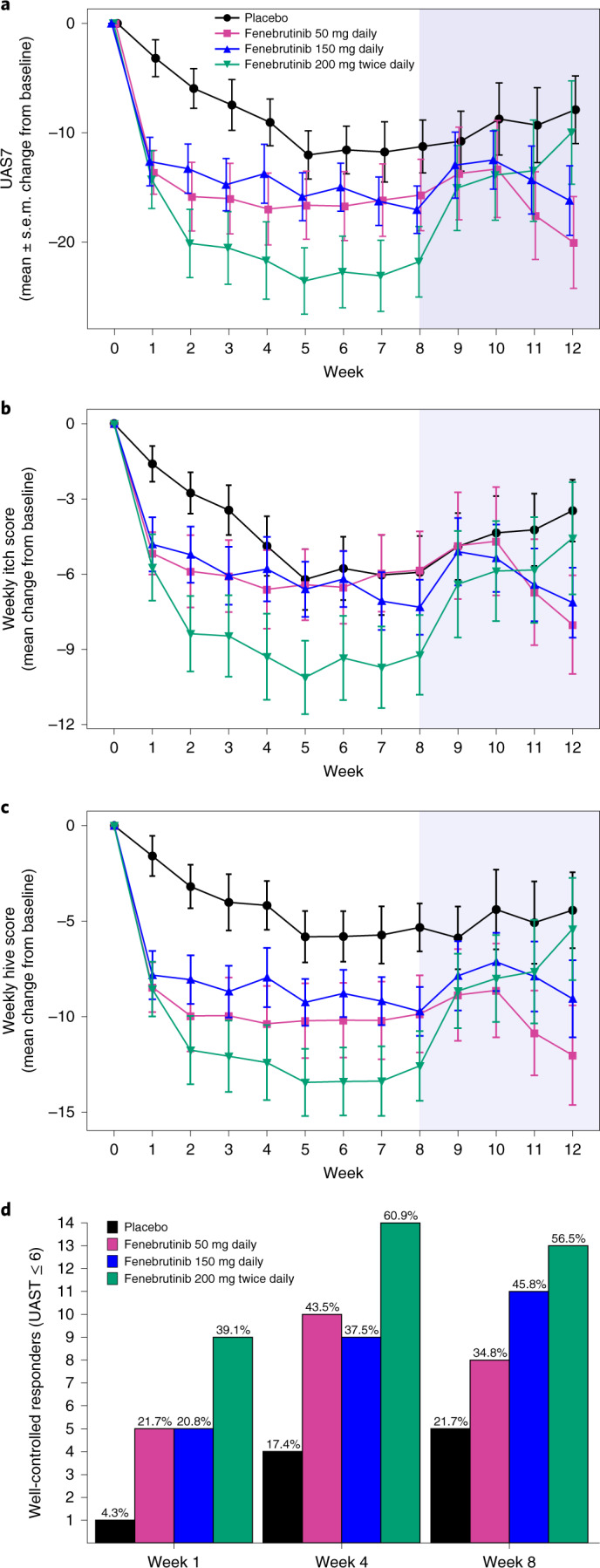
Effects of fenebrutinib on the UAS7, UAS7 components and proportion of well-controlled responders. **a**, Mean profile plot of change from baseline in the UAS7 by study week in the different treatment arms. **b**,**c**, Mean change from baseline in UAS7 components: weekly itch score (**b**) and weekly hive score (**c**). **a**–**c**, *n* = 23 patients for each arm at the initial time point; no imputation for missing values was performed. The center is the mean and the error bars are the s.e.m.; the blue shaded area is the follow-up period. **d**, Proportion of patients who were well-controlled responders (UAS7 ≤ 6). The complete patient numbers per arm are shown in Supplementary Table 1.

Compared with placebo, 150 mg daily and 200 mg twice daily of fenebrutinib resulted in greater mean reductions from baseline in the primary end point, CSU disease activity by UAS7 at week 8 (least squares mean difference versus placebo: 150 mg daily, −6.4; 200 mg twice daily, −9.5; Table 2 and Fig. 2a), which was consistent with changes observed in UAS7 weekly itch (least squares mean difference versus placebo: 150 mg daily, −2.3; 200 mg twice daily, −3.6; Table 2 and Fig. 2b) and hive scores (least squares mean difference versus placebo: 150 mg daily, −4.2; 200 mg twice daily, −6.0; Table 2 and Fig. 2c). Fenebrutinib treatment also led to dose-dependent increases in the proportion of patients with well-controlled disease (UAS7 ≤ 6) at week 8, a secondary end point (50 mg daily, 35%; 150 mg daily, 46%; 200 mg twice daily, 57% versus placebo, 22%; Table 2 and Fig. 2d), and the proportion of patients with complete response at week 8 (50 mg daily, 13%; 150 mg daily, 25%; 200 mg twice daily, 39% versus placebo, 4%; Table 2 and Supplementary Fig. 3). The cohort 1 efficacy outcomes were consistent with cohort 2 (Supplementary Table 2).

**Table 2:** Efficacy end points (modified intention-to-treat population) in cohort 2

End point	Placebo (*n* = 23)	Fenebrutinib		
		50 mg daily (*n* = 23)	150 mg daily (*n* = 24^a^)	200 mg twice daily (*n* = 23)
Primary end point				
UAS7 score, change from baseline to week 8				
Mean (95% CI)^**b**^	−11.2 (−16.3 to −6.0)	−11.7 (−16.9 to −6.5)	−17.6 (−22.6 to −12.7)	−20.7 (−25.8 to −15.6)
Least squares mean difference for treatment versus placebo (95% CI)		−0.5 (−7.8 to 6.8)	−6.4 (−13.4 to 0.6)	−9.5 (−16.7 to −2.4)
Secondary end points				
Well-controlled patients (UAS7 ≤ 6) at week 8, *n* (%)	5 (22)	8 (35)	11 (46)	13 (57)
UAS7 score, change from baseline to week 4				
Mean (95% CI)^a^	−9.6 (−14.8 to −4.3)	−12.4 (−17.8 to −7.0)	−14.6 (−19.7 to −9.4)	−20.3 (−25.6 to −15.0)
Least squares mean difference for treatment versus placebo (95% CI)		−2.8 (−10.3 to 4.7)	−5.0 (−12.3 to 2.2)	−10.8 (−18.2 to −3.3)
Exploratory end points				
Weekly itch score				
Change from baseline to week 4				
Mean (95% CI)^a^	−4.2 (−6.6 to −1.8)	−5.0 (−7.5 to −2.6)	−6.1 (−8.4 to −3.7)	−8.5 (−10.9 to −6.0)
Least squares mean difference for treatment versus placebo (95% CI)		−0.9 (−4.3 to 2.6)	−1.9 (−5.2 to 1.5)	−4.3 (−7.6 to −0.9)
Change from baseline to week 8				
Mean (95% CI)^a^	−5.1 (−7.5 to −2.7)	−4.4 (−6.8 to −1.9)	−7.4 (−9.7 to −5.1)	−8.7 (−11.1 to −6.3)
Least squares mean difference for treatment versus placebo (95% CI)		0.8 (−2.6 to 4.1)	−2.3 (−5.6 to 1.0)	−3.6 (−6.9 to −0.3)
Weekly hives score				
Change from baseline to week 4				
Mean (95% CI)^a^	−5.3 (−8.4 to −2.3)	−7.5 (−10.6 to −4.4)	−8.5 (−11.4 to −5.6)	−11.9 (−14.9 to −8.9)
Least squares mean difference for treatment versus placebo (95% CI)		−2.2 (−6.5 to 2.2)	−3.2 (−7.3 to 1.0)	−6.6 (−10.8 to −2.3)
Change from baseline to week 8				
Mean (95% CI)^a^	−6.0 (−9.0 to −3.1)	−7.4 (−10.3 to −4.5)	−10.3 (−13.0, to −7.5)	−12.0 (−14.9 to −9.1)
Least squares mean difference for treatment versus placebo (95% CI)		−1.4 (−5.5 to 2.8)	−4.2 (−8.2 to −0.3)	−6.0 (−10.0 to −1.9)
Well-controlled patients (UAS7 ≤ 6) at week 4, *n* (%)	4 (17)	10 (44)	9 (38)	14 (61)
Patients with complete response (UAS7 = 0), *n* (%)				
At week 4	1 (4)	2 (9)	3 (12)	8 (35)
At week 8	1 (4)	3 (13)	6 (25)	9 (39)
Patients achieving MID in UAS7 at week 8, *n* (%)	11 (48)	12 (52)	15 (62)	18 (78)
Patients achieving MID in weekly itch score at week 8, *n* (%)	10 (44)	12 (52)	16 (67)	17 (74)

^**a**^One patient in the 150-mg daily arm was discontinued on day 2 and was not included in the MMRM analyses of UAS7, weekly itch score and weekly hives score.

^b^Least squares mean estimates from an MMRM.

### BTK inhibition results in rapid improvement of CSU

**Extended Data Fig. 3:**
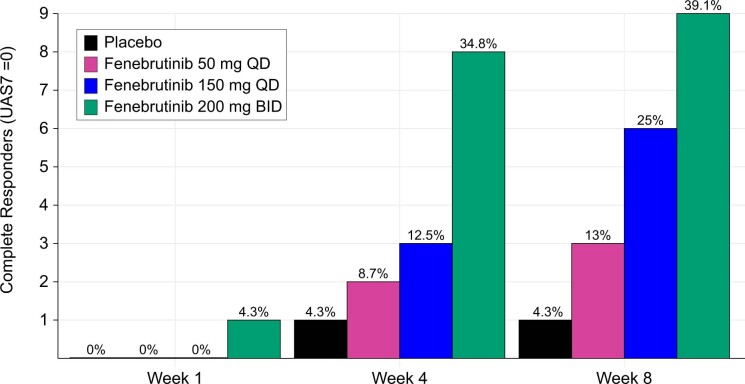
Complete responders in Cohort 2. Proportion of patients who were complete responders in Cohort 2.

**Extended Data Fig. 4:**
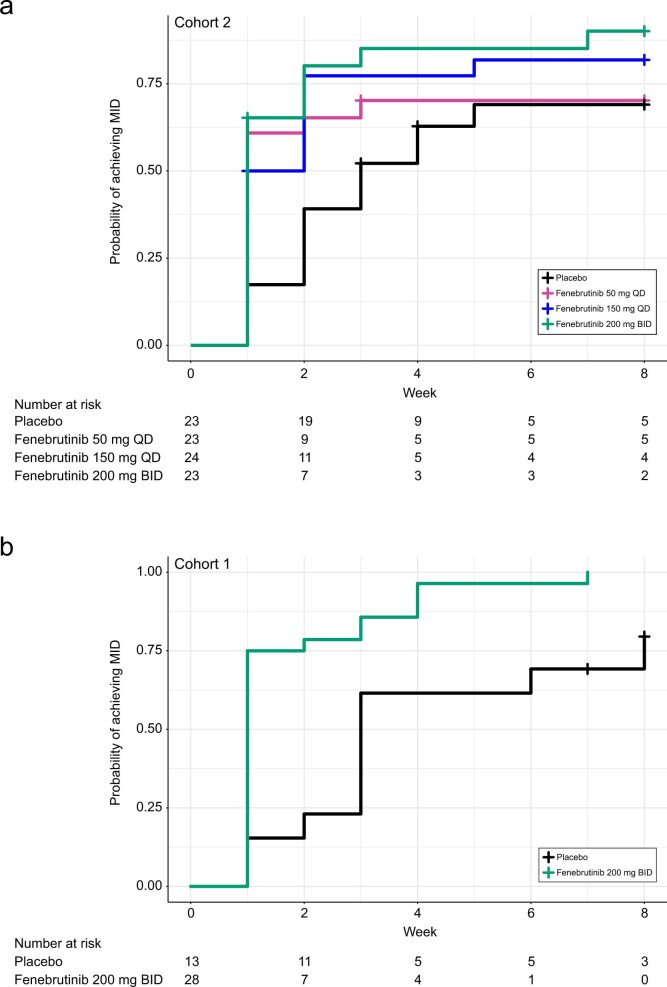
Time to MID in UAS7 score. Time to MID (Kaplan-Meier estimate) in UAS7 score (modified intention-to-treat population). (**a**) Cohort 2. (**b**) Cohort 1.

Near-maximal fenebrutinib efficacy was observed early in the study, with the change from baseline in UAS7 at week 4 (secondary end point), showing a treatment effect similar to the change in the UAS7 seen at week 8 (least squares mean difference versus placebo, 200 mg twice daily (week 4, −10.8; week 8, −9.5); 150 mg daily (week 4, −5.0; week 8, −6.4); 50 mg daily (week 4, −2.8; week 8, −0.5); Table 2 and Fig. 2a) and dose-dependent increases in the proportions of patients with well-controlled disease and complete response at week 4 (Table 2, Fig. 2d and Extended Data Fig. 3). Fenebrutinib reduced median times to the minimally important difference (MID) (200 mg twice daily, 1 week; 150 mg daily, 1.5 weeks; 50 mg daily, 1 week) over placebo (3 weeks) (Table 2 and Extended Data Fig. 4a; Extended Data Fig. 4b, cohort 1). Post-hoc analysis showed that even within the first week of treatment, fenebrutinib led to marked increases in the proportion of well-controlled patients (50 mg daily, 21.7%; 150 mg daily, 20.8%; 200 mg twice daily, 39.1%) compared with placebo (4.3%; Fig. 2d).

### Disease reduction in patients with and without type IIb autoimmunity

**Fig. 3:**
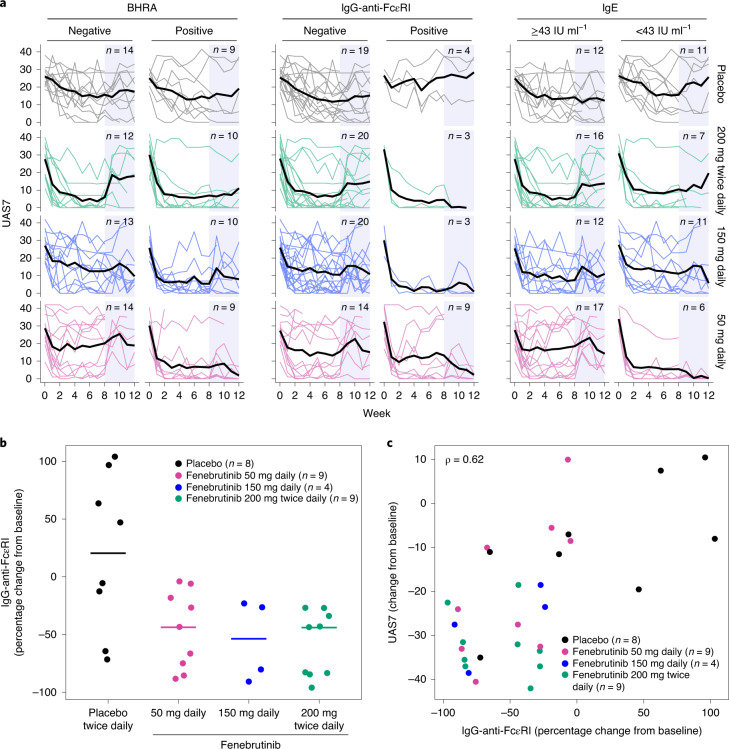
Effects of fenebrutinib on markers of type IIb autoimmunity. **a**, UAS7 values, stratified by baseline BHRA, IgG-anti-FcεRI and IgE concentrations in the placebo and fenebrutinib dose groups from baseline through week 12. Thin lines, individual patients; thick lines, means. No imputation for missing values was performed. The blue shaded area represents the follow-up period. **b**, Percentage change in IgG-anti-FcεRI at week 8. The horizontal bars represent the medians. **c**, Correlation of percentage change in IgG-anti-FcεR1 with change in UAS7 at week 8. **b**,**c**, Analyses were performed in patients who were IgG-anti-FcεRI^+^ at baseline. The *ρ* (rho) value is based on a Spearman correlation.

**Extended Data Fig. 5:**
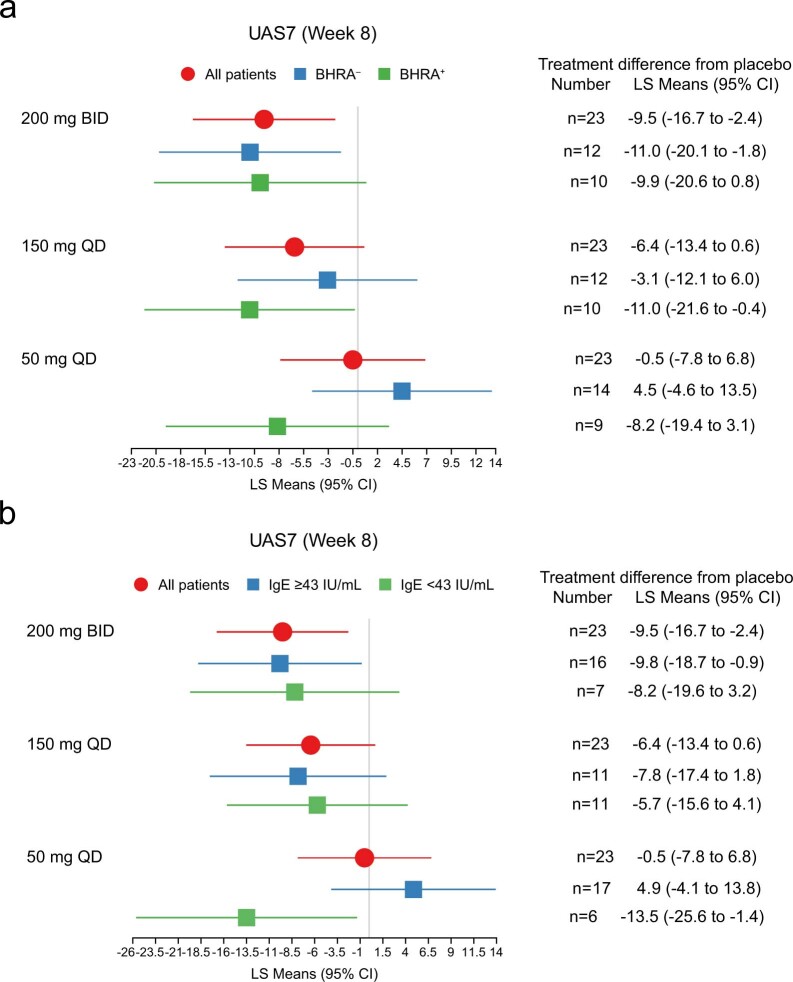
UAS7 at Week 8. UAS7 at Week 8 in all patients and patients stratified by (a) BHRA and (b) IgE in Cohort 2. Stratification by IgG-anti-FcεRI was not performed due to small sample size.

**Extended Data Fig. 6:**
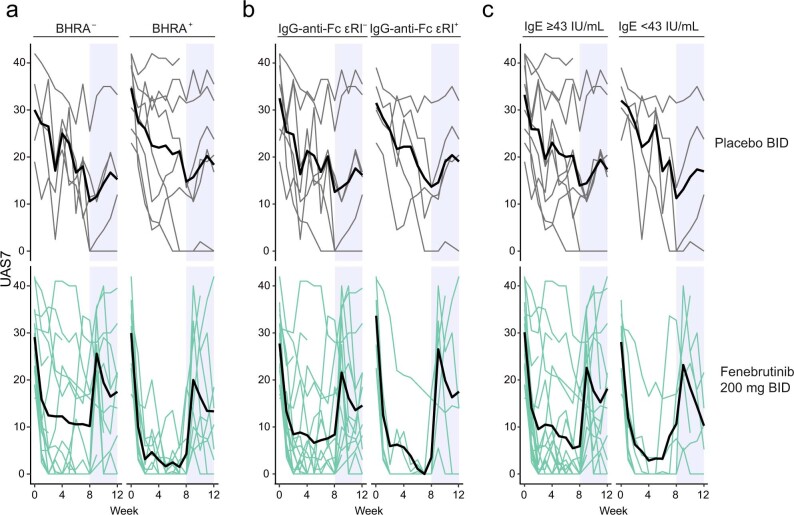
UAS7 stratified by (a) BHRA, (b) IgG-anti-FcεRI, and (c) IgE concentrations in Cohort 1. UAS7 stratified by (**a**) BHRA, (**b**) IgG-anti-FcεRI, and (**c**) IgE concentrations in Cohort 1. Thin lines, individual patients. Thick lines, means. Blue shaded area, follow-up period.

**Extended Data Fig. 7:**
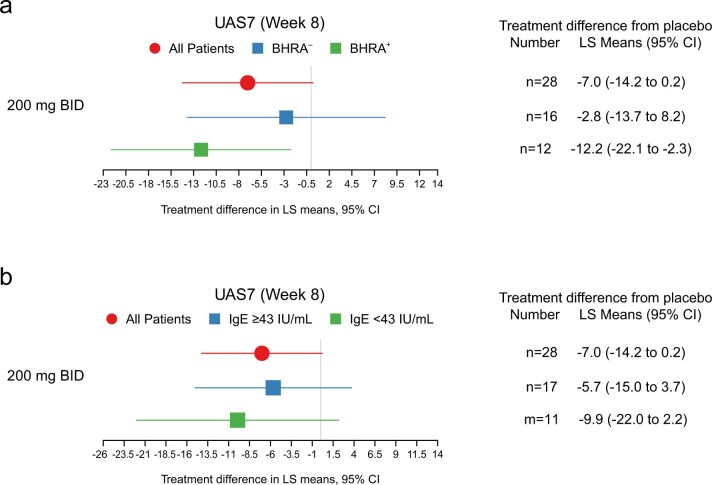
UAS7 at Week 8 in all patients and patients stratified by (a) BHRA and (b) IgE in Cohort 1. UAS7 at Week 8 in all patients and patients stratified by (a) BHRA and (b) IgE in Cohort 1. Stratification by IgG-anti-FcεRI was not performed due to small sample size.

At 200 mg twice daily, fenebrutinib improved disease activity in patients with and without markers of type IIb autoimmunity at baseline (BHRA^+^, IgG-anti-FcεRI^+^ and low serum IgE (<43 IU ml^−1^)^36^), indicated by similar UAS7 improvements for each subgroup compared to placebo (Fig. 3a and Extended Data Fig. 5). The cohort 1 patient subgroups receiving 200 mg twice daily of fenebrutinib showed similar results (Extended Data Fig. 6 and Extended Data Fig. 7).

### Type IIb autoimmunity and lower fenebrutinib dosing

All fenebrutinib groups showed a greater improvement in UAS7 compared to placebo in the BHRA^+^ subgroup (Fig. 3a and Extended Data Fig. 5a). At lower doses (50 mg, 150 mg daily), the UAS7 improved more in BHRA^+^ patients than in BHRA^−^ patients (Fig. 3a and Extended Data Fig. 5). Likewise, IgG-anti-FcεRI^+^ patients showed greater efficacy at the 50 mg and 150 mg daily doses than IgG-anti-FcεRI^−^ patients (Fig. 3a). Patients with low serum total IgE had a greater improvement in UAS7 relative to placebo at 50 mg daily compared to those with high serum IgE (Fig. 3a and Extended Data Fig. 5b).

### BTK inhibition decreases IgG-anti-FcεRI autoantibodies

**Extended Data Fig. 8:**
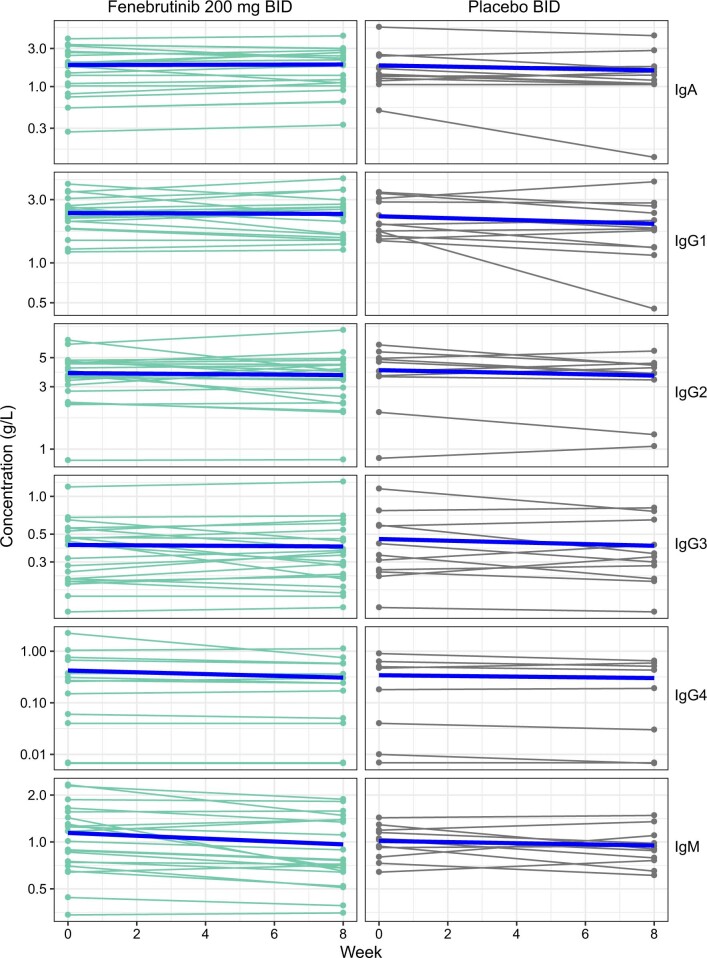
Change in total serum concentrations of immunoglobulin subclasses during the treatment period in Cohort 1. Change in total serum concentrations of immunoglobulin subclasses during the treatment period in Cohort 1. Thin lines, individual patients; thick blue lines, means.

Because fenebrutinib reduces autoantibody production in rheumatoid arthritis^35^ and lupus^34^, this mechanism may also mediate efficacy in type IIb CSU. Among patients with IgG-anti-FcεRI autoantibodies, fenebrutinib substantially reduced these autoantibodies at week 8 at all dose levels compared to placebo (median percentage change from baseline: −43.7, −53.6 and −44.0% in 50 mg daily, 150 mg daily and 200 mg twice daily fenebrutinib groups, respectively; placebo, 20.4%) (Fig. 3b). These changes were probably not due to a broader treatment effect on total antibodies; in cohort 1, compared to placebo, fenebrutinib did not cause obvious reductions in IgG subtypes (Extended Data Fig. 8), including IgG1 and IgG3, the predominant subclasses of IgG-anti-FcεRI in CSU^37^. Greater reductions in IgG-anti-FcεRI were associated with greater decreases in UAS7 at week 8 (Fig. 3c).

### Fenebrutinib is well-tolerated

The most common adverse events (AEs) in cohort 2 were urticaria, nasopharyngitis and headache (Table 3 and Supplementary Table 3), which is consistent with cohort 1 (Supplementary Tables 4 and 5). No serious AEs (SAEs) occurred in cohort 2 (Table 3) and 2 of the 3 SAEs reported in the 200 mg twice daily fenebrutinib arm in cohort 1 were related to treatment (Supplementary Table 4). These SAEs, periorbital cellulitis and an increase in hepatic enzymes, led to treatment withdrawal. One SAE, abdominal pain, was unrelated to the study drug and did not lead to a change in treatment. No deaths were reported in any treatment group. Severe urticaria was reported in four patients in cohort 1 after fenebrutinib treatment ceased. A similar proportion of patients in cohort 2 discontinued study treatment due to AEs in the fenebrutinib arms compared with the placebo arm (Table 3). No serious, severe or opportunistic infections occurred in cohort 2. One patient in the fenebrutinib group in cohort 1 experienced sinusitis and periorbital cellulitis (Supplementary Table 4 and Supplementary Table 5).

**Table 3:** AEs (safety population^a^) in cohort 2

	Placebo (*n* = 22)^b^	Fenebrutinib			All patients (*n* = 93)
		50 mg daily (*n* = 23)	150 mg daily (*n* = 24)	200 mg twice daily (*n* = 24)^b^	
At least one AE, *n* (%)	12 (54)	14 (61)	16 (67)	14 (58)	56 (60)
Overall total number of AEs	33	38	35	41	147
Any AE leading to discontinuation of the study drug, *n* (%)	1 (4)	2 (9)	0	1 (4)	4 (4)
Early withdrawal from the study treatment period due to an AE, *n* (%)	1 (4)	1 (4)	0	1 (4)	3 (3)
Any SAE, *n* (%)	0	0	0	0	0
Death, *n* (%)	0	0	0	0	0
Any AE suspected to be caused by the study drug, *n* (%)	6 (27)	4 (17)	5 (21)	6 (25)	21 (23)
Any severe AE, *n* (%)	2 (9)	1 (4)	1 (4)	1 (4)	5 (5)
Most common AEs (preferred term) in ≥3 patients overall, *n* (%)					
Urticaria	2 (9)	3 (13)	4 (17)	5 (21)	14 (15)
Nasopharyngitis	1 (4)	3 (13)	3 (13)	3 (12)	10 (11)
Headache	2 (9)	0	1 (4)	3 (12)	6 (6)
Nausea	0	1 (4)	2 (8)	2 (8)	5 (5)
Chronic spontaneous urticaria	1 (4)	1 (4)	2 (8)	0	4 (4)
Urinary tract infection	0	1 (4)	2 (8)	1 (4)	4 (4)
ALT increased	0	0	1 (4)	3 (12)	4 (4)
AST increased	0	0	1 (4)	2 (8)	3 (3)
Upper respiratory tract infection	1 (4)	0	0	2 (8)	3 (3)
Diarrhea	2 (9)	0	1 (4)	0	3 (3)
Fatigue	2 (9)	0	1 (4)	0	3 (3)

^a^The safety population was defined according to the treatment actually received.

^b^One patient who was randomized to the placebo group and received placebo was placed in the 200 mg twice daily group for safety analyses due to a data entry error.

AEs were coded using the Medical Dictionary for Regulatory Activities v.22.1. ALT, alanine aminotransferase; AST, aspartate aminotransferase.

Grade 2 and 3 liver transaminase elevations occurred between weeks 4 and 8 in 2 patients each in the 150 mg daily and 200 mg twice daily fenebrutinib groups (Supplementary Table 6, cohort 2). Similar elevations were seen in fenebrutinib-treated patients in cohort 1 (Supplementary Table 7; see patient narratives in the Supplementary Information). These increases were asymptomatic and resolved 10–30 d after fenebrutinib discontinuation. Patients were asymptomatic and no cases met the Hy’s law criteria for drug-induced liver injury^38^.

Compared with placebo, dose-dependent increases in mean creatinine occurred in the fenebrutinib arms starting in week 1 and continuing through week 8. No other clinically meaningful changes were observed in any other laboratory parameters.

## Discussion

Selective inhibition of BTK, a key protein downstream of FcεRI and BCR signaling, with fenebrutinib resulted in clinically meaningful treatment benefit in patients with CSU refractory to antihistamines (up to fourfold the approved dose). The rapid onset of efficacy suggests that fenebrutinib’s major mechanism of action in CSU is inhibition of FcεRI signaling via BTK inhibition in mast cells^29,39^ and basophils^33,40^. Furthermore, the reduction of disease activity with 200 mg twice daily fenebrutinib in patients with and without type IIb autoimmunity indicates that BTK is crucial for maintaining pathology in type I and IIb autoimmune CSU.

Patients with type IIb autoimmunity experienced improvements in disease control with fenebrutinib across all doses tested. However, at lower fenebrutinib doses, patients with type IIb autoimmunity demonstrated greater benefit than patients without type IIb-associated markers. This finding suggests that patients with type IIb autoimmunity may be more sensitive to BTK inhibition, while patients with type I autoallergy may gain additional clinical benefit from higher levels of BTK inhibition to achieve maximal efficacy. The use of 50 mg daily of fenebrutinib, the lowest dose, may optimize the benefit–risk profile in BHRA^+^ patients, given that CSU typically occurs in otherwise healthy individuals. Potential mechanisms that may contribute to the apparent greater sensitivity of patients with type IIb autoimmunity to BTK inhibition could include: lower density of surface FcεRI;^41^ weaker FcεRI activation by bivalent anti-FcεRI, relative to multivalent autoantigen–IgE complexes;^42^ lower levels of Syk, a BTK-activating kinase in the FcεRI pathway;^43^ and presence of autoantibodies that activate BTK-independent pathways (for example, anti-FcεRII) in patients without type IIb autoimmunity^44,45^.

Fenebrutinib treatment substantially reduced circulating IgG-anti-FcεRI autoantibodies at all doses tested. Greater reductions in IgG-anti-FcεRI were associated with greater decreases in disease activity, probably reflecting a reduction in autoantibody-mediated activation of mast cells and basophils in patients with type IIb autoimmunity. BTK inhibition did not appreciably affect overall serum Ig levels, which are thought to be maintained by long-lived plasma cells^46,47^ that downregulate *BTK* gene expression^48^. Fenebrutinib probably reduced autoantibody production through the inhibition of BCR-mediated differentiation, activation and proliferation of self-reactive, BTK-expressing plasmablasts^49^. Similarly, fenebrutinib treatment of patients with rheumatoid arthritis and lupus resulted in greater proportional reductions in rheumatoid factor^35^ and anti-double-stranded DNA^34^ compared to total IgM or IgG subclasses, respectively. Further studies are needed to determine whether BTK inhibition affects production of other autoantibodies in CSU and their relationship with efficacy.

Although AEs were generally well balanced and mild or moderate, more patients with CSU receiving fenebrutinib than placebo reported urticaria or worsening symptoms of CSU as AEs. However, most events occurred after cessation of treatment, suggesting that relief from CSU symptoms correlated with exposure to fenebrutinib. Nonserious, reversible and asymptomatic serum transaminase elevations occurred in the two higher fenebrutinib dose groups; none of the cases met the definition of severe drug-induced liver injury (Hy’s law)^38^ at any dose. Similarly, a low rate of transient, nonserious transaminase elevations was observed with fenebrutinib in the rheumatoid arthritis and lupus studies^34,35^. Elevations in transaminases have also been observed with other BTK inhibitors, indicating that these results are probably a class effect^50,51^.

Limitations of this trial included the inability to assess fenebrutinib efficacy beyond the eight-week treatment period and lack of data on prior clinical response to omalizumab at baseline for further analysis. In addition, quality of life was not directly measured in this study to complement the UAS7 data. Because the study objectives of characterizing fenebrutinib safety and pharmacokinetics in patients with CSU were met, leading to the early stopping of the study, it is possible that observed efficacy estimates are biased upward. Efficacy in males and non-white populations is unknown because the trial mostly enrolled females from North America and Europe. Additionally, while our results suggested better efficacy in patients with type IIb autoimmunity markers, our analysis was limited because of small numbers of patients in this subset. Although we were unable to directly compare fenebrutinib efficacy with other treatments because of the lack of an active comparator arm, reductions in UAS7 at eight weeks with fenebrutinib treatment (relative to placebo) appear to be similar to omalizumab in phase 3 studies of CSU^52–54^, and the most efficacious doses of ligelizumab, in a recently published phase 2 study^20^. In the future treatment landscape, BTK inhibitors may represent an alternative to anti-IgE therapy, with a particular role in patients with type IIb autoimmunity who are more refractory or slower to respond to anti-IgE therapy. Further studies of fenebrutinib in CSU are not currently planned; however, these data provide an insight into CSU pathophysiology to facilitate future research and development efforts in CSU by Genentech (the trial sponsor). Fenebrutinib is in phase 3 clinical trials for multiple sclerosis.

In conclusion, selective BTK inhibition by fenebrutinib diminished clinical signs and symptoms in patients with CSU refractory to up to fourfold the approved dose of H_1_ antihistamines. Across all doses, even those associated with submaximal BTK inhibition, fenebrutinib resulted in clinical benefit for patients with CSU with type IIb autoimmunity, a population that is more refractory to currently available treatments. These findings highlight the potential for treatments that target BTK to address alternate routes of mast cell activation in diseases such as CSU.

## Methods

### Study design

This phase 2, multicenter, randomized, double-blind, placebo-controlled study was conducted at 21 centers in Canada, Germany and the US. Screening started 1 May 2017 in Canada, the first patient was enrolled on 26 May 2017, the last patient last visit was on 25 October 2019 and the database was locked on 13 December 2019. Standard site outreach and physician referrals were utilized for patient recruitment. Patients were identified from 21 dermatology, allergy and clinical centers in Germany, Canada and the US. Potentially eligible patients were invited to take part in the study.

Nonbinding interim analyses with prespecified decision criteria were preplanned for both cohorts 1 and 2; preplanned adaptations were the amendment of the study to add cohort 2 (cohort 1 interim analysis) and stopping enrollment (cohort 2). Dosing regimens (Extended Data Fig. 1) were selected using pharmacokinetic/pharmacodynamic modeling based on studies in healthy individuals^33^ and were associated with plasma concentrations projected to achieve BTK inhibition of approximately 70–90%. Patients were permitted to use a single approved dose of loratadine (10 mg maximum) or cetirizine (10 mg maximum) within a 24-h period as rescue medication if symptoms worsened.

The study was originally designed as a pilot study to enable initial assessment of the clinical efficacy in CSU. The 200 mg dose was selected because it was expected to be well-tolerated and substantially inhibit BTK activity, based on results from the phase 1 studies. On the basis of the pharmacokinetic and pharmacokinetic/pharmacodynamic models constructed using data from relative bioavailability, and the phase 1 studies, the 200 mg twice daily dose was expected to provide a steady-state exposure achieving 90% maximal inhibitory concentration over the entire dosing interval in >75% of patients. The extent of target engagement required for clinical efficacy was unknown. Based on results from an interim analysis of the pilot study (cohort 1), a dose-ranging cohort (cohort 2) was initiated. Because the extent of target engagement required for clinical efficacy was unknown, doses for cohort 2 were selected to evaluate a range of target engagement and characterize the dose– and exposure–response relationships for safety and efficacy to select the optimal dose. As a result, replication was not performed for the 50 mg and 150 mg doses.

The study protocol was approved by central institutional review boards for the US/Canada and Germany (Supplementary Information). The study was conducted according to international guidelines including the Declaration of Helsinki (2013) and Council for International Organizations of Medical Sciences International Ethical Guidelines, Good Clinical Practice guidelines and applicable laws of other countries. Periodic safety reviews and interim analyses were performed by an internal monitoring committee that was unblinded to treatment assignments and did not have direct contact with patients, investigational staff or site monitors. The internal monitoring committee included representatives from clinical science, drug safety, biostatistics and statistical programming and analysis.

### Patients

Eligible patients were 18–75 years old, had CSU for ≥6 months and were symptomatic despite treatment with H_1_ antihistamines (up to fourfold the approved dose). Key inclusion criteria were: weekly UAS7 ≥ 16, on stable doses of H_1_ antihistamines starting at least 3 consecutive days immediately before the screening visit and continuing through day 1 (≥17 days total); completion of the urticaria patient daily eDiary in the 7 days before randomization; and no evidence of active or latent tuberculosis. Key exclusion criteria were: omalizumab treatment for CSU within four months before screening or primary nonresponse to omalizumab; other disease with wheals or angioedema; other itch-associated skin disease; routine systemic or topical corticosteroids or cyclosporine use; prior use of intravenous glucocorticoids for treatment of laryngeal angioedema; history of anaphylactic shock without clearly identifiable avoidable antigen; active infection; live or attenuated vaccine use; and any condition possibly affecting oral drug absorption. All patients provided written, informed consent. Patients were not compensated for participation in the trial.

### Randomization and masking

Genentech provided the specifications for an interactive voice/web-based response system (IxRS) with a stratified permuted blocks randomization scheme with stratification by country. The IxRS randomly allocated patients to each of four treatment arms (approximately 1:1:1:1; cohort 2) or to 200 mg oral twice daily of fenebrutinib or placebo (approximately 2:1; cohort 1). Genentech provided 50 mg fenebrutinib and matching placebo tablets.

Patients and study site personnel were blinded to individual treatment assignments throughout the study. Results of assessments that might have unblinded investigators to patient treatments, other than local standard and safety laboratory data, were not provided to site staff. During the trial, Genentech personnel, except for members of the IMC, monitored blinded clinical and safety data and had access to unblinded data if needed for safety evaluations. The internal monitoring committee was also responsible for reviewing the results of the preplanned interim analyses.

### Procedures

Patients received complete physical examinations at the day −14 visit and at the safety follow-up visit on day 85 or 4 weeks after the last dose, if the patient discontinued the study and/or study treatment. All patients took fenebrutinib and/or placebo tablets orally twice daily (every 12 h) to maintain blinding, with morning fenebrutinib doses administered in the clinic on days 1, 8 and 29, after all predose assessments. Mandatory morning visits occurred on days 1, 8, 29, 57 and 85. The last dose was taken on day 56.

Patients completed a daily electronic diary (eDiary) comprising questions regarding largest hive size, sleep interference score, activity interference, rescue medication use, angioedema episodes, number of calls to a doctor or nurse practitioner and study medication compliance. The eDiary was completed twice per day (morning and evening) during the study and was used to calculate the UAS7. The UAS is a composite score with numeric severity intensity ratings (0 = none to 3 = intense/severe) for (1) the number of wheals (hives) and (2) the intensity of the pruritus (itch) over the prior 12 h. The daily UAS equals the average of morning and evening scores. The UAS7 is the weekly sum of the daily UAS (maximum value of 42). The urticaria control test (UCT), a four-item questionnaire, assesses disease activity. The recall period is 4 weeks and the score range is 0–16 with an MID of 2.8 (ref. ^55^). Patients completed the UCT at baseline (day 1) and on days 29, 57 and 85 for both cohorts.

For biomarker assessments, serum samples were collected at screening and on days 1, 57 and 85. For details on the BHRA and IgG-anti-FcεRI assays, see the Supplementary Information.

### End points

The primary end point was change from baseline in the UAS7 at week 8. Secondary end points were change from baseline in UAS7 at week 4 and proportion of patients well-controlled (UAS7 ≤ 6) at week 8. Other exploratory efficacy end points included proportion of patients well-controlled at week 4, change from baseline in weekly itch score at weeks 4 and 8, change from baseline in weekly hive score at weeks 4 and 8, proportion of patients who achieved complete response (UAS7 = 0) at weeks 4 and 8, proportion of patients achieving the MID in UAS7 (reduction from baseline UAS7 ≥ 11)^56^ at weeks 4 and 8 and time to achieving MID in UAS7. Safety end points were the incidence and severity of AEs, and changes in vital signs, physical examination findings, electrocardiograms and clinical laboratory results.

### BHRA

The BHRA was measured using sera collected at screening or week 0 for each patient by Viracor Eurofins Clinical Diagnostics, as described previously by Cho and colleagues^28^. The BHRA was not measured for two patients due to sample unavailability.

Percentage histamine release was defined as: HR_sample_/HR_total_ × 100, where HR_sample_ is the histamine released by donor basophils in response to buffer, patient or healthy control serum, or anti-IgE as a positive control, and HR_total_ is the total histamine in lysed donor basophils. The BHRA values were defined as: %HR_sample_/%HR_healthy_ × 10, where %HR_sample_ is the percentage histamine release in response to patient serum and %HR_healthy_ is the mean percentage histamine release plus 2 s.d. in response to a panel of 5 healthy control serum samples. Samples with values ≥10 were considered BHRA^+^.

### IgG-anti-FcεRI ELISA

Recombinant human FcεR1a (rhFCER1A; Val26-Gln205 with a C-terminal 6-His tag; R&D Systems) was used to coat ELISA plates (Nunc MaxiSorp) at 2 µg ml^−1^ in PBS at 4 °C (for 18–72 h). On the day of the assay, plates were washed and blocked with ChonBlock Blocking/Sample Dilution Buffer (Chondrex) on a plate shaker for 2 h at room temperature. All washes in the protocol were as follows: three times with wash buffer (PBS, 0.05% Tween 20). Serum samples from patients with CSU were diluted 1:50 in ChonBlock Blocking/Sample Dilution Buffer, spiked with rhFCER1A or a nonspecific control protein (recombinant human PD-1; Leu25-Thr168 with a C-terminal 6-His tag; R&D Systems) at a final concentration of 2.5 µg ml^−1^ and incubated on a plate shaker for 1 h at room temperature. A mouse IgG × FCER1A (Abcam) standard curve (6-point, threefold dilution series, 0.014–10 ng ml^−1^) was prepared in ChonBlock Blocking/Sample Dilution Buffer. Plates were washed, samples and standards were added to the wells (in duplicate) and plates were incubated on a plate shaker for 2 h at room temperature. Goat × mouse IgG (minimal cross-reactivity), horseradish peroxidase (BioLegend) and AffiniPure Goat Anti-Human IgG-Fcγ (minimum cross-reactivity) (Jackson ImmunoResearch) were diluted in ChonBlock Detection Antibody Dilution Buffer (Chondrex) (1:8,000 and 1:10,000, respectively). Plates were washed, secondary antibodies were added to the appropriate wells and plates were incubated on a plate shaker for 1 h at room temperature. Plates were washed, 3,3′,5,5′-tetramethylbenzidine substrate solution (SeraCare) was added to all wells and plates were incubated on a plate shaker for 5–10 min at room temperature. The substrate reaction was stopped by adding 1 M of phosphoric acid and absorbance (450 and 630 nm) was read on a spectrophotometric plate reader (Molecular Devices VersaMax).

For each well, the 630 nm reading was subtracted from the 450 nm reading. Nonlinear regression curve fitting of the mouse IgG × FCER1A standard was performed and the calculated concentration of each serum sample (ng ml^−1^) was determined using Prism v.6 (GraphPad Software). Relative IgG-anti-FcεRI reactivity was determined by subtracting the sample concentrations from serum preincubated with rhFCER1A from the same serum sample preincubated with rhPD-1. For each experiment, the relative IgG-anti-FcεRI reactivity was determined for a set of 16 healthy volunteer samples and the mean + 3 s.d. was defined as the upper normal range (UNR) threshold, which varied slightly between experiments. Values ≤UNR were considered IgG-anti-FcεRI^−^ and values >UNR were considered IgG-anti-FcεRI^+^. To calculate the percentage change from baseline (in patients who were IgG-anti-FcεRI^+^ at baseline), values <UNR were set to the UNR and the following formula was used: RR(week 8) − RR(Baseline)/RR(Baseline) × 100, where RR is the relative IgG-anti-FcεRI reactivity for a given patient sample.

### Statistical analysis

The purpose of this phase 2 study was initial efficacy estimation and hypothesis generation. Assuming an s.d. of 13, a two-sided alpha level of 0.10 and a 10% dropout rate at week 8, we estimated that a sample size of 30 patients per arm provided approximately 90% power to detect an 11-point difference in the UAS7 change from baseline at week 8 between treatment groups. No adjustment of sample size based on the results of nonbinding interim analysis took place.

Efficacy analyses were conducted for all patients who received at least one dose of study treatment with patients grouped according to treatment assigned at randomization (that is, the modified intent-to-treat population); safety analyses were conducted for the safety population, with patients grouped according to actual treatment received. No participants were excluded from analysis. An internal monitoring committee periodically reviewed safety and performed interim analyses. Preplanned interim efficacy and safety analyses were specified in the protocol (Supplementary Information). Biomarker analyses were prespecified in a separate biomarker analysis plan (Supplementary Information) before the interim analysis readout.

A nonbinding interim analysis was planned and conducted for the time point once 80 patients in cohort 2 had efficacy assessments at week 4. Due to the hypothesis-generating nature of the study, no adjustment was made for sample size at the planning stage of the study to account for the interim look; no adjustment for confidence intervals (CIs) or *P* values obtained in the analysis was implemented. The decision criteria for the efficacy parameters were established using a normal Bayesian model to assess the posterior probability of seeing an equal or higher than prespecified effect size in a future phase 3 CSU trial assuming mean parameter density distribution calculated from the non-informative prior and the phase 2b interim analysis data and known s.d. Cutoffs were based on the chances of meeting an efficacy threshold in phase 3 of >60% (go) or <25% (no go).

Differences between fenebrutinib and placebo groups for continuous efficacy end points, including the primary end point, were analyzed using a mixed model for repeated measures (MMRM) with an unstructured covariance pattern for within-participant errors. Restricted maximum likelihood with the Kenward–Roger approximation for the denominator d.f. was used for the parameter estimates. The MMRM method assumes that data are missing at random^57^. High correlation between successive observations on an individual allows data from individuals who dropped out to contribute to the estimation of the effects at the final time point. All MMRM models included country, treatment group, visit and visit by treatment group interaction as covariates. As sensitivity analyses, analysis of covariance models were fitted with missing week 8 data imputed by last observation carried forward and best observation carried forward. Categorical end points were summarized as proportions; patients who discontinued treatment before the reporting time point were considered nonresponders. Kaplan–Meier estimates were used to analyze time-to-event end points.

The changes from baseline in UAS7 versus placebo are reported for the BHRA subsets as least squares mean differences based on the same model used for the primary analysis but including the BHRA subset indicator and visit-by-arm-by-BHRA interaction. A Spearman correlation was used to assess the relationship between percentage change from baseline in IgG-anti-FcεRI versus change from baseline in UAS7.

No multiple comparison adjustments were made and point estimates with unadjusted 95% CIs are reported. Statistical analyses were performed using SAS v.9.4 (SAS Institute) and R v.3.6.1 (https://www.r-project.org/).

### Reporting Summary

Further information on research design is available in the Nature Research Reporting Summary linked to this article.

## Online content

Any methods, additional references, Nature Research reporting summaries, source data, extended data, supplementary information, acknowledgements, peer review information; details of author contributions and competing interests; and statements of data and code availability are available at 10.1038/s41591-021-01537-w.

## Supplementary information

Supplementary Information List of IRBs, supplementary results, biomarker analysis plan, original protocol, final protocols and summary of protocol amendments.

Reporting Summary

## Data Availability

The study protocol and biomarker analysis plan are provided in the Supplementary Material. As per the Roche Global Policy on Sharing of Clinical Study Information, Roche supports data sharing with qualified investigators engaged in rigorous, independent scientific research. The data for this study were available as of October 2020. Access to Roche’s de-identified patient-level data is facilitated through the cross-industry request Vivli site at https://vivli.org. Requests for access to Roche data are made through the Vivli process and supported by a research proposal that is assessed by an independent review panel managed by the Wellcome Trust. The panel considers the scientific merit of each application. This independent group then decides whether or not the data should be provided. On average it takes a few months to access data in the Vivli platform but the timeline will vary depending on the number of data contributors, the number of studies and your availability to respond to comments. Analyses performed on the data must be in line with the purpose outlined in the research proposal and be approved by the independent review panel. The mechanisms for how data will be made available on the platform are outlined on the Vivli website (https://vivli.org/about/data-request-review-process/). The Vivli secure research environment allows research teams to access data and conduct analyses in a shared workspace that is equipped with analytical tools and may be flexibly configured. The download of Roche anonymized patient-level data from the secure environment is not permitted. For further restrictions and information, please visit https://vivli.org. Further details on Roche’s criteria for eligible studies are available at https://vivli.org/members/ourmembers/. Further details on Roche’s Global Policy on the Sharing of Clinical Information and how to request access to related clinical study documents can be accessed at https://go.gene.com/datasharing.
