# Supplementary material for: Fenebrutinib in H1 antihistamine-refractory chronic spontaneous urticaria: a randomized phase 2 trial
Source: Nat Med. 2021 Nov 8;27(11):1961–9. doi: 10.1038/s41591-021-01537-w (PMC8604722; doi:10.1038/s41591-021-01537-w)
Supplement: Supplementary file 1 — List of IRBs, supplementary results, biomarker analysis plan, original protocol, final protocols and summary of protocol amendments. [file 41591_2021_1537_MOESM1_ESM.pdf]

nature portfolio

https://doi.org/10.1038/s41591-021-01537-w

Supplementary information

Fenebrutinib in H_1 antihistamine-refractory chronic spontaneous urticaria: a randomized phase 2 trial

In the format provided by the authors and unedited

Supplementary Information

Fenebrutinib, in H1-Antihistamine-Refractory Chronic Spontaneous Urticaria: A Randomized Phase 2 Trial

Martin Metz, Gordon Sussman, Rémi Gagnon, Petra Staubach, Tonny Tanus, William H. Yang, Jeremy J. Lim, Holly J. Clarke, Joshua Galanter, Leslie W. Chinn, Tom Chu, Anastasia Teterina, Tracy Burgess, D. James Haddon, Timothy T. Lu, Marcus Maurer

Table of Contents

List of Institutional Review Boards 2
Supplementary Results
Supplementary Table 1. Patient numbers. 3
Supplementary Table 2. Baseline characteristics of patients in Cohort 1 (modified intention-to-treat population) 5
Supplementary Table 3. Efficacy endpoints in Cohort 1 (modified intention-to-treat population) 6
Supplementary Table 4. AEs by system organ class and preferred term (safety population) in Cohort 2. 7
Supplementary Table 5. Overview of AEs (safety population) in Cohort 1 11
Supplementary Table 6. AEs by system organ class and preferred term (safety population) in Cohort 1 12
Supplementary Table 7. Summary of liver transaminases by lab grade in Cohort 2 15
Supplementary Table 8. Summary of liver transaminases by lab grade in Cohort 1 16
Safety: ALT/AST abbreviated patient narratives 17
Biomarker Analysis Plan 18
Original Protocol (Version 1), including Statistical Considerations and Analysis Plan (Section 6) 22
Final Protocols: Version 5 (Germany) and Version 5 (Canada), including Statistical Considerations and Analysis Plan (Section 6) 123
Summary of Protocol Amendments (including changes to Statistical Considerations and Analysis Plan 352

List of Institutional Review Boards

Region	Site #	Principal Investigator	Institutional Review Board (IRB)	IRB Approval Date
Canada	301899	Sussman, Gordon	Advarra<br>6940 Columbia Gateway Drive, Suite 110<br>Columbia, MD 21046 USA	17-Feb-2017
Canada	301955	Gagnon, Remi	Advarra	17-Feb-2017
Germany	301958	Maurer, Marcus	Landesamt für Gesundheit und Soziales Geschäftsstelle der Ethik-Kommission des Landes Berlin<br>Turmstraße 21, Haus A<br>10559 Berlin, Germany	30-May-2017
Germany	301959	Staubach-Renz, Petra	Landesamt für Gesundheit und Soziales Geschäftsstelle der Ethik-Kommission des Landes	30-May-2017
Canada	309948	Cheema, Amarjit	Advarra	15-Dec-2017
Germany	314019	Bauer, Andrea	Landesamt für Gesundheit und Soziales Geschäftsstelle der Ethik-Kommission des Landes	06-Jun-2018
Germany	314020	Brehler, Randolf	Landesamt für Gesundheit und Soziales Geschäftsstelle der Ethik-Kommission des Landes	04-Jun-2018
United States	314060	Tanus, Tonny	Advarra	27-Apr-2018
Canada	314062	Yang, William	Advarra	29-May-2018
United States	314093	Shah-Patel, Heena	Advarra	30-Oct-2018
Germany	314118	Popp, Georg	Landesamt für Gesundheit und Soziales Geschäftsstelle der Ethik-Kommission des Landes	06-Jun-2018
Germany	314135	Sebastian, Michael	Landesamt für Gesundheit und Soziales Geschäftsstelle der Ethik-Kommission des Landes	04-Jun-2018
Canada	314596	Lynde, Charles	Advarra	06-Jun-2018
United States	314974	Lipson, Brian	Advarra	06-Jul-2018
United States	315185	Kent, Jr., Edward	Advarra	18-Jun-2018
United States	315457	Soong, Weily	Advarra	14-Aug-2018
United States	315591	Settipane, Russell	Advarra	10-Aug-2018
United States	315923	Hussain, Iftikhar	Advarra	08-Aug-2018
United States	316325	Manning, Michael	Advarra	07-Aug-2018
United States	317045	Johnson II, Thomas	Advarra	14-Aug-2018
United States	317104	Berger, William	Advarra	20-Sep-2018

Supplementary Table 1. Patient numbers.

Patient numbers for Figure 2a-c.

Arm	Cohort	Week 1	Week 2	Week 3	Week 4	Week 5	Week 6	Week 7	Week 8	Week 8	Week 10	Week 11	Week 12
GDC-0853 150 mg QD	2	23	23	23	23	23	23	23	22	17	17	17	15
GDC-0853 200 mg BID	2	23	22	22	21	22	21	21	21	15	14	13	11
GDC-0853 50 mg QD	2	23	22	20	19	19	19	19	19	11	12	11	10
Placebo BID	2	23	23	23	21	20	20	20	20	16	15	13	14

Arm	Cohort	Week 1	Week 2	Week 3	Week 4	Week 5	Week 6	Week 7	Week 8	Week 8	Week 10	Week 11	Week 12
GDC-0853 200 mg BID	1	28	28	25	24	24	22	22	22	21	20	21	20
Placebo BID	1	13	13	13	13	13	13	13	12	12	12	12	12

Patient numbers for Extended Data Figure 5a.

Arm	Group	Pt Number
GDC-0853 200 mg BID	All patients	23
	BHRA+ (`CU Index ≥10`)	10
	BHRA- (`CU Index <10`)	12
GDC-0853 150 mg QD	All patients	23
	BHRA+ (`CU Index ≥10`)	10
	BHRA- (`CU Index <10`)	12
GDC-0853 50 mg QD	All patients	23
	BHRA+ (`CU Index ≥10`)	9
	BHRA- (`CU Index <10`)	14
Placebo BID	All patients	23
	BHRA+ (`CU Index ≥10`)	9
	BHRA- (`CU Index <10`)	14

Patient numbers for Extended Data Figure 5b.

Arm	Group	Pt Number
GDC-0853 200 mg BID	All patients	23
	`IGE ≥43 IU/ml`	16
	`IGE <43 IU/ml`	7
GDC-0853 150 mg QD	All patients	23
	`IGE ≥43 IU/ml`	11
	`IGE <43 IU/ml`	11
GDC-0853 50 mg QD	All patients	23
	`IGE ≥43 IU/ml`	17

	`IGE <43 IU/ml`	6
Placebo BID	All patients	23
	`IGE ≥43 IU/ml`	12
	`IGE <43 IU/ml`	11

Patient numbers for Extended Data Figure 7a.

**Arm**	**Group**	**Pt Number**
GDC-0853 200 mg BID	All patients	28
	BHRA+ (`CU Index ≥10`)	12
	BHRA- (`CU Index <10`)	16
Placebo BID	All patients	13
	BHRA+ (`CU Index ≥10`)	8
	BHRA- (`CU Index <10`)	5

Patient numbers for Extended Data Figure 7b.

**Arm**	**Group**	**Pt Number**
GDC-0853 200 mg BID	All patients	28
	`IGE ≥43 IU/ml`	17
	`IGE <43 IU/ml`	11
Placebo BID	All patients	13
	`IGE ≥43 IU/ml`	8
	`IGE <43 IU/ml`	5

Supplementary Table 2. Baseline characteristics of patients in Cohort 1 (modified intention-to-treat population[a]).

Characteristic	Placebo (n=13)	Fenebrutinib 200 mg BID (n=28)	All Patients (N=41)
**Demographic**			
Age, mean (SD), y	43.6 (11.0)	41.3 (15.9)	42.0 (14.4)
Age group, no. (%)			
18-40 years	5 (39)	13 (46)	18 (44)
41-64 years	8 (62)	15 (54)	23 (56)
≥65 years	0	0	0
Female sex, no. (%)	11 (85)	22 (79)	33 (80)
Race, no. (%)			
White	10 (77)	24 (86)	34 (83)
Nonwhite	3 (23)	4 (14)	7 (17)
Weight, mean (SD), kg	84.5 (11.8)	75.5 (17.5)	78.3 (16.3)
Body-mass index, mean (SD), kg/m^2	30.2 (5.0)	27.1 (5.2)	28.1 (5.3)
**Clinical**			
Time since diagnosis of CSU, median (range), y	2.8 (0.5-30.1)	1.5 (0.0-22.3)	1.7 (0.0-30.1)
UAS7, mean (SD)	32.9 (8.1)	29.5 (7.0)	30.5 (7.4)
Weekly itch score, mean (SD)	15.2 (5.2)	12.9 (4.0)	13.6 (4.5)
Weekly hive score, mean (SD)	17.6 (5.5)	16.6 (4.7)	16.9 (4.9)
In-clinic UCT[b], mean (SD)	2.5 (2.3)	4.9 (3.7)	4.2 (3.5)
Presence of angioedema, no. (%)	7 (54)	15 (54)	22 (54)
Rescue medication use, no. (%)	8 (62)	20 (71)	28 (68)
Proportion of rescue medication-free days, mean (SD), %	45.1 (50.8)	48.9 (45.5)	47.7 (46.6)
Total IgE, mean (SD), IU/mL	351.1 (866.4)	150.5 (238.9)	214.1 (522.2)
$BHRA^+$ (≥10)[c], no. (%)	8 (62)	12 (43)	20 (49)

Abbreviations: BHRA, basophil histamine release assay; UCT, urticaria control test.
[a]The modified intention-to-treat population included all patients who had undergone randomization and received at least one dose of a study drug. Percentages may not total 100 because of rounding.
[b]Data are for 11 patients in the placebo group and 27 in the 200 mg BID fenebrutinib group.
[c]BHRA includes Week 0, Day 1 when screening biomarker sample was unavailable.

Supplementary Table 3. Efficacy endpoints in Cohort 1 (modified intention-to-treat population).

Endpoint	Placebo (n=13)	Fenebrutinib 200 mg BID (n=28)
**Primary endpoint**		
UAS7 score, Change from baseline to week 8		
Mean (95% CI)[a]	-17.2 (-23.1, -11.4)	-24.3 (-28.7, -19.9)
LS mean difference for treatment vs. placebo (95% CI)		-7.0 (-14.2, 0.2)
**Secondary endpoints**		
Well controlled patients (UAS7 ≤ 6) at week 8, no. (%)	4 (31)	16 (57)
UAS7 score, Change from baseline to week 4		
Mean (95% CI)[a]	-8.8 (-14.7, -2.8)	-21.6 (-25.9, -17.4)
LS mean difference for treatment vs. placebo (95% CI)		-12.9 (-20.2, -5.6)
**Exploratory endpoints**		
Weekly itch score		
Change from baseline to week 4		
Mean (95% CI)[a]	-4.0 (-6.8, -1.3)	-9.8 (-11.8, -7.8)
LS mean difference for treatment vs. placebo (95% CI)		-5.8 (-9.1, -2.4)
Change from baseline to week 8		
Mean (95% CI)[a]	-8.1 (-10.9, -5.3)	-10.6 (-12.7, -8.6)
LS mean difference for treatment vs. placebo (95% CI)		-2.5 (-6.0, 0.9)
Weekly hives score		
Change from baseline to week 4		
Mean (95% CI)[a]	-4.3 (-7.9, -0.8)	-12.0 (-14.5, -9.5)
LS mean difference for treatment vs. placebo (95% CI)		-7.7 (-12.0, -3.4)
Change from baseline to week 8		
Mean (95% CI)[a]	-9.0 (-12.4, -5.6)	-13.4 (-16.0, -10.8)
LS mean difference for treatment vs. placebo (95% CI)		-4.4 (-8.5, -0.2)
Well controlled patients (UAS7 ≤ 6) at week 4, no. (%)	2 (15)	16 (57)
Patients with complete response (UAS7 = 0), no. (%)		
At week 4	0	12 (42.9%)
At week 8	4 (31)	15 (54)
Patients achieving MID in UAS7 at week 8, no. (%)	8 (62)	20 (71)
Patients achieving MID in weekly itch score at week 8, no. (%)	9 (69.2%)	19 (67.9%)

Abbreviations: CI, confidence interval; LS, least squares; MID, minimally important difference.
[a]Least-squares means estimates from a mixed model for repeated measures (MMRM).

Supplementary Table 4. AEs by system organ class and preferred term (safety population[a]) in Cohort 2.

	Placebo (n=22)	Fenebrutinib 50 mg QD (n=23)	Fenebrutinib 150 mg QD (n=24)	Fenebrutinib 200 mg BID (n=24)	All Patients (N=93)
Total no. of patients with at least one AE, no. (%)	12 (54)	14 (61)	16 (67)	14 (58)	56 (60)
Overall total no. of events	33	38	35	41	147
**MedDRA System Organ Class** **MedDRA Preferred Term**	no. or no. (%)				
**Skin and subcutaneous tissue disorders**					
Total no. of patients with at least one AE	6 (27)	6 (26)	8 (33)	5 (21)	25 (27)
Total no. of events	6	8	10	5	29
Urticaria	2 (9)	3 (13)	4 (17)	5 (21)	14 (15)
Chronic spontaneous urticaria	1 (4)	1 (4)	2 (8)	0	4 (4)
Angioedema	1 (4)	0	1 (4)	0	2 (2)
Alopecia	0	0	1 (4)	0	1 (1)
Dermatitis contact	1 (4)	0	0	0	1 (1)
Erythema	0	1 (4)	0	0	1 (1)
Rash	0	1 (4)	0	0	1 (1)
Rash papular	1 (4)	0	0	0	1 (1)
Urticaria pressure	0	0	1 (4)	0	1 (1)
**Infections and infestations**					
Total no. of patients with at least one AE	4 (18)	4 (17)	6 (25)	7 (29)	21 (23)
Total no. of events	4	5	8	10	27
Nasopharyngitis	1 (4)	3 (13)	3 (12)	3 (12)	10 (11)
Urinary tract infection	0	1 (4)	2 (8)	1 (4)	4 (4)
Upper respiratory tract infection	1 (4)	0	0	2 (8)	3 (3)
Sinusitis	1 (4)	0	1 (4)	0	2 (2)
Bronchitis	0	0	0	1 (4)	1 (1)
Cystitis	0	0	0	1 (4)	1 (1)
Influenza	1 (4)	0	0	0	1 (1)
Skin infection	0	1 (4)	0	0	1 (1)
Viral infection	0	0	0	1 (4)	1 (1)
Viral upper respiratory tract infection	0	0	1 (4)	0	1 (1)
**Nervous system disorders**					
Total no. of patients with at least one AE	3 (14)	5 (22)	3 (12)	5 (21)	16 (17)
Total no. of events	3	5	3	6	17
Headache	2 (9)	0	1 (4)	3 (12)	6 (6)
Hypoesthesia	1 (4)	1 (4)	0	0	2 (2)
Restless legs syndrome	0	1 (4)	1 (4)	0	2 (2)
Burning sensation	0	0	0	1 (4)	1 (1)
Dizziness	0	0	0	1 (4)	1 (1)
Migraine	0	1 (4)	0	0	1 (1)

Supplementary Table 4. AEs by system organ class and preferred term (safety population[a]) in Cohort 2.

	Placebo (n=22)	Fenebrutinib 50 mg QD (n=23)	Fenebrutinib 150 mg QD (n=24)	Fenebrutinib 200 mg BID (n=24)	All Patients (N=93)
Paresthesia	0	1 (4)	0	0	1 (1)
Sciatica	0	1 (4)	0	0	1 (1)
Syncope	0	0	1 (4)	0	1 (1)
**Gastrointestinal disorders**					
Total no. of patients with at least one AE	5 (23)	2 (9)	4 (17)	3 (12)	14 (15)
Total no. of events	7	2	4	3	16
Nausea	0	1 (4)	2 (8)	2 (8)	5 (5)
Diarrhea	2 (9)	0	1 (4)	0	3 (3)
Abdominal discomfort	1 (4)	0	0	1 (4)	2 (2)
Abdominal pain	0	1 (4)	0	0	1 (1)
Abdominal pain upper	0	0	1 (4)	0	1 (1)
Constipation	1 (4)	0	0	0	1 (1)
Gastroesophageal reflux disease	1 (4)	0	0	0	1 (1)
Lip dry	1 (4)	0	0	0	1 (1)
Oral disorder	1 (4)	0	0	0	1 (1)
**General disorders and administration site conditions**					
Total no. of patients with at least one AE	2 (9)	3 (13)	1 (4)	2 (8)	8 (9)
Total no. of events	2	6	1	5	14
Fatigue	2 (9)	0	1 (4)	0	3 (3)
Peripheral swelling	0	1 (4)	0	1 (4)	2 (2)
Chest discomfort	0	1 (4)	0	0	1 (1)
Chest pain	0	0	0	1 (4)	1 (1)
Medical device pain	0	1 (4)	0	0	1 (1)
Medical device site hemorrhage	0	1 (4)	0	0	1 (1)
Edema peripheral	0	1 (4)	0	0	1 (1)
Pyrexia	0	0	0	1 (4)	1 (1)
Swelling	0	0	0	1 (4)	1 (1)
**Investigations**					
Total no. of patients with at least one AE	0	1 (4)	2 (8)	5 (21)	8 (9)
Total no. of events	0	1	3	7	11
Alanine aminotransferase increased	0	0	1 (4)	3 (12)	4 (4)
Aspartate aminotransferase increased	0	0	1 (4)	2 (8)	3 (3)
Blood creatine phosphokinase increased	0	0	0	1 (4)	1 (1)
Blood glucose increased	0	0	1 (4)	0	1 (1)
Creatinine renal clearance decreased	0	1 (4)	0	0	1 (1)
Transaminases increased	0	0	0	1 (4)	1 (1)
**Musculoskeletal and connective tissue disorders**					
Total no. of patients with at least one AE	2 (9)	3 (13)	0	1 (4)	6 (6)

Supplementary Table 4. AEs by system organ class and preferred term (safety population[a]) in Cohort 2.

	Placebo (n=22)	Fenebrutinib 50 mg QD (n=23)	150 mg QD (n=24)	200 mg BID (n=24)	All Patients (N=93)
Total no. of events	4	4	0	1	9
Back pain	1 (4)	1 (4)	0	0	2 (2)
Arthralgia	0	1 (4)	0	0	1 (1)
Flank pain	1 (4)	0	0	0	1 (1)
Joint swelling	0	0	0	1 (4)	1 (1)
Pain in extremity	0	1 (4)	0	0	1 (1)
Spondylolisthesis	0	1 (4)	0	0	1 (1)
**Injury, poisoning and procedural complications**					
Total no. of patients with at least one AE	1 (4)	2 (9)	0	1 (4)	4 (4)
Total no. of events	1	6	0	1	8
Arthropod sting	0	1 (4)	0	0	1 (1)
Contusion	0	0	0	1 (4)	1 (1)
Joint injury	0	1 (4)	0	0	1 (1)
Soft tissue injury	0	1 (4)	0	0	1 (1)
Sunburn	1 (4)	0	0	0	1 (1)
**Respiratory, thoracic and mediastinal disorders**					
Total no. of patients with at least one AE	1 (4)	0	2 (8)	1 (4)	4 (4)
Total no. of events	1	0	2	1	4
Cough	0	0	1 (4)	0	1 (1)
Nasal congestion	1 (4)	0	0	0	1 (1)
Oropharyngeal pain	0	0	0	1 (4)	1 (1)
Snoring	0	0	1 (4)	0	1 (1)
**Psychiatric disorders**					
Total no. of patients with at least one AE	1 (4)	0	1 (4)	1 (4)	3 (3)
Total no. of events	1	0	1	1	3
Abnormal dreams	0	0	0	1 (4)	1 (1)
Depression	1 (4)	0	0	0	1 (1)
Disorientation	0	0	1 (4)	0	1 (1)
**Hepatobiliary disorders**					
Total no. of patients with at least one AE	0	0	2 (8)	0	2 (2)
Total no. of events	0	0	2	0	2
Biliary colic	0	0	1 (4)	0	1 (1)
Hepatic function abnormal	0	0	1 (4)	0	1 (1)
**Blood and lymphatic system disorders**					
Total no. of patients with at least one AE	0	0	0	1 (4)	1 (1)
Total no. of events	0	0	0	1	1
Increased tendency to bruise	0	0	0	1 (4)	1 (1)
**Cardiac disorders**					

Supplementary Table 4. AEs by system organ class and preferred term (safety population[a]) in Cohort 2.

		Fenebrutinib			
	**Placebo (n=22)**	**50 mg QD (n=23)**	**150 mg QD (n=24)**	**200 mg BID (n=24)**	**All Patients (N=93)**
Total no. of patients with at least one AE	0	0	1 (4)	0	1 (1)
Total no. of events	0	0	1	0	1
Tachycardia	0	0	1 (4)	0	1 (1)
**Eye disorders**					
Total no. of patients with at least one AE	1 (4)	0	0	0	1 (1)
Total no. of events	1	0	0	0	1
Cataract	1 (4)	0	0	0	1 (1)
**Reproductive system and breast disorders**					
Total no. of patients with at least one AE	1 (4)	0	0	0	1 (1)
Total no. of events	2	0	0	0	2
Vaginal hemorrhage	1 (4)	0	0	0	1 (1)
**Surgical and medical procedures**					
Total no. of patients with at least one AE	1 (4)	0	0	0	1 (1)
Total no. of events	1	0	0	0	1
Wisdom teeth removal	1 (4)	0	0	0	1 (1)
**Vascular disorders**					
Total no. of patients with at least one AE	0	1 (4)	0	0	1 (1)
Total no. of events	0	1	0	0	1
Hematoma	0	1 (4)	0	0	1 (1)

Abbreviations: AE, adverse event.

[a]The safety population was defined according to the treatment actually received.

AEs were coded using MedDRA (Medical Dictionary for Regulatory Activities), version 22.1. For frequency counts by preferred term, multiple occurrences of the same AE in an individual were counted only once. For frequency counts of total number of events rows, multiple occurrences of the same AE in an individual were counted separately.

Supplementary Table 5. Overview of AEs (safety population[a]) in Cohort 1.

Event	Placebo (n=13)	Fenebrutinib 200 mg BID (n=28)	All Patients (N=41)
Patients with at least one AE, no. (%)	8 (62)	20 (71)	28 (68)
Overall total no. of AEs	16	58	74
Any AE leading to discontinuation of study drug, no. (%)	0	4 (14)	4 (10)
Early withdrawal from study due to an AE, no. (%)	0	3 (11)	3 (7)
Any SAE, no. (%)	0	3 (11)	3 (7)
Death, no. (%)	0	0	0
Any AE suspected to be caused by study drug, no. (%)	3 (23)	13 (46)	16 (39)
Any severe AE, no. (%)	0	8 (28)	8 (20)

Abbreviations: AE, adverse event. SAE, serious adverse event.
[a]The safety population was defined according to the treatment actually received.

Supplementary Table 6. AEs by system organ class and preferred term (safety population[a]) in Cohort 1.

	Placebo (n=13)	Fenebrutinib 200 mg BID (n=28)	All Patients (N=41)
Total no. of patients with at least one AE, no. (%)	8 (62)	20 (71)	28 (68)
Overall total no. of events	16	58	74
**MedDRA System Organ Class**<br>**MedDRA Preferred Term**		no. or no. (%)	
**Infections and infestations**			
Total no. of patients with at least one AE	5 (38)	10 (36)	15 (37)
Total no. of events	5	17	22
Nasopharyngitis	3 (23)	7 (25)	10 (24)
Eye infection	1 (8)	0	1 (2)
Gastrointestinal infection	0	1 (4)	1 (2)
Oral herpes	0	1 (4)	1 (2)
Periorbital cellulitis	0	1 (4)	1 (2)
Pharyngitis	0	1 (4)	1 (2)
Sinusitis	0	1 (4)	1 (2)
Tonsillitis	0	1 (4)	1 (2)
Tooth infection	1 (8)	0	1 (2)
Upper respiratory tract infection	0	1 (4)	1 (2)
**Nervous system disorders**			
Total no. of patients with at least one AE	4 (31)	7 (25)	11 (27)
Total no. of events	4	8	12
Headache	3 (23)	4 (14)	7 (17)
Dizziness	1 (8)	2 (7)	3 (7)
Migraine	0	1 (4)	1 (2)
**Skin and subcutaneous tissue disorders**			
Total no. of patients with at least one AE	0	8 (29)	8 (20)
Total no. of events	0	9	9
Urticaria	0	5 (18)	5 (12)
Chronic spontaneous urticaria	0	2 (7)	2 (5)
Angioedema	0	1 (4)	1 (2)
**Gastrointestinal disorders**			
Total no. of patients with at least one AE	0	7 (25)	7 (17)
Total no. of events	0	7	7
Nausea	0	2 (7)	2 (5)
Abdominal pain	0	1 (4)	1 (2)
Abdominal pain upper	0	1 (4)	1 (2)
Constipation	0	1 (4)	1 (2)
Diarrhea	0	1 (4)	1 (2)
Dry mouth	0	1 (4)	1 (2)

Supplementary Table 6. AEs by system organ class and preferred term (safety population[a]) in Cohort 1.

	Placebo (n=13)	Fenebrutinib 200 mg BID (n=28)	All Patients (N=41)
**Investigations**			
Total no. of patients with at least one AE	1 (8)	3 (11)	4 (10)
Total no. of events	1	5	6
Alanine aminotransferase increased	0	2 (7)	2 (5)
Aspartate aminotransferase increased	0	2 (7)	2 (5)
Hepatic enzyme increased	0	1 (4)	1 (2)
Weight decreased	1 (8)	0	1 (2)
**General disorders and administration site conditions**			
Total no. of patients with at least one AE	2 (15)	1 (4)	3 (7)
Total no. of events	2	1	3
Chills	1 (8)	0	1 (2)
Fatigue	0	1 (4)	1 (2)
Feeling abnormal	1 (8)	0	1 (2)
**Injury, poisoning and procedural complications**			
Total no. of patients with at least one AE	1 (8)	2 (7)	3 (7)
Total no. of events	2	2	4
Contusion	0	2 (7)	2 (5)
Bone contusion	1 (8)	0	1 (2)
Injury	1 (8)	0	1 (2)
**Musculoskeletal and connective tissue disorders**			
Total no. of patients with at least one AE	1 (8)	2 (7)	3 (7)
Total no. of events	1	2	3
Arthralgia	0	1 (4)	1 (2)
Back pain	1 (8)	0	1 (2)
Musculoskeletal stiffness	0	1 (4)	1 (2)
**Psychiatric disorders**			
Total no. of patients with at least one AE	0	2 (7)	2 (5)
Total no. of events	0	2	2
Initial insomnia	0	1 (4)	1 (2)
Insomnia	0	1 (4)	1 (2)
**Respiratory, thoracic and mediastinal disorders**			
Total no. of patients with at least one AE	0	2 (7)	2 (5)
Total no. of events	0	2	2
Asthma	0	1 (4)	1 (2)
Nasal dryness	0	1 (4)	1 (2)
**Cardiac disorders**			
Total no. of patients with at least one AE	0	1 (4)	1 (2)
Total no. of events	0	1	

Supplementary Table 6. AEs by system organ class and preferred term (safety population[a]) in Cohort 1.

	Placebo (n=13)	Fenebrutinib 200 mg BID (n=28)	All Patients (N=41)
Cardiovascular disorder	0	1 (4)	1 (2)
**Eye disorders**			
Total no. of patients with at least one AE	1 (8)	0	1 (2)
Total no. of events	1	0	1
Vision blurred	1 (8)	0	1 (2)

Abbreviations: AE, adverse event.
[a]The safety population was defined according to the treatment actually received.
AEs were coded using MedDRA (Medical Dictionary for Regulatory Activities), version 22.1. For frequency counts by preferred term, multiple occurrences of the same AE in an individual were counted only once. For frequency counts of total number of events rows, multiple occurrences of the same AE in an individual were counted separately.

Supplementary Table 7. Summary of liver transaminases by lab grade in Cohort 2.

	NCI-CTCAE Grade[a]	Placebo (n=22)	Fenebrutinib 50 mg QD (n=23)	Fenebrutinib 150 mg QD (n=24)	Fenebrutinib 200 mg BID (n=24)
		no. (%)			
AST	normal	16 (73)	23 (100)	18 (75)	17 (71)
	Grade 1	6 (27)		5 (21)	5 (21)
	Grade 2				
	Grade 3			1 (4)	2 (8)
ALT	normal	16 (73)	22 (96)	18 (75)	15 (62)
	Grade 1	6 (27)	1 (4)	4 (17)	8 (33)
	Grade 2			1 (4)	
	Grade 3			1 (4)	1 (4)
AST or ALT	normal	14 (64)	21 (91)	17 (71)	14 (58)
	Grade 1	8 (36)	1 (4)	5 (21)	8 (33)
	Grade 2			1 (4)	
	Grade 3			1 (4)	2 (8)

Abbreviations: AST, aspartate aminotransferase. ALT, alanine aminotransferase; NCI-CTCAE, National Cancer Institute-Common Terminology Criteria for Adverse Events; ULN, upper limit of normal.
[a]NCI-CTCAE grades: normal (≤ULN); Grade 1 (>ULN–3.0 x ULN); Grade 2 (>3.0–5.0 x ULN); Grade 3 (>5.0–20.0 x ULN).

Supplementary Table 8. Summary of liver transaminases by lab grade in Cohort 1.

	NCI-CTCAE Grade[a]	Placebo (n=13)	Fenebrutinib 200 mg BID (n=28)
		no. (%)	
AST	normal	13 (100)	20 (71)
	Grade 1		6 (21)
	Grade 2		1 (4)
	Grade 3		2 (7)
ALT	normal	11 (85)	18 (64)
	Grade 1	2 (15)	9 (32)
	Grade 2		
	Grade 3		3 (11)
AST or ALT	normal	11 (85)	18 (64)
	Grade 1	2 (15)	9 (32)
	Grade 2		
	Grade 3		3 (11)

Abbreviations: AST, aspartate aminotransferase; ALT, alanine aminotransferase; NCI-CTCAE, National Cancer Institute-Common Terminology Criteria for Adverse Events; ULN, upper limit of normal.

[a]NCI-CTCAE grades: normal (≤ULN); Grade 1 (>ULN–3.0 x ULN); Grade 2 (>3.0–5.0 x ULN); Grade 3 (>5.0–20.0 x ULN).

Safety: ALT/AST abbreviated patient narratives

Cohort 1 - 200 mg BID arm

Patient 1 was noted to have an elevated ALT (601 U/L; normal range, 6 to 34 U/L) and AST (429 U/L; normal range,: 9 to 34 U/L), and a normal bilirubin level (9 µmol/L; normal range, 3 to 21 µmol/L) on study day 29. Study treatment was discontinued on Day 30. Repeat testing on Day 35 confirmed the aminotransferase elevations (ALT 677 U/L, AST 319 U/L) and normal bilirubin (7 µmol/L). On follow-up, on Day 57, her transaminases normalized (ALT 23 U/L, AST 16 U/L, bilirubin 9 µmol/L).

Patient 2 was noted to have an elevated ALT (85 U/L; normal range, 6 to 34 U/L) and AST (53 U/L; normal range, 9 to 34 U/L), and a normal bilirubin level (7 µmol/L; normal range, 3 to 21 µmol/L) on study day 29. On Day 64, her aminotransferase elevations were more markedly elevated (ALT 286 U/L, AST 197 U/L) and her bilirubin remained normal (9 µmol/L), and she completed treatment. On follow-up, on Day 98, her transaminases improved (ALT 186 U/L, AST 89 U/L, bilirubin 9 µmol/L).

Patient 3 was noted to have an elevated ALT (272 U/L; normal range, 6 to 34 U/L) and AST (152 U/L; normal range, 9 to 34 U/L), and a normal bilirubin level (8 µmol/L; normal range, 3 to 21 µmol/L) on study day 56, when she completed treatment. At an unscheduled visit on Day 65, her aminotransferases had significantly improved (ALT 38 U/L, AST 18 U/L) and bilirubin remained normal (4 µmol/L). On follow-up, on Day 85, her transaminases normalized (ALT 15 U/L, AST 17 U/L, bilirubin 7 µmol/L).

Cohort 2 - 200 mg BID arm

Patient 4 was noted to have an elevated ALT (582 U/L; normal range, 6 to 34 U/L) and AST (243 U/L; normal range, 9 to 34 U/L), and a normal bilirubin level 21 µmol/L; normal range, 3 to 21 µmol/L) on study day 59. She was enrolled into a separate open-label extension (OLE) study, and she continued treatment with open-label fenebrutinib 200 mg BID. Her transaminase elevations were resolved on OLE Day 29 (ALT 18 U/L, AST 15 U/L, bilirubin 10). She was discontinued from the open-label study on OLE Day 48, with normal aminotransferases and bilirubin (ALT 15 U/L, AST 13 U/L, bilirubin 7 µmol/L).

Patient 5 was noted to have an elevated ALT (104 U/L; normal range, 6 to 43 U/L) and AST (236 U/L; normal range, 11 to 36 U/L), and a normal bilirubin level (12 µmol/L; normal range, 3 to 21 µmol/L) on study day 31. Study treatment was discontinued on Day 34. Repeat testing on Day 37 confirmed the aminotransferase elevations (ALT 119 U/L, AST 70 U/L) and normal bilirubin (12 µmol/L). His transaminases were normalizing on day 45 (ALT 48 U/L, AST 23 U/L, bilirubin 17 µmol/L), and on follow-up, on Day 73, his transaminases nearly normalized (ALT 49 U/L, AST 28 U/L, bilirubin 14 µmol/L).

Cohort 2 - 150 mg QD arm

Patient 6 was noted to have an elevated ALT (492 U/L; normal range, 6 to 32 U/L) and AST (360 U/L; normal range, 9 to 34 U/L), and a normal bilirubin level (14 µmol/L; normal range, 3 to 21 µmol/L) on study day 56, when she completed treatment. Upon repeat testing on Day 70, her aminotransferase elevations were improving (ALT 70 U/L, AST 49 U/L) and her bilirubin remained normal (10 µmol/L). On follow-up, on Day 86, her transaminases were nearly normal (ALT 30 U/L, AST 35 U/L, bilirubin 10 µmol/L). The patient was inadvertently enrolled in the separate open-label extension study 59 days later and resumed dosing with 200 mg BID of open-label fenebrutinib. She again developed aminotransferase elevations (ALT 351 U/L, AST 347 U/L, bilirubin 10 µmol/L) on Day 22 of that study. On Day 40 of the OLE study, her aminotransferases nearly normalized (ALT 58 U/L, AST 44 U/L, bilirubin 15 µmol/L) and treatment was discontinued.

Biomarker Analysis Plan

**BHRA predictive biomarker hypothesis**	In fenebrutinib-treated patients, the mean reduction in UAS7 from Baseline to Weeks 4 and 8 will be greater in BHRA$^+$ patients than in BHRA$^–$ patients. Fenebrutinib is predicted to inhibit IgG-anti-FcεRI and anti-IgE in BHRA$^+$ patients. Preliminary Phase 2a results suggest fenebrutinib may have less efficacy in BHRA$^–$ patients (versus BHRA$^+$ patients).
BHRA	● Viracor will perform BHRA measurements on Screening visit serum samples<br>● If a Screening visit serum sample is not available for BHRA measurement, the Baseline visit (Day 1 pre-dose) sample may be used in its place<br>● BHRA values <10 are considered negative and values ≥10 are considered positive[23]
Efficacy	● Mean change in UAS7 from Baseline to Weeks 4 and 8 will be the primary measures of efficacy<br>● Differences between BHRA$^-$ and BHRA$^+$ groups greater than or equal to the UAS7 minimally important difference (MID: 9.5–10.5) will be considered clinically meaningful[55]
Patients	● This analysis will use the same patients and visits used in the UAS7 topline efficacy readout<br>● If Screening and Baseline (Day 1 pre-dose) visit serum samples are not available for BHRA measurement for a given patient, they will be excluded from the analysis
Biostats outputs	● Tables of descriptive statistics of mean change in UAS7 from Baseline to Weeks 4 and 8 for BHRA$^–$ and BHRA$^+$ patient groups within each fenebrutinib arm (200 mg BID, 150 mg QD or 50 mg QD) as well as the placebo arm<br>● Number of BHRA$^+$ and BHRA$^–$ patients in each arm at Screening<br>● Baseline UAS7 for BHRA$^+$ and BHRA$^–$ groups in each arm<br>● Line plots of mean change in UAS7 from Baseline for each Week 1-12 for BHRA$^–$ and BHRA$^+$ patient groups within each fenebrutinib arm (200 mg BID, 150 mg QD or 50 mg QD) as well as the placebo arm<br>● Similar outputs to above for UAS7 components (itch, hives) will be used to aid interpretation<br>● The above outputs will be generated at the interim analysis and final analysis
Exploratory analyses	● Comparison of frequency of partial and complete responders by BHRA group and arm<br>● The Phase 2b fenebrutinib arms may be pooled to increase sample size (limited to all three arms, to avoid post-hoc selection bias)<br>● Others analyses, as needed to aid in interpretation
Statistical hypothesis testing	● We do not plan on using formal statistical hypothesis testing due to the small sample size<br>● Simple visual comparison and comparison to the MID will be used instead

**Serum IgE predictive biomarker hypothesis**	In fenebrutinib-treated patients, the mean reduction in UAS7 from Baseline to Weeks 4 and 8 will be similar in IgE-high patients to IgE-low patients. CSU patients who have high serum IgE at Baseline experience greater response to omalizumab than patients with low IgE.[36] Preliminary testing in the Phase 2a suggests that IgE-high and IgE-low groups had similar response to fenebrutinib.
Serum IgE	● Covance Central Labs will perform serum IgE measurements on Screening visit serum samples<br>● If a Screening visit serum sample is not available for serum IgE measurement, the Baseline visit (Day 1 pre-dose) sample may be used in its place<br>● Serum IgE ≥43 IU/ml will be considered high and <43 IU/ml will be considered low[36]
Efficacy	● Mean change in UAS7 from Baseline to Weeks 4 and 8 will be the primary measures of efficacy<br>● Differences between IgE-high and IgE-low groups greater than or equal to the UAS7 minimally important difference (MID: 9.5–10.5) will be considered clinically meaningful[55]
Patients	● This analysis will use the same patients and visits used in the UAS7 topline efficacy readout<br>● If Screening and Baseline (Day 1 pre-dose) visit serum samples are not available for serum IgE measurement for a given patient, they will be excluded from the analysis
Biostats outputs	● Tables of descriptive statistics of mean change in UAS7 from Baseline to Weeks 4 and 8 for IgE-high and IgE-low patient groups within each fenebrutinib arm (200 mg BID, 150 mg QD or 50 mg QD) as well as the placebo arm<br>● Number of IgE-high and IgE-low patients in each arm at Screening<br>● Baseline UAS7 for IgE-high and IgE-low groups in each arm<br>● Line plots of mean change in UAS7 from Baseline for each Week 1–12 for IgE-high and IgE-low patient groups within each fenebrutinib arm (200 mg BID, 150 mg QD or 50 mg QD) as well as the placebo arm<br>● Similar outputs to above for UAS7 components (itch, hives) will be used to aid interpretation<br>● The above outputs will be generated at the interim analysis and final analysis
Exploratory analyses	● Comparison of frequency of partial and complete responders by IgE-high and IgE-low group and arm<br>● The Phase 2b fenebrutinib arms may be pooled to increase sample size (limited to all three arms, to avoid post-hoc selection bias)<br>● Others analyses, as needed to aid in interpretation
Statistical hypothesis testing	● No formal statistical hypothesis testing will be performed due to small sample size<br>● Simple visual comparison and comparison to the MID will be used instead

**Exploratory biomarkers**		
Biomarker	Rationale	Prediction
IgG-anti-FcεRI and anti-IgE specific immunoassays	● BHRA is thought to measure the presence of IgG-anti-FcεRI and/or anti-IgE, but is not specific to either antibody<br>● Measuring IgG-anti-FcεRI and anti-IgE individually may subset patients with greater accuracy	● IgG-anti-FcεRI$^+$ and/or anti-IgE$^+$ patients will have greater response to fenebrutinib than negative patients
Blood basophils	● Lower numbers of circulating basophils have been associated with BHRA positivity in CSU<br>● Blood basophils could potentially serve as an alternative to identifying BHRA$^+$ patients	● Basophil-low patients will have greater response to fenebrutinib than basophil-high patients (where high is defined as ≥ median and low < median)
Eosinophil-associated biomarkers (e.g. blood eosinophils, IgG-anti-FcεRII and MBP)	● CSU patients may benefit from therapies targeting eosinophils (e.g. anti-IL5, anti-IL5RA and AK002 [anti-Siglec-8])<br>● High circulating eosinophil numbers may be predictive of response to omalizumab in CSU (unpublished)	● Patients with evidence of eosinophil activation or involvement will be less responsive to fenebrutinib than patients without eosinophil involvement

Abbreviations: CSU, chronic spontaneous urticaria; BHRA, basophil histamine release assay; MBP, major basic protein, MID, minimally important difference; UAS7, weekly Urticaria Activity Score

Original Protocol, including Statistical Considerations and Analysis Plan (Section 6)

PROTOCOL

**TITLE:**	**A PHASE IIa, MULTICENTER, RANDOMIZED, DOUBLE-BLIND, PLACEBO-CONTROLLED PILOT STUDY OF GDC-0853 IN PATIENTS WITH REFRACTORY CHRONIC SPONTANEOUS URTICARIA (CSU)**
**PROTOCOL NUMBER:**	GS39684
**VERSION NUMBER:**	1
**EUDRACT NUMBER:**	2016-004624-35
**TEST PRODUCT:**	GDC-0853 (RO7010939)
**MEDICAL MONITOR:**	[redacted]
**SPONSOR:**	Genentech, Inc.
**DATE FINAL:**	See electronic date stamp below

FINAL PROTOCOL APPROVAL

Approver's Name	Title	Date and Time (UTC)
[redacted]	Company Signatory	13-Dec-2016 20:20:24

CONFIDENTIAL

The information contained in this document, especially any unpublished data, is the property of Genentech, Inc. (or under its control) and therefore is provided to you in confidence as an investigator, potential investigator, or consultant, for review by you, your staff, and an applicable Ethics Committee or Institutional Review Board. It is understood that this information will not be disclosed to others without written authorization from Genentech except to the extent necessary to obtain informed consent from persons to whom the drug may be administered.

TABLE OF CONTENTS

PROTOCOL ACCEPTANCE FORM 8

PROTOCOL SYNOPSIS 9

1. BACKGROUND 19

1.1 Background on Chronic Spontaneous Urticaria 19

1.2 Background on Bruton's Tyrosine Kinase and GDC-0853 20

1.2.1 Bruton's Tyrosine Kinase 20

1.2.2 Nonclinical Experience with GDC-0853 21

1.2.3 Clinical Experience with GDC-0853 22

1.3 Study Rationale and Benefit-Risk Assessment 24

1.3.1 Infections 25

1.3.2 Bleeding 25

1.3.3 Cytopenias 25

1.3.4 Hepatotoxicity 25

1.3.5 Cardiovascular Effects 26

1.3.6 Malignancy 26

2. OBJECTIVES AND ENDPOINTS 26

3. STUDY DESIGN 28

3.1 Description of the Study 28

3.2 End of Study and Length of Study 30

3.3 Rationale for Study Design 30

3.3.1 Rationale for GDC-0853 Dose and Schedule 30

3.3.2 Rationale for Patient Population 31

3.3.3 Rationale for Control Group 32

[redacted]

3.3.5 Rationale for Pharmacokinetic Sample Collection Schedule 32

3.3.6 Rationale for Efficacy Endpoints 32

4. MATERIALS AND METHODS 33

4.1 Patients 33

4.1.1 Inclusion Criteria 33
4.1.2 Exclusion Criteria 35
4.2 Method of Treatment Assignment and Blinding 38
4.2.1 Randomization and Blinding 38
4.2.2 Unblinding 39
4.3 Study Treatment 39
4.3.1 Formulation, Packaging, and Handling of GDC-0853 and Placebo 39
4.3.2 Dosage, Administration, and Compliance of GDC-0853 and Placebo 40
4.3.2.1 GDC-0853 and Placebo Dose and Administration 40
4.3.2.2 GDC-0853 and Placebo Compliance 40
4.3.3 Investigational Medicinal Product Accountability 41
4.3.4 Post-Trial Access to GDC-0853 41
4.4 Concomitant Therapy and Additional Restrictions 41
4.4.1 Permitted Therapy 41
4.4.1.1 Dietary Supplements 42
4.4.1.2 Acid-Reducing Agents 42
4.4.2 Prohibited Therapy 43
4.4.2.1 Live or Attenuated Vaccinations 43
4.4.2.2 CYP3A Inhibition 43
4.4.3 Prohibited Food 45
4.4.4 Additional Restrictions 45
4.5 Study Assessments 45
4.5.1 Informed Consent Forms and Screening Log 45
4.5.2 Medical History and Demographic Data 45
4.5.3 Physical Examinations 45
4.5.4 Vital Signs 46
4.5.5 FricTest 46
4.5.6 Laboratory, Biomarker, and Other Biological Samples 46
4.5.7 Electrocardiograms 48
4.5.8 Patient-Reported Outcomes 49
4.5.8.1 Urticaria Patient Daily eDiary 49

4.5.8.2 Urticaria Activity Score 49
[redacted]
4.6 Patient, Treatment, Study, and Site Discontinuation 50
4.6.1 Patient Discontinuation 50
4.6.2 Study Treatment Discontinuation 50
4.6.3 Study and Site Discontinuation 51
5. ASSESSMENT OF SAFETY 51
5.1 Safety Plan 51
5.1.1 Safety Plan for Potential Risks Associated with GDC-0853 52
5.1.1.1 Infections 52
5.1.1.2 Vaccinations 53
5.1.1.3 Bleeding 54
5.1.1.4 Cytopenias 54
5.1.1.5 Gastrointestinal Effects 55
5.1.1.6 Hepatotoxicity 55
5.1.1.7 Cardiovascular Effects 56
5.1.1.8 Vascular Inflammation 57
5.1.1.9 Malignancy 57
5.1.2 Management of Patients Who Experience Specific Adverse Events 58
5.1.2.1 Management of Specific Adverse Events 58
5.1.2.2 Management of Increases in QT Interval 60
5.2 Safety Parameters and Definitions 60
5.2.1 Adverse Events 60
5.2.2 Serious Adverse Events (Immediately Reportable to the Sponsor) 61
5.2.3 Adverse Events of Special Interest (Immediately Reportable to the Sponsor) 62
5.3 Methods and Timing for Capturing and Assessing Safety Parameters 62
5.3.1 Adverse Event Reporting Period 62
5.3.2 Eliciting Adverse Event Information 63

5.3.3 Assessment of Severity of Adverse Events 63
5.3.4 Assessment of Causality of Adverse Events 63
5.3.5 Procedures for Recording Adverse Events............................ 64
5.3.5.1 Diagnosis versus Signs and Symptoms................................ 64
5.3.5.2 Adverse Events That Are Secondary to Other Events.. 64
5.3.5.3 Persistent or Recurrent Adverse Events................................ 65
5.3.5.4 Abnormal Laboratory Values 65
5.3.5.5 Abnormal Vital Sign Values 66
5.3.5.6 Abnormal Liver Function Tests 67
5.3.5.7 Deaths 67
5.3.5.8 Preexisting Medical Conditions... 68
5.3.5.9 Lack of Efficacy or Worsening of Chronic Spontaneous Urticaria 68
5.3.5.10 Hospitalization or Prolonged Hospitalization.......................... 68
5.3.5.11 Adverse Events Associated with an Overdose or Error in Drug Administration 69
5.3.5.12 Patient-Reported Outcome Data 69
5.4 Immediate Reporting Requirements from Investigator to Sponsor.. 69
5.4.1 Emergency Medical Contacts 70
5.4.2 Reporting Requirements for Serious Adverse Events and Adverse Events of Special Interest..................... 70
5.4.2.1 Events That Occur prior to Study Drug Initiation.................... 70
5.4.2.2 Events That Occur after Study Drug Initiation........................ 70
5.4.3 Reporting Requirements for Pregnancies.............................. 71
5.4.3.1 Pregnancies in Female Patients 71
5.4.3.2 Pregnancies in Female Partners of Male Patients 71
5.4.3.3 Congenital Anomalies/Birth Defects and Abortions 71
5.5 Follow-Up of Patients after Adverse Events 72
5.5.1 Investigator Follow-Up... 72
5.5.2 Sponsor Follow-Up 72
5.6 Adverse Events That Occur after the Adverse Event Reporting Period.. 72

5.7 Expedited Reporting to Health Authorities, Investigators, Institutional Review Boards, and Ethics Committees ... 72

6. STATISTICAL CONSIDERATIONS AND ANALYSIS PLAN ... 73
6.1 Determination of Sample Size ... 73
6.2 Summaries of Conduct of Study ... 74
6.3 Summaries of Treatment Group Comparability ... 74
6.4 Efficacy Analyses ... 74
6.4.1 Primary Efficacy Endpoint ... 74
6.4.2 Secondary Efficacy Endpoints ... 75
6.4.3 Exploratory Efficacy Endpoints ... 75
6.5 Safety Analyses ... 76
6.6 Pharmacokinetic Analyses ... 76
[redacted]

7. DATA COLLECTION AND MANAGEMENT ... 77
7.1 Data Quality Assurance ... 77
7.2 Electronic Case Report Forms ... 78
7.3 Electronic Patient-Reported Outcome Data ... 78
7.4 Source Data Documentation ... 78
7.5 Use of Computerized Systems ... 79
7.6 Retention of Records ... 79

8. ETHICAL CONSIDERATIONS ... 80
8.1 Compliance with Laws and Regulations ... 80
8.2 Informed Consent ... 80
8.3 Institutional Review Board or Ethics Committee ... 81
8.4 Confidentiality ... 81
8.5 Financial Disclosure ... 82

9. STUDY DOCUMENTATION, MONITORING, AND ADMINISTRATION ... 82
9.1 Study Documentation ... 82
9.2 Protocol Deviations ... 82
9.3 Site Inspections ... 82
9.4 Administrative Structure ... 83

9.5 Publication of Data and Protection of Trade Secrets 83
9.6 Protocol Amendments 84
10. REFERENCES 85

LIST OF TABLES

Table 1 Objectives and Corresponding Endpoints 27
Table 2 Twice Daily Patient Assessment of CSU Disease Activity (UAS Scale) 33
[redacted]
Table 4 Guidelines for Management of Patients Who Experience Specific Adverse Events 58
Table 5 Adverse Event Severity Grading Scale 63
Table 6 Causal Attribution Guidance 64

LIST OF FIGURES

Figure 1 Study Schema 30

LIST OF APPENDICES

Appendix 1 Schedule of Activities 89
Appendix 2 Childbearing Potential, Pregnancy Testing, and Contraception 93
Appendix 3 Urticaria Patient Daily eDiary 95
[redacted]

PROTOCOL ACCEPTANCE FORM

**TITLE:**	**A PHASE IIa, MULTICENTER, RANDOMIZED, DOUBLE-BLIND, PLACEBO-CONTROLLED PILOT STUDY OF GDC-0853 IN PATIENTS WITH REFRACTORY CHRONIC SPONTANEOUS URTICARIA (CSU)**
**PROTOCOL NUMBER:**	GS39684
**VERSION NUMBER:**	1
**EUDRACT NUMBER:**	2016-004624-35
**TEST PRODUCT:**	GDC-0853 (RO7010939)
**MEDICAL MONITOR:**	[redacted]
**SPONSOR:**	Genentech, Inc.

I agree to conduct the study in accordance with the current protocol.

____

Principal Investigator's Name (print)

____ ________________

Principal Investigator's Signature Date

Please return a copy of the signed form as instructed by the CRO. Please retain the original for your study files.

PROTOCOL SYNOPSIS

**TITLE:**	**A PHASE IIa, MULTICENTER, RANDOMIZED, DOUBLE-BLIND, PLACEBO-CONTROLLED PILOT STUDY OF GDC-0853 IN PATIENTS WITH REFRACTORY CHRONIC SPONTANEOUS URTICARIA (CSU)**
**PROTOCOL NUMBER:**	GS39684
**VERSION NUMBER:**	1
**EUDRACT NUMBER:**	2016-004624-35
**TEST PRODUCT:**	GDC-0853 (RO7010939)
**PHASE:**	Phase IIa
**INDICATION:**	Refractory Chronic Spontaneous Urticaria (CSU)
**SPONSOR:**	Genentech, Inc.

Objectives and Endpoints

This study will evaluate the efficacy, safety, and pharmacokinetics of GDC-0853 compared with placebo in patients with chronic spontaneous urticaria (CSU) refractory to anti-histamines (up to 4 times the approved dose per local treatment guidelines). Specific objectives and corresponding endpoints for the study are outlined below.

Objectives and Corresponding Endpoints

Objectives	Corresponding Endpoints
**Efficacy Objective:**	
• To evaluate the efficacy of GDC-0853 compared with placebo in patients with CSU who are refractory to anti-histamines	**Primary Endpoint:**<br>• Change from baseline in the UAS7 at Day 57 (Week 8)<br>**Secondary Endpoints:**<br>• Proportion of patients who are well controlled (UAS7 ≤ 6) at Day 57<br>• Change from baseline in the UAS7 at Day 29 (Week 4)<br>**Exploratory Endpoints:**<br>• Change from baseline in the weekly itch score at Day 29<br>• Change from baseline in the weekly itch score at Day 57<br>• Change from baseline in the weekly hives score at Day 57<br>• Proportion of patients who are well controlled (UAS7 ≤ 6) at Day 29<br>• Proportion of patients who achieve complete response (UAS7 = 0) at Day 29<br>• Proportion of patients who achieve complete response (UAS7 = 0) at Day 57<br>• Proportion of patients achieving MID in UAS7 at Day 57 (reduction from baseline ≥ 11 points)<br>• Proportion of patients achieving MID in the weekly itch score at Day 57 (reduction from baseline ≥ 5 points)

Objectives and Corresponding Endpoints (Continued)

Objectives	Corresponding Endpoints
	Exploratory Endpoints (Continued):<br>• Time to achieving MID in UAS7 (reduction from baseline ≥ 11 points)<br>• [redacted]
**Safety Objective:**	
• To evaluate the safety of GDC-0853 compared with placebo	• The nature, frequency, timing, and severity of adverse events<br>• Change from baseline in targeted vital signs, physical examination findings, ECGs, and clinical laboratory results following GDC-0853 administration
**Pharmacokinetic Objective:**	
• To characterize the pharmacokinetics of GDC-0853 in patients using a population PK approach	• Steady-state PK parameters ($AUC_{0\text{-}t}$, C_{max}, t_{max}, C_{trough}, $t_{1/2}$, apparent CL/F)
[redacted]	
[redacted]	[redacted]
[redacted]	[redacted]
[redacted]	[redacted]
[redacted]	
[redacted]	[redacted]

$AUC_{0\text{-}t}$ = area under the concentration–time curve from time 0 to time t; CL/F = apparent clearance; C_{max} = maximum concentration observed; C_{trough} = steady-state concentration at the end of a dosing interval; CSU = chronic spontaneous urticaria; MID = minimally important difference; [redacted]; PK = pharmacokinetic; $t_{1/2}$ = half-life; t_{max} = time to maximum concentration; UAS7 = Urticaria Activity Score over 7 days; [redacted]

Study Design

Description of Study

This pilot study is a multicenter, randomized, double-blind, placebo-controlled, parallel-group study of the efficacy and safety of GDC-0853 as add-on therapy for the treatment of adult

patients 18 – 75 years old who have been diagnosed with CSU and who remain symptomatic despite treatment with H1 antihistamines (including doses up to 4 times the approved dose per local treatment guidelines). The study will enroll approximately 45 patients across multiple sites. After screening, eligible patients will be randomly allocated in a 2:1 ratio to receive GDC-0853 or matching placebo for 8 weeks and will maintain stable doses of standard-of-care H1 antihistamine therapy throughout the study.

The study will consist of 3 distinct study periods over a time-period of 14 weeks as outlined below:

- Screening period: Day −14 to Day −1
- Treatment Period: Day 1 to Day 57 (Week 0 to Week 8)
- Follow-Up Period: Day 57 to Day 85 (Week 8 to Week 12)

Patients will have a screening period of approximately 2 weeks to establish their eligibility for the study and baseline symptom scores. For the duration of the screening period, patients must maintain stable doses of their pre-screening combination therapy with standard-of-care H1 antihistamines (i.e., up to 4 times the approved dose per local treatment guidelines). The screening period will consist of visits at Day −14 and Day −7. Patients must meet all of the following criteria to enter the screening period:

- Documented treatment with a regimen that includes standard-of-care H1 antihistamine for CSU at Day −14 and for at least the 3 consecutive days immediately prior to Day −14
- Willing and able to complete a symptom electronic diary (Urticaria Patient Daily eDiary) twice daily throughout the screening period to establish the patient's Urticaria Activity Score over 7 days (UAS7) score.

To be eligible for randomization, for the 7 days prior to randomization, patients must meet all of the following:

- Seven consecutive days of entries in the Urticaria Patient Daily eDiary, and
- UAS7 symptom score of ≥ 16 (range: 0 – 42)

Only in exceptional circumstances, when information concerning eligibility is outstanding (e.g., delayed laboratory results), will a longer screening period be permitted up to 3 business days. Upon approval from the Medical Monitor, patients may be re-screened or maybe retested during the screening period. Circumstances that may permit re-screening or re-testing include, but are not limited to, a laboratory test result that does not meet eligibility requirements.

On Day 1, eligible patients will be randomly allocated in a 2:1 ratio to receive GDC-0853 200 mg orally (PO) twice daily (BID) or placebo for 8 weeks. The primary efficacy endpoint will be at Day 57 (Week 8). Throughout the treatment period, patients must maintain stable doses of their pre-randomization H1 antihistamine therapy.

After completion of the 8-week treatment period, all patients will enter a 4-week safety follow-up period to allow for further characterization of the pharmacokinetics and pharmacodynamics of GDC-0853, and collection of additional efficacy and safety data. No study treatment will be given; patients must maintain stable doses of their pre-randomization CSU H1 antihistamine treatment. In the safety follow-up period, patients may add up to one additional H1 antihistamine therapy in case of worsened symptoms. The goal of allowing additional H1 antihistamine therapy after the treatment period is to reduce patient dropout for improved safety evaluation.

In addition to their daily background therapy, for the duration of the study all patients will be able to use a single approved dose of loratadine (10 mg maximum) or cetirizine (10 mg maximum) within a 24-hour period as rescue medication if symptoms worsen. Patients should record the use of this medication in their eDiary. Patients receiving proton-pump inhibitors (PPIs) or H2 receptor antagonists (H2RAs) should be stabilized on a regimen beginning at least 2 weeks prior to randomization and continuing throughout the study.

Number of Patients

Approximately 45 patients, aged 18 to 75 years old who have been diagnosed with refractory CSU and who remain symptomatic despite standard-of-care H1 antihistamine (i.e., up to 4 times the approved dose per local treatment guidelines), will be enrolled in this study.

Target Population

Inclusion Criteria

Patients must meet the following criteria for study entry:

- Willing to give written informed consent, adhere to the visit schedules, comply with the study drug regimen, and meet other study requirements
- Aged 18 – 75 years, inclusive
- Diagnosis of CSU refractory to H1 antihistamines at the time of randomization, as defined by all of the following:
  - The presence of itch and hives for > 6 consecutive weeks at any time prior to enrollment despite current use of H1 antihistamine, consistent with standard of care (i.e., up to 4 times the approved dose per local treatment guidelines) during this time period
  - UAS7 score ≥ 16 during the 7 days prior to randomization (Day 1)
  - Patients must have been on daily stable doses of H1 antihistamine, consistent with standard of care (i.e., up to 4 times the approved dose per local treatment guidelines) treatment for CSU starting at least 3 consecutive days immediately prior to the screening visit through Day 1and must document current use on all visits.
  - CSU diagnosis for ≥ 6 months
- Willing and able to complete an Urticaria Patient Daily eDiary for the duration of the study
- Completion of 7 days of the Urticaria Patient Daily eDiary entries in the 7 days prior to randomization
- No evidence of active or latent or inadequately treated infection with tuberculosis (TB) as defined by the following:
  - A negative QuantiFERON-TB-Gold® (QFT) performed at the screening visit or within the 3 months prior to screening -OR-
  - If QFT unavailable, a Mantoux purified protein derivative (PPD) skin test as defined by the Centers for Disease Control and Prevention guidelines, performed at the screening visit or within the 3 months prior to screening -AND-
  - Any additional procedures (e.g., chest X-Ray) required per local guidelines to rule out latent or active TB

  Patients with a history of Bacille Calmette-Guérin (BCG) vaccination should be screened using the QFT test, only.

    An indeterminate QFT test should be repeated.

    A positive QFT test or two successive indeterminate QFT results should be considered a positive diagnostic TB test.

    An indeterminate QFT test followed by a negative QFT test should be considered a negative diagnostic TB test.
- Only for patients currently receiving PPIs or H2RAs: Treatment must be at a stable dose during the 2 week screening period prior to randomization and with a plan to remain at a stable dose for the duration of the study
- For women of childbearing potential (including those who have had a tubal ligation): Agreement to remain abstinent (refrain from heterosexual intercourse) or use contraceptive methods that result in a failure rate of < 1% per year during the treatment period and for at least 4 weeks after the last dose of study drug

    A woman is considered to be of childbearing potential if she is postmenarcheal, has not reached a postmenopausal state (≥ 12 continuous months of amenorrhea with no identified cause other than menopause), and has not undergone surgical sterilization (removal of ovaries and/or uterus).

Examples of contraceptive methods with a failure rate of < 1% per year include bilateral tubal ligation, male sterilization, established proper use of hormonal contraceptives that inhibit ovulation, hormone-releasing intrauterine devices, and copper intrauterine devices. Women using estrogen-containing hormonal contraceptives as a method of contraception must also use a barrier, such as a male condom, in conjunction with the hormonal contraceptives.

The reliability of sexual abstinence should be evaluated in relation to the duration of the clinical trial and the preferred and usual lifestyle of the patient. Periodic abstinence (e.g., calendar, ovulation, symptothermal, or post-ovulation methods) and withdrawal are not acceptable methods of contraception.

- For men: agreement to remain abstinent (refrain from heterosexual intercourse) or use contraceptive measures and agreement to refrain from donating sperm, as defined below:

  With female partners of childbearing potential or pregnant female partners, men must remain abstinent or use a condom during the treatment period and for at least 4 weeks after the last dose of study treatment to avoid exposing the embryo. Men must refrain from donating sperm during this same period.

  The reliability of sexual abstinence should be evaluated in relation to the duration of the clinical trial and the preferred and usual lifestyle of the patient. Periodic abstinence (e.g., calendar, ovulation, symptothermal, or post-ovulation methods) and withdrawal are not acceptable methods of contraception.

Exclusion Criteria

Patients who meet any of the following criteria will be excluded from study entry:

- Treatment with omalizumab or other monoclonal antibody therapies used to treat CSU within 4 months prior to screening or primary nonresponse to omalizumab
- Use of a non-biologic investigational drug or participation in an investigational study with a non-biologic drug within 30 days prior to study drug administration on Day 1 (or within 5 half-lives of the investigational product, whichever is greater)
- Use of a biologic investigational therapy or participation in an investigational study involving biologic therapy within 90 days or 5 half-lives, whichever is greater, prior to study drug administration on Day 1
- Previous treatment with GDC-0853 or other Burton’s tyrosine kinase (BTK) inhibitors
- Patients whose urticaria is solely due to physical urticaria
- Other diseases with symptoms of urticaria or angioedema, including urticarial vasculitis, urticaria pigmentosa, erythema multiforme, mastocytosis, hereditary or acquired angioedema, lymphoma, or leukemia
- Atopic dermatitis, bullous pemphigoid, dermatitis herpetiformis, or other skin disease associated with itch such as psoriasis
- Routine (daily or every other day during 5 or more consecutive days) doses of the following medications within 30 days prior to screening: systemic or cutaneous (topical) corticosteroids (prescription or over the counter), hydroxychloroquine, methotrexate, cyclosporine, or cyclophosphamide
- Prior utilization of intravenous (IV) steroids for treatment of laryngeal angioedema
- IV immunoglobulin G (IVIG) or plasmapheresis within 30 days prior to screening
- History of anaphylactic shock without clearly identifiable avoidable antigen (e.g., due to food allergy)
- Hypersensitivity to GDC-0853 or any component of the formulation
- Major surgery, within 8 weeks prior to screening or surgery planned prior to end of study (12 weeks after randomization)
- Require any prohibited concomitant medications
- History of live attenuated vaccine within 6 weeks prior to randomization or requirement to receive these vaccinations at any time during study drug treatment

Seasonal influenza and H1N1 vaccination is permitted if the inactivated vaccine formulation is administered.

- Evidence of clinically significant cardiac, neurologic, psychiatric, pulmonary, renal, hepatic, endocrine (including uncontrolled diabetes mellitus), metabolic, or gastrointestinal (GI) disease that, in the investigator's opinion, would compromise the safety of the patient, interfere with the interpretation of the study results, or otherwise preclude patient participation.

  Any items that are cause for uncertainty must be reviewed with the Medical Monitor.
- Uncontrolled disease states, such as asthma, psoriasis, or inflammatory bowel disease, where flares are commonly treated with oral or parenteral corticosteroids
- History of vasculitis
- Current liver disease
- Any known active infection (with the exception of fungal nail infections or oral herpes)
- History of recurrent bacterial, viral, mycobacterial or fungal infections (defined as >2 similar episodes requiring anti-microbial treatment within the previous 12 months), with the exception of recurrent oral or genital herpes (herpes simplex virus 1/herpes simplex virus 2) or uncomplicated urinary tract infections in females.
- Any history of opportunistic infections that, in the investigator or Sponsor's judgment, would raise safety concerns regarding the patient's participation in the study
- Any major episode of infection requiring hospitalization or treatment with IV antimicrobials within 8 weeks prior to and during screening or treatment with oral antimicrobials within 2 weeks prior to and during screening

  Antimicrobials include antifungal, antibacterial, and antiviral agents.
- History of or currently active primary or secondary immunodeficiency, including known history of HIV infection
- Evidence of chronic and/or active hepatitis B or C

  Positive hepatitis B surface antigen (HBsAg) or hepatitis C serology (regardless of treatment status)

  Positive hepatitis B core antibody (HBcAb)
- History of cancer, including hematologic malignancy and solid tumors, within 10 years before screening

  Basal or squamous cell carcinoma of the skin that has been excised and is considered cured and in situ carcinoma of the cervix treated with apparent success by curative therapy >1 year prior to screening are not exclusionary.
- Women who are pregnant, nursing (breastfeeding), or intending to become pregnant during the study or within 4 weeks after completion of the study
- For women of childbearing potential (including those who have had a tubal ligation): positive serum pregnancy test result at screening or on Day 1.

  A serum pregnancy test is needed on Day 1 only if the urine pregnancy test is positive.
- History of alcohol, drug, or chemical abuse within the 12 months prior to screening as determined by the investigator
- Need for systemic anti-coagulation with warfarin, other oral or injectable anti-coagulants, or anti-platelet agents other than non-steroidal anti-inflammatory drugs (NSAIDs), aspirin, and other salicylates
- History of non-gallstone–related pancreatitis or chronic pancreatitis
- History of hospitalizations or transfusion for a GI bleed
- History of cerebrovascular accident (CVA) within 10 years or any history of hemorrhagic CVA
- History of spontaneous intracranial hemorrhage or history of traumatic intracranial hemorrhage within 10 years

- Known bleeding diathesis
- Screening 12-lead ECG that demonstrates clinically relevant abnormalities that may affect patient safety or interpretation of study results, including

  QT interval corrected using Fridericia's formula (QTcF) > 440 ms demonstrated by at least two ECGs > 30 minutes apart
- History of ventricular dysrhythmias or risk factors for ventricular dysrhythmias such as long QT syndrome and other genetic risk factors (e.g., Brugada syndrome), structural heart disease (e.g., severe left ventricular systolic dysfunction, severe left ventricular hypertrophy), coronary heart disease (symptomatic, or with ischemia demonstrated by diagnostic testing, prior coronary artery bypass grafting, or coronary lesions > 70% diameter stenosis that have not been or cannot be re-vascularized), clinically significant electrolyte abnormalities (e.g., hypokalemia, hypomagnesemia, hypocalcemia), or family history of sudden unexplained death or cardiac ion channel mutations (e.g., congenital long QT syndrome)
- Current treatment with medications that are well known to prolong the QT interval (see https://crediblemeds.org/index.php/login/dlcheck) at doses that have a clinically meaningful effect on QT, as determined by the investigator; the investigator may contact the Sponsor for confirmation if needed
- Any condition possibly affecting oral drug absorption (e.g., gastrectomy, clinically significant diabetic gastroenteropathy, or certain types of bariatric surgery such as gastric bypass)

  Procedures such as gastric banding, that simply divide the stomach into separate chambers, are not exclusionary.
- Any uncontrolled clinically significant laboratory abnormality that would affect safety, interpretation of study data, or the patient's participation in the study
- For German sites: Subjects who live in detention on court order or on regulatory action as per local and national law (see §40 subsection 1 sentence 3 no. 4 Arzneimittelgesetz).

The following exclusion criteria are based on screening laboratory tests. Laboratory tests may be repeated once during the screening period unless otherwise indicated:

- Creatinine > 1.5 times the upper limit of normal (ULN; may be repeated if 1.5–2 × ULN)
- ALT or AST > 1.5 times ULN (may be repeated if 1.5–3 × ULN)
- Total bilirubin > ULN (may be repeated if 1–3 × ULN)
- Hemoglobin < 9.5 g/dL (may be repeated if 9–9.4 g/dL)
- ANC < 1.5×10^9/L (may be repeated if $1.2–1.5 \times 10^9$/L)
- Platelet count < 100×10^9/L (may be repeated if $80–100 \times 10^9$/L)
- IgG < 500 mg/dL (should not be repeated)
- Abnormalities in hepatic synthetic function tests (e.g., PT, INR, PTT, albumin) judged by the investigator to be clinically significant

End of Study

The end of this study is defined as the date when all patients have completed the study completion visit or early termination visit or have otherwise been discontinued from the study.

Length of Study

The total duration of this study for each subject is approximately 14 weeks, including screening, treatment, and safety follow-up periods.

The total length of the study, from screening of the first patient to the end of the study, is expected to be approximately 10 months.

<u>Investigational Medicinal Products</u>

The investigational medicinal product for this study is GDC-0853. Patients will receive GDC-0853 200 mg PO BID or placebo for 8 weeks

Non-Investigational Medicinal Products

Throughout the treatment period, patients must maintain stable doses of their pre-randomization H1 antihistamine therapy. In addition to their daily background therapy, for the duration of the study all patients will be able to use a single approved dose of loratadine (10 mg maximum) or cetirizine (10 mg maximum) within a 24-hour period as rescue medication if symptoms worsen.

Statistical Methods

Primary Analysis

The primary efficacy endpoint is the change from baseline in the UAS7 at Day 57 (Week 8).

The Urticaria Activity Score (UAS) is to be recorded twice daily (i.e., morning and evening) using an eDiary that will be provided to each patient. Scores ranging from 0 (none) to 3 (severe) will be entered for each of the two UAS domains consisting of number of wheals (hives) and intensity of pruritis (itch) resulting in a total possible score of 0 to 6. The daily UAS is calculated as the average of the morning and evening scores. When either the morning or evening score is missing, the non-missing UAS for that day (morning or evening) will be used as the daily UAS, and when both the morning and evening UAS are missing, the daily UAS will be deemed missing. The UAS7 is the sum of the daily UAS over the 7 days prior to the time point of interest. The baseline UAS7 will be calculated as the sum of daily UAS values over the week (7 days) prior to Day 1.

When one or more daily UAS values is missing, over the week prior to a timepoint of interest, rules for deriving UAS7 will be as follows:

- If a patient has at least 4 completed daily scores on the UAS (both domains) over the 7 days prior to the time point of interest, the UAS7 will be defined as the average of the available daily scores, multiplied by 7.
- If a patient has fewer than 4 completed daily scores on the UAS over the 7 days prior to the time point of interest, then the UAS7 will be considered missing for that time point.

The primary endpoint will be analyzed using a mixed model for repeated measures model. Additional model covariates will include baseline UAS7 and its interaction with visit. Missing data will be handled by the model under the missing-at-random assumption without need for imputation. As a sensitivity analysis, an analysis of covariance (ANCOVA) model adjusted for country and baseline UAS7 will be fit, and missing Day 57 data will be imputed by last observation carried forward.

Determination of Sample Size

The purpose of this study is to evaluate the efficacy of GDC-0853 compared with placebo in improving the UAS7. Point and interval estimates of the change from baseline of the UAS7 within each treatment group as well as of the difference in change from baseline of the UAS7 between treatment groups will be presented.

The study will enroll approximately 45 patients. Patients will be randomized in a 2:1 ratio to receive treatment with either GDC-0853 or placebo. The sample size of approximately 30 patients in the GDC-0853 arm and 15 patients in the placebo arm provides approximately 80% power to detect an 11-point difference in the UAS7 change from baseline at Day 57 between treatment groups under the following assumptions:

- The absolute change from baseline at Day 57 is normally distributed with a standard deviation of 13.
- Two-sided alpha is 0.10.
- Drop out at Day 57 is 10%, leading to a 10% loss of information.

LIST OF ABBREVIATIONS AND DEFINITIONS OF TERMS

Abbreviation	Definition
ANCOVA	analysis of covariance
AUC	area under the concentration-time curve
AUC_{0-24}	area under the concentration-time curve from time 0 to 24 hours
AUC_{0-t}	area under the concentration-time curve from time 0 to time t
BCG	Bacille Calmette-Guérin
BCR	B-cell receptor
BID	twice a day
BTK	Bruton's tyrosine kinase
C_{max}	maximum observed concentration
C_{trough}	steady-state concentration at the end of a dosing interval
CIU	chronic idiopathic urticaria
CL/F	apparent clearance
CRP	C-reactive protein
CSR	clinical study report
CSU	chronic spontaneous urticaria
CTCAE	Common Terminology Criteria for Adverse Events
CVA	cerebrovascular accident
DLAE	dose-limiting adverse events
DLT	dose-limiting toxicity
EC	Ethics Committee
eCRF	electronic Case Report Form
EDC	electronic data capture
FcεRI	high affinity IgE receptor
FDA	Food and Drug Administration
GI	gastrointestinal
H2RA	H2 receptor antagonist
HBcAb	hepatitis B core antibody
HBsAg	hepatitis B surface antigen
IC_{90}	90% inhibitory concentration
ICH	International Conference on Harmonisation
Ig	immunoglobulin
IL-1	interleukin 1
IL-6	interleukin 6
IMP	investigational medicinal product
IND	Investigational New Drug (application)

Abbreviation	Definition
IRB	Institutional Review Board
IV	intravenous
IVIG	intravenous immunoglobulin G
IxRS	interactive voice or web-based response system
LTRA	leukotriene receptor antagonist
MAD	multiple-ascending dose
MID	minimally important difference
mITT	modified intent-to-treat
MMRM	mixed model for repeated measures
NCI	National Cancer Institute
NOAEL	no observed adverse effect level
NSAID	non-steroidal anti-inflammatory drug
PD	pharmacodynamic
PK	pharmacokinetic
PO	by mouth, orally
PPD	Purified Protein Derivative
PPI	proton-pump inhibitor
PRO	patient-reported outcome
QD	once a day
QFT	QuantiFERON-TB-Gold
QTcF	QT interval corrected using Fridericia's formula
RA	rheumatoid arthritis
SAD	single-ascending dose
SLE	systemic lupus erythematosus
$t_{1/2}$	half-life
t_{max}	time to maximum concentration
TB	tuberculosis
[redacted]	[redacted]
TNF-α	tumor necrosis factor alpha
UAS	Urticaria Activity Score
UAS7	Urticaria Activity Score over 7 days
[redacted]	[redacted]
XLA	X-linked agammaglobulinemia

1. BACKGROUND

1.1 BACKGROUND ON CHRONIC SPONTANEOUS URTICARIA

Chronic spontaneous urticaria (CSU, also referred to as chronic idiopathic urticaria [CIU]) is defined by the presence of wheals (hives), angioedema, or both for at least 6 weeks without an obvious cause (Greaves 2003). Previous estimates of the prevalence of CSU were approximately 0.1%, which persists in 20% of CSU patients 2 decades after diagnosis (Greaves 2000; Saini 2014). More recent evidence indicates that the point prevalence of the disease is approximately 1% (Maurer et al. 2011). Affected patients experience frequent pruritic hives with associated erythema and/or episodes of angioedema. CSU is reported to be associated with angioedema in approximately 50% of cases (McGirt et al. 2006). The classic urticarial description is a wheal and flare with a pale elevated lesion and surrounding erythema, ranging in size from a few millimeters to a few centimeters across, usually occurring in groups and often coalescing to form large confluent lesions.

The etiology of CSU is not clear. There are several theories including one proposing an infectious origin and another related to an autoimmune origin (Kaplan 2002). Some studies have found that approximately 30%–60% of patients with CSU have an autoimmune component as evidenced by the presence of a positive autologous serum skin test (Fiebiger et al. 1995; Tong et al. 1997; Zweiman et al. 1998). Another hypothesis regarding the etiology of CSU is that of a specific IgE antibody targeted to an endogenous antigen (Altrichter et al. 2011). Crosslinking of this IgE antibody docked in the high affinity IgE receptor (FcεRI) could result in the activation of skin mast cells and release of chemical mediators, such as histamine, that lead to the wheal and flare formation of a hive. In fact, recent findings in a study of more than 450 patients with CSU indicate that greater than 50% of CSU patients have IgE antibodies directed against thyroperoxidase (Altrichter et al. 2011). While an autoimmune etiology can be found in a large percentage of patients, many patients do not have an identified autoimmune etiology despite having a similar disease presentation (Ferrer 2015).

The final common pathway in CSU is the abnormal activation of mast cells and basophils in the skin. In patients with CSU, increased numbers of mast cells can be found in both affected and unaffected skin (Kay et al. 2014). Furthermore, mast cells from CSU patients are more sensitive, have lower thresholds for activation, and respond more robustly by releasing more histamine and other inflammatory mediators. Similarly, increased numbers of basophils have been seen in the lesional and non-lesional skin of patients with CSU (Ying et al. 2002). In patients with CSU, there is a paradoxical basopenia due to increased recruitment of basophils in diseased skin. The peripheral basopenia is inversely correlated with severity of disease activity. In comparison with healthy controls, studies have shown that blood basophils of CSU patients have a reduced capacity to release histamine following IgE stimulation. This paradoxical reduction is attributed to prior in vivo activation in the skin (Kern and Lichtenstein 1976).

However, when basophils from CSU patients are incubated with serum from other CSU patients or even normal sera, they release more histamine than basophils from healthy donors. Collectively, these data suggest that basophil signaling and activation are dysregulated in patients with CSU (Luquin et al. 2005).

Roughly half of patients with CSU achieve symptomatic control with H1 antihistamine therapy at approved doses. In some cases, the dose of antihistamine is increased (up to 4 times the approved dose per local treatment guidelines) and additional therapies, such as leukotriene receptor antagonists (LTRAs), are used although increased doses of antihistamines and LTRAs are not approved for the treatment of CSU. CSU can be a debilitating condition because of a lack of clinical response as well as the unpredictable course of the disease, both of which can have a profound negative influence on the patient's quality of life (Tilles 2005).

Patients may remain symptomatic despite ongoing H1 antihistamine treatment (up to 4 times the approved dose per local treatment guidelines; Powell et al. 2015), and for this group of patients, therapies such as immunosuppressants (including cyclosporine, corticosteroids, intravenous immunoglobulin G [IVIG], and methotrexate) and plasmapheresis have been used (Kozel and Sabroe 2004). These agents have variable success and may be associated with severe adverse effects. More recently, omalizumab was approved for treatment of refractory CSU/CIU.

1.2 BACKGROUND ON BRUTON'S TYROSINE KINASE AND GDC-0853

1.2.1 Bruton's Tyrosine Kinase

Discovery of the genetic basis for primary immunodeficiencies has been the source of new therapeutic targets in immunomodulatory therapies (Puri et al. 2013; Bugatti et al. 2014; Whang and Chang 2014). In humans, mutations in the gene for Bruton's tyrosine kinase (BTK), which is located on the X chromosome, can result in the development of an immunodeficiency state characterized by a significant absence of circulating B cells (Bruton 1952; Tsukada et al. 1993; Vetrie et al. 1993; Conley et al. 2005) and very low immunoglobulin levels due to a defect in B-cell differentiation at the pro– to pre–B cell stage that precludes assembly of the B-cell receptor (BCR) complex and immunoglobulin gene expression (Reth and Nielsen 2014). Affected male patients have a primary immune deficiency, X-linked agammaglobulinemia (XLA), and are susceptible to recurrent infections starting shortly after birth. Patients with XLA can live relatively normal lives on a standard therapy of intravenous (IV) immunoglobulin, which suggests that BTK can be safely inhibited, especially in people with established immune systems. IV immunoglobulin replacement therapy lowers the rate of infection, reduces hospitalization rates for patients with XLA, and has greatly improved the long-term prognosis of these patients.

BTK is essential for the differentiation and activity of B cells during immune system ontology and normal adaptive immune responses. BTK is activated by phosphatidylinositol 3-kinase–dependent plasma membrane recruitment and phosphorylation on tyrosine Y551 by the Src-family kinase Lyn. Autophosphorylation and activation also occurs on tyrosine Y223 in a BTK-specific manner. Once activated, BTK induces PLCγ2- and Ca^{2+}-dependent signaling, which leads to the activation of NF-κB– and NFAT-dependent pathways leading to cellular activation and differentiation (Niiro and Clark 2002). In addition, BTK is important in FcεRI signaling in both basophils and mast cells, the key cell types in the pathogenesis of CSU. BTK null mice have impaired FcεRI signaling resulting in decreased histamine and inflammatory cytokine release (Hata et al. 1998; Iyer et al. 2011).

1.2.2 Nonclinical Experience with GDC-0853

GDC-0853 is a highly selective, orally administered, reversible inhibitor of BTK that is being developed by Genentech, Inc. as a potential therapeutic for autoimmune diseases, including CSU. GDC-0853 has undergone extensive investigation in nonclinical in vitro and in vivo studies to characterize its pharmacological, metabolic, and toxicological properties (see the GDC-0853 Investigator's Brochure for further details).

In vitro cell-based experiments suggest that antagonism of BTK leads to inhibition of BCR-dependent B-cell proliferation and a reduction of inflammatory cytokine production from myeloid cells (including tumor necrosis factor alpha [TNF-α], interleukin 1 [IL-1], and interleukin 6 [IL-6]) by preventing signaling through the FcγRIII receptor (Di Paolo et al. 2011; Liu et al. 2011). GDC-0853 effectively blocks BCR- and CD40-mediated activation and proliferation of B cells. BTK in B cells also plays a role in TLR4-mediated B-cell proliferation and class switching. In monocytes, GDC-0853 inhibits TLR4- and immune complex-mediated inflammatory cytokine production, including TNF-α, which contributes to disease pathogenesis in rheumatoid arthritis (RA). In dendritic cells, BTK contributes to TLR8-mediated cytokine production (TNF-α and IL-6) (Sochorová et al. 2007). In basophils, BTK-dependent activation of the FcεRI leads to activation and upregulation of CD63.

As described above, the pathophysiology of CSU is not completely understood. A key step is FcεRI-activation and release of histamine and other inflammatory cytokines from mast cells and basophils, leading to the wheal and flare formation of a hive as well as angioedema. In support of the importance of BTK in FcεRI signaling and the pathogenesis of CSU, BTK null mice have impaired FcεRI signaling, resulting in decreased histamine and inflammatory cytokine release (Hata et al. 1998; Iyer et al. 2011).

Consistent with these findings, in vitro experiments with human mast cell lines demonstrated that GDC-0853 could effectively inhibit the release of histamine after activation of FcεRI by cross-linking IgE bound to FcεRI on the surface of mast cells. In

addition, in a Phase Ib study in healthy volunteers, oral administration of GDC-0853 inhibited ex vivo basophil activation as measured by diminished cell surface expression of CD63. As such, GDC-0853 inhibits the activity of two specific cell types that play key roles in disease pathology in CSU.

The GDC-0853 safety profile has been assessed in repeat-dose, general toxicology studies (once a day [QD] oral dosing) ranging from 1 week to 9 months in rats and dogs; in vitro and in vivo genetic toxicology studies; in vitro phototoxicity evaluation; in vitro and in vivo safety pharmacology studies of the central nervous, respiratory, and cardiovascular systems; and embryo-fetal development (Seg II) studies in rats and rabbits. Overall, GDC-0853 was well tolerated for up to 6 months in rats (up to 104 μM • hr) and up to 9 months in dogs (up to 36 μM • hr). Notable findings identified in nonclinical toxicology studies include vascular inflammation (≥ 56 μM • hr) in dogs, hepatotoxicity (180 μM • hr) in dogs and rats, and a minimal increase in corrected QT interval (QTc; 7 ms or 3%; extrapolated unbound maximum observed concentration [C_{max}] of 3.17 μM) in dogs. Fetal malformations in rats (at 627 μM • hr) and rabbits (≥ 10.6 μM • hr) warrant the continued use of highly effective contraception in clinical trials. On the basis of the nonclinical and clinical safety data to date, GDC-0853 is expected to be well tolerated at the doses and duration administered in the current study.

1.2.3 Clinical Experience with GDC-0853

As of August 2016, 195 subjects have received single or multiple doses of GDC-0853 in ongoing or completed studies (171 healthy subjects and 24 patients with hematological malignancies).

Study GO29089 is a Phase I, open-label study in which GDC-0853 has been evaluated in patients with relapsed or refractory B-cell non-Hodgkin's lymphoma or chronic lymphocytic leukemia. In order to focus on the autoimmune indications, Genentech elected to stop development of GDC-0853 in oncology, and the Phase I study is continuing without further patient enrollment. Enrollment was stopped after completion of the 400-mg dose level at which time 24 patients had been enrolled in 3 cohorts: 100 mg, 200 mg, and 400 mg GDC-0853 daily. Seven patients remain in the study, and all have undergone intrapatient dose escalation to 400 mg QD GDC-0853. The mean duration of daily dosing for these 7 patients has been 21 months (range of 18 – 23 months). GDC-0853 was well tolerated with no dose-limiting toxicities (DLTs), maximum tolerated dose was not reached, and adverse events have been generally non-serious National Cancer Institute Common Terminology Criteria for Adverse Events, Version 4.0 (NCI CTCAE v4.0) Grade 1 or Grade 2 events that have been clinically manageable. The adverse events regardless of causality reported in ≥ 15% of patients include fatigue, nausea, diarrhea, headache, abdominal pain, dizziness, cough, and thrombocytopenia. As of August 2016, 11 serious adverse events had been reported in 5 patients, of whom 2 had a fatal outcome (i.e., complications of H1N1 influenza and influenza pneumonia).

Study GP29318 was a two-part, single-ascending dose (SAD) study to assess the safety, tolerability, and pharmacokinetics of GDC-0853 administered to 93 healthy subjects. In Part 1, the single-dose–escalation portion, 71 subjects were randomized to panels of 8 subjects (6:2 active:placebo ratio) per dose group (0.5 – 600 mg), with 53 subjects receiving active GDC-0853. In Part 2, 100 mg GDC-0853 was administered to 40 subjects in the open-label food and pilot rabeprazole effect study. There were no serious adverse events and no withdrawals due to adverse events during the conduct of Study GP29318. In Part 1 of the study, there were no dose-limiting adverse events (DLAEs) at single doses up to 600 mg GDC-0853. All adverse events were mild in intensity (Grade 1; Toxicity Grading Scale for Healthy Adult and Adolescent Volunteers Enrolled in Preventive Vaccine Clinical Trials) and transient. No adverse events increased in intensity or frequency with dose escalation. There were two treatment-emergent adverse events of mild self-limited headache reported as related to GDC-0853 administration. There were no trends in safety laboratory findings, vital sign changes, physical examination findings, or ECG changes. There were no trends in hepatic laboratory changes following single doses of GDC-0853 in healthy subjects. Refer to the GDC-0853 Investigator's Brochure for further information on Study GP29318, including pharmacokinetics.

Study GA29347 was a multiple-ascending dose (MAD) study to assess the safety, tolerability, and pharmacokinetics of multiple doses of GDC-0853 administered to 30 healthy subjects for 14 days. Forty subjects were randomized to panels of 8 subjects (6:2 active:placebo) per dose group, at doses of 20 mg twice a day (BID), 60 mg BID, 150 mg BID, 250 mg BID, or 500 mg QD for 14 days, with 30 subjects receiving active GDC-0853. The study drug was well tolerated. There were no serious adverse events and no withdrawals due to adverse events during the conduct of the study. All adverse events were mild in intensity (Grade 1) and transient, with no relationship to dose. Adverse events included skin reactions (i.e., rash, contact dermatitis, and skin irritation from ECG leads), nausea, headache, insomnia, toothache, tinnitus, and asymptomatic bacteriuria. There were no trends in safety laboratory, vital sign, physical examination, or ECG findings.

Study GP29832 was a relative bioavailability study designed to evaluate the effects of formulation, food, and proton-pump inhibitor (PPI) or methotrexate co-administration on the pharmacokinetics of GDC-0853 in healthy subjects. GDC-0853 was well tolerated when administered to 48 healthy subjects at the 200-mg dose level.

Study GA29350 is a multicenter Phase II dose ranging study comparing the efficacy and safety of GDC-0853 versus placebo and adalimumab in patients with RA who have had an inadequate response to previous methotrexate therapy and versus placebo in patients with an inadequate response to previous tumor necrosis factor therapy. The study began enrollment in September 2016, and the total planned enrollment is approximately 580 patients.

Study GA30044 is the first study investigating GDC-0853 in systemic lupus erythematosus (SLE). This is a multicenter, Phase II, randomized, double-blind, placebo-controlled, parallel-group, dose-ranging study designed to evaluate the efficacy and safety of GDC-0853 in patients with moderate-to-severe active SLE in combination with standard-of-care therapy.

Refer to the GDC-0853 Investigator's Brochure for detailed background information on GDC-0853 as well as for additional details on nonclinical and clinical studies.

1.3 STUDY RATIONALE AND BENEFIT-RISK ASSESSMENT

Omalizumab, an anti-IgE monoclonal antibody, has demonstrated efficacy in treating patients with CSU, highlighting the key role of IgE in CSU pathogenesis. Inhibition of IgE-mediated FcεRI signaling by BTK inhibition offers a promising mechanism for the treatment of CSU. In preclinical in vitro studies, BTK inhibition was able to prevent the release of histamine and other inflammatory cytokines from mast cells after FcεRI engagement and cross-linking. In addition, in the healthy volunteer SAD and MAD studies, oral administration of GDC-0853 was able to inhibit ex vivo basophil activation as demonstrated by diminished CD63 cell surface expression. As such, GDC-0853 inhibits the two key pathogenic cell types in CSU. The aim of this study is to determine if GDC-0853 can effectively treat patients with CSU, as measured by reduction in disease activity.

Humans with a mutation in the XLA gene and who, therefore, lack functional BTK can live relatively normal lives on a standard therapy of IV immunoglobulin (Kaveri et al. 2011), suggesting that BTK can be safely inhibited in patients with CSU who have functional immune systems to explore this hypothesis. Clinical experience with GDC-0853 to date has not generated safety concerns that would preclude further evaluation in patients with CSU. GDC-0853 has been administered to 195 subjects to date (i.e., 171 healthy subjects and 24 patients with hematological malignancies) at doses from 0.5 to 600 mg and has been well tolerated with no safety signals. In the SAD (Study GP29318), MAD (Study GA29347), relative bioavailability (Study GP29832), and oncology (Study GO29089) studies, GDC-0853 was well tolerated with no DLAEs or DLTs. In the oncology study, there were 2 deaths due to complications of confirmed influenza (i.e., H1N1 influenza and influenza pneumonia).

Several measures will be taken to ensure the safety of patients participating in this study based on the potential risks for GDC-0853 based on nonclinical and clinical studies and published literature (see Section 5.1.1). Eligibility criteria have been designed to exclude patients at higher risk for potential toxicities. In addition, the sites selected for this study will be specialty dermatology and immunology clinics with experience in treating CSU.

1.3.1 Infections

GDC-0853 is a targeted immunomodulator; however, as a reversible inhibitor, the degree to which GDC-0853 antagonism of BTK signaling may suppress immune activity is unknown. Patients participating in this study may be at risk for infections, including opportunistic infections. Therefore, patients at high risk for infection will be excluded (see Section 5.1.1.1). Patients will be carefully monitored throughout the study for infections. GDC-0853 will be discontinued in any patient who develops a serious infection, opportunistic infection, or any infection requiring treatment with an IV antimicrobial agent.

1.3.2 Bleeding

BTK is expressed in platelets and is involved in platelet function via GPVI/collagen receptor signaling and GP1b receptor signaling. Platelets from patients with XLA demonstrate decreased activation in response to submaximal collagen stimulation but normal response to thrombin; clinically, there is no reported bleeding propensity in patients with XLA. In the GDC-0853 clinical study involving oncology patients (GO29089), 2 patients experienced Grade ≥3 gastrointestinal (GI) bleeding. These events were not dose related and occurred in patients on non-steroidal anti-inflammatory drugs (NSAIDs)/acetylsalicylic acid with a history of gastroesophageal or peptic ulcer disease.

It is unknown whether GDC-0853 will increase the risk of bleeding in patients with CSU who receive antiplatelet or anticoagulant therapies. Therefore, the eligibility criteria exclude patients at high risk for bleeding complications.

1.3.3 Cytopenias

Neutropenia, anemia, and thrombocytopenia have been observed in patients with hematologic malignancies who received GDC-0853. No clinically significant changes in cell counts were observed in the healthy volunteer studies. Events have been monitorable and clinically manageable. Cell counts will be monitored regularly throughout the study.

1.3.4 Hepatotoxicity

Evidence of hepatobiliary injury was observed in animals administered relatively high doses of GDC-0853 in repeat-dose toxicity studies. In clinical studies to date, including single dose and multiple dosing for 14 days in healthy subjects and QD dosing for over 1 year in patients with hematological malignancies, there have been no adverse events of liver enzyme elevations or trends toward elevations in laboratory evaluations. To minimize this risk, exclusion criteria have been defined for abnormal liver enzyme and function tests and current liver disease (see Section 4.1.2).

1.3.5 Cardiovascular Effects

GDC-0853 is considered to have a low potential to cause QT interval prolongation or to directly affect other cardiovascular parameters at therapeutic exposures. A minimal increase in corrected QT (QTc; 7 ms or 3%) interval was noted at 45 mg/kg in the single-dose cardiovascular safety pharmacology study in telemetry-instrumented dogs. Cardiac safety will be evaluated in all patients at baseline and throughout the study, with routine monitoring of vital signs, including heart rate and blood pressure, collection of ECGs, and reporting of cardiac adverse events.

1.3.6 Malignancy

The impact of BTK inhibition on the development of malignancies is not known; however, malignancies are considered a potential concern for all immunomodulatory agents. Patients with a history of cancer within 10 years of screening will be excluded from study participation, except for basal or squamous cell carcinoma of the skin that has been excised and is considered cured and in situ carcinoma of the cervix treated with apparent success by curative therapy more than 1 year prior to screening.

Overall, GDC-0853 has been well tolerated in Phase I healthy subjects and an oncology study. On the basis of the compelling mechanism for BTK inhibition in CSU, the benefit-risk ratio for this study is deemed acceptable. The safety profile of GDC-0853 will be further characterized in this Phase IIa study. A robust safety monitoring plan that describes the potential risks for GDC-0853 and the risk-mitigation strategies to minimize risks for the patients in this trial is provided in Section 5.

Please refer to the most recent GDC-0853 Investigator's Brochure for additional details on clinical and nonclinical studies and additional safety information.

2. OBJECTIVES AND ENDPOINTS

This study will evaluate the efficacy, safety, and pharmacokinetics of GDC-0853 compared with placebo in patients with CSU refractory to anti-histamines (up to 4 times the approved dose per local treatment guidelines). Specific objectives and corresponding endpoints for the study are outlined in Table 1.

Table 1 Objectives and Corresponding Endpoints

Objectives	Corresponding Endpoints
**Efficacy Objective:**	
• To evaluate the efficacy of GDC-0853 compared with placebo in patients with CSU who are refractory to anti-histamines	**Primary Endpoint:**<br>• Change from baseline in the UAS7 at Day 57 (Week 8)<br>**Secondary Endpoints:**<br>• Proportion of patients who are well controlled (UAS7 ≤ 6) at Day 57<br>• Change from baseline in the UAS7 at Day 29 (Week 4)<br>**Exploratory Endpoint:**<br>• Change from baseline in the weekly itch score at Day 29<br>• Change from baseline in the weekly itch score at Day 57<br>• Change from baseline in the weekly hives score at Day 57<br>• Proportion of patients who are well controlled (UAS7 ≤ 6) at Day 29<br>• Proportion of patients who achieve complete response (UAS7 $=0$) at Day 29<br>• Proportion of patients who achieve complete response (UAS7 $=0$) at Day 57<br>• Proportion of patients achieving MID in UAS7 at Day 57 (reduction from baseline ≥ 11 points)<br>• Proportion of patients achieving MID in the weekly itch score at Day 57 (reduction from baseline ≥ 5 points)<br>• Time to achieving MID in UAS7 (reduction from baseline ≥ 11 points)<br>• [redacted]
**Safety Objective:**	
• To evaluate the safety of GDC-0853 compared with placebo	• The nature, frequency, timing, and severity of adverse events<br>• Change from baseline in targeted vital signs, physical examination findings, ECGs, and clinical laboratory results following GDC-0853 administration
**Pharmacokinetic Objective:**	
• To characterize the pharmacokinetics of GDC-0853 in patients using a population PK approach	• Steady-state PK parameters (AUC_{0-t}, C_{max}, t_{max}, C_{trough}, $t_{1/2}$, apparent CL/F)

Table 1 Objectives and Corresponding Endpoints (cont.)

[redacted]	
[redacted]	[redacted]
[redacted]	[redacted]
[redacted]	[redacted]
[redacted]	
[redacted]	[redacted]
[redacted]	[redacted]

AUC_{0-t} = area under the concentration–time curve from time 0 to time t; CL/F = apparent clearance; C_{max} = maximum concentration observed; C_{trough} = steady-state concentration at the end of a dosing interval; CSU = chronic spontaneous urticaria; [redacted]; MID = minimally important difference; [redacted] PK = pharmacokinetic; $t_{1/2}$ = half-life; t_{max} = time to maximum concentration; UAS7 = Urticaria Activity Score over 7 days; [redacted]

3. STUDY DESIGN

3.1 DESCRIPTION OF THE STUDY

This pilot study is a multicenter, randomized, double-blind, placebo-controlled, parallel-group study of the efficacy and safety of GDC-0853 as add-on therapy for the treatment of adult patients 18 – 75 years old who have been diagnosed with CSU and who remain symptomatic despite treatment with H1 antihistamines (including doses up to 4 times the approved dose per local treatment guidelines). The study will enroll approximately 45 patients across multiple sites. After screening, eligible patients will be randomly allocated in a 2:1 ratio to receive GDC-0853 or matching placebo for 8 weeks and will maintain stable doses of standard-of-care H1 antihistamine therapy throughout the study.

The study will consist of 3 distinct study periods over a time-period of 14 weeks as outlined below (see Figure 1):

- Screening period: Day −14 to Day −1
- Treatment Period: Day 1 to Day 57 (Week 0 to Week 8)
- Follow-Up Period: Day 57 to Day 85 (Week 8 to Week 12)

Patients will have a screening period of approximately 2 weeks to establish their eligibility for the study and baseline symptom scores. For the duration of the screening period, patients must maintain stable doses of their pre-screening combination therapy with standard-of-care H1 antihistamines (i.e., up to 4 times the approved dose per local treatment guidelines). The screening period will consist of visits at Day −14 and Day −7. Patients must meet all of the following criteria to enter the screening period:

- Documented treatment with a regimen that includes standard-of-care H1 antihistamine for CSU at Day −14 and for at least the 3 consecutive days immediately prior to Day −14 (see Section 4.4.1 for list of H1 antihistamines available for use in this study)
- Willing and able to complete a symptom electronic diary (Urticaria Patient Daily eDiary) twice daily throughout the screening period to establish the patient's Urticaria Activity Score over 7 days (UAS7) score.

To be eligible for randomization, for the 7 days prior to randomization, patients must meet all of the following:

- Seven consecutive days of entries in the Urticaria Patient Daily eDiary, and
- UAS7 symptom score of $\geq$16 (range: 0−42)

Only in exceptional circumstances, when information concerning eligibility is outstanding (e.g., delayed laboratory results), will a longer screening period be permitted up to 3 business days. Upon approval from the Medical Monitor, patients may be re-screened or maybe retested during the screening period. Circumstances that may permit re-screening or re-testing include, but are not limited to, a laboratory test result that does not meet eligibility requirements.

On Day 1, eligible patients will be randomly allocated in a 2:1 ratio to receive GDC-0853 200 mg orally (PO) BID or placebo for 8 weeks. The primary efficacy endpoint will be at Day 57 (Week 8). Throughout the treatment period, patients must maintain stable doses of their pre-randomization H1 antihistamine therapy.

After completion of the 8-week treatment period, all patients will enter a 4-week safety follow-up period to allow for further characterization of the pharmacokinetics and pharmacodynamics of GDC-0853, and collection of additional efficacy and safety data. No study treatment will be given; patients must maintain stable doses of their pre-randomization CSU H1 antihistamine treatment. In the safety follow-up period,

patients may add up to one additional H1 antihistamine therapy in case of worsened symptoms (see Section 4.4.1). The goal of allowing additional H1 antihistamine therapy after the treatment period is to reduce patient dropout for improved safety evaluation.

In addition to their daily background therapy, for the duration of the study, all patients will be able to use a single approved dose of loratadine (10 mg maximum) or cetirizine (10 mg maximum) within a 24-hour period as rescue medication if symptoms worsen. Patients should record the use of this medication in their eDiary. Patients receiving PPIs or H2 receptor antagonists (H2RAs) should be stabilized on a regimen beginning at least 2 weeks prior to randomization and continuing throughout the study (see Section 4.4.1.2).

Figure 1 Study Schema

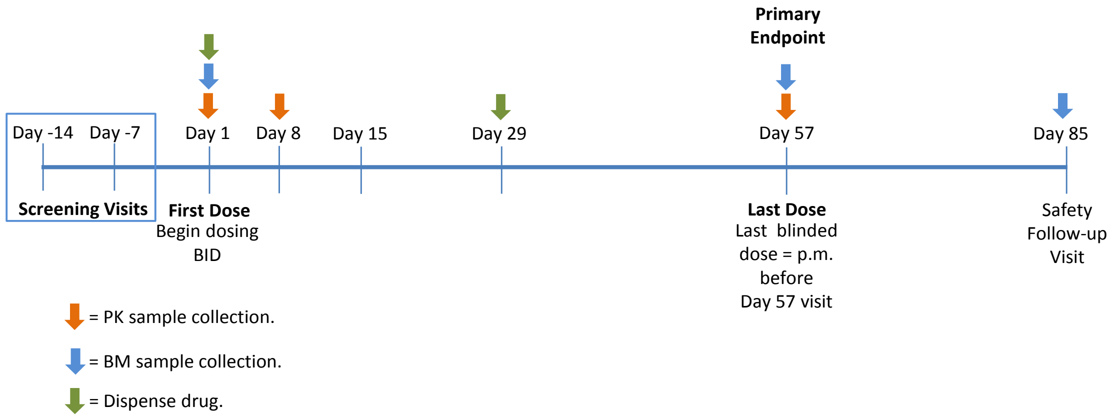

BM = biomarker; PK = pharmacokinetic; BID = twice a day.

3.2 END OF STUDY AND LENGTH OF STUDY

The end of this study is defined as the date when all patients have completed the study completion visit or early termination visit or have otherwise been discontinued from the study. The total duration of this study for each subject is approximately 14 weeks, including screening, treatment, and safety follow-up periods.

The total length of the study, from screening of the first patient to the end of the study, is expected to be approximately 10 months.

3.3 RATIONALE FOR STUDY DESIGN

3.3.1 Rationale for GDC-0853 Dose and Schedule

In the proposed study, patients randomly allocated to the GDC-0853 arm will receive 200 mg of GDC-0853 PO BID daily for 8 weeks. This dose is expected to be well tolerated and to substantially inhibit BTK inhibitor activity, based on results from the Phase I studies. Dose-dependent target inhibition was demonstrated in the Phase I SAD and MAD studies with use of different pharmacodynamic (PD) assays (e.g.,

phospho-BTK activity and CD63 in basophils). On the basis of the pharmacokinetic (PK) and PK/PD models constructed using data from the relative bioavailability, SAD, and MAD healthy volunteer studies and of the tolerability of dosing up to 250 mg BID, the 200 mg BID dose is expected to provide a steady-state exposure achieving 90% maximal inhibitory concentration (IC_{90}) over the entire dosing interval in greater than 75% of patients. It should be noted that the extent of target engagement required for clinical efficacy is unknown. However, as this is a pilot study, the dose tested will enable initial assessment of clinical efficacy in CSU.

A fumaric acid tablet formulation of GDC-0853 will be used in this study. In the relative bioavailability Study GP29832, the tablet formulation decreased PK variability and appeared to improve absorption by mitigating pH-dependent solubility compared with the powder-in-capsule formulation. The proposed dose in this study, 200 mg BID (fumaric acid tablet), is expected to achieve exposures (area under the concentration−time curve [AUC] and C_{max}) that are in the range of those observed following administration of 250 mg BID (powder-in-capsule) in Study GA29347 and 5-fold below the exposures at the no observed adverse effect level (NOAEL) of the nonclinical chronic toxicity studies (see the GDC-0853 Investigator's Brochure).

Study GP29832 also characterized the effect of food and the effect of the PPI rabeprazole on the pharmacokinetics of GDC-0853. A moderate-fat meal did not substantially change the systemic exposure following a single administration of 200 mg GDC-0853, since the fumaric acid tablet formulation (fed:fasted geometric mean ratio 90% CI) for area under the concentration−time curve from time 0 to 24 hours (AUC_{0-24}) and C_{max} were 1.08 (0.97−1.22) and 0.85 (0.72−1.01), respectively, supporting administration without regard to food. Co-administration of multiple doses of rabeprazole with a single dose of 200 mg GDC-0853 with the fumaric acid tablet formulation decreased GDC-0853 systemic exposure (AUC_{0-24}) by approximately 32% and 44% in the fasted and fed states, respectively, compared with tablet alone. In order to evaluate PK in a relevant patient population, stable use of a PPI regimen will be permitted during the study.

3.3.2 Rationale for Patient Population

Patients enrolled in this study will have a CSU diagnosis for $\geq$6 months and will be refractory to treatment with a combination of H1 antihistamines consistent with standard of care (i.e., up to 4 times the approved dose per local treatment guidelines) as demonstrated by the presence of itch and hives for $>$6 consecutive weeks on this treatment at any time prior to enrollment. In addition, patients will have a UAS7 score of $\geq$16 during the 7 days prior to randomization despite current use of combination therapy.

While H1 antihistamines are the mainstay of therapy for CSU, some patients do not respond or respond only partially to these therapies, and these patients tend to

experience more severe disease. This patient population was selected for this study because of the unmet medical need for more effective oral treatments.

3.3.3 Rationale for Control Group

A placebo-treated control group is required for this study in order to achieve its efficacy and safety objectives given the inherent variability in symptoms and the different rates of improvement in the placebo arms of prior studies. Patients in the placebo arm will continue to receive stable standard-of-care anti-H1 therapy throughout the study. In addition, the study will allow for rescue medications for persistent symptoms.

[redacted]

3.3.5 Rationale for Pharmacokinetic Sample Collection Schedule

The sampling schedule is designed to assess multiple pre-dose (prior to study drug administration in clinic) plasma GDC-0853 concentrations, which will enable the estimation of systemic GDC-0853 exposures and subsequent exposure-response analyses. Results will be used to inform dosing regimens for future studies of GDC-0853.

3.3.6 Rationale for Efficacy Endpoints

The change in the UAS7 (see Table 2 for daily assessment of Urticaria Activity Score [UAS]) has been chosen as the primary efficacy endpoint as it has been used in pivotal trials in CSU to measure reduction in CSU disease severity. The UAS7 is a summation of the average daily (a.m./p.m.) scores on the UAS (range: 0 – 6), which is a composite diary score with numeric severity intensity ratings on a scale of 0–3 (0 = none to 3 = intense/severe) for two domains: the intensity of the itch and the number of wheals/hives (see Table 2). The UAS will be recorded by the patient twice daily (morning and evening) in the patient Urticaria Patient Daily eDiary. UAS7 scores range from 0 – 42 and the minimally important difference (MID) is considered to be a reduction from baseline of ≥ 9.5 to 10.5 points (Mathias et al. 2012). The baseline UAS7 is the sum of the daily scores on the UAS over the 7 days prior to randomization (Day 1 visit), and the UAS7 at Day 57 is the sum of daily scores on the UAS over the 7 days prior to the Day 57 visit. The same principles of calculating baseline and Day 57 weekly scores will be applied to the other weekly outcomes unless otherwise stated.

The kinetics of response to GDC-0853 will also be carefully evaluated throughout the course of the study at regular intervals (at least every 1 – 2 weeks for a period of 8 weeks). In addition, disease recurrence or duration of treatment benefit after study drug is withdrawn during the safety follow-up period will be measured during this study. This will provide initial guidance for the duration of therapy in future studies.

Table 2 Twice Daily Patient Assessment of CSU Disease Activity (UAS Scale)

Score	Wheals (Hives)	Pruritus (Itch)
0	None	None
1	Mild (1–6 hives/12 hour)	Mild
2	Moderate (7–12 hives/12 hour)	Moderate
3	Intense (>12 hives/12 hour)	Severe

CSU = chronic spontaneous urticaria; UAS = Urticaria Activity Score.

4. MATERIALS AND METHODS

4.1 PATIENTS

Approximately 45 patients, aged 18 to 75 years old who have been diagnosed with refractory CSU and who remain symptomatic despite standard-of-care H1 antihistamine therapy (i.e., up to 4 times the approved dose per local treatment guidelines), will be enrolled in this study.

4.1.1 Inclusion Criteria

Patients must meet the following criteria for study entry:

- Willing to give written informed consent, adhere to the visit schedules, comply with the study drug regimen, and meet other study requirements
- Aged 18–75 years, inclusive
- Diagnosis of CSU refractory to H1 antihistamines at the time of randomization, as defined by all of the following:
  - The presence of itch and hives for >6 consecutive weeks at any time prior to enrollment despite current use of H1 antihistamines, consistent with standard of care (i.e., up to 4 times the approved dose per local treatment guidelines) during this time period
  - UAS7 score ≥16 during the 7 days prior to randomization (Day 1)
  - Patients must have been on daily stable doses of H1 antihistamines, consistent with standard-of-care therapy (i.e., up to 4 times the approved dose per local treatment guidelines) for CSU starting at least 3 consecutive days immediately prior to the screening visit through Day 1 and must document current use on all visits.
  - CSU diagnosis for ≥6 months
- Willing and able to complete an Urticaria Patient Daily eDiary for the duration of the study
- Completion of 7 days of the Urticaria Patient Daily eDiary entries in the 7 days prior to randomization

- No evidence of active or latent or inadequately treated infection with tuberculosis (TB) as defined by the following:
  - A negative QuantiFERON-TB-Gold® (QFT) performed at the screening visit or within the 3 months prior to screening -OR-
  - If QFT unavailable, a Mantoux purified protein derivative (PPD) skin test as defined by the Centers for Disease Control and Prevention guidelines, performed at the screening visit or within the 3 months prior to screening -AND-
  - Any additional procedures (e.g., chest X-Ray) required per local guidelines to rule out latent or active TB

  Patients with a history of Bacille Calmette-Guérin (BCG) vaccination should be screened using the QFT test, only.
  - An indeterminate QFT test should be repeated.
  - A positive QFT test or two successive indeterminate QFT results should be considered a positive diagnostic TB test.
  - An indeterminate QFT test followed by a negative QFT test should be considered a negative diagnostic TB test.
- Only for patients currently receiving PPIs or H2RAs: Treatment must be at a stable dose during the 2-week screening period prior to randomization and with a plan to remain at a stable dose for the duration of the study.
- For women of childbearing potential (including those who have had a tubal ligation): Agreement to remain abstinent (refrain from heterosexual intercourse) or use contraceptive methods that result in a failure rate of $<1\%$ per year during the treatment period and for at least 4 weeks after the last dose of study drug (see Section 5.4.3.1).

    A woman is considered to be of childbearing potential if she is postmenarcheal, has not reached a postmenopausal state (≥ 12 continuous months of amenorrhea with no identified cause other than menopause), and has not undergone surgical sterilization (removal of ovaries and/or uterus).

    Examples of contraceptive methods with a failure rate of $<1\%$ per year include bilateral tubal ligation, male sterilization, established proper use of hormonal contraceptives that inhibit ovulation, hormone-releasing intrauterine devices, and copper intrauterine devices. Women using estrogen-containing hormonal contraceptives as a method of contraception must also use a barrier, such as a male condom, in conjunction with the hormonal contraceptives.

    The reliability of sexual abstinence should be evaluated in relation to the duration of the clinical trial and the preferred and usual lifestyle of the patient. Periodic abstinence (e.g., calendar, ovulation, symptothermal, or post-ovulation methods) and withdrawal are not acceptable methods of contraception.

- For men: agreement to remain abstinent (refrain from heterosexual intercourse) or use contraceptive measures and agreement to refrain from donating sperm, as defined below (also see Section 5.4.3.2):

  With female partners of childbearing potential or pregnant female partners, men must remain abstinent or use a condom during the treatment period and for at least 4 weeks after the last dose of study treatment to avoid exposing the embryo. Men must refrain from donating sperm during this same period.

  The reliability of sexual abstinence should be evaluated in relation to the duration of the clinical trial and the preferred and usual lifestyle of the patient. Periodic abstinence (e.g., calendar, ovulation, symptothermal, or post-ovulation methods) and withdrawal are not acceptable methods of contraception.

4.1.2 Exclusion Criteria

Patients who meet any of the following criteria will be excluded from study entry:

- Treatment with omalizumab or other monoclonal antibody therapies used to treat CSU within 4 months prior to screening or primary nonresponse to omalizumab
- Use of a non-biologic investigational drug or participation in an investigational study with a non-biologic drug within 30 days prior to study drug administration on Day 1 (or within 5 half-lives of the investigational product, whichever is greater)
- Use of a biologic investigational therapy or participation in an investigational study involving biologic therapy within 90 days or 5 half-lives, whichever is greater, prior to study drug administration on Day 1
- Previous treatment with GDC-0853 or other BTK inhibitors
- Patients whose urticaria is solely due to physical urticaria
- Other diseases with symptoms of urticaria or angioedema, including urticarial vasculitis, urticaria pigmentosa, erythema multiforme, mastocytosis, hereditary or acquired angioedema, lymphoma, or leukemia
- Atopic dermatitis, bullous pemphigoid, dermatitis herpetiformis, or other skin disease associated with itch such as psoriasis
- Routine (daily or every other day during 5 or more consecutive days) doses of the following medications within 30 days prior to screening: systemic or cutaneous (topical) corticosteroids (prescription or over the counter), hydroxychloroquine, methotrexate, cyclosporine, or cyclophosphamide
- Prior utilization of IV steroids for treatment of laryngeal angioedema
- IVIG or plasmapheresis within 30 days prior to screening
- History of anaphylactic shock without clearly identifiable avoidable antigen (e.g., due to food allergy)
- Hypersensitivity to GDC-0853 or any component of the formulation
- Major surgery within 8 weeks prior to screening or surgery planned prior to end of study (12 weeks after randomization)
- Require any prohibited concomitant medications (see Section 4.4.2)
- History of live attenuated vaccine within 6 weeks prior to randomization or requirement to receive these vaccinations at any time during study drug treatment

Seasonal influenza and H1N1 vaccination is permitted if the inactivated vaccine formulation is administered.

- Evidence of clinically significant cardiac, neurologic, psychiatric, pulmonary, renal, hepatic, endocrine (including uncontrolled diabetes mellitus), metabolic, or GI disease that, in the investigator's opinion, would compromise the safety of the patient, interfere with the interpretation of the study results or otherwise preclude patient participation

  Any items that are cause for uncertainty must be reviewed with the Medical Monitor.

- Uncontrolled disease states, such as asthma, psoriasis, or inflammatory bowel disease, where flares are commonly treated with oral or parenteral corticosteroids
- History of vasculitis
- Current liver disease
- Any known active infection (with the exception of fungal nail infections or oral herpes)
- History of recurrent bacterial, viral, mycobacterial or fungal infections (defined as >2 similar episodes requiring anti-microbial treatment within the previous 12 months), with the exception of recurrent oral or genital herpes (herpes simplex virus 1/herpes simplex virus 2) or uncomplicated urinary tract infections in females.
- Any history of opportunistic infections that, in the investigator or Sponsor's judgment, would raise safety concerns regarding the patient's participation in the study
- Any major episode of infection requiring hospitalization or treatment with IV antimicrobials within 8 weeks prior to and during screening or treatment with oral antimicrobials within 2 weeks prior to and during screening

  Antimicrobials include antifungal, antibacterial, and antiviral agents.

- History of or currently active primary or secondary immunodeficiency, including known history of HIV infection
- Evidence of chronic and/or active hepatitis B or C
  - Positive hepatitis B surface antigen (HBsAg) or hepatitis C serology (regardless of treatment status)
  - Positive hepatitis B core antibody (HBcAb)
- History of cancer, including hematologic malignancy and solid tumors, within 10 years before screening

  Basal or squamous cell carcinoma of the skin that has been excised and is considered cured and in situ carcinoma of the cervix treated with apparent success by curative therapy >1 year prior to screening are not exclusionary.

- Women who are pregnant, nursing (breastfeeding), or intending to become pregnant during the study or within 4 weeks after completion of the study

- For women of childbearing potential (including those who have had a tubal ligation): positive serum pregnancy test result at screening or on Day 1.

  A serum pregnancy test is needed on Day 1 only if the urine pregnancy test is positive (see Section 4.1.1 for definition of “childbearing potential”).

- History of alcohol, drug, or chemical abuse within the 12 months prior to screening as determined by the investigator
- Need for systemic anti-coagulation with warfarin, other oral or injectable anti-coagulants, or anti-platelet agents other than NSAIDs, aspirin, and other salicylates
- History of non-gallstone–related pancreatitis or chronic pancreatitis
- History of hospitalizations or transfusion for a GI bleed
- History of cerebrovascular accident (CVA) within 10 years or any history of hemorrhagic CVA
- History of spontaneous intracranial hemorrhage or history of traumatic intracranial hemorrhage within 10 years
- Known bleeding diathesis
- Screening 12-lead ECG that demonstrates clinically relevant abnormalities that may affect patient safety or interpretation of study results, including
  - QT interval corrected using Fridericia’s formula (QTcF) > 440 ms demonstrated by at least two ECGs > 30 minutes apart
- History of ventricular dysrhythmias or risk factors for ventricular dysrhythmias such as long QT syndrome and other genetic risk factors (e.g., Brugada syndrome), structural heart disease (e.g., severe left ventricular systolic dysfunction, severe left ventricular hypertrophy), coronary heart disease (symptomatic or with ischemia demonstrated by diagnostic testing, prior coronary artery bypass grafting, or coronary lesions > 70% diameter stenosis that have not been or cannot be re-vascularized), clinically significant electrolyte abnormalities (e.g., hypokalemia, hypomagnesemia, hypocalcemia), or family history of sudden unexplained death or cardiac ion channel mutations (e.g., congenital long QT syndrome)
- Current treatment with medications that are well known to prolong the QT interval (see https://crediblemeds.org/index.php/login/dlcheck) at doses that have a clinically meaningful effect on QT, as determined by the investigator; the investigator may contact the Sponsor for confirmation if needed
- Any condition possibly affecting oral drug absorption (e.g., gastrectomy, clinically significant diabetic gastroenteropathy, or certain types of bariatric surgery such as gastric bypass)

  Procedures such as gastric banding, that simply divide the stomach into separate chambers, are not exclusionary.

- Any uncontrolled clinically significant laboratory abnormality that would affect safety, interpretation of study data, or the patient’s participation in the study

- For German sites: Subjects who live in detention on court order or on regulatory action as per local and national law (see §40 subsection 1 sentence 3 no. 4 Arzneimittelgesetz; Medicinal Products Act).

The following exclusion criteria are based on screening laboratory tests. Laboratory tests may be repeated once during the screening period unless otherwise indicated (see Section 5.3.5.4):

- Creatinine $>$1.5 times the upper limit of normal (ULN; may be repeated if 1.5–2 $\times$ ULN)
- ALT or AST $>$1.5 times ULN (may be repeated if 1.5–3 $\times$ ULN)
- Total bilirubin $>$ULN (may be repeated if 1–3 $\times$ ULN)
- Hemoglobin $<$9.5 g/dL (may be repeated if 9–9.4 g/dL)
- ANC $<1.5 \times 10^9$/L (may be repeated if 1.2–1.5 $\times 10^9$/L)
- Platelet count $<100 \times 10^9$/L (may be repeated if 80–100 $\times 10^9$/L)
- IgG $<$500 mg/dL (should not be repeated)
- Abnormalities in hepatic synthetic function tests (e.g., PT, INR, PTT, albumin) judged by the investigator to be clinically significant

4.2 METHOD OF TREATMENT ASSIGNMENT AND BLINDING

4.2.1 Randomization and Blinding

This study is randomized, double-blinded, and placebo-controlled to minimize potential bias in treatment assignment, patient monitoring, and efficacy assessments. Patients will be randomly allocated to 200 mg PO BID GDC-0853 or placebo at an approximately 2:1 ratio via an interactive voice or web-based response system (IxRS), using a stratified permuted blocks randomization scheme with stratification by country.

Patients and study site personnel will be blinded to the individual treatment assignments throughout the study. Only standard and safety laboratory data results from the local laboratory (such as CBC, chemistries, and pregnancy testing) will be available to sites. Results of other assessments performed after randomization that might unblind investigators to the treatment patients received will not be provided to sites or to the Sponsor's staff directly involved in study conduct. [redacted]

Although PK samples must be collected from patients assigned to the comparator arm to maintain the blinding of treatment assignment, PK assay results for these patients are generally not needed for the safe conduct or proper interpretation of this trial. Sponsor personnel or a designee responsible for performing PK assays will be unblinded to patients' treatment assignments to identify appropriate PK samples to be analyzed.

Samples from patients assigned to the placebo arm will not be analyzed except by request (e.g., to evaluate a possible error in dosing).

Patient and study site personnel will be blinded to treatment assignments throughout the study. During trial conduct, the Sponsor will monitor blinded clinical and safety data on safety and study conduct on an ongoing basis. If required for safety evaluations, Sponsor team personnel, but not the sites, not directly involved in the conduct of the study will have access to unblinded data. These Sponsor team personnel may include individuals with clinical and medical experience, biostatisticians, and individuals responsible for analyzing and interpreting the pharmacodynamics and pharmacokinetics of the study drug.

4.2.2 Unblinding

If unblinding is necessary for patient management (e.g., in the case of a serious adverse event for which patient management might be affected by knowledge of treatment assignment), the investigator will be able to break the treatment code by contacting the IxRS. Treatment codes should not be broken except in emergency situations. If the investigator wishes to know the identity of the study drug for any other reason, he or she should contact the Medical Monitor directly. The investigator should document and provide an explanation for any premature unblinding (e.g., accidental unblinding, unblinding due to a serious adverse event).

For regulatory reporting purposes, and if required by local health authorities, the Sponsor will break the treatment code for all serious, unexpected, suspected adverse reactions (see Section 5.7) that are considered by the investigator or Sponsor to be related to study drug.

4.3 STUDY TREATMENT

The investigational medicinal product (IMP) for this study is GDC-0853.

4.3.1 Formulation, Packaging, and Handling of GDC-0853 and Placebo

GDC-0853 will be provided by the Sponsor as 50-mg dose strength tablets with corresponding matching placebo tablets, which will be indistinguishable in appearance. Study drug (GDC-0853 or placebo) will be dispensed at the Day 1 and 29 visits.

Tablets will be supplied in bottles for the treatment arm to which the patient is randomly allocated. Each bottle will be labeled per local regulatory requirements. GDC-0853 and placebo tablets should be stored between 2°C and 8°C. Please refer to the pharmacy manual for detailed instructions on study drug storage and preparation.

For information on the formulation and handling of GDC-0853, see the GDC-0853 Investigator's Brochure.

4.3.2 Dosage, Administration, and Compliance of GDC-0853 and Placebo

4.3.2.1 GDC-0853 and Placebo Dose and Administration

The GDC-0853 dose level is 200 mg (4 tablets) BID (total of 8 tablets each day) with matching placebo (see Section 3.1). Patients will take GDC-0853/placebo BID, approximately every 12 hours starting on Day 1 and ending on Day 56. Although Day 57 is the last day of the study treatment period, no study drug will be given on Day 57. For mandatory morning clinic visits (i.e., Days 1 and 29), patients should be instructed that the morning dose of study drug will be taken in the clinic. On other clinic visit days, if the visit occurs in the morning, the patient should be instructed that the morning dose of study drug will be taken in the clinic.

GDC-0853 or placebo may be taken orally with or without food, except on Days 1 and 29 (see Appendix 1), when the morning dose of oral study drug will be administered at the morning (mandatory) clinic visit while fasting. The dates and times of the most recent prior meal, last dose of oral study drug (prior to clinic visit), and timing of oral study drug administration in clinic should be recorded at each clinic visit. Patients should be instructed that a missed dose should not be taken with the next scheduled dose.

In addition, any antacids (e.g., Maalox®, Pepto-Bismol®, Rolaids®) should be recorded as concomitant medications, including date and time of last administration. Administration of study drug should be staggered with antacid use (i.e., study drug should be taken 2 hours before or 2 hours after the antacid).

At the Day 1 and 29 visits, sufficient study medication tablets will be dispensed to complete dosing until the end of the study. When study medication is administered at the site, it will be administered under supervision of study personnel, and the amount of study medication dispensed must be recorded.

4.3.2.2 GDC-0853 and Placebo Compliance

The following measures will be taken to assess patient compliance with study drug. Patients will be directed to bring the study drug bottle to each visit after randomization. In addition, sites will be responsible for prepopulating the dates on the dosing label (affixed to the bottle) for when patients are scheduled to take study drug. The patients will record the times (a.m. or p.m.) that they take each dose in their eDiary. The number of tablets issued minus the number of tablets returned will be used to calculate the number of tablets taken and compliance.

Compliance will be documented on the source record. Any overdose or incorrect administration of study drug should be noted on the Study Drug Administration electronic Case Report Form (eCRF). Adverse events associated with an overdose or incorrect administration of study drug should be recorded on the Adverse Event eCRF. If

compliance is ≤ 80%, the investigator or designee is to counsel the patient and ensure steps are taken to improve compliance.

4.3.3 Investigational Medicinal Product Accountability

All IMPs required for completion of this study (GDC-0853 and placebo) will be provided by the Sponsor where required by local health authority regulations. The study site will acknowledge receipt of IMPs using the IxRS to confirm the shipment condition and content. Any damaged shipments will be replaced.

IMPs either will be disposed of at the study site according to the study site's institutional standard operating procedure or will be returned to the Sponsor with the appropriate documentation. The site's method of IMP destruction must be agreed to by the Sponsor. The site must obtain written authorization from the Sponsor before any IMP is destroyed, and IMP destruction must be documented on the appropriate form.

Accurate records of all IMPs received at, dispensed from, returned to, and disposed of by the study site should be recorded on the Drug Inventory Log.

4.3.4 Post-Trial Access to GDC-0853

Currently, the Sponsor (Genentech, a member of the Roche Group) does not have any plans to provide GDC-0853 or any other study treatments or interventions to patients who have completed the study. Patients in this study will be permitted to participate in future studies of GDC-0853 if they meet eligibility criteria. The Sponsor may evaluate whether to continue providing GDC-0853 in accordance with the Roche Global Policy on Continued Access to Investigational Medicinal Product, available at the following Web site: http://www.roche.com/policy_continued_access_to_investigational_medicines.pdf

4.4 CONCOMITANT THERAPY AND ADDITIONAL RESTRICTIONS

Concomitant therapy includes any medication (e.g., prescription drugs, over-the-counter drugs, vaccines, herbal or homeopathic remedies, nutritional supplements) used by a patient from 14 days prior to initiation of study drug to the study completion/ discontinuation visit. All such medications should be reported to the investigator and recorded on the Concomitant Medications eCRF.

4.4.1 Permitted Therapy

Patients who use oral contraceptives, hormone-replacement therapy, or other maintenance therapy should continue their use. All concomitant medications should be reported to the investigator and recorded on the appropriate eCRF. Patients will be encouraged to use the minimal dose required to control their symptoms.

The following H1 antihistamine medications are allowed:

- Cetirizine 10–40 mg QD

- Levocetirizine 5–20 mg QD
- Fexofenadine 180–720 mg QD
- Loratadine 10–40 mg QD
- Desloratidine 5–20 mg QD
- Rupatadine 10–40 mg QD
- Bilastine 10–40 mg QD
- Ebastine 10–40 mg QD

All patients will be allowed to take study-defined, second-generation, H1 antihistamine medications consistent with standard of care (i.e., up to 4 times the approved dose per local treatment guidelines) during the screening, treatment, and follow-up periods. Patients should remain on a stable H1 antihistamine regimen throughout the study period. Loratadine (10 mg) or cetirizine (10 mg) will be provided and used on an as-needed basis (maximum 1 per day) during screening, treatment, and follow-up periods). Therapies used for the treatment of CSU prior to enrollment will be collected as part of the patient's medical history.

Patients taking either LTRAs or H2 blockers for diseases other than CSU (e.g., asthma or gastroesophageal reflux disease, respectively) at screening will be permitted to continue their use at a stable dose during the study. These diseases must be recorded as part of the medical history collected during the screening period. Inhaled asthma controllers, including inhaled corticosteroids, are permitted during the study.

4.4.1.1 Dietary Supplements

For the purposes of this protocol, dietary supplements are defined as vitamins, minerals, purified food substances, and herbals with pharmaceutical properties.

Vitamins, minerals, and purified food substances are allowed in amounts not known to be associated with adverse effects (e.g., hypervitaminosis). Herbals with pharmaceutical properties are allowed only if there is acceptable evidence of no CYP3A inhibition or induction (refer to Section 4.4.2 for a list of prohibited concomitant medications, including herbal products). Otherwise, herbals with pharmaceutical properties must be discontinued for at least 4 weeks prior to the first dose of study medication, unless there are sufficient data available regarding the duration of an herbal medication's PK and PD effects to allow a shorter washout to be specified (e.g., 5 half-lives). Please direct any questions to the Medical Monitor.

4.4.1.2 Acid-Reducing Agents

Patients who use antacids (e.g., Maalox®, Pepto-Bismol®, Rolaids®) for symptomatic relief of heartburn should take GDC-0853 or matching placebo at least 2 hours before or 2 hours after antacid administration because gastric acid improves GDC-0853 absorption.

Patients may be treated with PPIs or H2RAs at up to the maximum recommended dose according to local labeling. The dose should remain stable for at least the 2 weeks before randomization and throughout the study.

At visits with scheduled PK assessments (see Appendix 1), any use of PPIs, H2RAs, and/or other antacids (e.g., Maalox®, Pepto-Bismol®, Rolaids®) should be recorded as concomitant medications, including the date and time of last administration.

4.4.2 Prohibited Therapy

Prior to the screening visit (Day −14) and during the study, including the safety follow-up period, the following medications and treatments will be restricted. Patients who receive these medications as therapy for CSU will be discontinued from the study treatment but will be followed for safety evaluation:

- Systemic or topical corticosteroids (prescription or over the counter), hydroxychloroquine, methotrexate, cyclosporine, or cyclophosphamide
- Doxepin
- Omalizumab
- IVIG
- Plasmapheresis
- LTRAs

4.4.2.1 Live or Attenuated Vaccinations

Immunization with a live or attenuated vaccine is prohibited within 6 weeks prior to study drug administration on Day 1 and for the duration of study participation, including the 4-week safety follow-up period after the administration of the last dose. See Section 5 for further details and precautions around vaccinations.

4.4.2.2 CYP3A Inhibition

In vitro studies suggest that GDC-0853 is a time-dependent inhibitor of CYP3A with inhibitory constant (K_i) values of approximately 10 μM (Study 13-0384). Although peak plasma concentrations are anticipated to be much lower than 10 μM (preliminary results from Study GA29347 indicate that a BID dose of 250-mg powder in capsule resulted in a mean steady-state C_{max} of approximately 849 nM), it is possible that GDC-0853 inhibition of CYP3A may alter the metabolism of CYP3A substrates, including estrogen derivatives such as ethinylestradiol, subsequently leading to an increase in plasma concentrations of these drugs (see the GDC-0853 Investigator's Brochure). Medications that are sensitive substrates of CYP3A or substrates of CYP3A with a narrow therapeutic window should be used with caution during this study (refer to Section 4.4.1 for a list of relevant medications).

Ethinylestradiol is metabolized by CYP3A; therefore, plasma concentrations may increase in the presence of GDC-0853. The use of hormone-replacement therapy

containing ethinylestradiol or hormonal contraceptives containing ethinylestradiol, with the concomitant use of a barrier method, is permitted during this study (see Section 4.1.1); however, these agents should be used with caution and patients should be counseled regarding the potential risks and benefit of these medications per the local prescribing information. Any increase in ethinylestradiol plasma concentrations is anticipated to be modest at most because CYP-mediated oxidation appears to be a relatively minor component of orally administered ethinylestradiol (Zhang et al. 2007). Although contraceptive efficacy is not expected to be impacted, increased ethinylestradiol plasma concentrations may lead to an increase in common side effects, such as nausea, breast tenderness, and headaches, and to a theoretical increase in rare dose-related events such as thromboembolism (Inman et al. 1970).

In vitro data suggest that GDC-0853 is metabolized by CYP3A, and there is a moderate to high potential for a drug-drug interaction with any medication that strongly inhibits or induces this enzyme. Therefore, medications in the following categories (listed in detail in Section 4.4.1) should be avoided for 7 days or 5 half-lives, whichever is longer, prior to the first dose of study drug until the last dose of study drug. If use of one of these medications is necessary, the risks and benefits should be discussed with the Medical Monitor prior to concomitant administration with study drug.

- Moderate or strong CYP3A inhibitors
- Moderate or strong CYP3A inducers

Data also suggest that GDC-0853 inhibits CYP3A, and there is a moderate to high potential for a drug-drug interaction with any medication that is metabolized by CYP3A. Plasma concentrations of the medications in the following categories (listed in detail in Section 4.4.1) may increase; therefore, they should be used with caution:

- Sensitive CYP3A substrates
- CYP3A substrates with a narrow therapeutic index

The medications listed above and in Section 4.4.1 are not necessarily comprehensive. Thus, the investigator should consult the prescribing information for any concomitant medication as well as the Internet references provided below when determining whether a certain medication is metabolized by or strongly inhibits or induces CYP3A. The investigator should contact the Medical Monitor if questions arise regarding medications not listed above.

http://www.fda.gov/downloads/Drugs/GuidanceComplianceRegulatoryInformation/Guidances/UCM292362.pdf

http://medicine.iupui.edu/clinpharm/ddis/table.aspx

4.4.3 Prohibited Food

Use of the following foods is prohibited during the study and for at least 7 days prior to initiation of study treatment: furanocoumarin derivatives as found in grapefruit, Seville orange, pomegranate, or star fruit juice or products.

4.4.4 Additional Restrictions

Patients should be fasting overnight for $>$8 hours prior to the PK draw on Days 1, 29, and 57 (see Appendix 1).

4.5 STUDY ASSESSMENTS

Please see Appendix 1 for the schedule of activities to be performed during the study.

4.5.1 Informed Consent Forms and Screening Log

Written informed consent for participation in the study must be obtained before performing any study-related procedures. Informed Consent Forms for enrolled patients and for patients who are not subsequently enrolled will be maintained at the study site.

All screening evaluations must be completed and reviewed to confirm that patients meet all eligibility criteria before enrollment. The investigator will maintain a screening log to record details of all patients screened and to confirm eligibility or record reasons for screening failure, as applicable.

4.5.2 Medical History and Demographic Data

Comprehensive medical and surgical history, including a comprehensive review of the patient's CSU medical history, will be collected at the Day −14 visit. This review will include onset of symptoms, date of diagnosis, and therapies received for CSU. In addition, history of omalizumab (Xolair®) use and reason for discontinuation will be collected.

Concomitant medical usage will be collected at all visits, including unscheduled visits. Demographic data will include age, sex, and self-reported race/ethnicity.

4.5.3 Physical Examinations

A complete physical examination should be performed at the Day −14 visit and should include an evaluation of the head, eyes, ears, nose, and throat, and the cardiovascular, dermatological, musculoskeletal, respiratory, GI, genitourinary, and neurological systems. Any abnormality identified at baseline should be recorded on the General Medical History and Baseline Conditions eCRF.

Subsequent examinations may be limited to detect changes in symptoms of CSU as well as directed by patient complaints regarding adverse events. Changes from baseline abnormalities should be recorded in patient notes. New or worsened clinically significant abnormalities should be recorded as adverse events on the Adverse Event eCRF.

4.5.4 Vital Signs

Vital signs will include measurements of heart rate, systolic and diastolic blood pressure while the patient is in a seated position, and temperature. Vital signs will be assessed as outlined in the Schedule of Activities in Appendix 1 and during other unscheduled study visits when clinically indicated. The patients' height and weight will be measured once during the screening visit.

4.5.5 FricTest

For subjects who have a history of dermographism, a FricTest® will be performed at screening (Day −14), baseline (Day 1), and Days 57 and 85. The FricTest® is a flat, plastic comb with four round-ended plastic pins, 3 mm in diameter and of different lengths. The FricTest® defines provocation thresholds and severity of dermographism (i.e., 4 pins inducing wheals defines severe dermographism).

4.5.6 Laboratory, Biomarker, and Other Biological Samples

Samples for the following laboratory tests will be sent to one or several central laboratories for analysis as per the Schedule of Activities in Appendix 1:

- Hematology: hemoglobin, hematocrit, platelet count, RBC count, WBC count, percent and absolute differential counts (neutrophils, bands, lymphocytes, monocytes, eosinophils, basophils, other cells)
- Serum chemistry: sodium, potassium, chloride, bicarbonate, glucose, BUN, creatinine, calcium, phosphorus, magnesium, total and direct bilirubin, total protein, albumin, ALT, AST, LDH, alkaline phosphatase, creatine phosphokinase, and uric acid
- Urinalysis including dipstick (pH, specific gravity, glucose, protein, ketones, blood) and microscopic examination (sediment, RBCs, WBCs, casts, crystals, epithelial cells, bacteria)
- Coagulation: INR, activated PTT, PT, fibrinogen
- Fasting lipid panel
- Viral serology
  - Hepatitis B: HBsAg, total HBcAb, and hepatitis B surface antibody
  - Hepatitis C antibody

[redacted]

The following samples will be sent to the Sponsor or a designee for analysis:

[redacted]

- Plasma samples for PK analysis

See the Schedule of Activities provided in Appendix 1 for specific timepoints.

Samples for the following laboratory tests will be sent to the study site's local laboratory for analysis:

- Pregnancy test

  All women of childbearing potential (including those who have had a tubal ligation) will have a serum pregnancy test at screening. Urine pregnancy tests will be performed at specified subsequent visits. If a urine pregnancy test result is positive, it must be confirmed by a serum pregnancy test. Should a positive result be recorded at any time, the procedures detailed in Section 5.4.3 should be followed. If a local urine pregnancy test shows a positive result, then study drug will not be administered that day. Other study procedures should also be postponed and the result must be confirmed by a serum pregnancy test prior to proceeding.

- QFT or PPD (if QFT not available) and additional procedures (e.g., chest X-ray) to rule out latent or active TB per local guidelines

See the Schedule of Activities provided in Appendix 1 for specific timepoints.

[redacted]

[redacted] [redacted]

[redacted]	[redacted]
[redacted]	[redacted]
[redacted]	[redacted]
[redacted]	[redacted]

[redacted]

For sampling procedures, storage conditions, and shipment instructions, see the laboratory manual.

Biological samples will be destroyed when the final clinical study report (CSR) has been completed, with the following exceptions:

- Plasma samples collected for PK analysis will be destroyed no later than 5 years after the final CSR has been completed.
- Blood, urine, and serum samples collected for biomarker analyses will be destroyed no later than 15 years after the final CSR has been completed.

When a patient withdraws from the study, samples collected prior to the date of withdrawal may still be analyzed, unless the patient specifically requests that the samples be destroyed or local laws require destruction of the samples.

Data arising from sample analysis will be subject to the confidentiality standards described in Section 8.4.

4.5.7 Electrocardiograms

A single ECG recording, without artifacts, must be obtained at specified timepoints, as indicated in Appendix 1. The ECG intervals (e.g., PR, QRS, QT, QTcF, and RR) and heart rate from this ECG will be entered into the eCRF. Any morphologic waveform changes or other ECG abnormalities must be documented on the eCRF. ECGs can be performed without specific restrictions (e.g., may be any time of day, before or after dosing, fasting or fed).

All ECG recordings must be performed using a standard high-quality, high-fidelity digital electrocardiograph machine equipped with computer-based interval measurements. Lead placement should be as consistent as possible. ECGs for each patient should be obtained from the same machine whenever possible. ECG recordings must be performed after the patient has been resting in a supine position for at least 10 minutes prior to beginning the ECG recording. All ECGs are to be obtained prior to other procedures scheduled at that same time (e.g., vital sign measurements, blood draws) and should not be obtained within 3 hours after any meal if possible. Body position should be consistently maintained for each ECG evaluation to prevent changes in heart rate. Circumstances that may induce changes in heart rate, including environmental distractions (e.g., television, radio, conversation) should be avoided during the pre-ECG resting period and during ECG recording.

For safety monitoring purposes, the investigator must review, sign, and date all ECG tracings. Paper copies of ECG tracings will be kept as part of the patient's permanent study file at the site. If considered appropriate by the Sponsor, ECGs may be analyzed retrospectively at a central laboratory.

If at a particular post-dose timepoint the mean QTcF is $>$500 ms and/or $>$60 ms longer than the baseline value, another ECG must be recorded, ideally within the next 5 minutes, and ECG monitoring should continue until QTcF has stabilized on two successive ECGs. The Medical Monitor should be notified as soon as possible within 24 hours. Standard-of-care treatment may be instituted per the discretion of the investigator. If a PK sample is not scheduled for that timepoint, an unscheduled PK sample should be obtained. A decision on study drug discontinuation should be made, as described in Section 4.6.2. The investigator should also evaluate the patient for potential concurrent risk factors (e.g., electrolyte abnormalities, co-medications known to prolong the QT interval, severe bradycardia).

4.5.8 Patient-Reported Outcomes

Data from two patient reported outcomes (PRO) tools will be collected via questionnaires to document the treatment benefit of GDC-0853: the Urticaria Patient Daily eDiary [redacted]. The eDiary and [redacted], translated into the local language as required, will be completed in their entirety at specified timepoints during the study. [redacted]

Patients will use an electronic device to capture the Urticaria Patient Daily eDiary (see Appendix 3). The electronic device and/or instructions for completing the questionnaires electronically will be provided by the investigator staff. The data will be transmitted to a centralized database maintained by the electronic device vendor. The data will be available for access by appropriate study personnel.

[redacted]

4.5.8.1 Urticaria Patient Daily eDiary

The Urticaria Patient Daily eDiary includes the UAS, which will be used to calculate the UAS7. The eDiary comprises questions regarding largest hive size, sleep interference score, activity interference question, rescue medication use, angioedema episodes, number of calls to doctor or nurse practitioner, and study medication compliance.

The eDiary is to be completed twice per day (a.m./p.m.) by the patient for the duration of the study. The eDiary will be given to the patient at the Day −14 visit.

4.5.8.2 Urticaria Activity Score

During the week prior to Day 1 (i.e., Week −1), UAS7 will be recorded twice daily for the purposes of enrollment eligibility. Subsequently, the UAS will be recorded twice daily using the Urticaria Patient Daily eDiary.

The UAS is a composite, eDiary-recorded score with numeric severity intensity ratings (0 = none to 3 = intense/severe) for a) the number of wheals (hives) and b) the intensity of the pruritus (itch) over the past 12 hours (twice daily; see Section 3.3.6). The UAS7 will be calculated automatically and is the weekly sum of the daily UAS, which is the composite score of the intensity of pruritus and the number of wheals. The maximum UAS7 value is 42; the intensity of the itch and the number of wheals/hives are graded in Table 2.

[redacted]

4.6 PATIENT, TREATMENT, STUDY, AND SITE DISCONTINUATION

4.6.1 Patient Discontinuation

Patients have the right to voluntarily withdraw from the study at any time for any reason. In addition, the investigator has the right to withdraw a patient from the study at any time. Reasons for withdrawal from the study may include, but are not limited to, the following:

- Patient withdrawal of consent at any time
- Any medical condition that the investigator or Sponsor determines may jeopardize the patient's safety if he or she continues in the study
- Investigator or Sponsor determines it is in the best interest of the patient
- Patient non-compliance (e.g., drug compliance ≤ 80%, missed visits, missing Urticaria Patient Daily eDiary entries) per investigator's discretion

For patients who withdraw from the study, every effort should be made to complete an early termination visit including the assessments on the Schedule of Activities (see Appendix 1). The primary reason for withdrawal from the study should be documented on the appropriate eCRF. However, patients will not be followed for any reason after consent has been withdrawn. Patients who withdraw from the study will not be replaced.

If the patient discontinues the study prior to Day 85 visit, an early termination visit should be conducted and should return for the 4-week safety follow-up visit (see Appendix 1). Patients who discontinue during the safety follow-up period prior to completion of the 4-week safety follow-up will be asked to complete an early termination visit (see Appendix 1).

If a patient withdraws for reasons related to a serious adverse event, every attempt should be made to follow the patient until resolution of the event.

4.6.2 Study Treatment Discontinuation

Patients must discontinue study treatment if they experience any of the following:

- Pregnancy
- Malignancy
- Any serious infection or infection requiring treatment with an IV antimicrobial agent
- Any prohibited medication as defined in Section 4.4.2

Patients who discontinue study treatment prematurely for the reasons listed above will be asked to return to the clinic for an early termination visit (see Section 4.6.1) followed by 4 weeks of safety follow-up (Safety Follow-up Visit at Day 85 [Week 12]).

The primary reason for study treatment discontinuation should be documented on the appropriate eCRF. Patients who discontinue study treatment prematurely will not be replaced.

4.6.3 Study and Site Discontinuation

The Sponsor has the right to terminate this study at any time. Reasons for terminating the study may include, but are not limited to, the following:

- The incidence or severity of adverse events in this or other studies indicates a potential health hazard to patients.
- Patient enrollment or completion of the study is unsatisfactory.

The Sponsor will notify the investigator if the Sponsor decides to discontinue the study.

The Sponsor has the right to close a site at any time. Reasons for closing a site may include, but are not limited to, the following:

- Excessively slow recruitment
- Poor protocol adherence
- Inaccurate or incomplete data recording
- Non-compliance with the International Council on Harmonisation (ICH) guideline for Good Clinical Practice
- No study activity (i.e., all patients have completed the study and all obligations have been fulfilled)

5. ASSESSMENT OF SAFETY

5.1 SAFETY PLAN

The safety plan for patients in this study is based on nonclinical and clinical experience with GDC-0853 in completed and ongoing studies, as well as published literature, on other BTK inhibitors and BTK biology. The important potential safety risks for GDC-0853 are outlined below. Please refer to the GDC-0853 Investigator's Brochure for a complete summary of safety information.

Several measures will be taken to ensure the safety of patients participating in this study. Eligibility criteria have been designed to exclude patients at higher risk for potential toxicities. Patients will undergo safety monitoring during the study, including monitoring of vital signs, physical examination, ECGs, and routine laboratory safety assessments (hematology, chemistry, and urinalysis) and assessment of the nature, frequency, and severity of adverse events. In addition, guidelines for managing potential adverse

events, including criteria for treatment interruption or discontinuation, and enhanced safety reporting are provided below.

5.1.1 Safety Plan for Potential Risks Associated with GDC-0853

5.1.1.1 Infections

GDC-0853 is a reversible inhibitor of BTK, and the degree to which GDC-0853 antagonism of BTK signaling may suppress immune activity is unknown. On the basis of patients with XLA, a primary immunodeficiency of B cells and immunoglobulin production, it is anticipated that inhibitors of BTK may raise the risk for certain bacterial infections (Lederman and Winkelstein 1985; Broides et al. 2006), enteroviral infections (Misbah et al. 1992; Ziegner et al. 2002), intestinal infections with giardia and *Campylobacter* species (Winkelstein et al. 2006; van den Bruele et al. 2010), or other opportunistic infections, which are cleared primarily by B-cell adaptive immune responses. This risk is likely independent of sex for patients exogenously administered GDC-0853.

Effects on lymphocytes and immunoglobulins in rats and dogs were reversible and considered to be related to pharmacological activity involving BTK inhibition. See Section 1.2.2 for related primary nonclinical toxicity findings and the GDC-0853 Investigator's Brochure for further details.

To date, no immune-challenge experiments (e.g., T-dependent antigen response test) have been conducted in animals. It is not known whether these effects on B cells and IgG concentrations in animals will translate to humans or whether such changes would have functional or deleterious impact on immune function.

Infections, including pneumonia and fatal influenza, have occurred in patients with B-cell malignancies treated with GDC-0853. In studies with healthy subjects with single doses and with dosing for 14 days, self-limited Grade 1 events of nasopharyngitis were reported but did not lead to any change in study drug dosing. One healthy subject had asymptomatic bacteriuria, which resolved while study drug dosing continued.

Patients will be excluded from the study if they have a history of hospitalization due to an infection in the 8 weeks before screening, evidence of active or latent or inadequately treated infection with *Mycobacterium* TB, known active infection (current) or history of recurrent infection, or any known immunodeficiency including IgG $<$ 500 mg/dL.

[redacted] All patients in the study should be monitored for fever and potential infectious complications, including opportunistic infections and TB, and should be evaluated promptly. Physicians or a health care provider should give patients advice to prevent potential transmission of and exposure to endemic infections according to local or Centers for Disease Control and Prevention guidelines. Patients should be advised to seek immediate medical

attention if they develop signs and symptoms suggestive of an infection. All infections occurring during the study, including but not limited to respiratory infections, cutaneous infections, urinary tract infections, systemic viral infections, and episodes of suspicious or febrile diarrhea, should be evaluated using serology or polymerase chain reaction, if available, and cultured, if feasible, and any identified organisms noted in the eCRF. Any serious infection, infection requiring IV antimicrobials, or any opportunistic infection is considered an adverse event of special interest and should be reported to the Sponsor as outlined in Section 5.4.2.

Guidelines for management of study treatment in the event that infections are observed in patients are provided in Section 5.1.2.

Please refer to the GDC-0853 Investigator's Brochure for further details.

5.1.1.2 Vaccinations

The effect of GDC-0853 upon the efficacy of vaccinations is unknown. It is recommended that appropriate vaccinations per local guidelines be up to date before study participation. Patients will be excluded from study participation and will not be dosed with GDC-0853 if they have been vaccinated with live, attenuated vaccines (e.g., the intranasal live attenuated influenza vaccines, BCG, varicella) within 6 weeks before planned dosing. In addition, immunization with a live or attenuated vaccine is prohibited for the duration of study participation, including the 4-week safety follow-up period after the administration of the last dose.

In addition, current routine household contact with children or others who have been vaccinated with live vaccine components may pose a risk to the patient during study treatment with GDC-0853. Some of these vaccines include varicella ("chickenpox") vaccine, oral polio vaccine, and the inhaled flu vaccine. Following vaccination with live component vaccines, the virus may be shed in bodily fluids, including stool, and there is a potential risk that the virus may be transmitted to the patient.

General guidelines for immunosuppressed patients suggest that exposure to vaccinated individuals should be avoided following vaccination with these vaccines for the stated time periods:

- Varicella or attenuated typhoid fever vaccination for 4 weeks following vaccination
- Oral polio vaccination for 6 weeks following vaccination
- Attenuated rotavirus vaccine for 10 days following vaccination
- FluMist® (inhaled flu vaccine) for 1 week following vaccination

Please refer to the GDC-0853 Investigator's Brochure for further details.

5.1.1.3 Bleeding

No decrease in platelets, changes in coagulation parameters, or bleeding events were observed in nonclinical studies with GDC-0853. Bleeding events, including non-serious NCI CTCAE v4.0 Grade 1 bruising and serious Grade ≥3 GI bleeding, have been reported in patients with hematological malignancies treated with GDC-0853 in Study GO29089. The GI bleeding events have not been dose related, and the events occurred in patients who were taking concomitant NSAIDs and who had a history of gastroesophageal or peptic ulcer disease. The impact of BTK inhibition as a potential risk factor for bleeding is unknown. BTK is expressed in platelets and is involved in platelet function via GPVI/collagen receptor signaling and GP1b receptor signaling. Platelets from patients with XLA, a genetic deficiency of BTK, demonstrate decreased activation in response to submaximal collagen stimulation but normal response to thrombin; clinically, there is no reported bleeding propensity of patients with XLA.

Bruising or bleeding events related to GDC-0853 have not been reported in healthy subjects.

It is unknown whether GDC-0853 will increase the risk of bleeding in patients, especially in those receiving anti-platelet or anticoagulant therapies. As a precautionary safety measure, patients will be excluded from study participation if they have a need for systemic anticoagulation with warfarin or other oral or injectable anticoagulants or anti-platelet agents (other than NSAIDs, aspirin, and other salicylates), any history of hospitalizations or transfusion for a GI bleed, any history of a hemorrhagic CVA, any history of spontaneous intracranial hemorrhage, traumatic intracranial hemorrhage within 10 years prior to the study, or a known bleeding diathesis. Patients should be advised to seek immediate medical attention if they develop signs and symptoms suggestive of clinically significant bleeding.

Bleeding events of moderate or greater severity are considered adverse events of special interest and should be reported to the Sponsor in an expedited manner as outlined in Section 5.4.2.

Guidelines for management of study treatment in the event that bleeding is observed in patients are provided in Section 5.1.2. Please refer to the GDC-0853 Investigator’s Brochure for further details.

5.1.1.4 Cytopenias

Cytopenias have been observed in patients with hematological malignancies who received GDC-0853, including neutropenia, anemia, and thrombocytopenia; events have been monitorable and clinically manageable (see the GDC-0853 Investigator’s Brochure for further details).

Patients should be monitored regularly with hematology laboratory evaluations as outlined in the schedule of activities (see Appendix 1) and should receive appropriate supportive care as clinically indicated. Patients should be advised to seek immediate medical attention if they develop signs and symptoms suggestive of cytopenias (e.g., persistent fever, bruising, bleeding, pallor). Cytopenias should be managed according to local clinical guidelines.

Guidelines for managing study treatment in the event that cytopenia is observed are provided in Section 5.1.2. Please refer to the GDC-0853 Investigator's Brochure for further details.

5.1.1.5 Gastrointestinal Effects

Body weight gain and food consumption changes have been observed in animals, including nonsignificant increases in male Wistar-Han rats administered ≥ 2 mg/kg/day (4.3 μM • hr) for 6 months, and significant reductions in rats administered 100 mg/kg/day (1438 μM • hr) and dogs administered 25 mg/kg (180 μM • hr) for 4 weeks. These effects on body weight gain and food consumption were reversible following discontinuation of GDC-0853 dosing.

NCI CTCAE v4.0 Grade 1 diarrhea, nausea, and abdominal pain have been reported in patients with B-cell malignancies; however, the events have resolved and have not led to study drug discontinuation. Healthy subjects in the MAD Study GA29347 reported events of mild self-limited nausea.

Throughout the study, patients will be monitored for GI side effects.

Guidelines for management of study treatment in the event of GI side effects in patients are provided in Section 5.1.1.5. Please refer to the GDC-0853 Investigator's Brochure for further details.

5.1.1.6 Hepatotoxicity

Evidence of hepatobiliary injury was observed in animals administered relatively high doses of GDC-0853 in repeat-dose toxicity studies. Dose-dependent increases in ALT, AST, and/or bilirubin have been observed in rats administered ≥ 6 mg/kg/day (≥ 17 μM • hr) and dogs administered ≥ 10 mg/kg/day (≥ 36 μM • hr), with corresponding microscopic changes in the liver of dogs administered 25 mg/kg/day (180 μM • hr). The hepatotoxicity findings in dogs were associated with moribundity in two high-dose animals. The NOAEL for these findings was considered to be 10 mg/kg (36 μM • hr) in dogs, the most sensitive species, given the absence of GDC-0853–related hepatotoxicity at this dose when administered for 9 months. This exposure provides a 5-fold safety multiple above the study dose of 200 mg BID (projected $AUC_{0\text{-}24}$ of 6.7 μM • hr). The hepatotoxicity findings were fully reversible and considered monitorable by changes in

plasma transaminases and bilirubin that occurred at doses lower than those producing histopathology findings (see the GDC-0853 Investigator's Brochure for further details).

In clinical studies to date, including single dosing and multiple dosing for 14 days in healthy subjects and QD dosing for over 2 years in patients with hematological malignancies, there have been no adverse events of liver enzyme elevations or trends toward elevations in laboratory evaluations.

As a safety risk-mitigation measure, to be eligible for the study, AST and/or ALT levels should be no more than 1.5 × ULN, and total bilirubin levels should be normal at screening. Safety monitoring for potential hepatotoxicity includes baseline and routine evaluations of AST/ALT and total bilirubin levels throughout the study as outlined in the schedule of activities (see Appendix 1).

Laboratory results of either an AST or ALT > 5 × ULN or an AST or ALT > 3 × ULN in combination with a total bilirubin > 2 × ULN, of which at least 35% is direct bilirubin or there is clinical jaundice, are considered adverse events of special interest and should be reported to the Sponsor in an expedited manner as outlined in Section 5.4.2.

Guidelines for the management of study treatment in the event of hepatotoxicity in patients are provided in Section 5.1.2. Please refer to the GDC-0853 Investigator's Brochure for further details.

5.1.1.7 Cardiovascular Effects

GDC-0853 is considered to have a low potential to cause QT interval prolongation or to directly affect other cardiovascular parameters, at therapeutic exposures. A minimal increase in QTc (7 ms or 3%) interval was noted at 45 mg/kg in the single-dose cardiovascular safety pharmacology study in telemetry-instrumented dogs. Based on extrapolated/interpolated toxicokinetic data, the unbound C_{max} at 45 mg/kg (considered a NOAEL) and no-observed–effect level of 15 mg/kg were 23-fold and 7-fold higher, respectively, than the mean unbound C_{max} in humans at the 200-mg BID dose. There have been no GDC-0853–related changes in ECG parameters in the 4-week or 9-month dog toxicity studies.

Analysis of ECG data from the SAD and MAD studies in healthy subjects did not demonstrate any significant increase in either QRS interval or QTcF intervals. However, cardiac safety will be evaluated in all patients at baseline and throughout this study, with routine monitoring of vital signs (including heart rate and blood pressure), routine safety ECGs, and collection of adverse events (see Section 5.1.1.7 and Section 5.2.1).

Management of patients with sustained QTcF prolongation (QTcF that is > 500 ms and/or > 60 ms longer than the baseline value) should include recording another ECG, ideally within the next 5 minutes, and ECG monitoring should continue until QTcF has

stabilized on two successive ECGs. The Medical Monitor should be notified as soon as possible within 24 hours. Standard-of-care treatment may be instituted per the discretion of the investigator. If a PK sample is not scheduled for that timepoint, an unscheduled PK sample should be obtained. A decision on study drug discontinuation should be made, as described in Section 4.6.2. The investigator should also evaluate the patient for potential concurrent risk factors (e.g., electrolyte abnormalities, co-medications known to prolong the QT interval, severe bradycardia).

Guidelines for management of study treatment in the event in the event of cardiovascular effects in patients are provided in Section 5.1.2. Please refer to the GDC-0853 Investigator's Brochure for further details.

5.1.1.8 Vascular Inflammation

Vascular inflammation (vasculitis) was observed in dogs administered GDC-0853 at ≥10 mg/kg/day (≥56 μM•hr) in the 4-week toxicity study, and these changes were not completely reversed by the end of the 4-week recovery period. There was no consistent correlation with any clinical biomarkers. However, in the 9-month toxicity study in dogs, no GDC-0853–related vascular inflammation was observed up to the highest dose of 10 mg/kg/day (36 μM•hr), which is considered to be the NOAEL (AUC) for the canine vascular inflammation findings. This exposure provides a 5-fold safety multiple above the study dose of 200 mg BID (projected AUC_{0-24} of 6.7 μM•hr). The translatability of these findings to humans is unknown; however, Beagle dogs are susceptible to spontaneous development of polyarteritis syndrome (Snyder et al. 1995) and may be more sensitive to any drug-induced effects. Further, there are several examples of approved therapies for which there is no correlation between the finding of vasculitis in dogs or rats at clinically relevant exposures and adverse outcomes in patients (FDA 2011). Guidelines for management of study treatment in the event of vasculitis in patients are provided in Section 5.1.2. Please refer to the GDC-0853 Investigator's Brochure for further details.

5.1.1.9 Malignancy

The impact of BTK inhibition on the development of malignancies is not known; however, malignancies have been identified as a potential concern for immunomodulatory agents. Malignancies have been reported in patients with XLA, including lymphoreticular malignancies, gastric and colorectal adenocarcinoma, and squamous cell carcinoma of the lung.

Patients with a history of cancer, including hematologic malignancy and solid tumors, within 10 years before screening will be excluded from the study. Basal or squamous cell carcinoma of the skin that has been excised and is considered cured and in situ carcinoma of the cervix treated with apparent success by curative therapy more than 1 year prior to screening are not exclusionary.

All malignancies are adverse events of special interest and should be reported to the Sponsor in an expedited manner as outlined in Section 5.2.3.

Guidelines for management of study treatment in the event of malignancies in patients are provided in Section 5.1.2. Please refer to the GDC-0853 Investigator's Brochure for further details.

5.1.2 Management of Patients Who Experience Specific Adverse Events

5.1.2.1 Management of Specific Adverse Events

Guidelines for management of specific adverse events are outlined in Table 4.

Table 4 Guidelines for Management of Patients Who Experience Specific Adverse Events

Event	Action to be Taken [a]
**Infection [b]**	
Serious infection, opportunistic infection, or any infection requiring treatment with an IV antimicrobial agent	Discontinue study treatment and report event as an adverse event of special interest.
Self-limited infections that require treatment	Withhold study treatment during antimicrobial therapy. Study treatment may resume after consultation with the Medical Monitor.
Bleeding	Bleeding events of moderate or greater severity are considered adverse events of special interest and should be reported to the Sponsor in an expedited manner.<br>For serious bleeding events or bleeding events requiring transfusion, radiologic endoscopic, or elective operative intervention, withhold study treatment and consult with the Medical Monitor.

Table 4 Guidelines for Management of Patients Who Experience Specific Adverse Events (cont.)

Event	Action to be Taken [a]
**Gastrointestinal effects**	
Nausea, vomiting, and/or diarrhea	Manage according to site institutional guidelines.<br>Consider administration of study treatment with food as a possible mitigation strategy.
**Malignancy**	
Any malignancy	Discontinue study treatment, with the exception of non-serious local and resectable basal or squamous cell carcinoma of the skin. Report event as an adverse event of special interest to the Sponsor in an expedited manner.
**Hepatotoxicity**	
AST or ALT > 3.0–5.0 × ULN	Withhold study treatment and consult with the Medical Monitor.
AST or ALT > 3 × ULN in combination with a total bilirubin > 2 × ULN, of which at least 35% is direct bilirubin, or clinical jaundice	Discontinue study treatment. Report event(s) as adverse event of special interest (Hy's law) to the Sponsor in an expedited manner.
AST or ALT > 5 × ULN	Discontinue study treatment. Any elevation of an AST or ALT > 5 × ULN should be reported as an adverse event of special interest to the Sponsor in an expedited manner.
**Cardiovascular effects**	
Sustained (at least two ECG measurements > 30 minutes apart) QTcF that is > 500 ms and/or > 60 ms longer than the baseline value	Unless there is a clear alternative cause other than study drug, discontinue study treatment. [c]
Sustained absolute QTcF that is > 515 ms	Unless there is a clear alternative cause other than study drug, discontinue study treatment. [c]
An episode of torsades de pointes or a new ECG finding of clinical concern	Unless there is a clear alternative cause other than study drug, discontinue study treatment. [c]
**Vascular inflammation**	
Vasculitis	Discontinue study treatment and consult with the Medical Monitor.

IV = intravenous; QTcF = QT interval corrected using Fridericia's formula; ULN = upper limit of normal.

Note: "Study treatment" includes study drug (GDC-0853 or placebo).

[a] Any patient who discontinues study treatment should enter safety follow-up, if possible.

[b] Appropriate laboratory investigations, including but not limited to cultures, should be performed to establish the etiology of any serious infection.

[c] In rare circumstances, it may be acceptable to resume study drug, provided that any ECG abnormalities have resolved and that the patient is appropriately monitored. Clinical judgment should be applied.

5.1.2.2 Management of Increases in QT Interval

Study drug should be discontinued in patients who develop any of the following:

- Sustained (at least two ECG measurements > 30 minutes apart) QTcF that is > 500 ms and > 60 ms longer than the baseline value
- Sustained absolute QTcF that is > 515 ms
- An episode of torsades de pointes or a new ECG finding of clinical concern

Of note, if there is a new intraventricular conduction block, the increase in QRS complex duration should be subtracted from the QTcF change, because this represents an increase in QTcF unrelated to alterations in repolarization. Also of note, it is not uncommon to record arrhythmias, such as non-sustained ventricular tachycardia, supraventricular tachycardia, pauses, or atrial fibrillation, in healthy volunteers receiving placebo during periods of extended ECG monitoring. Therefore, it is critical that expert cardiology advice be sought to confirm any ECG changes and to ascertain the likelihood of a drug-induced arrhythmia versus the background occurrence of this arrhythmia. In such a situation, saving all available ECG data is highly suggested.

Management of patients with sustained QTcF prolongation should include close monitoring, with ECGs repeated at least hourly until two successive ECGs show resolution of the findings, correction of any electrolyte abnormalities, and possible discontinuation of other concomitant medications that are known to prolong the QT interval. Consultation with a cardiologist or electrophysiologist is recommended to help in the management of such patients.

5.2 SAFETY PARAMETERS AND DEFINITIONS

Safety assessments will consist of monitoring and recording adverse events, including serious adverse events and adverse events of special interest, performing protocol-specified safety laboratory assessments, measuring protocol-specified vital signs, and conducting other protocol-specified tests that are deemed critical to the safety evaluation of the study.

Certain types of events require immediate reporting to the Sponsor, as outlined in Section 5.4.

5.2.1 Adverse Events

According to the ICH guideline for Good Clinical Practice, an adverse event is any untoward medical occurrence in a clinical investigation subject administered a pharmaceutical product, regardless of causal attribution. An adverse event can therefore be any of the following:

- Any unfavorable and unintended sign (including an abnormal laboratory finding), symptom, or disease temporally associated with the use of a medicinal product, whether or not considered related to the medicinal product

- Any new disease or exacerbation of an existing disease (a worsening in the character, frequency, or severity of a known condition), except as described in Section 5.3.5.10
- Recurrence of an intermittent medical condition (e.g., headache) not present at baseline
- Any deterioration in a laboratory value or other clinical test (e.g., ECG, X-ray) that is associated with symptoms or leads to a change in study treatment or concomitant treatment or discontinuation from study drug
- Adverse events that are related to a protocol-mandated intervention, including those that occur prior to assignment of study treatment (e.g., screening invasive procedures such as biopsies)

5.2.2 Serious Adverse Events (Immediately Reportable to the Sponsor)

A serious adverse event is any adverse event that meets any of the following criteria:

- Is fatal (i.e., the adverse event actually causes or leads to death)
- Is life threatening (i.e., the adverse event, in the view of the investigator, places the patient at immediate risk of death)

  This does not include any adverse event that had it occurred in a more severe form or was allowed to continue might have caused death.
- Requires or prolongs inpatient hospitalization (see Section 5.3.5.11)
- Results in persistent or significant disability/incapacity (i.e., the adverse event results in substantial disruption of the patient's ability to conduct normal life functions)
- Is a congenital anomaly/birth defect in a neonate/infant born to a mother exposed to study drug
- Is a significant medical event in the investigator's judgment (e.g., may jeopardize the patient or may require medical/surgical intervention to prevent one of the outcomes listed above)

The terms "severe" and "serious" are not synonymous. Severity refers to the intensity of an adverse event (e.g., rated as mild, moderate, or severe; see Section 5.3.3); the event itself may be of relatively minor medical significance (such as severe headache without any further findings).

Severity and seriousness need to be independently assessed for each adverse event recorded on the eCRF.

Serious adverse events are required to be reported by the investigator to the Sponsor immediately (i.e., no more than 24 hours after learning of the event; see Section 5.4.2 for reporting instructions).

5.2.3 Adverse Events of Special Interest (Immediately Reportable to the Sponsor)

Adverse events of special interest are required to be reported by the investigator to the Sponsor immediately (i.e., no more than 24 hours after learning of the event; see Section 5.4.2 for reporting instructions). Adverse events of special interest for this study include the following:

- Any serious infection, any infections requiring IV antimicrobials and any opportunistic infections
- Bleeding events of moderate or greater severity
- All malignancies
- Adverse events of special interest for general drug development
  - A laboratory result of AST or ALT $>5\times$ ULN
  - Cases of potential drug-induced liver injury that include an ALT or AST $>3\times$ ULN in combination with a total bilirubin $>2\times$ ULN, of which at least 35% is direct bilirubin or there is clinical jaundice, as defined by Hy's law (see Section 5.1.1.6)
- Suspected transmission of an infectious agent by the study drug, as defined below:

  Any organism, virus, or infectious particle (e.g., prion protein transmitting transmissible spongiform encephalopathy), pathogenic or non-pathogenic, is considered an infectious agent. A transmission of an infectious agent may be suspected from clinical symptoms or laboratory findings that indicate an infection in a patient exposed to a medicinal product. This term applies only when a contamination of the study drug is suspected.

5.3 METHODS AND TIMING FOR CAPTURING AND ASSESSING SAFETY PARAMETERS

The investigator is responsible for ensuring that all adverse events (see Section 5.2.1 for definition) are recorded on the Adverse Event eCRF and reported to the Sponsor in accordance with instructions provided in this section and in Section 5.4 – Section 5.6.

For each adverse event recorded on the Adverse Event eCRF, the investigator will make an assessment of seriousness (see Section 5.2.2), severity (see Section 5.3.3), and causality (see Section 5.3.4).

5.3.1 Adverse Event Reporting Period

Investigators will seek information on adverse events at each patient contact. All adverse events, whether reported by the patient or noted by study personnel, will be recorded in the patient's medical record and on the Adverse Event eCRF.

After informed consent has been obtained **but prior to initiation of study drug**, only serious adverse events caused by a protocol-mandated intervention (e.g., invasive

procedures such as biopsy sample collection, discontinuation of medications) should be reported (see Section 5.4.2 for instructions for reporting serious adverse events).

After initiation of study drug, all adverse events will be reported until 4 weeks after the last dose of study drug the patients receives. After this period, the investigator should report any serious adverse events that are believed to be related to prior study drug treatment (see Section 5.4.2).

5.3.2 Eliciting Adverse Event Information

A consistent methodology of non-directive questioning should be adopted for eliciting adverse event information at all patient evaluation timepoints. Examples of non-directive questions include the following:

"How have you felt since your last clinic visit?"

"Have you had any new or changed health problems since you were last here?"

5.3.3 Assessment of Severity of Adverse Events

Table 5 provides the adverse event grading scale for severity.

Table 5 Adverse Event Severity Grading Scale

Severity	Description
Mild	Discomfort noticed, but no disruption of normal daily activity
Moderate	Discomfort sufficient to reduce or affect normal daily activity
Severe	Incapacitating with inability to work or to perform normal daily activity

Note: Regardless of severity, some events may also meet seriousness criteria. Refer to definition of a serious adverse event (see Section 5.2.2).

5.3.4 Assessment of Causality of Adverse Events

Investigators should use their knowledge of the patient, the circumstances surrounding the event, and an evaluation of any potential alternative causes to determine whether an adverse event is considered to be related to the study drug, indicating "yes" or "no" accordingly. The following guidance should be taken into consideration (see also Table 6):

- Temporal relationship of event onset to the initiation of study drug
- Course of the event, with special consideration of the effects of dose reduction, discontinuation of study drug, or reintroduction of study drug (as applicable)
- Known association of the event with the study drug or with similar treatments
- Known association of the event with the disease under study
- Presence of risk factors in the patient or use of concomitant medications known to increase the occurrence of the event

- Presence of non-treatment–related factors that are known to be associated with the occurrence of the event

Table 6 Causal Attribution Guidance

Is the adverse event suspected to be caused by the study drug on the basis of facts, evidence, science-based rationales, and clinical judgment?	
YES	There is a plausible temporal relationship between the onset of the adverse event and administration of the study drug, and the adverse event cannot be readily explained by the patient's clinical state, intercurrent illness, or concomitant therapies; and/or the adverse event follows a known pattern of response to the study drug; and/or the adverse event abates or resolves upon discontinuation of the study drug or dose reduction and, if applicable, reappears upon re-challenge.
NO	An adverse event will be considered related, unless it fulfills the criteria specified below. Evidence exists that the adverse event has an etiology other than the study drug (e.g., preexisting medical condition, underlying disease, intercurrent illness, or concomitant medication); and/or the adverse event has no plausible temporal relationship to administration of the study drug (e.g., cancer diagnosed 2 days after first dose of study drug).

For patients receiving combination therapy, causality will be assessed individually for each protocol-mandated therapy.

5.3.5 Procedures for Recording Adverse Events

Investigators should use correct medical terminology/concepts when recording adverse events on the Adverse Event eCRF. Avoid colloquialisms and abbreviations.

Only one adverse event term should be recorded in the event field on the Adverse Event eCRF.

5.3.5.1 Diagnosis versus Signs and Symptoms

A diagnosis (if known) should be recorded on the Adverse Event eCRF rather than individual signs and symptoms (e.g., record only liver failure or hepatitis rather than jaundice, asterixis, and elevated transaminases). However, if a constellation of signs and/or symptoms cannot be medically characterized as a single diagnosis or syndrome at the time of reporting, each individual event should be recorded on the Adverse Event eCRF. If a diagnosis is subsequently established, all previously reported adverse events based on signs and symptoms should be nullified and replaced by one adverse event report based on the single diagnosis, with a starting date that corresponds to the starting date of the first symptom of the eventual diagnosis.

5.3.5.2 Adverse Events That Are Secondary to Other Events

In general, adverse events that are secondary to other events (e.g., cascade events or clinical sequelae) should be identified by their primary cause, with the exception of severe or serious secondary events. A medically significant secondary adverse event

that is separated in time from the initiating event should be recorded as an independent event on the Adverse Event eCRF. For example:

- If vomiting results in mild dehydration with no additional treatment in a healthy adult, only vomiting should be reported on the eCRF.
- If vomiting results in severe dehydration, both events should be reported separately on the eCRF.
- If a severe GI hemorrhage leads to renal failure, both events should be reported separately on the eCRF.
- If dizziness leads to a fall and consequent fracture, all three events should be reported separately on the eCRF.
- If neutropenia is accompanied by an infection, both events should be reported separately on the eCRF.

All adverse events should be recorded separately on the Adverse Event eCRF if it is unclear as to whether the events are associated.

5.3.5.3 Persistent or Recurrent Adverse Events

A persistent adverse event is one that extends continuously, without resolution, between patient evaluation timepoints. Such events should be recorded only once on the Adverse Event eCRF. The initial severity (intensity or grade) of the event will be recorded at the time the event is first reported. If a persistent adverse event becomes more severe, the most extreme severity should also be recorded on the Adverse Event eCRF. If the event becomes serious, it should be reported to the Sponsor immediately (i.e., no more than 24 hours after learning that the event became serious; see Section 5.4.2 for reporting instructions). The Adverse Event eCRF should be updated by changing the event from "non-serious" to "serious," providing the date that the event became serious and completing all data fields related to serious adverse events.

A recurrent adverse event is one that resolves between patient evaluation timepoints and subsequently recurs. Each recurrence of an adverse event should be recorded as a separate event on the Adverse Event eCRF.

5.3.5.4 Abnormal Laboratory Values

Not every laboratory abnormality qualifies as an adverse event. A laboratory test result must be reported as an adverse event if it meets any of the following criteria:

- Is accompanied by clinical symptoms
- Results in a change in study treatment (e.g., dosage modification, treatment interruption, or treatment discontinuation)
- Results in a medical intervention (e.g., potassium supplementation for hypokalemia) or a change in concomitant therapy
- Is clinically significant in the investigator’s judgment

It is the investigator's responsibility to review all laboratory findings. Medical and scientific judgment should be exercised in deciding whether an isolated laboratory abnormality should be classified as an adverse event.

If a clinically significant laboratory abnormality is a sign of a disease or syndrome (e.g., alkaline phosphatase and bilirubin 5 × ULN associated with cholestasis), only the diagnosis (i.e., cholestasis) should be recorded on the Adverse Event eCRF.

If a clinically significant laboratory abnormality is not a sign of a disease or syndrome, the abnormality itself should be recorded on the Adverse Event eCRF, along with a descriptor indicating whether the test result is above or below the normal range (e.g., "elevated potassium," as opposed to "abnormal potassium"). If the laboratory abnormality can be characterized by a precise clinical term per standard definitions, the clinical term should be recorded as the adverse event. For example, an elevated serum potassium level of 7.0 mEq/L should be recorded as "hyperkalemia."

Observations of the same clinically significant laboratory abnormality from visit to visit should only be recorded once on the Adverse Event eCRF (see Section 5.3.5.3 for details on recording persistent adverse events).

5.3.5.5 Abnormal Vital Sign Values

Not every vital sign abnormality qualifies as an adverse event. A vital sign result must be reported as an adverse event if it meets any of the following criteria:

- Is accompanied by clinical symptoms
- Results in a change in study treatment (e.g., dosage modification, treatment interruption, or treatment discontinuation)
- Results in a medical intervention or a change in concomitant therapy
- Is clinically significant in the investigator's judgment

It is the investigator's responsibility to review all vital sign findings. Medical and scientific judgment should be exercised in deciding whether an isolated vital sign abnormality should be classified as an adverse event.

If a clinically significant vital sign abnormality is a sign of a disease or syndrome (e.g., high blood pressure), only the diagnosis (i.e., hypertension) should be recorded on the Adverse Event eCRF.

Observations of the same clinically significant vital sign abnormality from visit to visit should only be recorded once on the Adverse Event eCRF (see Section 5.3.5.3 for details on recording persistent adverse events).

5.3.5.6 Abnormal Liver Function Tests

The finding of an elevated ALT or AST ($> 3 \times$ baseline value) in combination with either an elevated total bilirubin ($> 2 \times$ ULN) or clinical jaundice in the absence of cholestasis or other causes of hyperbilirubinemia is considered to be an indicator of severe liver injury (as defined by Hy's law). Therefore, investigators must report as an adverse event the occurrence of either of the following:

- Treatment-emergent ALT or AST $> 3 \times$ baseline value in combination with total bilirubin $> 2 \times$ ULN (of which $\geq 35\%$ is direct bilirubin)
- Treatment-emergent ALT or AST $> 3 \times$ baseline value in combination with clinical jaundice

The most appropriate diagnosis or (if a diagnosis cannot be established) the abnormal laboratory values should be recorded on the Adverse Event eCRF (see Section 5.3.5.2) and reported to the Sponsor immediately (i.e., no more than 24 hours after learning of the event), either as a serious adverse event or an adverse event of special interest (see Section 5.4.2).

5.3.5.7 Deaths

All deaths that occur during the protocol-specified adverse event reporting period (see Section 5.3.1), regardless of relationship to study drug, must be recorded on the Adverse Event eCRF and immediately reported to the Sponsor (see Section 5.4.2). This includes death attributed to progression of CSU.

Death should be considered an outcome and not a distinct event. The event or condition that caused or contributed to the fatal outcome should be recorded as the single medical concept on the Adverse Event eCRF. Generally, only one such event should be reported. The term **"sudden death"** should be used only for the occurrence of an abrupt and unexpected death due to presumed cardiac causes in a patient with or without preexisting heart disease, within 1 hour after the onset of acute symptoms or, in the case of an unwitnessed death, within 24 hours after the patient was last seen alive and stable. If the cause of death is unknown and cannot be ascertained at the time of reporting, **"unexplained death"** should be recorded on the Adverse Event eCRF. If the cause of death later becomes available (e.g., after autopsy), "unexplained death" should be replaced by the established cause of death.

If the death is attributed to angioedema of CSU, "CSU angioedema" should be recorded on the Adverse Event eCRF.

Deaths that occur after the adverse event reporting period should be reported as described in Section 5.6.

5.3.5.8 Preexisting Medical Conditions

A preexisting medical condition is one that is present at the screening visit for this study. Such conditions should be recorded on the General Medical History and Baseline Conditions eCRF.

A preexisting medical condition should be recorded as an adverse event only if the frequency, severity, or character of the condition worsens during the study. When recording such events on the Adverse Event eCRF, it is important to convey the concept that the preexisting condition has changed by including applicable descriptors (e.g., "more frequent headaches").

5.3.5.9 Lack of Efficacy or Worsening of Chronic Spontaneous Urticaria

Medical occurrences or symptoms of deterioration that are anticipated as part of CSU should be recorded as an adverse event if judged by the investigator to have unexpectedly worsened in severity or frequency or changed in nature compared to baseline at any time during the study. When recording an unanticipated worsening of CSU on the Adverse Event eCRF, it is important to convey the concept that the condition has changed by including applicable descriptors (e.g., "accelerated CSU").

5.3.5.10 Hospitalization or Prolonged Hospitalization

Any adverse event that results in hospitalization (i.e., inpatient admission to a hospital) or prolonged hospitalization should be documented and reported as a serious adverse event (per the definition of serious adverse event in Section 5.2.2), except as outlined below.

The following hospitalization scenarios are not considered to be adverse events:

- Hospitalization for respite care
- Hospitalization for a preexisting condition, provided that all of the following criteria are met:
  - The hospitalization was planned prior to the study or was scheduled during the study when elective surgery became necessary because of the expected normal progression of the disease
  - The patient has not experienced an adverse event

The following hospitalization scenarios are not considered to be serious adverse events, but should be reported as adverse events instead:

- Hospitalization that was necessary because of patient requirement for outpatient care outside of normal outpatient clinic operating hours. For this scenario, record the underlying medical condition which resulted in hospitalization on the Adverse Event eCRF.

5.3.5.11 Adverse Events Associated with an Overdose or Error in Drug Administration

An overdose is the accidental or intentional use of a drug in an amount higher than the dose being studied. An overdose or incorrect administration of study treatment is not itself an adverse event, but it may result in an adverse event. All adverse events associated with an overdose or incorrect administration of study drug should be recorded on the Adverse Event eCRF. If the associated adverse event fulfills seriousness criteria, the event should be reported to the Sponsor immediately (i.e., no more than 24 hours after learning of the event; see Section 5.4.2).

No safety data related to overdosing of GDC-0853 are available.

5.3.5.12 Patient-Reported Outcome Data

Adverse event reports will not be derived from PRO data by the Sponsor, and safety analyses will not be performed using PRO data. However, if any PRO responses suggestive of a possible adverse event are identified during site review of the PRO data, the investigator will determine whether the criteria for an adverse event have been met and, if so, will report the event on the Adverse Event eCRF.

5.4 IMMEDIATE REPORTING REQUIREMENTS FROM INVESTIGATOR TO SPONSOR

Certain events require immediate reporting to allow the Sponsor to take appropriate measures to address potential new risks in a clinical trial. The investigator must report such events to the Sponsor immediately; under no circumstances should reporting take place more than 24 hours after the investigator learns of the event. The following is a list of events that the investigator must report to the Sponsor within 24 hours after learning of the event, regardless of relationship to study drug:

- Serious adverse events (see Section 5.4.2 for further details)
- Adverse events of special interest (see Section 5.4.2 for further details)
- Pregnancies (see Section 5.4.3 for further details)

The investigator must report new significant follow-up information for these events to the Sponsor immediately (i.e., no more than 24 hours after becoming aware of the information). New significant information includes the following:

- New signs or symptoms or a change in the diagnosis
- Significant new diagnostic test results
- Change in causality based on new information
- Change in the event’s outcome, including recovery
- Additional narrative information on the clinical course of the event

Investigators must also comply with local requirements for reporting serious adverse events to the local health authority and Institutional Review Board or Ethics Committee (IRB/EC).

5.4.1 Emergency Medical Contacts

Medical Monitor Contact Information

Medical Monitor contact information:

Medical Monitor: [redacted]

Telephone Nos.: [redacted]

5.4.2 Reporting Requirements for Serious Adverse Events and Adverse Events of Special Interest

5.4.2.1 Events That Occur prior to Study Drug Initiation

After informed consent has been obtained but prior to initiation of study drug, only serious adverse events caused by a protocol-mandated intervention should be reported. The Serious Adverse Event/Adverse Event of Special Interest Reporting Form provided to investigators should be completed and submitted to the Sponsor or its designee immediately (i.e., no more than 24 hours after learning of the event), either by faxing or by scanning and emailing the form using the fax number or email address provided to investigators.

5.4.2.2 Events That Occur after Study Drug Initiation

After initiation of study drug, serious adverse events and adverse events of special interest will be reported until 4 weeks after the last dose of study drug. Investigators should record all case details that can be gathered immediately (i.e., within 24 hours after learning of the event) on the Adverse Event eCRF and submit the report via the electronic data capture (EDC) system. A report will be generated and sent to Safety Risk Management by the EDC system.

In the event that the EDC system is unavailable, the Serious Adverse Event/Adverse Event of Special Interest Reporting Form provided to investigators should be completed and submitted to the Sponsor or its designee immediately (i.e., no more than 24 hours after learning of the event), either by faxing or by scanning and emailing the form using the fax number or email address provided to investigators. Once the EDC system is available, all information will need to be entered and submitted via the EDC system.

Instructions for reporting serious adverse events that occur $>$4 weeks after the last dose of study treatment are provided in Section 5.6.

5.4.3 Reporting Requirements for Pregnancies

5.4.3.1 Pregnancies in Female Patients

Female patients of childbearing potential will be instructed to immediately inform the investigator if they become pregnant during the study or within 4 weeks after the last dose of study drug. A Clinical Trial Pregnancy Reporting Form should be completed and submitted to the Sponsor or its designee immediately (i.e., no more than 24 hours after learning of the pregnancy), either by faxing or by scanning and emailing the form using the fax number or email address provided to investigators. Pregnancy should not be recorded on the Adverse Event eCRF. The investigator should discontinue study drug and counsel the patient, discussing the risks of the pregnancy and the possible effects on the fetus. Monitoring of the patient should continue until conclusion of the pregnancy. Any serious adverse events associated with the pregnancy (e.g., an event in the fetus, an event in the mother during or after the pregnancy, or a congenital anomaly/birth defect in the child) should be reported on the Adverse Event eCRF. In addition, the investigator will submit a Clinical Trial Pregnancy Reporting Form when updated information on the course and outcome of the pregnancy becomes available.

5.4.3.2 Pregnancies in Female Partners of Male Patients

Male patients will be instructed through the Informed Consent Form to immediately inform the investigator if their partner becomes pregnant during the study or within 4 weeks after the last dose of study drug. A Clinical Trial Pregnancy Reporting Form should be completed and submitted to the Sponsor or its designee immediately (i.e., no more than 24 hours after learning of the pregnancy), either by faxing or by scanning and emailing the form using the fax number or email address provided to investigators. Attempts should be made to collect and report details of the course and outcome of any pregnancy in the partner of a male patient exposed to study drug. The pregnant partner will need to sign an Authorization for Use and Disclosure of Pregnancy Health Information to allow for follow-up on her pregnancy. After the authorization has been signed, the investigator will submit a Clinical Trial Pregnancy Reporting Form when updated information on the course and outcome of the pregnancy becomes available. An investigator who is contacted by the male patient or his pregnant partner may provide information on the risks of the pregnancy and the possible effects on the fetus, to support an informed decision in cooperation with the treating physician and/or obstetrician.

5.4.3.3 Congenital Anomalies/Birth Defects and Abortions

Any congenital anomaly/birth defect in a child born to a female patient exposed to study drug or the female partner of a male patient exposed to study drug should be classified as a serious adverse event, recorded on the Adverse Event eCRF, and reported to the Sponsor immediately (i.e., no more than 24 hours after learning of the event; see Section 5.4.2). Any abortion should be reported in the same fashion (as the Sponsor considers abortions to be medically significant).

5.5 FOLLOW-UP OF PATIENTS AFTER ADVERSE EVENTS

5.5.1 Investigator Follow-Up

The investigator should follow each adverse event until the event has resolved to baseline grade or better, the event is assessed as stable by the investigator, the patient is lost to follow-up, or the patient withdraws consent. Every effort should be made to follow all serious adverse events considered to be related to study drug or trial-related procedures until a final outcome can be reported.

During the study period, resolution of adverse events (with dates) should be documented on the Adverse Event eCRF and in the patient's medical record to facilitate source data verification.

All pregnancies reported during the study should be followed until pregnancy outcome.

5.5.2 Sponsor Follow-Up

For serious adverse events, adverse events of special interest, and pregnancies, the Sponsor or a designee may follow up by telephone, fax, email, and/or a monitoring visit to obtain additional case details and outcome information (e.g., from hospital discharge summaries, consultant reports, autopsy reports) in order to perform an independent medical assessment of the reported case.

5.6 ADVERSE EVENTS THAT OCCUR AFTER THE ADVERSE EVENT REPORTING PERIOD

The Sponsor should be notified if the investigator becomes aware of any serious adverse event that occurs after the end of the adverse event reporting period (defined as 4 weeks after the last dose of study drug; see Section 5.3.1), if the event is believed to be related to prior study drug treatment. These events should be reported through use of the Adverse Event eCRF. However, if the EDC system is not available, the investigator should report these events directly to the Sponsor or its designee, either by faxing or by scanning and emailing the Serious Adverse Event/Adverse Event of Special Interest Reporting Form using the fax number or email address provided to investigators.

5.7 EXPEDITED REPORTING TO HEALTH AUTHORITIES, INVESTIGATORS, INSTITUTIONAL REVIEW BOARDS, AND ETHICS COMMITTEES

The Sponsor will promptly evaluate all serious adverse events and adverse events of special interest against cumulative product experience to identify and expeditiously communicate possible new safety findings to investigators, IRBs, ECs, and applicable health authorities based on applicable legislation.

To determine reporting requirements for single adverse event cases, the Sponsor will assess the expectedness of these events using the following reference document:

- GDC-0853 Investigator's Brochure

The Sponsor will compare the severity of each event and the cumulative event frequency reported for the study with the severity and frequency reported in the applicable reference document.

Reporting requirements will also be based on the investigator's assessment of causality and seriousness, with allowance for upgrading by the Sponsor as needed.

6. STATISTICAL CONSIDERATIONS AND ANALYSIS PLAN

The primary and secondary efficacy analyses will be based on a modified intent-to-treat (mITT) approach. All patients who received at least one dose of study drug will be included in the mITT population, with patients grouped according to the treatment assigned at randomization. Safety analyses will be conducted on the safety-evaluable population, defined as all patients who received at least one dose of study drug, with patients grouped according to the actual treatment received.

The final analysis of data from the 8-week, placebo-controlled period will be performed when the following two criteria have been met: 1) All patients have either completed the Day 57 visit or discontinued from the placebo-controlled period prematurely and 2) all data from the placebo-controlled period are in the database and have been cleaned and verified. Patients and study site personnel will remain blinded to the individual treatment assignment until after the study is completed (i.e., after all patients have either completed the safety follow-up period or discontinued early from the study), the database is locked, and the study analyses are final.

The focus of the trial is estimation and generation of hypotheses to be confirmed in future trials; therefore, Type I error control is not addressed.

6.1 DETERMINATION OF SAMPLE SIZE

The purpose of this study is to evaluate the efficacy of GDC-0853 compared with placebo in improving the UAS7. Point and interval estimates of the change from baseline of the UAS7 within each treatment group as well as of the difference in change from baseline of the UAS7 between treatment groups will be presented.

The study will enroll approximately 45 patients. Patients will be randomized in a 2:1 ratio to receive treatment with either GDC-0853 or placebo. The sample size of approximately 30 patients in the GDC-0853 arm and 15 patients in the placebo arm provides approximately 80% power to detect an 11-point difference in the UAS7 change from baseline at Day 57 between treatment groups under the following assumptions:

- The absolute change from baseline at Day 57 is normally distributed with a standard deviation of 13.
- Two-sided alpha is 0.10.
- Dropout rate at Day 57 is 10%, leading to a 10% loss of information.

6.2 SUMMARIES OF CONDUCT OF STUDY

The number of patients who enroll, discontinue, or complete the study will be summarized. Reasons for premature study withdrawal will be listed and summarized. Enrollment and major protocol deviations will be listed and evaluated for their potential effects on the interpretation of study results.

6.3 SUMMARIES OF TREATMENT GROUP COMPARABILITY

Baseline demographics, disease characteristics, and exposure to study drug will be summarized by treatment group using descriptive statistics. For categorical endpoints, the descriptive statistics will include counts and proportions. For continuous endpoints, the descriptive statistics will include the number of observations, mean, standard deviation, median, minimum, and maximum.

6.4 EFFICACY ANALYSES

Statistical testing will be performed as a two-sided test with a statistical significance level of 0.10. No multiplicity adjustments will be performed to control overall Type I error, and positive tests will be viewed as hypothesis generating rather than confirmatory.

Continuous longitudinal efficacy endpoints will be analyzed using a mixed model for repeated measures (MMRM) and descriptive statistics as appropriate. An unstructured covariance pattern will be specified to model the within-subject errors. Parameters will be estimated with the use of restricted maximum likelihood, and the Kenward-Roger method will be used for calculating the denominator degrees of freedom. The MMRM method assumes that data are missing at random. That is, MMRM assumes that given the statistical model and given the observed values of the endpoint, missing data are independent of the unobserved values (O'Kelly and Ratitch 2014). High correlation between successive observations on a subject allows data from subjects who dropped out to make a contribution to estimation of the effects at the final timepoint.

All MMRM models will include country, treatment group, visit, and visit by treatment group interaction as covariates.

Time-to-event endpoints will be analyzed using a Cox proportional hazards model. Categorical endpoints will be analyzed using an appropriate statistical method, such as Cochran-Mantel-Haenszel test or Fisher's exact test.

Details of all statistical methods will be provided in the Data Analysis Plan.

6.4.1 Primary Efficacy Endpoint

The primary efficacy endpoint is the change from baseline in the UAS7 at Day 57 (Week 8).

The UAS is to be recorded twice daily (i.e., morning and evening) using an eDiary that will be provided to each patient. Scores ranging from 0 (none) to 3 (severe) will be entered for each of the two UAS domains consisting of number of wheals (hives) and intensity of pruritis (itch) resulting in a total possible score of 0 to 6 (see Table 2). The daily UAS is calculated as the average of the morning and evening scores. When either the morning or evening score is missing, the non-missing UAS for that day (morning or evening) will be used as the daily UAS, and when both the morning and evening UAS are missing, the daily UAS will be deemed missing. The UAS7 is the sum of the daily UAS over the 7 days prior to the timepoint of interest. The baseline UAS7 will be calculated as the sum of daily UAS values over the week (7 days) prior to Day 1.

When one or more daily UAS values is missing, over the week prior to a timepoint of interest, rules for deriving UAS7 will be as follows:

- If a patient has at least 4 completed daily scores on the UAS (both domains) over the 7 days prior to the timepoint of interest, the UAS7 will be defined as the average of the available daily scores, multiplied by 7.
- If a patient has fewer than 4 completed daily scores on the UAS over the 7 days prior to the timepoint of interest, then the UAS7 will be considered missing for that timepoint.

The primary endpoint will be analyzed using a MMRM model as specified in Section 6.4. Additional model covariates will include baseline UAS7 and its interaction with visit. Missing data will be handled by the model under the missing-at-random assumption without need for imputation.

As a sensitivity analysis, an analysis-of-covariance (ANCOVA) model adjusted for country and baseline UAS7 will be fit. Missing Day 57 data will be imputed by last observation carried forward.

6.4.2 Secondary Efficacy Endpoints

The secondary efficacy endpoints are as follows:

- Proportion of patients who are well controlled (UAS7 $\leq$6) at Day 57
- Change from baseline in the UAS7 at Day 29 (Week 4)

These endpoints will be analyzed as specified in Section 6.4.

6.4.3 Exploratory Efficacy Endpoints

The exploratory efficacy endpoints include the following:

- Change from baseline in the weekly itch score at Day 29
- Change from baseline in the weekly itch score at Day 57
- Change from baseline in the weekly hives score at Day 57
- Proportion of patients who are well controlled (UAS7 $\leq$6) at Day 29

- Proportion of patients who achieve complete response (UAS7 = 0) at Day 29
- Proportion of patients who achieve complete response (UAS7 = 0) at Day 57
- Proportion of patients achieving MID in UAS7 at Day 57 (reduction from baseline ≥ 11 points)
- Proportion of patients achieving MID in the weekly itch score at Day 57 (reduction from baseline ≥ 5 points)
- Time to achieving MID in UAS7 (reduction from baseline ≥ 11 points)
- [redacted]
- [redacted]
- [redacted]
- [redacted]

Further details on the analysis of exploratory endpoints will be included in the Data Analysis Plan.

6.5 SAFETY ANALYSES

Adverse events will be graded according to the adverse event severity grading scale described in Section 5.3.3. Summaries of adverse events, serious adverse events, deaths, adverse events of special interest, adverse events that lead to discontinuation, ECG findings, laboratory test results, and vital sign measurements will be presented.

6.6 PHARMACOKINETIC ANALYSES

The PK endpoints are as follows:

- Steady-state PK parameters (i.e., area under the concentration–time curve from time 0 to time t [$AUC_{0\text{-}t}$], C_{max}, time to maximum concentration [t_{max}], steady-state concentration at the end of a dosing interval [C_{trough}], half-life [$t_{1/2}$], apparent clearance [CL/F])

[redacted]

- [redacted]
- [redacted]
- [redacted]
- [redacted]
- [redacted]
- [redacted]

The PK analyses will include patients with sufficient data to enable estimation of key parameters (e.g., AUC, t_{max}, C_{max}, $t_{1/2}$), with patients grouped according to treatment received.

Individual and mean plasma GDC-0853 concentration versus time data will be tabulated and plotted. The plasma pharmacokinetics of GDC-0853 will be summarized by estimating total exposure (AUC), C_{max}, total clearance, volume of distribution at steady state, and terminal half-life (as appropriate for data collected). Estimates for these parameters will be tabulated and summarized (mean, standard deviation, coefficient of variation, median, minimum, and maximum). Inter-patient variability will be evaluated.

Additional PK analyses will be conducted as appropriate.

[redacted]

[redacted]

- [redacted]
- [redacted]

[redacted]

7. DATA COLLECTION AND MANAGEMENT

7.1 DATA QUALITY ASSURANCE

The Sponsor will be responsible for data management of this study, including quality checking of the data. Data entered manually will be collected via EDC through use of eCRFs. Sites will be responsible for data entry into the EDC system. In the event of discrepant data, the Sponsor will request data clarification from the sites, which the sites will resolve electronically in the EDC system.

The Sponsor will produce an EDC Study Specification document that describes the quality checking to be performed on the data. Central laboratory data will be sent directly to the Sponsor, using the Sponsor's standard procedures to handle and process the electronic transfer of these data.

eCRFs and correction documentation will be maintained in the EDC system's audit trail. System backups for data stored by the Sponsor and records retention for the study data will be consistent with the Sponsor's standard procedures.

PRO data will be collected through the use of an electronic device provided by a vendor. The device is designed for entry of data in a way that is attributable, secure, and accurate, in compliance with U.S. Food and Drug Administration (FDA) regulations for electronic records (21 CFR Part 11). The electronic data are available for view access only via secure access to a web server method. Only identified and trained users may view the data, and their actions become part of the audit trail. The Sponsor will have view access only. System backups for data stored by the Sponsor and records retention for the study data will be consistent with the Sponsor's standard procedures.

7.2 ELECTRONIC CASE REPORT FORMS

eCRFs are to be completed through use of a Sponsor-designated EDC system. Sites will receive training and have access to a manual for appropriate eCRF completion. eCRFs will be submitted electronically to the Sponsor and should be handled in accordance with instructions from the Sponsor.

All eCRFs should be completed by designated, trained site staff. eCRFs should be reviewed and electronically signed and dated by the investigator or a designee.

At the end of the study, the investigator will receive patient data for his or her site in a readable format on a compact disc that must be kept with the study records. Acknowledgement of receipt of the compact disc is required.

7.3 ELECTRONIC PATIENT-REPORTED OUTCOME DATA

Patients will use an electronic device to capture PRO data. The data will be transmitted via wireless or web automatically after entry or uploaded by site staff at the appropriate frequency to a centralized database maintained by the electronic device vendor.

Once the study is complete, the data, audit trail, and trial and system documentation will be archived. The investigator will receive patient data for the site in both human- and machine-readable formats on an archival-quality compact disc that must be kept with the study records as source data. Acknowledgement of receipt of the compact disc is required. In addition, the Sponsor will receive all data in a machine-readable format on a compact disc.

7.4 SOURCE DATA DOCUMENTATION

Study monitors will perform ongoing source data verification to confirm that critical protocol data (i.e., source data) entered into the eCRFs by authorized site personnel are accurate, complete, and verifiable from source documents.

Source documents (paper or electronic) are those in which patient data are recorded and documented for the first time. They include, but are not limited to, hospital records, clinical and office charts, laboratory notes, memoranda, patient-reported outcomes, evaluation checklists, pharmacy dispensing records, recorded data from automated

instruments, copies of transcriptions that are certified after verification as being accurate and complete, microfiche, photographic negatives, microfilm or magnetic media, X-rays, patient files, and records kept at pharmacies, laboratories, and medico-technical departments involved in a clinical trial.

Before study initiation, the types of source documents that are to be generated will be clearly defined in the Trial Monitoring Plan. This includes any protocol data to be entered directly into the eCRFs (i.e., no prior written or electronic record of the data) and considered source data.

Source documents that are required to verify the validity and completeness of data entered into the eCRFs must not be obliterated or destroyed and must be retained per the policy for retention of records described in Section 7.6.

To facilitate source data verification, the investigators and institutions must provide the Sponsor direct access to applicable source documents and reports for trial-related monitoring, Sponsor audits, and IRB/EC review. The study site must also allow inspection by applicable health authorities.

7.5 USE OF COMPUTERIZED SYSTEMS

When clinical observations are entered directly into a study site's computerized medical record system (i.e., in lieu of original hardcopy records), the electronic record can serve as the source document if the system has been validated in accordance with health authority requirements pertaining to computerized systems used in clinical research. An acceptable computerized data collection system allows preservation of the original entry of data. If original data are modified, the system should maintain a viewable audit trail that shows the original data as well as the reason for the change, name of the person making the change, and date of the change.

7.6 RETENTION OF RECORDS

Records and documents pertaining to the conduct of this study and the distribution of IMP, including eCRFs, electronic PRO data (if applicable), Informed Consent Forms, laboratory test results, and medication inventory records, must be retained by the Principal Investigator for at least 15 years after completion or discontinuation of the study or for the length of time required by relevant national or local health authorities, whichever is longer. After that period of time, the documents may be destroyed, subject to local regulations.

No records may be disposed of without the written approval of the Sponsor. Written notification should be provided to the Sponsor prior to transferring any records to another party or moving them to another location.

8. ETHICAL CONSIDERATIONS

8.1 COMPLIANCE WITH LAWS AND REGULATIONS

This study will be conducted in full conformance with the ICH E6 guideline for Good Clinical Practice and the principles of the Declaration of Helsinki, or the laws and regulations of the country in which the research is conducted, whichever affords the greater protection to the individual. The study will comply with the requirements of the ICH E2A guideline (Clinical Safety Data Management: Definitions and Standards for Expedited Reporting). Studies conducted in the United States or under a U.S. Investigational New Drug (IND) application will comply with U.S. FDA regulations and applicable local, state, and federal laws. Studies conducted in the European Union or European Economic Area will comply with the E.U. Clinical Trial Directive (2001/20/EC).

8.2 INFORMED CONSENT

The Sponsor’s sample Informed Consent Form (and ancillary sample Informed Consent Forms, if applicable) will be provided to each site. If applicable, it will be provided in a certified translation of the local language. The Sponsor or its designee must review and approve any proposed deviations from the Sponsor's sample Informed Consent Forms or any alternate consent forms proposed by the site (collectively, the "Consent Forms") before IRB/EC submission. The final IRB/EC−approved Consent Forms must be provided to the Sponsor for health authority submission purposes according to local requirements.

If applicable, the Informed Consent Form will contain separate sections for any optional procedures. The investigator or authorized designee will explain to each patient the objectives, methods, and potential risks associated with each optional procedure. Patients will be told that they are free to refuse to participate and may withdraw their consent at any time for any reason. A separate, specific signature will be required to document a patient's agreement to participate in optional procedures. Patients who decline to participate will not provide a separate signature.

The Consent Forms must be signed and dated by the patient or the patient’s legally authorized representative before his or her participation in the study. The case history or clinical records for each patient shall document the informed consent process and that written informed consent was obtained prior to participation in the study.

The Consent Forms should be revised whenever there are changes to study procedures or when new information becomes available that may affect the willingness of the patient to participate. The final revised IRB/EC-approved Consent Forms must be provided to the Sponsor for health authority submission purposes.

Patients must be re-consented to the most current version of the Consent Forms (or to a significant new information/findings addendum in accordance with applicable laws and

IRB/EC policy) during their participation in the study. For any updated or revised Consent Forms, the case history or clinical records for each patient shall document the informed consent process and that written informed consent was obtained using the updated/revised Consent Forms for continued participation in the study.

A copy of each signed Consent Form must be provided to the patient or the patient's legally authorized representative. All signed and dated Consent Forms must remain in each patient's study file or in the site file and must be available for verification by study monitors at any time.

8.3 INSTITUTIONAL REVIEW BOARD OR ETHICS COMMITTEE

This protocol, the Informed Consent Forms, any information to be given to the patient, and relevant supporting information must be submitted to the IRB/EC by the Principal Investigator and reviewed and approved by the IRB/EC before the study is initiated. In addition, any patient recruitment materials must be approved by the IRB/EC.

The Principal Investigator is responsible for providing written summaries of the status of the study to the IRB/EC annually or more frequently in accordance with the requirements, policies, and procedures established by the IRB/EC. Investigators are also responsible for promptly informing the IRB/EC of any protocol amendments (see Section 9.6).

In addition to the requirements for reporting all adverse events to the Sponsor, investigators must comply with requirements for reporting serious adverse events to the local health authority and IRB/EC. Investigators may receive written IND safety reports or other safety-related communications from the Sponsor. Investigators are responsible for ensuring that such reports are reviewed and processed in accordance with health authority requirements and the policies and procedures established by their IRB/EC, and archived in the site's study file.

8.4 CONFIDENTIALITY

The Sponsor maintains confidentiality standards by coding each patient enrolled in the study through assignment of a unique patient identification number. This means that patient names are not included in data sets that are transmitted to any Sponsor location.

Patient medical information obtained by this study is confidential and may be disclosed to third parties only as permitted by the Informed Consent Form (or separate authorization for use and disclosure of personal health information) signed by the patient, unless permitted or required by law.

Medical information may be given to a patient's personal physician or other appropriate medical personnel responsible for the patient's welfare, for treatment purposes.

Given the complexity and exploratory nature of the analyses, data derived from exploratory biomarker specimens will generally not be provided to study investigators or patients unless required by law. The aggregate results of any conducted research will be available in accordance with the effective Sponsor policy on study data publication (see Section 9.5).

Data generated by this study must be available for inspection upon request by representatives of national and local health authorities, Sponsor monitors, representatives, and collaborators, and the IRB/EC for each study site, as appropriate.

8.5 FINANCIAL DISCLOSURE

Investigators will provide the Sponsor with sufficient, accurate financial information in accordance with local regulations to allow the Sponsor to submit complete and accurate financial certification or disclosure statements to the appropriate health authorities. Investigators are responsible for providing information on financial interests during the course of the study and for 1 year after completion of the study (i.e., last patient, last visit).

9. STUDY DOCUMENTATION, MONITORING, AND ADMINISTRATION

9.1 STUDY DOCUMENTATION

The investigator must maintain adequate and accurate records to enable the conduct of the study to be fully documented, including, but not limited to, the protocol, protocol amendments, Informed Consent Forms, and documentation of IRB/EC and governmental approval. In addition, at the end of the study, the investigator will receive the patient data, including an audit trail containing a complete record of all changes to data.

9.2 PROTOCOL DEVIATIONS

The investigator should document and explain any protocol deviations. The investigator should promptly report any deviations that might have an impact on patient safety and data integrity to the Sponsor and to the IRB/EC in accordance with established IRB/EC policies and procedures.

9.3 SITE INSPECTIONS

Site visits will be conducted by the Sponsor or an authorized representative for inspection of study data, subjects' medical records, and eCRFs. The investigator will permit national and local health authorities; Sponsor monitors, representatives, and collaborators; and the IRBs/ECs to inspect facilities and records relevant to this study.

9.4 ADMINISTRATIVE STRUCTURE

This trial is sponsored by Genentech. This pilot study will be conducted at study sites experienced in conducting clinical trials in CSU and will enroll approximately 45 patients. Data will be recorded via an EDC system from Medidata Solutions (New York, NY) using eCRFs (see Section 7.2). The contract research organization will be responsible for submission to IRB/ECs for approval of the study protocol, patient recruitment, data collection, and reporting. An IxRS will be used to assign patients to treatment groups and to manage ongoing investigational product requests and shipments.

9.5 PUBLICATION OF DATA AND PROTECTION OF TRADE SECRETS

Regardless of the outcome of a trial, the Sponsor is dedicated to openly providing information on the trial to healthcare professionals and to the public, both at scientific congresses and in peer-reviewed journals. The Sponsor will comply with all requirements for publication of study results. For more information, refer to the Roche Global Policy on Sharing of Clinical Trials Data at the following Web site:

www.roche.com/roche_global_policy_on_sharing_of_clinical_study_information.pdf

The results of this study may be published or presented at scientific congresses. For all clinical trials in patients involving an IMP for which a marketing authorization application has been filed or approved in any country, the Sponsor aims to submit a journal manuscript reporting primary clinical trial results within 6 months after the availability of the respective Clinical Study Report. In addition, for all clinical trials in patients involving an IMP for which a marketing authorization application has been filed or approved in any country, the Sponsor aims to publish results from analyses of additional endpoints and exploratory data that are clinically meaningful and statistically sound.

The investigator must agree to submit all manuscripts or abstracts to the Sponsor prior to submission for publication or presentation. This allows the Sponsor to protect proprietary information and to provide comments based on information from other studies that may not yet be available to the investigator.

In accordance with standard editorial and ethical practice, the Sponsor will generally support publication of multicenter trials only in their entirety and not as individual center data. In this case, a coordinating investigator will be designated by mutual agreement.

Authorship will be determined by mutual agreement and in line with International Committee of Medical Journal Editors authorship requirements. Any formal publication of the study in which contribution of Sponsor personnel exceeded that of conventional monitoring will be considered as a joint publication by the investigator and the appropriate Sponsor personnel.

Any inventions and resulting patents, improvements, and/or know-how originating from the use of data from this study will become and remain the exclusive and unburdened property of the Sponsor, except where agreed otherwise.

9.6 PROTOCOL AMENDMENTS

Any protocol amendments will be prepared by the Sponsor. Protocol amendments will be submitted to the IRB/EC and to regulatory authorities in accordance with local regulatory requirements.

Approval must be obtained from the IRB/EC and regulatory authorities (as locally required) before implementation of any changes, except for changes necessary to eliminate an immediate hazard to patients or changes that involve logistical or administrative aspects only (e.g., change in Medical Monitor or contact information).

10. REFERENCES

Altrichter S, Peter HJ, Pisarevskaja D, et al. IgE mediated autoallergy against thyroid peroxidase–a novel pathomechanism of chronic spontaneous urticaria? PLoS ONE 2011;6:e14794.

Broides A, Yang W, Conley ME. Genotype/phenotype correlations in X-linked agammaglobulinemia. Clin Immunol 2006;118:195–200.

Bruton OC. Agammaglobulinemia. Pediatrics 1952;9:722–8.

Bugatti S, Vitolo B, Caporali R, et al. B cells in rheumatoid arthritis: from pathogenic players to disease biomarkers. Biomed Res Int 2014;2014:681678. doi: 10.1155/2014/681678

Conley ME, Broides A, Hernandez-Trujillo V, et al. Genetic anaylis of patients with defects in early B-cell development. Immunol Rev 2005;203:216–34.

Di Paolo JA, Huang T, Balazs M, et al. Specific BTK Inhibition suppresses B cell and myeloid cell-mediated arthritis. Nat Chem Biol 2011;7:41–50.

[FDA] U.S. Food and Drug Administration. Pharmacology review of Incivek (telaprevir). 2011. Available from: http://www.accessdata.fda.gov/drugsatfda_docs/nda/2011/201917Orig1s000TOC.cfm

Ferrer M. Immunological events in chronic spontaneous urticaria. Clin Transl Allergy 2015;5:360. doi: 10.1186/s13601-015-0074-7

Fiebiger E, Maurer D, Holub H, et al. Serum IgG autoantibodies directed against the α chain of FcεRI: a selective marker and pathogenetic factor for a distinct subset of chronic urticaria patients? J Clin Invest 1995;96:2606–12. doi: 10.1172/JCI118325

Greaves M. Chronic urticaria. J Allergy Clin Immunol 2000;105:664–72.

Greaves M. Chronic idiopathic urticaria. Curr Opin Allergy Clin Immunol 2003;3:363–8.

Hata D, Kawakami Y, Inagaki N, et al. Involvement of Bruton's tyrosine kinase in FcεRI-dependent mast cell degranulation and cytokine production. J Exp Med. 1998 Apr 20;187:1235–47.

Inman WH, Vessey MP, Westerholm B, et al. Thromboembolic disease and the steroidal content of oral contraceptives. A report to the Committee on Safety of Drugs. Br Med J 1970;2:203–9.

Iyer AS, Morales JL, Huang W, et al. Absence of Tec family kinases interleukin-2 inducible T cell kinase (Itk) and Bruton's tyrosine kinase (Btk) severely impairs FcεRI-dependent mast cell responses. J Bio Chem 2011;286:9503–13. doi: 10.1074/jbc.M110.1656131

Kaplan AP. Chronic urticaria and angioedema. N Engl J Med 2002;346:175–9.

Kaveri SV, Maddur MS, Hegde P, et al. Intravenous immunoglobulins in immunodeficiences: more that mere replacement therapy. Clin Exp Immunol 2011;164(Suppl 2):2–5. doi: 10.1111/j.1365-2249.2011.04387.x

Kay AB, Ying S, Ardelean E, et al. Elevations in vascular markers and eosinophils in chronic spontaneous urticarial weals with low-level persistence in uninvolved skin. Br J Dermatol 2014;171:505–11. doi: 10.1111/bjd.12991

Kern F, Lichtenstein LM. Defective histamine release in chronic urticaria. J Clin Invest 1976;57:1369–77.

Kozel MA, Sabroe RA. Chronic urticaria, aetiology, management and current and future treatment options. Drugs 2004;64:2515–36.

[redacted]

Lederman HM, Winkelstein JA. X-linked agammaglobulinema: an analysis of 96 patients. Medicine (Baltimore) 1985;64:145–56.

Liu L, Di Paolo J, Barbosa J, et al. Antiarthritis effect of a novel Bruton's tyrosine kinase (BTK) inhibitor in rat collagen-induced arthritis and mechanism-based pharmacokinetic/pharmacodynamic modeling: relationships between inhibition of BTK phosphorylation and efficacy. J Pharmacol Exp Ther 2011;338:154–63.

Luquin E, Kaplan AP, Ferrer M. Increased responsiveness of basophils of patients with chronic urticaria to sera but hypo-responsiveness to other stimuli. Clin Exp Allergy 2005;35:456–60. doi: 10.1111/j.1365-2222.2005.02212.x

Mathias SD, Crosby RD, Zazzali JL, et al. Evaluating the minimally important difference of the urticaria activity score and other measures of disease activity in patients with chronic idiopathic urticaria. Ann Allergy Asthma Immunol 2012;108:20–4. doi: 10.1016/j.anai.2011.09.008

Maurer M, Weller K, Bindslev-Jensen C, et al. Unmet clinical needs in chronic spontaneous urticaria. A GA2LEN task force report. Allergy 2011;66:317–30.

McGirt LY, Vasagar K, Gober LM, et al. Successful treatment of recalcitrant chronic idiopathic urticaria with sulfasalazine. Arch Dermatol 2006;142:1337–42.

Medicinal Products Act in the version published on 12 December 2005 (Federal Law Gazette [BGBl.]) Part I p. 3394, last amended by Article 2a of the Law of 27 March 2014 (Federal Law Gazette I p. 261).

Misbah SA, Spickett GP, Ryba PC, et al. Chronic enteroviral meningoencephalitis in agammaglobulinemia: case report and literature review. J Clin Immunol 1992;12:266–70.

Niiro H, Clark EA. Regulation of B-cell fate by antigen-receptor signals. Nat Rev Immunol 2002;2:945–56.

O'Kelly M, Ratitch B. Clinical trials with missing data: a guide for practitioners. Chichester, UK: John Wiley & Sons, 2014.

Powell RJ, Leech SC, Till S, et al. BSACI guideline for the management of chronic urticaria and angioedema. Clin Exp Allergy 2015;45:547–65. doi: 10.1111/cea.12494

Puri K, Di Paolo J, Gold M. B-cell receptor signaling inhibitors for treatment of autoimmune inflammatory diseases and B-cell malignancies. Int Rev Immunol 2013;32:397–427.

Reth M, Nielsen P. Signaling circuits in early B-cell development. Adv Immunol 2014;122:129–75. doi: 10.1016/B978-0-12-800267-4.00004-3

Saini SS. Chronic spontaneuos urticaria: etiology and pathogensis. Immunol Allergy Clin North Am 2014;34:33–52. doi: 10.1016/j.iac.2013.09.012

Sochorová K, Horvath R, Rozkova D, et al. Impaired toll-like receptor 8-mediated IL-6 and TNF-α production in antigen-presenting cells from patients with X-linked agammaglobulinemia. Blood 2007;109:2553–6.

Snyder PW, Kazacos EA, Scott-Moncrieff JC, et al. Pathologic features of naturally occurring juvenile polyarteritis in beagle dogs. Vet Pathol 1995;32:337–45.

Tilles SA. Approach to therapy in chronic urticaria: when Benadryl is not enough. Allergy Asthma Proc 2005;26:9–12.

Tong LJ, Balakrishnan G, Kochan J. et al. Assessment of autoimmunity in patients with chronic urticarial. J Allergy Clin Immunol 1997;99:461–5.

Tsukada S, Saffran DC, Rawlings DJ, et al. Deficient expression of a B cell cytoplasmic tyrosine kinase in human X-linked agammaglobulinemia. Cell 1993;72:279–90. doi:10.1016/0092-8674(93)90667-F

van den Bruele T, Mourad-Baars PE, Claas EC, et al. Campylobacter jejuni bacteremia and Helicobacter pylori in a patient with X-linked agammaglobulinemia. Eur J Clin Microbiol Infect Dis 2010;29:1315–9.

Vetrie D, Vořechovský I, Sideras P, et al. The gene involved in X-linked agammaglobulinaemia is a member of the src family of protein-tyrosine kinases. Nature 1993;361:226–33. doi:10.1038/361226a0

Whang JA, Chang BY. Bruton's tyrosine kinase inhibitors for the treatment of rheumatoid arthritis. Drug Discov Today 2014;19:1200–4. doi: 10.1016/j.drudis.2014.03.028

Winkelstein JA, Marino MC, Lederman HM, et al. X-linked agammaglobulinema: report on a United States registry of 201 patients. Medicine (Baltimore) 2006;85:193–202.

Ying S, Kikuchi Y, Meng Q, et al. TH1/TH2 cytokines and inflammatory cells in skin biopsy specimens from patients with chronic idiopathic urticaria: comparison with the allergen-induced late-phase cutaneous reaction. J Allergy Clin Immunol 2002;109:694–700.

Zhang H, Cui D, Wang B, et al. Pharmacokinetic drug interactions involving 17alpha-ethinylestradiol: a new look at an old drug. Clin Pharmacokinet 2007;46:133–57.

Ziegner UH, Kobayashi RH, Cunningham-Rundles C, et al. Progressive neurodegeneration in patients with primary immunodeficiency disease on IVIG treatment. Clin Immunol 2002;102:19–24.

Zweiman B, Valenzano M, Atkins PC. Modulation of serum histamine releasing activity in chronic idiopathic urticaria. Immunopharmacology 1998;39:225–34.

Appendix 1
Schedule of Activities

	Screening		Treatment Period						Safety Follow Up	Early Term.	Unscheduled Visit
Week	Screening		0	1	2	4	6 [a]	8	12		
Day (± days)	−14 (−4/+2)	−7	1 [b]	8 (±1)	15 (±1)	29 [b] (±2)	43 (±2)	57 [b,c] (±2)	85 (±2)		
Informed consent	x										
Demographic data	x										
General medical history and baseline conditions	x										
Inclusion/exclusion criteria	x	x	x								
Randomization			x								
Train patient to complete Urticaria Patient Daily eDiary (including UAS) [d]	x	x									
Distribute eDiary to patient [e]	x										
Urticaria Patient Daily eDiary [e]		x	x	x	x	x	x	x	x	x	
[redacted]	[redacted]					x		x	x		
Concomitant medications [g]	x	x	x	x	x	x	x	x	x	x	x
Adverse events			x	x	x	x	x	x	x	x	x
Vital signs [h]	x	x	x	x	x	x		x	x	x	x [i]
Height	x										
Weight	x		x			x		x	x	x	x [i]
Complete physical examination [j]	x								x		
Limited physical examination [k]		x	x	x	x	x		x		x	x [i]
ECG [l]	x		x	x					x	x	x [i]
Hepatitis Screening [m]	x										
QFT (PPD if QFT not available)	x										
Chest X-ray [n]	x										

Appendix 1
Schedule of Activities (cont.)

	Screening		Treatment Period						Safety Follow Up		
Week			0	1	2	4	6 [a]	8	12		
Day (± days)	–14 (–4/+2)	–7	1 [b]	8 (±1)	15 (±1)	29 [b] (±2)	43 (±2)	57 [b,c] (±2)	85 (±2)	Early Term.	Unscheduled Visit
GDC-0853/placebo administration in clinic [o, p]			x	x	x	x					
Drug Dispensing			x			x					
Hematology [q]	x	x	x	x	x	x		x	x	x	x [i]
Chemistry [r]	x	x	x	x	x	x		x	x	x	x [i]
Fasting Lipid Panel			x			x		x	x		
Coagulation studies [s]	x									x	x [i]
Pregnancy test [t]	x		x	x	x	x		x	x	x	
Urinalysis [u]	x		x					x	x	x	
[redacted]	x								x	x	
[redacted]	x								x	x	
FricTest (for patients with dermographism only)	x		x					x	x	x	
Plasma PK assessment [w]			x	x				x		x	x [i]
[redacted]	x		x					x	x	x	
[redacted]	x		x					x	x	x	
[redacted]			x					x			
Phone call [y]							x				

BID = twice a day; eCRF = electronic case report form; eDiary = electronic diary (patient reported outcomes); HBcAb = hepatitis B core antibody; HBsAb = hepatitis B surface antibody; HBsAg = hepatitis B surface antigen; HCV Ab = hepatitis C antibody; PD = pharmacodynamic; PK = pharmacokinetic; PPD = purified protein derivativeDerivative; PPI = proton pump inhibitor; QFT = QuantiFERON-TB Gold®; QTcF = QT interval corrected using Fridericia's formula; TB = tuberculosis [redacted]; Term. = Termination; UAS = Urticaria Activity Score; UAS7 = Urticaria Activity Score over 7 days; [redacted]

Appendix 1
Schedule of Activities (cont.)

[a] Phone call instead of a clinic visit.

[b] Morning clinic visit is required for visits on Days 1, 29, and 57; for other study visits, morning visits are recommended. For mandatory morning visits, the patient should be fasting (overnight, >8 hours) prior to the first PK blood draw and fasting lipid panel.

[c] Day 57 is the last day of the study treatment period; however, no study drug will be taken at the visit Day 57. The last dose of blinded study drug will be the p.m. dose on Day 56 or the day before the Day 57 visit if it does not occur on Day 57.

[d] Patients should be trained to use the eDiary at Day −14. At Day −7, staff should query patients for any questions they may have concerning the use of the eDiary and ensure patients understand correct usage before randomization.

[e] Patient is to complete the eDiary twice daily, approximately every 12 hours (a.m./p.m.), every day for the duration of the study. The eDiary includes the UAS7 (itch score, number of hives) and other patient reported outcomes.

[redacted]

[g] Includes any medication (e.g., prescription drugs, over-the-counter drugs, vaccines, herbal or homeopathic remedies, vitamins, and nutritional or dietary supplements) used by a patient from 12 weeks prior to initiation of study drug until 4 weeks after the last dose of study drug. In addition, at each clinic visit, any use of PPIs, H2 receptor antagonists, and/or other antacids (e.g., Maalox®, Pepto-Bismol®, Rolaids®) should be recorded as concomitant medications, including the date, dose, and time of last administration.

[h] Includes respiratory rate, pulse rate, temperature, and systolic and diastolic blood pressures while the patient is in a seated position for at least 5 minutes.

[i] This procedure is optional per the investigator's discretion.

[j] A complete physical examination should include an evaluation of the head, eyes, ears, nose, and throat and the cardiovascular, dermatological, musculoskeletal, respiratory, gastrointestinal, and neurological systems. Patients should be screened for dermographism. Any abnormality identified at baseline should be recorded on the General Medical History and Baseline Condition eCRF.

[k] Perform a limited, symptom-directed examination at specified timepoints or as clinically indicated. Record new or worsened clinically significant abnormalities on the Adverse Event eCRF.

[l] Interpretable digital ECG recording (e.g., without artifacts) will be obtained. The ECG intervals (e.g., PR, QRS, QT, QTcF, and RR) and heart rate from the ECGs will be entered into the eCRF; ECGs for each patient should be obtained from the same machine whenever possible. ECGs can be performed without specific restrictions (e.g., can be any time of day, before or after dosing, fasting or fed) but are to be obtained prior to other procedures scheduled at that same time (e.g., vital sign measurements, blood draws). ECGs must be performed after the patient has been resting in a supine position for at least 10 minutes prior to beginning the ECG recording. Environmental distractions (e.g., television, radio, conversation) should be avoided during the pre-ECG resting period and during ECG recording.

[m] HBsAg, HBsAb, HBcAb, and HCV Ab.

[n] Performed only if required by local guidelines to rule out active TB infection.

[o] On mandatory morning clinic visit days (Days 1 and 29), patients should be instructed that the morning dose of study drug will be taken in the clinic. On other clinic visit days, if the visit occurs in the morning, the patient should be instructed that the morning dose of study drug will be taken in the clinic. The morning dose should be taken after all pre-dose assessments are complete (i.e., ECG, questionnaires, and PK and biomarker sample collection).

[p] Patients will take GDC-0853/placebo BID approximately every 12 hours starting on Day 1 and ending on Day 56 (pm) or the day before the Day 57 visit if it does not occur on Day 57. One dose (a total of 4 tablets) of GDC-0853/placebo should be taken with water by mouth BID (a total of 8 tablets each day). The dates and times of the most recent prior meal, last dose of oral study drug (prior to clinic visit), and timing of study drug administration in clinic should be recorded at each clinic visit. The last dose of blinded study drug is the p.m. dose on Day 56 (i.e., day before the Day 57 visit) for all patients.

[q] Includes WBC count, RBC count, hemoglobin, hematocrit, platelet count, and WBC differential (i.e., neutrophils, eosinophils, basophils, monocytes, lymphocytes, and other cells if present).

[r] Includes sodium, potassium, chloride, bicarbonate, glucose, BUN or urea, creatinine, total protein, albumin, phosphorus, calcium, total and direct bilirubin, alkaline phosphatase, ALT, AST, uric acid, LDH, and lipase.

[s] Includes PT, PTT, INR, and fibrinogen.

[t] All women of childbearing potential, including those who have had a tubal ligation, will have a serum pregnancy test at screening. Urine pregnancy tests will be performed locally at specified subsequent visits. If a urine pregnancy test result is positive, it must be confirmed by a serum pregnancy test (performed locally).

[u] Includes dipstick, including pH, specific gravity, glucose, protein, ketones, blood, and microscopic examination (e.g., sediment, RBCs, WBCs, casts, crystals, epithelial cells, bacteria if present).

[redacted]

[w] Collect PK samples prior to drug administration, and the patient should be fasting overnight for $>$8 hours.

[redacted]

[y] Site staff to remind patients to take the study drug and to complete their eDiaries.

Appendix 2
Childbearing Potential, Pregnancy Testing, and Contraception

For Women

All women of childbearing potential (including those who have had a tubal ligation) will have a serum pregnancy test at screening and a urine pregnancy test on Study Day 1 prior to administration of study drug and monthly at appropriate clinic visits. If a urine pregnancy test result is positive, study drug will not be administered until pregnancy is ruled out. The result must be confirmed by a serum pregnancy test (conducted by the local laboratory). Refer to Section 5.4.3 of the protocol for management of a patient with a confirmed pregnancy.

All female patients are considered to be of childbearing potential unless they meet one of the following criteria:

- The patient has been postmenopausal (non−therapy-induced amenorrhea) for at least 12 continuous months with no other identified cause.
- The patient had a surgical bilateral oophorectomy (with or without hysterectomy) more than 6 weeks prior to enrollment.
- The patient had a hysterectomy.

Female patients of reproductive or childbearing potential who are unwilling to use a method of contraception that results in a failure rate of <1 % per year or remain abstinent (refrain from heterosexual intercourse) during the treatment period and for at least 4 weeks after the last dose of study drug will be excluded from study participation.

Abstinence is acceptable only if it is in line with the preferred and usual lifestyle of the patient. Periodic abstinence (e.g., calendar, ovulation, symptothermal, or post-ovulation methods) and withdrawal are not acceptable methods of contraception.

Examples of contraceptive methods with a failure rate of <1% per year include the following:

- Sterilization, bilateral surgical tubal ligation
- Intrauterine device
- Combined oral contraceptive pill [1]
- Contraceptive transdermal patch (estrogen and progestin containing)[1]
- Hormonal vaginal device
- Progestogen-only hormonal contraception associated with inhibition of ovulation
- Implants for contraception
- Injections for contraception (with prolonged release)
- Sole sexual partner consisting of surgically sterilized male partner with appropriate postsurgical verification of the absence of spermatozoa in the ejaculate. Patients

may provide verbal confirmation that the partner completed appropriate follow-up after vasectomy. Sites are not required to obtain partner medical records.

[1] Women using estrogen-containing hormonal contraceptives as a method of contraception must also use a barrier such as a male condom in conjunction with the hormonal contraceptives.

For Men:

All men must agree to remain abstinent (refrain from heterosexual intercourse) or use contraceptive measures, and agreement to refrain from donating sperm, as defined below:

- With female partners of childbearing potential or pregnant female partners, men must remain abstinent or use a condom during the treatment period and for at least 4 weeks after the last dose of GDC-0853 to avoid exposing the embryo. Men must refrain from donating sperm during this same period.

For Men and Women

The reliability of sexual abstinence should be evaluated in relation to the duration of the clinical trial and the preferred and usual lifestyle of the patient. Periodic abstinence (e.g., calendar, ovulation, symptothermal, and post-ovulation methods) and withdrawal are not acceptable methods of contraception.

Appendix 3
Urticaria Patient Daily eDiary

General Instructions

Please answer each question to the best of your ability.

There are no right or wrong answers.

For each question, please choose the response that describes your experience.

Please pay close attention to the timeframe of interest. Some questions ask about the **past 12 hours**, while others ask about the **past 24 hours**.

Instructions for Counting the Number of Hives and Measuring the Size of the Largest Hive

Count each hive separately even if you have more than one hive grouped together with other hives.

Please use the ruler that you have been given to measure the size of your largest hive. If you need help, please have someone else take this measurement for you. **Please do not measure a group of hives as one hive.**

Appendix 3 Urticaria Patient Daily eDiary (cont.)

Today's Date

		-				-				

Day **Month** **Year**

Please complete this section <u>every morning</u> throughout the duration of the study. (Please circle only one response.)

1. Thinking about the **<u>past 12 hours</u>**, please record the severity of itch and the number of hives you may have had associated with your skin condition. **<u>Please count each hive separately</u>** even if you have more than one hive grouped together with other hives.

Itch (severity)	Hives (number)
0 = none<br>1 = mild<br>2 = moderate<br>3 = severe	0 = none<br>1 = between 1 and 6 hives<br>2 = between 7 and 12 hives<br>3 = greater than 12 hives

This next question asks you to estimate the size of your largest hive in centimeters (cm). Please use the ruler that you have been provided with to make this measurement. If your largest hive is located on your back or in a place that is hard to reach, please have someone else take this measurement for you. When measuring the largest hive size, **<u>please do not measure a group of hives as one hive.</u>**

Largest Hive (size)
0 = none<br>1 = less than 1.25 centimeter (cm)<br>2 = between 1.25 centimeter (cm) and 2.5 centimeters (cm)<br>3 = greater than 2.5 centimeters (cm)

Appendix 3 Urticaria Patient Daily eDiary (cont.)

Today's Date

		-				-				

Day **Month** **Year**

Please complete this section <u>every evening</u> throughout the duration of the study. (Please circle only one response.)

2. Thinking about the **<u>past 12 hours</u>**, please record the severity of itch and the number of hives you may have had associated with your skin condition. **<u>Please count each hive separately</u>** even if you have more one than one hive grouped together with other hives.

Itch (severity)	Hives (number)
0 = none<br>1 = mild<br>2 = moderate<br>3 = severe	0 = none<br>1 = between 1 and 6 hives<br>2 = between 7 and 12 hives<br>3 = greater than 12 hives

This next question asks you to estimate the size of your largest hive in centimeters (cm). Please use the ruler that you have been provided with to make this measurement. If your largest hive is located on your back or in a place that is hard to reach, please have someone else take this measurement for you. When measuring the largest hive size, **<u>please do not measure a group of hives as one hive.</u>**

Largest Hive (size)
0 = none<br>1 = less than 1.25 centimeter (cm)<br>2 = between 1.25 centimeter (cm) and 2.5 centimeters (cm)<br>3 = greater than 2.5 centimeters (cm)

Appendix 3 Urticaria Patient Daily eDiary (cont.)

Today's Date

		–				–				

Day **Month** **Year**

Please complete this section <u>twice each day (a.m. and p.m.)</u> throughout the duration of the study (preferably at the same time each day).

(Please circle only one response.)

3. Please rate how much your hives or itch interfered with your sleep during the **past 24 hours**.

   0 No interference

   1 Mild, little interference with sleep

   2 Moderate, awoke occasionally, some interference with sleep

   3 Substantial, woke up often, severe interference with sleep

4. Please rate how much your hives or itch interfered with your daily activities during **the past 24 hours**. This could include work, school, sports, hobbies, and activities with friends and family.

   0 No interference

   1 Mild, little interference with daily activities

   2 Moderate, some interference with daily activities

   3 Substantial, severe interference with daily activities

Appendix 3 Urticaria Patient Daily eDiary (cont.)

These next questions are about your symptoms and how you managed them during the past 24 hours.

5. During the **past 24 hours**, did you use loratadine or cetirizine in order to control symptoms of your skin condition such as itch or hives?

   0 = No

   1 = Yes

6a. During the **past 24 hours**, did you have any rapid swelling on your face, (especially your eyelids or lips), inside your mouth (including your throat or tongue), or elsewhere on your body? This rapid swelling, also called angioedema, is at a deeper level under your skin than hives.

   0 = No **(GO TO Question 7)**   1 = Yes

6b. If Yes, how did you treat this rapid swelling? **(Circle all that apply.)**

   0 Did nothing (GO TO Question 7)

   1 Took some prescription or non-prescription medication

   2 Called my doctor, nurse or nurse practitioner

   3 Went to see my doctor, nurse, or nurse practitioner

   4 Went to the emergency room at the hospital

   5 Was hospitalized

7. During the **past 24 hours**, did you or someone else call your doctor, nurse or nurse practitioner because of your skin condition?

   0 = No   1 = Yes

[redacted]

[redacted]

[redacted]

[redacted]

[redacted]

[redacted]

[redacted] [redacted] [redacted] [redacted] [redacted]

[redacted]

[redacted] [redacted] [redacted] [redacted] [redacted]

[redacted]

[redacted] [redacted] [redacted] [redacted] [redacted]

[redacted]

[redacted] [redacted] [redacted] [redacted] [redacted]

Final Protocols: Version 5 (Germany) and Version 5 (Canada), including Statistical Considerations and Analysis Plan (Section 6)

PROTOCOL

**TITLE:**	**A PHASE II, MULTICENTER, RANDOMIZED, DOUBLE-BLIND, PLACEBO-CONTROLLED PILOT AND DOSE-RANGING STUDY OF GDC-0853 IN PATIENTS WITH REFRACTORY CHRONIC SPONTANEOUS URTICARIA (CSU)**
**PROTOCOL NUMBER:**	GS39684
**VERSION NUMBER:**	5 (Canada)
**EUDRACT NUMBER:**	2016-004624-35
**TEST PRODUCT:**	Fenebrutinib (GDC-0853, RO7010939)
**MEDICAL MONITOR:**	[redacted]
**SPONSOR:**	Genentech, Inc.
**DATE FINAL:**	Version 1: 13 December 2016
**DATES AMENDED:**	Version 2 (Germany Only): 11 April 2017<br>Version 3: 19 December 2017<br>Version 4: 9 August 2018<br>Version 5 (Canada): See electronic date stamp below.

PROTOCOL AMENDMENT APPROVAL

Approver's Name	Title	Date and Time (UTC)
[redacted]	Company Signatory	01-Feb-2019 22:03:19

CONFIDENTIAL

The information contained in this document, especially any unpublished data, is the property of Genentech, Inc. (or under its control) and therefore is provided to you in confidence as an investigator, potential investigator, or consultant, for review by you, your staff, and an applicable Ethics Committee or Institutional Review Board. It is understood that this information will not be disclosed to others without written authorization from Genentech except to the extent necessary to obtain informed consent from persons to whom the drug may be administered.

PROTOCOL AMENDMENT, VERSION 5 (CANADA): RATIONALE

Protocol GS39684 has been amended to remove the option for patients to enter the open-label extension (OLE) Study GS40868 after completing the 8-week treatment period. Study GS40868 has not been approved by the Canadian health authority so the OLE study is not available to patients.

The following changes have been made:

- OLE language has been removed throughout the protocol. Figure 1, "Study Schema," has been updated. Section 3.1.2 has been deleted and the appropriate sections have been revised (Sections 3.1, 3.1.2, and 4.3.2.1 and Appendix 1).
- Exclusion criteria specific to German sites has been deleted (Section 4.1.2).
- Post-trial access to fenebrutinib has been updated for the situation in which continued access to the study drug is unlikely per the Roche Global Policy on Continued Access to Investigation Medicinal Product and there are no plans for an extension study (Section 4.3.4).
- An administrative error for the Urticaria Patient Daily eDiary has been corrected (Appendix 3).

Additional minor changes have been made to improve clarity and consistency. Substantive new information appears in italics. This amendment represents cumulative changes to the original protocol.

TABLE OF CONTENTS

PROTOCOL AMENDMENT ACCEPTANCE FORM 10

PROTOCOL SYNOPSIS 11

1. BACKGROUND 24

1.1 Background on Chronic Spontaneous Urticaria 24

1.2 Background on Bruton's Tyrosine Kinase and Fenebrutinib 25

1.2.1 Bruton's Tyrosine Kinase 25

1.2.2 Nonclinical Experience with Fenebrutinib 26

1.2.3 Clinical Experience with Fenebrutinib 27

1.3 Study Rationale and Benefit-Risk Assessment...................... 29

1.3.1 Infections 30

1.3.2 Bleeding.. 30

1.3.3 Cytopenias.. 31

1.3.4 Hepatotoxicity 31

1.3.5 Cardiovascular Effects.. 31

1.3.6 Malignancy 31

2. OBJECTIVES AND ENDPOINTS 32

3. STUDY DESIGN 33

3.1 Description of the Study.. 33

3.1.1 Internal Monitoring Committee.. 36

3.2 End of Study and Length of Study 37

3.3 Rationale for Study Design 37

3.3.1 Rationale for Fenebrutinib Dose and Schedule 37

3.3.2 Rationale for Patient Population 38

3.3.3 Rationale for Control Group.. 39

[redacted]

3.3.5 Rationale for Pharmacokinetic Sample Collection Schedule.. 39

3.3.6 Rationale for Efficacy Endpoints.. 39

4. MATERIALS AND METHODS 40

4.1 Patients 40
4.1.1 Inclusion Criteria 40
4.1.2 Exclusion Criteria 42
4.2 Method of Treatment Assignment and Blinding 45
4.2.1 Randomization and Blinding 45
4.2.2 Unblinding 46
4.3 Study Treatment 46
4.3.1 Formulation, Packaging, and Handling 47
4.3.1.1 Fenebrutinib and Placebo 47
4.3.1.2 Background Therapy: Standard-of-Care H1 Antihistamines for CSU 47
4.3.2 Dosage, Administration, and Compliance of Fenebrutinib and Placebo 47
4.3.2.1 Fenebrutinib and Placebo Dose and Administration 47
4.3.2.2 Fenebrutinib and Placebo Compliance 48
4.3.2.3 Background Therapy: Standard-of-Care H1 Antihistamines for CSU 49
4.3.3 Investigational Medicinal Product Accountability 49
4.3.4 Post-Trial Access to Fenebrutinib 50
4.4 Concomitant Therapy and Additional Restrictions 50
4.4.1 Permitted Therapy 50
4.4.2 Cautionary Therapy 51
4.4.2.1 Acid-Reducing Agents 51
4.4.2.2 Statins 51
4.4.2.3 CYP3A and BCRP-Mediated Drug Interactions 51
4.4.3 Prohibited Therapy 53
4.4.3.1 Live or Attenuated Vaccinations 53
4.4.4 Prohibited Food 53
4.4.5 Additional Restrictions 53
4.5 Study Assessments 54
4.5.1 Informed Consent Forms and Screening Log 54
4.5.2 Eligibility at Screening 54
4.5.2.1 Retesting: Laboratory Inclusion/Exclusion 54

4.5.2.2 Re-Screening 54
4.5.3 Medical History and Demographic Data 54
4.5.4 Physical Examinations 55
4.5.5 Vital Signs 55
4.5.6 FricTest 55
4.5.7 Laboratory, Biomarker, and Other Biological Samples 55
4.5.8 Electrocardiograms 57
4.5.9 Patient-Reported Outcomes 58
4.5.9.1 Urticaria Patient Daily eDiary 58
4.5.9.2 Urticaria Activity Score 59
[redacted]
4.6 Patient, Treatment, Study, and Site Discontinuation 60
4.6.1 Patient Discontinuation 60
4.6.2 Study Treatment Discontinuation 61
4.6.3 Study and Site Discontinuation 61
5. ASSESSMENT OF SAFETY 62
5.1 Safety Plan 62
5.1.1 Safety Plan for Potential Risks Associated with Fenebrutinib 62
5.1.1.1 Infections 62
5.1.1.2 Vaccinations 64
5.1.1.3 Bleeding 64
5.1.1.4 Cytopenias 65
5.1.1.5 Gastrointestinal Effects 66
5.1.1.6 Hepatotoxicity 66
5.1.1.7 Cardiovascular Effects 67
5.1.1.8 Vascular Inflammation 68
5.1.1.9 Malignancy 68
5.1.2 Management of Patients Who Experience Specific Adverse Events 69
5.1.2.1 Management of Specific Adverse Events 69

5.1.2.2 Management of Increases in QT Interval 70
5.2 Safety Parameters and Definitions 71
5.2.1 Adverse Events 71
5.2.2 Serious Adverse Events (Immediately Reportable to the Sponsor) 71
5.2.3 Adverse Events of Special Interest (Immediately Reportable to the Sponsor).. 72
5.3 Methods and Timing for Capturing and Assessing Safety Parameters... 73
5.3.1 Adverse Event Reporting Period 73
5.3.2 Eliciting Adverse Event Information 73
5.3.3 Assessment of Severity of Adverse Events 73
5.3.4 Assessment of Causality of Adverse Events 74
5.3.5 Procedures for Recording Adverse Events............................ 75
5.3.5.1 Diagnosis versus Signs and Symptoms................................. 75
5.3.5.2 Adverse Events That Are Secondary to Other Events.. 75
5.3.5.3 Persistent or Recurrent Adverse Events................................ 75
5.3.5.4 Abnormal Laboratory Values 76
5.3.5.5 Abnormal Vital Sign Values 77
5.3.5.6 Abnormal Liver Function Tests 77
5.3.5.7 Deaths 77
5.3.5.8 Preexisting Medical Conditions... 78
5.3.5.9 Lack of Efficacy or Worsening of Chronic Spontaneous Urticaria 78
5.3.5.10 Hospitalization or Prolonged Hospitalization.......................... 78
5.3.5.11 Adverse Events Associated with an Overdose or Error in Drug Administration 79
5.3.5.12 Patient-Reported Outcome Data 79
5.4 Immediate Reporting Requirements from Investigator to Sponsor.. 79
5.4.1 Emergency Medical Contacts 80
5.4.2 Reporting Requirements for Serious Adverse Events and Adverse Events of Special Interest 80
5.4.2.1 Events That Occur prior to Study Drug Initiation.................... 80

5.4.2.2 Events That Occur after Study Drug Initiation 80
5.4.3 Reporting Requirements for Pregnancies 81
5.4.3.1 Pregnancies in Female Patients 81
5.4.3.2 Pregnancies in Female Partners of Male Patients 81
5.4.3.3 Congenital Anomalies/Birth Defects and Abortions 82
5.5 Follow-Up of Patients after Adverse Events 82
5.5.1 Investigator Follow-Up 82
5.5.2 Sponsor Follow-Up 82
5.6 Adverse Events That Occur after the Adverse Event Reporting Period 82
5.7 Expedited Reporting to Health Authorities, Investigators, Institutional Review Boards, and Ethics Committees 83

6. STATISTICAL CONSIDERATIONS AND ANALYSIS PLAN 83
6.1 Determination of Sample Size 83
6.1.1 Cohort 1: Pilot Assessment 83
6.1.2 Cohort 2: Dose-Ranging Assessment 84
6.2 Summaries of Conduct of Study 84
6.3 Summaries of Treatment Group Comparability 85
6.4 Efficacy Analyses 85
6.4.1 Primary Efficacy Endpoint 85
6.4.2 Secondary Efficacy Endpoints 86
6.4.3 Exploratory Efficacy Endpoints 86
6.5 Safety Analyses 87
6.6 Pharmacokinetic Analyses 87
[redacted]
6.8 Interim Analysis 88
6.8.1 Cohort 1: Planned Interim Analysis 88
6.8.2 Cohort 2: Optional Interim Analysis 88

7. DATA COLLECTION AND MANAGEMENT 88
7.1 Data Quality Assurance 88
7.2 Electronic Case Report Forms 89
7.3 Electronic Patient-Reported Outcome Data 89

7.4 Source Data Documentation ... 89
7.5 Use of Computerized Systems ... 90
7.6 Retention of Records ... 90

8. ETHICAL CONSIDERATIONS ... 91
8.1 Compliance with Laws and Regulations ... 91
8.2 Informed Consent ... 91
8.3 Institutional Review Board or Ethics Committee ... 92
8.4 Confidentiality ... 92
8.5 Financial Disclosure ... 93

9. STUDY DOCUMENTATION, MONITORING, AND ADMINISTRATION ... 93
9.1 Study Documentation ... 93
9.2 Protocol Deviations ... 93
9.3 Site Inspections ... 93
9.4 Administrative Structure ... 94
9.5 Publication of Data and Protection of Trade Secrets ... 94
9.6 Protocol Amendments ... 95

10. REFERENCES ... 96

LIST OF TABLES

Table 1 Objectives and Corresponding Endpoints ... 32
Table 2 Twice Daily Patient Assessment of CSU Disease Activity (UAS Scale) ... 40
Table 3 Fenebrutinib Dosing Regimen by Treatment Arm (Cohort 2) ... 48
[redacted]
Table 5 Guidelines for Management of Patients Who Experience Specific Adverse Events ... 69
Table 6 Adverse Event Severity Grading Scale ... 74
Table 7 Causal Attribution Guidance ... 74

LIST OF FIGURES

Figure 1 Study Schema ... 36

LIST OF APPENDICES

Appendix 1 Schedule of Activities (Cohorts 1 and 2) ... 100
Appendix 2 Childbearing Potential, Pregnancy Testing, and Contraception ... 105
Appendix 3 Urticaria Patient Daily eDiary ... 107
[redacted]
Appendix 5 Concomitant Medications (Including Foods and Herbal Products) ... 113

PROTOCOL AMENDMENT ACCEPTANCE FORM

**TITLE:**	**A PHASE II, MULTICENTER, RANDOMIZED, DOUBLE-BLIND, PLACEBO-CONTROLLED PILOT AND DOSE-RANGING STUDY OF GDC-0853 IN PATIENTS WITH REFRACTORY CHRONIC SPONTANEOUS URTICARIA (CSU)**
**PROTOCOL NUMBER:**	GS39684
**VERSION NUMBER:**	5 (Canada)
**EUDRACT NUMBER:**	2016-004624-35
**TEST PRODUCT:**	Fenebrutinib (GDC-0853, RO7010939)
**MEDICAL MONITOR:**	[redacted]
**SPONSOR:**	Genentech, Inc.

I agree to conduct the study in accordance with the current protocol.

____

Principal Investigator's Name (print)

____ ____________________

Principal Investigator's Signature Date

Please return a copy of the signed form as instructed by the CRO. Please retain the original for your study files.

PROTOCOL SYNOPSIS

**TITLE:**	**A PHASE II, MULTICENTER, RANDOMIZED, DOUBLE-BLIND, PLACEBO-CONTROLLED PILOT AND DOSE-RANGING STUDY OF GDC-0853 IN PATIENTS WITH REFRACTORY CHRONIC SPONTANEOUS URTICARIA (CSU)**
**PROTOCOL NUMBER:**	GS39684
**VERSION NUMBER:**	5 (Canada)
**EUDRACT NUMBER:**	2016-004624-35
**TEST PRODUCT:**	Fenebrutinib (GDC-0853, RO7010939)
**PHASE:**	Phase II
**INDICATION:**	Refractory Chronic Spontaneous Urticaria (CSU)
**SPONSOR:**	Genentech, Inc.

Objectives and Endpoints

This pilot and dose-ranging study will evaluate the efficacy, safety, and pharmacokinetics of fenebrutinib compared with placebo in patients with chronic spontaneous urticaria (CSU) refractory to anti-histamines (up to 4 times the approved dose per local treatment guidelines). Specific objectives and corresponding endpoints for the study are outlined below.

Objectives and Corresponding Endpoints

Objectives	Corresponding Endpoints
**Efficacy Objective:**	
• To evaluate the efficacy of fenebrutinib compared with placebo in patients with CSU who are refractory to anti-histamines	**Primary Endpoint:**<br>• Change from baseline in the UAS7 at Day 57 (Week 8)<br>**Secondary Endpoints:**<br>• Proportion of patients who are well controlled (UAS7 ≤ 6) at Day 57<br>• Change from baseline in the UAS7 at Day 29 (Week 4)<br>**Exploratory Endpoints:**<br>• Change from baseline in the weekly itch score at Day 29<br>• Change from baseline in the weekly itch score at Day 57<br>• Change from baseline in the weekly hives score at Day 57<br>• Proportion of patients who are well controlled (UAS7 ≤ 6) at Day 29<br>• Proportion of patients who achieve complete response (UAS7 = 0) at Day 29<br>• Proportion of patients who achieve complete response (UAS7 = 0) at Day 57<br>• Proportion of patients achieving MID in UAS7 at Day 57 (reduction from baseline ≥ 11 points)<br>• Proportion of patients achieving MID in the weekly itch score at Day 57 (reduction from baseline ≥ 5 points)

Objectives and Corresponding Endpoints (Continued)

Objectives	Corresponding Endpoints
	Exploratory Endpoints (Continued):<br>• Time to achieving MID in UAS7 (reduction from baseline ≥ 11 points)<br>[redacted]
**Safety Objective:**	
• To evaluate the safety of fenebrutinib compared with placebo	• The nature, frequency, timing, and severity of adverse events<br>• Change from baseline in targeted vital signs, physical examination findings, ECGs, and clinical laboratory results following fenebrutinib administration
**Pharmacokinetic Objective:**	
• To characterize the pharmacokinetics of fenebrutinib in patients	• Plasma concentrations of fenebrutinib at specified timepoints
[redacted]	
[redacted]	[redacted]
[redacted]	[redacted]
[redacted]	[redacted]
[redacted]	
[redacted]	[redacted]

AUC_{0-t} = area under the concentration–time curve from time 0 to time t [redacted] CL/F = apparent clearance; C_{max} = maximum concentration observed; C_{trough} = steady-state concentration at the end of a dosing interval; CSU = chronic spontaneous urticaria; MID = minimally important difference; [redacted] PK = pharmacokinetic; $t_{1/2}$ = half-life; t_{max} = time to maximum concentration; UAS7 = Urticaria Activity Score over 7 days [redacted]

Study Design

Description of Study

This pilot and dose-ranging study is a multicenter, randomized, double-blind, placebo-controlled, parallel-group study of the efficacy and safety of fenebrutinib as add-on therapy for the treatment of adult patients 18 – 75 years old who have been diagnosed with CSU and who remain symptomatic despite treatment with H1 antihistamines (including doses up to 4 times the approved dose per local treatment guidelines). The study will consist of two cohorts. Cohort 1 will enroll approximately 45 patients across multiple sites. After screening, eligible patients will

be randomly allocated in a 2:1 ratio to receive fenebrutinib 200 mg orally (PO) twice daily (BID) or matching placebo for 8 weeks and will maintain stable doses of standard-of-care H1 antihistamine therapy throughout the study. On the basis of results from an interim analysis of Cohort 1, a dose-ranging cohort, Cohort 2, will be opened and approximately 120 patients will be randomly allocated in a 1:1:1:1 ratio to receive 50 mg PO daily (QD), 150 mg PO QD, 200 mg PO BID of fenebrutinib, or placebo, respectively, for 8 weeks and will maintain stable doses of standard-of-care H1 antihistamine therapy (background therapy) throughout the study.

Both cohorts will consist of 3 distinct study periods over a time-period of 14 weeks as outlined below:

- Screening period: Day −14 to Day −1
- Treatment Period: Day 1 to Day 57 (Week 0 to Week 8)
- Follow-Up Period: Day 57 to Day 85 (Week 8 to Week 12)

Patients in both cohorts will have a screening period of approximately 2 weeks to establish their eligibility for the study and baseline symptom scores. For the duration of the screening period, patients must maintain stable doses of their pre-screening combination therapy with standard-of-care H1 antihistamines (i.e., up to 4 times the approved dose per local treatment guidelines). The screening period will consist of visits at Day −14 and Day −7. Patients must meet all of the following criteria to enter the screening period:

- Documented treatment with a regimen that includes standard-of-care H1 antihistamine for CSU at Day −14 and for at least the 3 consecutive days immediately prior to Day −14
- Willing and able to complete a symptom electronic diary (Urticaria Patient Daily eDiary) twice daily throughout the screening period to establish the patient's Urticaria Activity Score over 7 days (UAS7) score.

To be eligible for randomization in both cohorts, for the 7 days prior to randomization, patients must meet all of the following:

- Seven consecutive days of entries in the Urticaria Patient Daily eDiary, and
- UAS7 symptom score of ≥16 (range: 0–42)

Only in exceptional circumstances, when information concerning eligibility is outstanding (e.g., delayed laboratory results), will a longer screening period be permitted up to 3 business days. Upon approval from the Medical Monitor, patients may be re-screened or may be retested during the screening period. Circumstances that may permit re-screening or retesting include, but are not limited to, a laboratory test result that does not meet eligibility requirements.

On Day 1, eligible patients in Cohort 1 will be randomly allocated in a 2:1 ratio to receive fenebrutinib 200 mg orally (PO) twice daily (BID) or placebo for 8 weeks. Eligible patients in Cohort 2 will be will be randomly allocated in a 1:1:1:1 ratio to receive 50 mg PO daily (QD), 150 mg PO QD, 200 mg PO BID of fenebrutinib, or placebo, respectively, for 8 weeks. The primary efficacy endpoint will be at Day 57 (Week 8). Throughout the treatment period, patients must maintain stable doses of their pre-randomization H1 antihistamine therapy.

After completion of the 8-week treatment period, patients in both cohorts will enter a 4-week safety follow-up period to allow for further characterization of the pharmacokinetics and pharmacodynamics of fenebrutinib, and collection of additional efficacy and safety data. During safety follow-up period, no study treatment will be given; patients must maintain stable doses of their pre-randomization CSU H1 antihistamine treatment (background therapy). In the safety follow-up period, patients may add up to one additional H1 antihistamine therapy in case of worsened symptoms. The goal of allowing additional H1 antihistamine therapy after the treatment period is to reduce patient dropout for improved safety evaluation.

In addition to their daily background therapy, for the duration of the study all patients will be able to use a single approved dose of loratadine (10 mg maximum) or cetirizine (10 mg maximum) within a 24-hour period as rescue medication if symptoms worsen. If a patient needs rescue therapy and is already on background treatment with cetirizine or loratadine, the patient may receive 10 mg more of the same drug only if the total daily dose remains below 4 times the approved dose. Otherwise, the alternate rescue medication may be used. Patients should record the use of this medication in their eDiary. Patients receiving proton-pump inhibitors

(PPIs) or H2 receptor antagonists (H2RAs) should be stabilized on a regimen beginning at least 2 weeks prior to randomization and continuing throughout the study.

Internal Monitoring Committee

For Cohort 2, periodic safety reviews and any interim analysis will be performed by the Sponsor's internal monitoring committee (IMC) as outlined in the IMC charter. This committee will be unblinded to treatment assignments and will include Sponsor representatives from the following functions: Clinical Science, Drug Safety, Biostatistics, and Statistical Programming and Analysis. The IMC members will not have direct contact with investigational staff or site monitors. The IMC may decide to unblind the study team to enable decision-making and potential interactions with regulatory bodies. The IMC may invite representatives from other functional areas on an ad-hoc basis when additional expertise is required (e.g., Clinical Pharmacology, Research, etc.) or additional Sponsor scientists to participate in data analyses and review.

At any time during the study, the Sponsor may choose to inactivate and suspend enrollment and further dosing for a given treatment arm (in Cohort 2) or reduce the dose due to safety concerns and as guided by the IMC. In Cohort 2, subsequently enrolled patients will be randomly allocated to the remaining active arms.

Number of Patients

Approximately 45 patients, aged 18 to 75 years old who have been diagnosed with refractory CSU and who remain symptomatic despite standard-of-care H1 antihistamine (i.e., up to 4 times the approved dose per local treatment guidelines), will be enrolled in Cohort 1. On the basis of results from an interim analysis of Cohort 1, a dose-ranging cohort, Cohort 2, will be opened and approximately 120 patients will be enrolled.

Target Population

<u>Inclusion Criteria</u>

Patients in Cohort 1 and 2 must meet the following criteria for study entry:

- Willing to give written informed consent, adhere to the visit schedules, comply with the study drug regimen, and meet other study requirements
- Aged 18 – 75 years, inclusive
- Diagnosis of CSU refractory to H1 antihistamines at the time of randomization, as defined by all of the following:
  - The presence of itch and hives for >6 consecutive weeks at any time prior to enrollment despite current use of H1 antihistamine, consistent with standard of care (i.e., up to 4 times the approved dose per local treatment guidelines) during this time period
  - UAS7 score ≥ 16 during the 7 days prior to randomization (Day 1)
  - Patients must have been on daily stable doses of H1 antihistamine, consistent with standard of care (i.e., up to 4 times the approved dose per local treatment guidelines) treatment for CSU starting at least 3 consecutive days immediately prior to the screening visit through Day 1and must document current use on all visits.
  - CSU diagnosis for ≥ 6 months
- Willing and able to complete an Urticaria Patient Daily eDiary for the duration of the study
- Completion of 7 days of the Urticaria Patient Daily eDiary entries in the 7 days prior to randomization (7 of 7 days must be completed [i.e., must complete an entry every day] with up to 2 non-consecutive entries missed)
- No evidence of active or latent or inadequately treated infection with tuberculosis (TB) as defined by the following:
  - A negative QuantiFERON-TB-Gold® (QFT) performed at the screening visit or within the 3 months prior to screening (for German sites only: QFT is the preferred test)

    If QFT is unavailable, a negative Mantoux purified protein derivative (PPD) skin test as defined by the Centers for Disease Control and Prevention guidelines, may be performed at the screening visit or within the 3 months prior to screening -AND-

Any additional procedures (e.g., chest X-Ray) required per local guidelines to rule out latent or active TB

NOTE: A documented negative screening for TB via the PPD test or a negative QFT within 3 months prior to screening (and if required per local standard of care, a chest X-ray), is sufficient and no further screening with QFT is required.

Patients with a history of Bacille Calmette-Guérin (BCG) vaccination should be screened using the QFT test, only.

- An indeterminate QFT test should be repeated.
- A positive QFT test or two successive indeterminate QFT results should be considered a positive diagnostic TB test.
- An indeterminate QFT test followed by a negative QFT test should be considered a negative diagnostic TB test.

- Only for patients currently receiving PPIs or H2RAs: Treatment must be at a stable dose during the 2 week screening period prior to randomization and with a plan to remain at a stable dose for the duration of the study
- For women of childbearing potential: Agreement to remain abstinent (refrain from heterosexual intercourse) or use contraceptive methods that result in a failure rate of $<1\%$ per year during the treatment period and for at least 4 weeks after the last dose of study drug. Women must refrain from donating eggs during this same period.

  A woman is considered to be of childbearing potential if she is postmenarcheal, has not reached a postmenopausal state (≥ 12 continuous months of amenorrhea with no identified cause other than menopause), and has not undergone surgical sterilization (removal of ovaries and/or uterus).

  Examples of contraceptive methods with a failure rate of $<1\%$ per year include bilateral tubal ligation, male sterilization, established proper use of hormonal contraceptives that inhibit ovulation, hormone-releasing intrauterine devices, and copper intrauterine devices. Women using estrogen-containing hormonal contraceptives as a method of contraception <u>must</u> also use a barrier, such as a male condom, in conjunction with the hormonal contraceptives.

  The reliability of sexual abstinence should be evaluated in relation to the duration of the clinical trial and the preferred and usual lifestyle of the patient. Periodic abstinence (e.g., calendar, ovulation, symptothermal, or post-ovulation methods) and withdrawal are not acceptable methods of contraception.

- For men: agreement to remain abstinent (refrain from heterosexual intercourse) or use contraceptive measures and agreement to refrain from donating sperm, as defined below:

  With female partners of childbearing potential or pregnant female partners, men must remain abstinent or use a condom during the treatment period and for at least 4 weeks after the last dose of study treatment to avoid exposing the embryo. Men must refrain from donating sperm during this same period.

  The reliability of sexual abstinence should be evaluated in relation to the duration of the clinical trial and the preferred and usual lifestyle of the patient. Periodic abstinence (e.g., calendar, ovulation, symptothermal, or post-ovulation methods) and withdrawal are not acceptable methods of contraception.

<u>Exclusion Criteria</u>

Patients in Cohort 1 and 2 who meet any of the following criteria will be excluded from study entry:

- Treatment with omalizumab or other monoclonal antibody therapies used to treat CSU within 4 months prior to screening or primary nonresponse to omalizumab
- Use of a non-biologic investigational drug or participation in an investigational study with a non-biologic drug within 30 days prior to study drug administration on Day 1 (or within 5 half-lives of the investigational product, whichever is greater)

- Use of a biologic investigational therapy or participation in an investigational study involving biologic therapy within 90 days or 5 half-lives, whichever is greater, prior to study drug administration on Day 1
- Previous treatment with fenebrutinib or other Bruton's tyrosine kinase (BTK) inhibitors
- Patients whose urticaria is solely due to physical urticaria
- Other diseases with symptoms of urticaria or angioedema, including urticarial vasculitis, urticaria pigmentosa, erythema multiforme, mastocytosis, hereditary or acquired angioedema, lymphoma, or leukemia
- Atopic dermatitis, bullous pemphigoid, dermatitis herpetiformis, or other skin disease associated with itch such as psoriasis
- Routine (daily or every other day during 5 or more consecutive days) doses of the following medications within 30 days prior to screening: systemic or cutaneous (topical) corticosteroids (prescription or over the counter), hydroxychloroquine, methotrexate, cyclosporine, or cyclophosphamide
- Prior utilization of intravenous (IV) steroids for treatment of laryngeal angioedema
- IV immunoglobulin G (IVIG) or plasmapheresis within 30 days prior to screening
- History of anaphylactic shock without clearly identifiable avoidable antigen (e.g., due to food allergy)
- Hypersensitivity to fenebrutinib or any component of the formulation
- Major surgery, within 8 weeks prior to screening or surgery planned prior to end of study (12 weeks after randomization)
- Require any prohibited concomitant medications
- History of live attenuated vaccine within 6 weeks prior to randomization or requirement to receive these vaccinations at any time during study drug treatment

    Seasonal influenza and H1N1 vaccination is permitted if the inactivated vaccine formulation is administered.
- Evidence of clinically significant cardiac, neurologic, psychiatric, pulmonary, renal, hepatic, endocrine (including uncontrolled diabetes mellitus), metabolic, or gastrointestinal (GI) disease that, in the investigator's opinion, would compromise the safety of the patient, interfere with the interpretation of the study results, or otherwise preclude patient participation.

    Any items that are cause for uncertainty must be reviewed with the Medical Monitor.
- Uncontrolled disease states, such as asthma, psoriasis, or inflammatory bowel disease, where flares are commonly treated with oral or parenteral corticosteroids
- History of vasculitis
- Current liver disease
- Any known active infection (with the exception of fungal nail infections or oral herpes)
- History of recurrent bacterial, viral, mycobacterial or fungal infections (defined as >2 similar episodes requiring anti-microbial treatment within the previous 12 months), with the exception of recurrent oral or genital herpes (herpes simplex virus 1/herpes simplex virus 2) or uncomplicated urinary tract infections in females.
- Any history of opportunistic infections that, in the investigator or Sponsor's judgment, would raise safety concerns regarding the patient's participation in the study
- Any major episode of infection requiring hospitalization or treatment with IV antimicrobials within 8 weeks prior to and during screening or treatment with oral antimicrobials within 2 weeks prior to and during screening

    Antimicrobials include antifungal, antibacterial, and antiviral agents.
- History of or currently active primary or secondary immunodeficiency, including known history of HIV infection

- Evidence of chronic and/or active hepatitis B or C

  Positive hepatitis B surface antigen (HBsAg) or hepatitis C serology (regardless of treatment status)

  Positive hepatitis B core antibody (HBcAb)

- History of cancer, including hematologic malignancy and solid tumors, within 10 years before screening

  Basal or squamous cell carcinoma of the skin that has been excised and is considered cured and in situ carcinoma of the cervix treated with apparent success by curative therapy > 1 year prior to screening are not exclusionary.

- Women who are pregnant, nursing (breastfeeding), or intending to become pregnant during the study or within 4 weeks after completion of the study
- For women of childbearing potential (including those who have had a tubal ligation): positive serum pregnancy test result at screening or on Day 1.

  A serum pregnancy test is needed on Day 1 only if the urine pregnancy test is positive.

- History of alcohol, drug (e.g., tetrahydrocannabinol [THC], marijuana), or chemical abuse within the 12 months prior to screening as determined by the investigator
- Need for systemic anti-coagulation with warfarin, other oral or injectable anti-coagulants, or anti-platelet agents other than non-steroidal anti-inflammatory drugs (NSAIDs), aspirin, and other salicylates
- History of non-gallstone–related pancreatitis or chronic pancreatitis
- History of hospitalizations or transfusion for a GI bleed
- History of cerebrovascular accident (CVA) within 10 years or any history of hemorrhagic CVA
- History of spontaneous intracranial hemorrhage or history of traumatic intracranial hemorrhage within 10 years
- Known bleeding diathesis
- Screening 12-lead ECG that demonstrates clinically relevant abnormalities that may affect patient safety or interpretation of study results, including

  QT interval corrected using Fridericia's formula (QTcF) > 440 ms demonstrated by at least two ECGs > 30 minutes apart

- History of ventricular dysrhythmias or risk factors for ventricular dysrhythmias such as long QT syndrome and other genetic risk factors (e.g., Brugada syndrome), structural heart disease (e.g., severe left ventricular systolic dysfunction, severe left ventricular hypertrophy), coronary heart disease (symptomatic, or with ischemia demonstrated by diagnostic testing, prior coronary artery bypass grafting, or coronary lesions > 70% diameter stenosis that have not been or cannot be re-vascularized), clinically significant electrolyte abnormalities (e.g., hypokalemia, hypomagnesemia, hypocalcemia), or family history of sudden unexplained death or cardiac ion channel mutations (e.g., congenital long QT syndrome)
- Current treatment with medications that are well known to prolong the QT interval (see https://crediblemeds.org/index.php/login/dlcheck) at doses that have a clinically meaningful effect on QT, as determined by the investigator; the investigator may contact the Sponsor for confirmation if needed
- Current treatment with astemizole, terfenadine, and/or ebastine
- Any condition possibly affecting oral drug absorption (e.g., gastrectomy, clinically significant diabetic gastroenteropathy, or certain types of bariatric surgery such as gastric bypass)

  Procedures such as gastric banding, that simply divide the stomach into separate chambers, are not exclusionary.

- Any uncontrolled clinically significant laboratory abnormality that would affect safety, interpretation of study data, or the patient's participation in the study

The following exclusion criteria are based on screening laboratory tests. Laboratory tests may be repeated once during the screening period unless otherwise indicated:

- Creatinine $>$ 1.5 times the upper limit of normal (ULN; may be repeated if 1.5–2 $\times$ ULN)
- Creatinine clearance $<$ 70 mL/min (may be repeated if 60–69 mL/min) as estimated by the Cockcroft-Gault Equation
- ALT or AST $>$ 1.5 times ULN (may be repeated if 1.5–3 $\times$ ULN)
- Total bilirubin $>$ ULN (may be repeated if 1–3 $\times$ ULN)
- Hemoglobin $<$ 11 g/dL (may be repeated if 10–10.9 g/dL)
- ANC $<$ 1.5 $\times$ 10^9/L (may be repeated if 1.2–1.5 $\times$ 10^9/L)
- Platelet count $<$ 100 $\times$ 10^9/L (may be repeated if 80–100 $\times$ 10^9/L)
- IgG $<$ 500 mg/dL (should not be repeated)
- Abnormalities in hepatic synthetic function tests (e.g., PT, INR, PTT, albumin) judged by the investigator to be clinically significant

End of Study

The end of this study is defined as the date when all patients have completed the study completion visit or early termination visit or have otherwise been discontinued from the study.

Length of Study

The total duration of this study for each subject is approximately 14 weeks, including screening, treatment, and safety follow-up periods.

The total length of the study, from screening of the first patient to the end of the study, is expected to be approximately 19 months.

Investigational Medicinal Products

The investigational medicinal product for this study is fenebrutinib. For Cohort 1, patients will receive fenebrutinib 200 mg PO BID or placebo for 8 weeks. For Cohort 2, will receive fenebrutinib 50 mg QD, 150 mg QD, 200 mg BID, or placebo for 8 weeks.

Non-Investigational Medicinal Products

Throughout the treatment period, patients must maintain stable doses of their pre-randomization H1 antihistamine therapy. In addition to their daily background therapy, for the duration of the study all patients will be able to use a single approved dose of loratadine (10 mg maximum) or cetirizine (10 mg maximum) within a 24-hour period as rescue medication if symptoms worsen. If a patient needs rescue therapy and is already on background treatment with cetirizine or loratadine, the patient may receive 10 mg more of the same drug only if the total daily dose remains below 4 times the approved dose. Otherwise, the alternate rescue medication may be used.

Statistical Methods

Primary Analysis

The primary efficacy endpoint is the change from baseline in the UAS7 at Day 57 (Week 8).

The Urticaria Activity Score (UAS) is to be recorded twice daily (i.e., morning and evening) using an eDiary that will be provided to each patient. Scores ranging from 0 (none) to 3 (severe) will be entered for each of the two UAS domains consisting of number of wheals (hives) and intensity of pruritus (itch) resulting in a total possible score of 0 to 6. The daily UAS is calculated as the average of the morning and evening scores. When either the morning or evening score is missing, the non-missing UAS for that day (morning or evening) will be used as the daily UAS, and when both the morning and evening UAS are missing, the daily UAS will be deemed missing. The UAS7 is the sum of the daily UAS over the 7 days prior to the time point of interest. The baseline UAS7 will be calculated as the sum of daily UAS values over the week (7 days) prior to Day 1.

When one or more daily UAS values is missing, over the week prior to a timepoint of interest, rules for deriving UAS7 will be as follows:

- If a patient has at least 4 completed daily scores on the UAS (both domains) over the 7 days prior to the time point of interest, the UAS7 will be defined as the average of the available daily scores, multiplied by 7.
- If a patient has fewer than 4 completed daily scores on the UAS over the 7 days prior to the time point of interest, then the UAS7 will be considered missing for that time point.

The primary endpoint will be analyzed using a mixed model for repeated measures model. Additional model covariates will include baseline UAS7 and its interaction with visit. Missing data will be handled by the model under the missing-at-random assumption without need for imputation. As a sensitivity analysis, an analysis of covariance (ANCOVA) model adjusted for country and baseline UAS7 will be fit, and missing Day 57 data will be imputed by last observation carried forward.

Determination of Sample Size

Cohort 1: Pilot Assessment

The purpose of this cohort is to evaluate the efficacy of fenebrutinib compared with placebo in improving the UAS7. Point and interval estimates of the change from baseline of the UAS7 within each treatment group as well as of the difference in change from baseline of the UAS7 between treatment groups will be presented.

The cohort will enroll approximately 45 patients. Patients will be randomized in a 2:1 ratio to receive treatment with either fenebrutinib or placebo. The sample size of approximately 30 patients in the fenebrutinib arm and 15 patients in the placebo arm provides approximately 80% power to detect an 11-point difference in the UAS7 change from baseline at Day 57 between treatment groups under the following assumptions:

- The absolute change from baseline at Day 57 is normally distributed with a standard deviation of 13.
- Two-sided alpha is 0.10.
- Drop out at Day 57 is 10%, leading to a 10% loss of information.

Cohort 2: Dose-Ranging Assessment

The purpose of this cohort is estimation and hypothesis generation regarding the dose-ranging effects of fenebrutinib compared with placebo in improving the UAS7. Point and interval estimates of the change from baseline of the UAS7 within each treatment group as well as of the difference in change from baseline of the UAS7 between treatment groups vs placebo will be presented.

The cohort will enroll approximately 120 patients. Patients will be randomly allocated in a 1:1:1:1 ratio to receive treatment with one of three dose levels of fenebrutinib or placebo. The sample size of approximately 30 in each arm provides approximately 90% power to detect an 11-point difference in the UAS7 change from baseline at Day 57 between treatment groups, under the following assumptions:

- The absolute change from baseline at Day 57 is normally distributed with a standard deviation of 13
- Two-sided alpha is 0.10
- Dropout rate at Day 57 is 10%, leading to a 10% loss of information.

The overall sample size may be adjusted depending on the outcome of a planned interim analysis for Cohort 1, which will include an evaluation of these assumptions.

Interim Analysis

Cohort 1: Planned Interim Analysis

An interim analysis will be performed after approximately 33 patients have completed their 8–week treatment period. The purpose of this analysis is to assess the efficacy of the 200-mg fenebrutinib BID daily arm compared with the placebo, to guide internal decision-making around

issues such as ungating of Cohort 2, adequacy of sample sizes for safety and/or efficacy analyses in Cohort 2, or to inform further development decisions. Summaries of safety and efficacy data by treatment groups will be prepared and reviewed by Sponsor personnel who do not have direct contact with investigational staff, monitors, and patients. Further details of the interim analysis will be specified in the data analysis plan (DAP) prior to the conduct of the interim analysis. Access to treatment assignment information will follow the Sponsor’s standard procedures.

Cohort 2: Optional Interim Analysis

Given the hypothesis-generating nature of this study, the Sponsor may choose to conduct up to two interim efficacy analyses. The decision to conduct an optional interim analysis and the timing of the analysis will be documented in the Sponsor’s trial master file prior to the conduct of the interim analysis. The interim analysis will be performed and interpreted by members of the IMC. Access to treatment assignment information will follow the Sponsor’s standard procedures.

LIST OF ABBREVIATIONS AND DEFINITIONS OF TERMS

Abbreviation	Definition
ANCOVA	analysis of covariance
AUC	area under the concentration-time curve
AUC_{0-24}	area under the concentration-time curve from time 0 to 24 hours
AUC_{0-t}	area under the concentration-time curve from time 0 to time t
BCG	Bacille Calmette-Guérin
BCR	B-cell receptor
BCRP	breast cancer resistance protein
BID	twice a day
BTK	Bruton's tyrosine kinase
C_{max}	maximum observed concentration
C_{trough}	steady-state concentration at the end of a dosing interval
CIU	chronic idiopathic urticaria
CL/F	apparent clearance
CRP	C-reactive protein
CSR	clinical study report
CSU	chronic spontaneous urticaria
CTCAE	Common Terminology Criteria for Adverse Events
CVA	cerebrovascular accident
DAP	data analysis plan
DLAE	dose-limiting adverse events
DLT	dose-limiting toxicity
EC	Ethics Committee
eCRF	electronic Case Report Form
EDC	electronic data capture
FcεRI	high affinity IgE receptor
FDA	Food and Drug Administration
GI	gastrointestinal
H2RA	H2 receptor antagonist
HBcAb	hepatitis B core antibody
HBsAg	hepatitis B surface antigen
IC_{70}	70% maximal inhibitory concentration
IC_{80}	80% maximal inhibitory concentration
IC_{90}	90% maximal inhibitory concentration
ICH	International Conference on Harmonisation
Ig	immunoglobulin

Abbreviation	Definition
IL-1	interleukin 1
IL-6	interleukin 6
IMC	Internal monitoring committee
IMP	investigational medicinal product
IND	Investigational New Drug (application)
IRB	Institutional Review Board
IV	intravenous
IVIG	intravenous immunoglobulin G
IxRS	interactive voice or web-based response system
LTRA	leukotriene receptor antagonist
MAD	multiple-ascending dose
MID	minimally important difference
mITT	modified intent-to-treat
MMRM	mixed model for repeated measures
NCI	National Cancer Institute
NOAEL	no observed adverse effect level
NSAID	non-steroidal anti-inflammatory drug
PD	pharmacodynamic
PK	pharmacokinetic
PO	by mouth, orally
PPD	Purified Protein Derivative
PPI	proton-pump inhibitor
PRO	patient-reported outcome
QD	once a day
QFT	QuantiFERON-TB-Gold
QTcF	QT interval corrected using Fridericia's formula
RA	rheumatoid arthritis
SAD	single-ascending dose
SLE	systemic lupus erythematosus
$t_{1/2}$	half-life
t_{max}	time to maximum concentration
TB	tuberculosis
[redacted]	[redacted]
THC	tetrahydrocannabinol
TNF-α	tumor necrosis factor alpha
UAS	Urticaria Activity Score
UAS7	Urticaria Activity Score over 7 days

Abbreviation	Definition
[redacted]	[redacted]
XLA	X-linked agammaglobulinemia

1. BACKGROUND

1.1 BACKGROUND ON CHRONIC SPONTANEOUS URTICARIA

Chronic spontaneous urticaria (CSU, also referred to as chronic idiopathic urticaria [CIU]) is defined by the presence of wheals (hives), angioedema, or both for at least 6 weeks without an obvious cause (Greaves 2003). Previous estimates of the prevalence of CSU were approximately 0.1%, which persists in 20% of CSU patients 2 decades after diagnosis (Greaves 2000; Saini 2014). More recent evidence indicates that the point prevalence of the disease is approximately 1% (Maurer et al. 2011). Affected patients experience frequent pruritic hives with associated erythema and/or episodes of angioedema. CSU is reported to be associated with angioedema in approximately 50% of cases (McGirt et al. 2006). The classic urticaria description is a wheal and flare with a pale elevated lesion and surrounding erythema, ranging in size from a few millimeters to a few centimeters across, usually occurring in groups and often coalescing to form large confluent lesions.

The etiology of CSU is not clear. There are several theories including one proposing an infectious origin and another related to an autoimmune origin (Kaplan 2002). Some studies have found that approximately 30%−60% of patients with CSU have an autoimmune component as evidenced by the presence of a positive autologous serum skin test (Fiebiger et al. 1995; Tong et al. 1997; Zweiman et al. 1998). Another hypothesis regarding the etiology of CSU is that of a specific IgE antibody targeted to an endogenous antigen (Altrichter et al. 2011). Crosslinking of this IgE antibody docked in the high affinity IgE receptor (FcεRI) could result in the activation of skin mast cells and release of chemical mediators, such as histamine, that lead to the wheal and flare formation of a hive. In fact, recent findings in a study of more than 450 patients with CSU indicate that greater than 50% of CSU patients have IgE antibodies directed against thyroperoxidase (Altrichter et al. 2011). While an autoimmune etiology can be found in a large percentage of patients, many patients do not have an identified autoimmune etiology despite having a similar disease presentation (Ferrer 2015).

The final common pathway in CSU is the abnormal activation of mast cells and basophils in the skin. In patients with CSU, increased numbers of mast cells can be found in both affected and unaffected skin (Kay et al. 2014). Furthermore, mast cells from CSU patients are more sensitive, have lower thresholds for activation, and respond more robustly by releasing more histamine and other inflammatory mediators. Similarly, increased numbers of basophils have been seen in the lesional and non-lesional skin of patients with CSU (Ying et al. 2002). In patients with CSU, there is a paradoxical basopenia thought to be due to increased recruitment of basophils to diseased skin. The peripheral basopenia is inversely correlated with severity of disease activity. In comparison with healthy controls, studies have shown that blood basophils of CSU patients have a reduced capacity to release histamine following IgE stimulation. This paradoxical reduction is attributed to prior in vivo activation in the skin (Kern and Lichtenstein 1976). However, when basophils from CSU patients are incubated with

serum from other CSU patients or even normal sera, they release more histamine than basophils from healthy donors. Collectively, these data suggest that basophil signaling and activation are dysregulated in patients with CSU (Luquin et al. 2005).

Roughly half of patients with CSU achieve symptomatic control with H1 antihistamine therapy at approved doses. In some cases, the dose of antihistamine is increased (up to 4 times the approved dose per local treatment guidelines) and additional therapies, such as leukotriene receptor antagonists (LTRAs), are used although increased doses of antihistamines and LTRAs are not approved for the treatment of CSU. CSU can be a debilitating condition because of a lack of clinical response as well as the unpredictable course of the disease, both of which can have a profound negative influence on the patient's quality of life (Tilles 2005).

Patients may remain symptomatic despite ongoing H1 antihistamine treatment (up to 4 times the approved dose per local treatment guidelines; Powell et al. 2015), and for this group of patients, therapies such as immunosuppressants (including cyclosporine, corticosteroids, intravenous immunoglobulin G [IVIG], and methotrexate) and plasmapheresis have been used (Kozel and Sabroe 2004). These agents have variable success and may be associated with severe adverse effects. More recently, omalizumab was approved for treatment of refractory CSU/CIU.

1.2 BACKGROUND ON BRUTON'S TYROSINE KINASE AND FENEBRUTINIB

1.2.1 Bruton's Tyrosine Kinase

Discovery of the genetic basis for primary immunodeficiencies has been the source of new therapeutic targets in immunomodulatory therapies (Puri et al. 2013; Bugatti et al. 2014; Whang and Chang 2014). In humans, mutations in the gene for Bruton's tyrosine kinase (BTK), which is located on the X chromosome, can result in the development of an immunodeficiency state characterized by a significant absence of circulating B cells (Bruton 1952; Tsukada et al. 1993; Vetrie et al. 1993; Conley et al. 2005) and very low immunoglobulin levels due to a defect in B-cell differentiation at the pro– to pre–B cell stage that precludes assembly of the B-cell receptor (BCR) complex and immunoglobulin gene expression (Reth and Nielsen 2014). Affected male patients have a primary immune deficiency, X-linked agammaglobulinemia (XLA), and are susceptible to recurrent infections starting shortly after birth. Patients with XLA can live relatively normal lives on a standard therapy of intravenous (IV) immunoglobulin, which suggests that BTK can be safely inhibited, especially in people with established immune systems. IV immunoglobulin replacement therapy lowers the rate of infection, reduces hospitalization rates for patients with XLA, and has greatly improved the long-term prognosis of these patients.

BTK is essential for the differentiation and activity of B cells during immune system ontogeny and normal adaptive immune responses. BTK is activated by phosphatidylinositol 3-kinase–dependent plasma membrane recruitment and

phosphorylation on tyrosine Y551 by the Src-family kinase Lyn. Autophosphorylation and activation also occurs on tyrosine Y223 in a BTK-specific manner. Once activated, BTK induces PLCγ2- and Ca^{2+}-dependent signaling, which leads to the activation of NF-κB– and NFAT-dependent pathways leading to cellular activation and differentiation (Niiro and Clark 2002). In addition, BTK is important in FcεRI signaling in both basophils and mast cells, the key cell types in the pathogenesis of CSU. BTK null mice have impaired FcεRI signaling resulting in decreased histamine and inflammatory cytokine release (Hata et al. 1998; Iyer et al. 2011).

1.2.2 Nonclinical Experience with Fenebrutinib

Fenebrutinib (also referred to as GDC-0853 or RO7010939) is a highly selective, orally administered, reversible inhibitor of BTK that is being developed by Genentech, Inc. as a potential therapeutic for autoimmune diseases, including CSU. Fenebrutinib has undergone extensive investigation in nonclinical in vitro and in vivo studies to characterize its pharmacological, metabolic, and toxicological properties (see the Fenebrutinib Investigator's Brochure for further details).

In vitro cell-based experiments suggest that antagonism of BTK leads to inhibition of BCR-dependent B-cell proliferation and a reduction of inflammatory cytokine production from myeloid cells (including tumor necrosis factor alpha [TNF-α], interleukin 1 [IL-1], and interleukin 6 [IL-6]) by preventing signaling through the FcγRIII receptor (Di Paolo et al. 2011; Liu et al. 2011). Fenebrutinib effectively blocks BCR- and CD40-mediated activation and proliferation of B cells. BTK in B cells also plays a role in TLR4-mediated B-cell proliferation and class switching. In monocytes, fenebrutinib inhibits TLR4- and immune complex-mediated inflammatory cytokine production, including TNF-α, which contributes to disease pathogenesis in rheumatoid arthritis (RA).

As described above, the pathophysiology of CSU is not completely understood. A key step is FcεRI-activation and release of histamine and inflammatory cytokines from mast cells and basophils, leading to the wheal and flare formation of a hive as well as angioedema. In support of the importance of BTK in FcεRI signaling and the pathogenesis of CSU, BTK null mice have impaired FcεRI signaling, resulting in decreased histamine and inflammatory cytokine release (Hata et al. 1998; Iyer et al. 2011).

Consistent with these findings, in vitro experiments with human mast cell lines demonstrated that fenebrutinib could effectively inhibit the release of histamine induced by cross-linking of IgE bound to FcεRI on the surface of mast cells. In addition, in a Phase Ib study in healthy volunteers, oral administration of fenebrutinib inhibited ex vivo basophil activation as measured by diminished cell surface expression of CD63 after cross-linking of IgE. As such, fenebrutinib inhibits the activity of two specific cell types that play key roles in disease pathology in CSU.

The fenebrutinib safety profile has been assessed in repeat-dose, general toxicology studies (once a day [QD] oral dosing) ranging from 1 week to 9 months in rats and dogs; in vitro and in vivo genetic toxicology studies; in vitro phototoxicity evaluation; in vitro and in vivo safety pharmacology studies of the central nervous, respiratory, and cardiovascular systems; and embryo-fetal development (Seg II) studies in rats and rabbits. Overall, fenebrutinib was well tolerated for up to 6 months in rats (up to 104 μM • hr) and up to 9 months in dogs (up to 36 μM • hr). Notable findings identified in nonclinical toxicology studies include vascular inflammation (≥ 56 μM • hr) in dogs, hepatotoxicity (180 μM • hr) in dogs and rats, and a minimal increase in corrected QT interval (QTc; 7 ms or 3%; extrapolated unbound maximum observed concentration [C_{max}] of 3.17 μM) in dogs. Fetal malformations in rats (at 627 μM • hr) and rabbits (≥ 10.6 μM • hr) warrant the continued use of highly effective contraception in clinical trials. On the basis of the nonclinical and clinical safety data to date, fenebrutinib is expected to be well tolerated at the doses and duration administered in the current study.

1.2.3 Clinical Experience with Fenebrutinib

As of 12 March 2018, fenebrutinib or placebo has been administered to 1099 subjects (i.e., 333 healthy subjects, 24 patients with hematological malignancies, 576 patients with rheumatoid arthritis, 129 patients with systemic lupus erythematosus, and 37 patients with chronic spontaneous urticaria) at doses with a range of 0.5–600 mg and has been generally well tolerated with no safety concerns that have led to a change in the conduct of the studies.

Study GO29089 is a Phase I, open-label study in which fenebrutinib has been evaluated in patients with relapsed or refractory B-cell non-Hodgkin's lymphoma or chronic lymphocytic leukemia. In order to focus on the autoimmune indications, Genentech elected to stop development of fenebrutinib in oncology, and the Phase I study is continuing without further patient enrollment. Enrollment was stopped after completion of the 400-mg dose level at which time 24 patients had been enrolled in 3 cohorts: 100 mg, 200 mg, and 400 mg fenebrutinib daily. Seven patients remain in the study, and all have undergone intrapatient dose escalation to 400 mg QD fenebrutinib. The mean duration of daily dosing for these 7 patients has been 21 months (range of 18 – 23 months). Fenebrutinib was well tolerated with no dose-limiting toxicities (DLTs), maximum tolerated dose was not reached, and adverse events have been generally non-serious National Cancer Institute Common Terminology Criteria for Adverse Events, Version 4.0 (NCI CTCAE v4.0) Grade 1 or Grade 2 events that have been clinically manageable. The adverse events regardless of causality reported in ≥ 15% of patients include fatigue, nausea, diarrhea, headache, abdominal pain, dizziness, cough, and thrombocytopenia. As of August 2016, 11 serious adverse events had been reported in 5 patients, of whom 2 had a fatal outcome (i.e., complications of H1N1 influenza and influenza pneumonia).

Study GP29318 was a two-part, single-ascending dose (SAD) study to assess the safety, tolerability, and pharmacokinetics of fenebrutinib administered to 93 healthy subjects. In Part 1, the single-dose–escalation portion, 71 subjects were randomized to panels of 8 subjects (6:2 active:placebo ratio) per dose group (0.5 – 600 mg), with 53 subjects receiving active fenebrutinib. In Part 2, 100 mg fenebrutinib was administered to 40 subjects in the open-label food and pilot rabeprazole effect study. There were no serious adverse events and no withdrawals due to adverse events during the conduct of Study GP29318. In Part 1 of the study, there were no dose-limiting adverse events (DLAEs) at single doses up to 600 mg fenebrutinib. All adverse events were mild in intensity (Grade 1; Toxicity Grading Scale for Healthy Adult and Adolescent Volunteers Enrolled in Preventive Vaccine Clinical Trials) and transient. No adverse events increased in intensity or frequency with dose escalation. There were two treatment-emergent adverse events of mild self-limited headache reported as related to fenebrutinib administration. There were no trends in safety laboratory findings, vital sign changes, physical examination findings, or ECG changes. There were no trends in hepatic laboratory changes following single doses of fenebrutinib in healthy subjects. Administration of fenebrutinib inhibited ex vivo basophil activation as demonstrated by diminished CD63 cell surface expression after cross-linking of IgE. Refer to the Fenebrutinib Investigator's Brochure for further information on Study GP29318, including pharmacokinetics.

Study GA29347 was a multiple-ascending dose (MAD) study to assess the safety, tolerability, and pharmacokinetics of multiple doses of fenebrutinib administered to 30 healthy subjects for 14 days. Forty subjects were randomized to panels of 8 subjects (6:2 active:placebo) per dose group, at doses of 20 mg twice a day (BID), 60 mg BID, 150 mg BID, 250 mg BID, or 500 mg QD for 14 days, with 30 subjects receiving active fenebrutinib. The study drug was well tolerated. There were no serious adverse events and no withdrawals due to adverse events during the conduct of the study. All adverse events were mild in intensity (Grade 1) and transient, with no relationship to dose. Adverse events included skin reactions (i.e., rash, contact dermatitis, and skin irritation from ECG leads), nausea, headache, insomnia, toothache, tinnitus, and asymptomatic bacteriuria. There were no trends in safety laboratory, vital sign, physical examination, or ECG findings. Similar to the SAD study, dose-dependent inhibition of CD63 expression was observed following fenebrutinib administration, with sustained inhibition over the duration of dosing.

Study GP29832 was a relative bioavailability study designed to evaluate the effects of formulation, food, and proton-pump inhibitor (PPI) or methotrexate co-administration on the pharmacokinetics of fenebrutinib in healthy subjects. Fenebrutinib was well tolerated when administered to 48 healthy subjects at the 200-mg dose level.

Study GA29350 is a multicenter Phase II dose ranging study comparing the efficacy and safety of fenebrutinib versus placebo and adalimumab in patients with RA who have had an inadequate response to previous methotrexate therapy and versus placebo in

patients with an inadequate response to previous tumor necrosis factor therapy. The study began enrollment in September 2016, and the total planned enrollment is approximately 580 patients.

Study GA30044 is the first study investigating fenebrutinib in systemic lupus erythematosus (SLE). This is a multicenter, Phase II, randomized, double-blind, placebo-controlled, parallel-group, dose-ranging study designed to evaluate the efficacy and safety of fenebrutinib in patients with moderate-to-severe active SLE in combination with standard-of-care therapy.

The drug-drug interaction Study GP39616 has recently concluded. While final results are not yet available, preliminary data suggests:

- Fenebrutinib is a Breast Cancer Resistance Protein (BCRP) inhibitor. Consequently, fenebrutinib may alter transport of BCRP substrates and result in increased plasma concentrations of BCRP substrates.

The study also confirmed the following:

- Fenebrutinib is a mild inhibitor of CYP3A at clinically relevant doses. Consequently, fenebrutinib may alter metabolism of CYP3A substrates and result in increased plasma concentrations of CYP3A substrates.
- Fenebrutinib is a moderately sensitive substrate of CYP3A at clinically relevant doses. There is a moderate potential for levels of fenebrutinib to increase in patients taking concomitant medications that inhibit CYP3A and decrease in patients taking medications that induce CYP3A.

Refer to the Fenebrutinib Investigator's Brochure for detailed background information on fenebrutinib as well as for additional details on nonclinical and clinical studies.

1.3 STUDY RATIONALE AND BENEFIT-RISK ASSESSMENT

Omalizumab, an anti-IgE monoclonal antibody, has demonstrated efficacy in treating patients with CSU, highlighting the key role of IgE in CSU pathogenesis. Inhibition of IgE-mediated FcεRI signaling by BTK inhibition offers a promising mechanism for the treatment of CSU. In preclinical in vitro studies, BTK inhibition was able to prevent the release of histamine and inflammatory cytokines from mast cells after FcεRI engagement and cross-linking. In addition, in the healthy volunteer SAD and MAD studies, oral administration of fenebrutinib was able to inhibit ex vivo basophil activation as demonstrated by diminished CD63 cell surface expression. As such, fenebrutinib inhibits two key pathogenic cell types in CSU. The aim of this study is to determine if fenebrutinib can effectively treat patients with CSU, as measured by reduction in disease activity.

Humans with XLA lack functional BTK but can live relatively normal lives on a standard therapy of IV immunoglobulin (Kaveri et al. 2011), suggesting that BTK can be safely inhibited in patients with CSU who have functional immune systems. Clinical experience

with fenebrutinib to date has not generated safety concerns that would preclude further evaluation in patients with CSU. In the SAD (Study GP29318), MAD (Study GA29347), relative bioavailability (Study GP29832), and oncology (Study GO29089) studies, fenebrutinib was well tolerated with no DLAEs or DLTs. In the oncology study, there were 2 deaths due to complications of confirmed influenza (i.e., H1N1 influenza and influenza pneumonia).

Several measures will be taken to ensure the safety of patients participating in this study based on the potential risks for fenebrutinib based on nonclinical and clinical studies and published literature (see Section 5.1.1). Eligibility criteria have been designed to exclude patients at higher risk for potential toxicities. In addition, the sites selected for this study will be specialty dermatology and immunology clinics with experience in treating CSU.

1.3.1 Infections

Fenebrutinib is a targeted immunomodulator; however, as a reversible inhibitor, the degree to which fenebrutinib antagonism of BTK signaling may suppress immune activity is unknown. Patients participating in this study may be at risk for infections, including opportunistic infections. Therefore, patients at high risk for infection will be excluded (see Section 5.1.1.1). Patients will be carefully monitored throughout the study for infections. Fenebrutinib will be discontinued in any patient who develops a serious infection, opportunistic infection, or any infection requiring treatment with an IV antimicrobial agent.

1.3.2 Bleeding

BTK is expressed in platelets and is involved in platelet function via GPVI/collagen receptor signaling and GP1b receptor signaling. Platelets from patients with XLA demonstrate decreased activation in response to submaximal collagen stimulation but normal response to thrombin; clinically, there is no reported bleeding propensity in patients with XLA. In the fenebrutinib clinical study involving oncology patients (GO29089), 2 patients experienced Grade ≥ 3 gastrointestinal (GI) bleeding. These events were not dose related and occurred in patients on non-steroidal anti-inflammatory drugs (NSAIDs)/acetylsalicylic acid with a history of gastroesophageal or peptic ulcer disease.

It is unknown whether fenebrutinib will increase the risk of bleeding in patients with CSU who receive antiplatelet or anticoagulant therapies. Therefore, the eligibility criteria exclude patients at high risk for bleeding complications.

1.3.3 Cytopenias

Neutropenia, anemia, and thrombocytopenia have been observed in patients with hematologic malignancies who received fenebrutinib. No clinically significant changes in cell counts were observed in the healthy volunteer studies. Events have been monitorable and clinically manageable. Cell counts will be monitored regularly throughout the study.

1.3.4 Hepatotoxicity

Evidence of hepatobiliary injury was observed in animals administered relatively high doses of fenebrutinib in repeat-dose toxicity studies. In clinical studies to date, in autoimmune indications, enrolling over 800 patients, multiple cases of treatment emergent Grade 3 (or severe) elevations of ALT, some of which were considered serious adverse events, have been observed in the randomized clinical studies, which remain blinded to the Sponsor in terms of treatment assignment, as well as in open label extensions of fenebrutinib. These cases have been seen in blinded studies in CSU. None of the cases of transaminase elevations resulted in clinical jaundice or bilirubin $>2\times$ ULN (Hy's Law). All transaminase elevations have been reversible when dosing of blinded study medication/placebo was withheld. These findings have not been seen in single dose and multiple dosing for 14 days in healthy subjects and QD dosing in patients with hematological malignancies. To minimize this risk, exclusion criteria have been defined for abnormal liver enzyme and function tests and current liver disease (see Section 4.1.2). For further information on nonclinical and clinical findings of hepatotoxicity, please see the Fenebrutinib Investigator's Brochure.

1.3.5 Cardiovascular Effects

Fenebrutinib is considered to have a low potential to cause QT interval prolongation or to directly affect other cardiovascular parameters at therapeutic exposures. A minimal increase in corrected QT (QTc; 7 ms or 3%) interval was noted at 45 mg/kg in the single-dose cardiovascular safety pharmacology study in telemetry-instrumented dogs. Cardiac safety will be evaluated in all patients at baseline and throughout the study, with routine monitoring of vital signs, including heart rate and blood pressure, collection of ECGs, and reporting of cardiac adverse events.

1.3.6 Malignancy

The impact of BTK inhibition on the development of malignancies is not known; however, malignancies are considered a potential concern for all immunomodulatory agents. Patients with a history of cancer within 10 years of screening will be excluded from study participation, except for basal or squamous cell carcinoma of the skin that has been excised and is considered cured and in situ carcinoma of the cervix treated with apparent success by curative therapy more than 1 year prior to screening.

Overall, fenebrutinib has been well tolerated in Phase I healthy subjects and an oncology study. On the basis of the compelling mechanism for BTK inhibition in CSU,

the benefit-risk ratio for this study is deemed acceptable. The safety profile of fenebrutinib will be further characterized in this Phase II study. A robust safety monitoring plan that describes the potential risks for fenebrutinib and the risk-mitigation strategies to minimize risks for the patients in this trial is provided in Section 5.

Please refer to the most recent Fenebrutinib Investigator's Brochure for additional details on clinical and nonclinical studies and additional safety information.

2. OBJECTIVES AND ENDPOINTS

This pilot and dose-ranging study will evaluate the efficacy, safety, and pharmacokinetics of fenebrutinib compared with placebo in patients with CSU refractory to anti-histamines (up to 4 times the approved dose per local treatment guidelines). Specific objectives and corresponding endpoints for the study are outlined in Table 1.

Table 1 Objectives and Corresponding Endpoints

Objectives	Corresponding Endpoints
**Efficacy Objective:**	
• To evaluate the efficacy of fenebrutinib compared with placebo in patients with CSU who are refractory to anti-histamines	**Primary Endpoint:**<br>• Change from baseline in the UAS7 at Day 57 (Week 8)<br>**Secondary Endpoints:**<br>• Proportion of patients who are well controlled (UAS7 $\leq$6) at Day 57<br>• Change from baseline in the UAS7 at Day 29 (Week 4)<br>**Exploratory Endpoint:**<br>• Change from baseline in the weekly itch score at Day 29<br>• Change from baseline in the weekly itch score at Day 57<br>• Change from baseline in the weekly hives score at Day 57<br>• Proportion of patients who are well controlled (UAS7 $\leq$6) at Day 29<br>• Proportion of patients who achieve complete response (UAS7 = 0) at Day 29<br>• Proportion of patients who achieve complete response (UAS7 = 0) at Day 57<br>• Proportion of patients achieving MID in UAS7 at Day 57 (reduction from baseline $\geq$11 points)<br>• Proportion of patients achieving MID in the weekly itch score at Day 57 (reduction from baseline $\geq$5 points)<br>• Time to achieving MID in UAS7 (reduction from baseline $\geq$11 points)<br>[redacted]

Table 1 Objectives and Corresponding Endpoints (cont.)

Objectives	Corresponding Endpoints
**Safety Objective:**	
• To evaluate the safety of fenebrutinib compared with placebo	• The nature, frequency, timing, and severity of adverse events<br>• Change from baseline in targeted vital signs, physical examination findings, ECGs, and clinical laboratory results following fenebrutinib administration
**Pharmacokinetic Objective:**	
• To characterize the pharmacokinetics of fenebrutinib in patients	• Plasma concentrations of fenebrutinib at specified timepoints
[redacted]	
[redacted]	[redacted]
[redacted]	[redacted]
[redacted]	[redacted]
[redacted]	
[redacted]	[redacted]

$AUC_{0\text{-}t}$ = area under the concentration–time curve from time 0 to time t; [redacted] CL/F = apparent clearance; C_{max} = maximum concentration observed; C_{trough} = steady-state concentration at the end of a dosing interval; CSU = chronic spontaneous urticaria; Ig = immunoglobulin; MID = minimally important difference; [redacted] PK = pharmacokinetic; $t_{1/2}$ = half-life; t_{max} = time to maximum concentration; UAS7 = Urticaria Activity Score over 7 days; [redacted]

3. STUDY DESIGN

3.1 DESCRIPTION OF THE STUDY

This pilot and dose-ranging study is a multicenter, randomized, double-blind, placebo-controlled, parallel-group study of the efficacy and safety of fenebrutinib as

add-on therapy for the treatment of adult patients 18 – 75 years old who have been diagnosed with CSU and who remain symptomatic despite treatment with H1 antihistamines (including doses up to 4 times the approved dose per local treatment guidelines). The study will consist of two cohorts. Cohort 1 will enroll approximately 45 patients across multiple sites. After screening, eligible patients will be randomly allocated in a 2:1 ratio to receive fenebrutinib 200 mg orally (PO) twice daily (BID) or matching placebo for 8 weeks and will maintain stable doses of standard-of-care H1 antihistamine therapy throughout the study. On the basis of results from an interim analysis of Cohort 1, a dose-ranging cohort, Cohort 2, will be opened and approximately 120 patients will be randomly allocated in a 1:1:1:1 ratio to receive 50 mg PO daily (QD), 150 mg PO QD, 200 mg PO BID of fenebrutinib, or placebo, respectively, for 8 weeks and will maintain stable doses of standard-of-care H1 antihistamine therapy (background therapy) throughout the study.

Both cohorts will consist of 3 distinct study periods over a time-period of 14 weeks as outlined below (see Figure 1):

- Screening period: Day –14 to Day –1
- Treatment Period: Day 1 to Day 57 (Week 0 to Week 8)
- Follow-Up Period: Day 57 to Day 85 (Week 8 to Week 12)

Patients in both cohorts will have a screening period of approximately 2 weeks to establish their eligibility for the study and baseline symptom scores. For the duration of the screening period, patients must maintain stable doses of their pre-screening combination therapy with standard-of-care H1 antihistamines (i.e., up to 4 times the approved dose per local treatment guidelines). The screening period will consist of visits at Day –14 and Day –7. Patients must meet all of the following criteria to enter the screening period:

- Documented treatment with a regimen that includes standard-of-care H1 antihistamine for CSU at Day –14 and for at least the 3 consecutive days immediately prior to Day –14 (see Section 4.4.1 for list of H1 antihistamines available for use in this study)
- Willing and able to complete a symptom electronic diary (Urticaria Patient Daily eDiary) twice daily throughout the screening period to establish the patient's Urticaria Activity Score over 7 days (UAS7) score.

To be eligible for randomization in both cohorts, for the 7 days prior to randomization, patients must meet all of the following:

- Seven consecutive days of entries in the Urticaria Patient Daily eDiary, and
- UAS7 symptom score of $\geq$ 16 (range: 0 – 42)

Only in exceptional circumstances, when information concerning eligibility is outstanding (e.g., delayed laboratory results), will a longer screening period be permitted up to

3 business days. Upon approval from the Medical Monitor, patients may be re-screened or maybe retested during the screening period (see Section 4.5.2.2 and Section 4.5.2.1, respectively). Circumstances that may permit re-screening or retesting include, but are not limited to, a laboratory test result that does not meet eligibility requirements.

The primary efficacy endpoint will be at Day 57 (Week 8). Throughout the treatment period, patients must maintain stable doses of their pre-randomization H1 antihistamine therapy.

After completion of the 8-week treatment period, patients in both cohorts will enter a 4-week safety follow-up period to allow for further characterization of the pharmacokinetics and pharmacodynamics of fenebrutinib and collection of additional efficacy and safety data. During safety follow-up period, no study treatment will be given; patients must maintain stable doses of their pre-randomization CSU H1 antihistamine treatment (background therapy). In the safety follow-up period, patients may add up to one additional H1 antihistamine therapy in case of worsened symptoms (see Section 4.4.3). The goal of allowing additional H1 antihistamine therapy after the treatment period is to reduce patient dropout for improved safety evaluation.

In addition to their daily background therapy, for the duration of the study, all patients will be able to use a single approved dose of loratadine (10 mg maximum) or cetirizine (10 mg maximum) within a 24-hour period as rescue medication if symptoms worsen. If a patient needs rescue therapy and is already on background treatment with cetirizine or loratadine, the patient may receive 10 mg more of the same drug only if the total daily dose remains below 4 times the approved dose. Otherwise, the alternate rescue medication may be used. Patients should record the use of this medication in their eDiary. Patients receiving PPIs or H2 receptor antagonists (H2RAs) should be stabilized on a regimen beginning at least 2 weeks prior to randomization and continuing throughout the study (see Section 4.4.2.1).

Figure 1 Study Schema

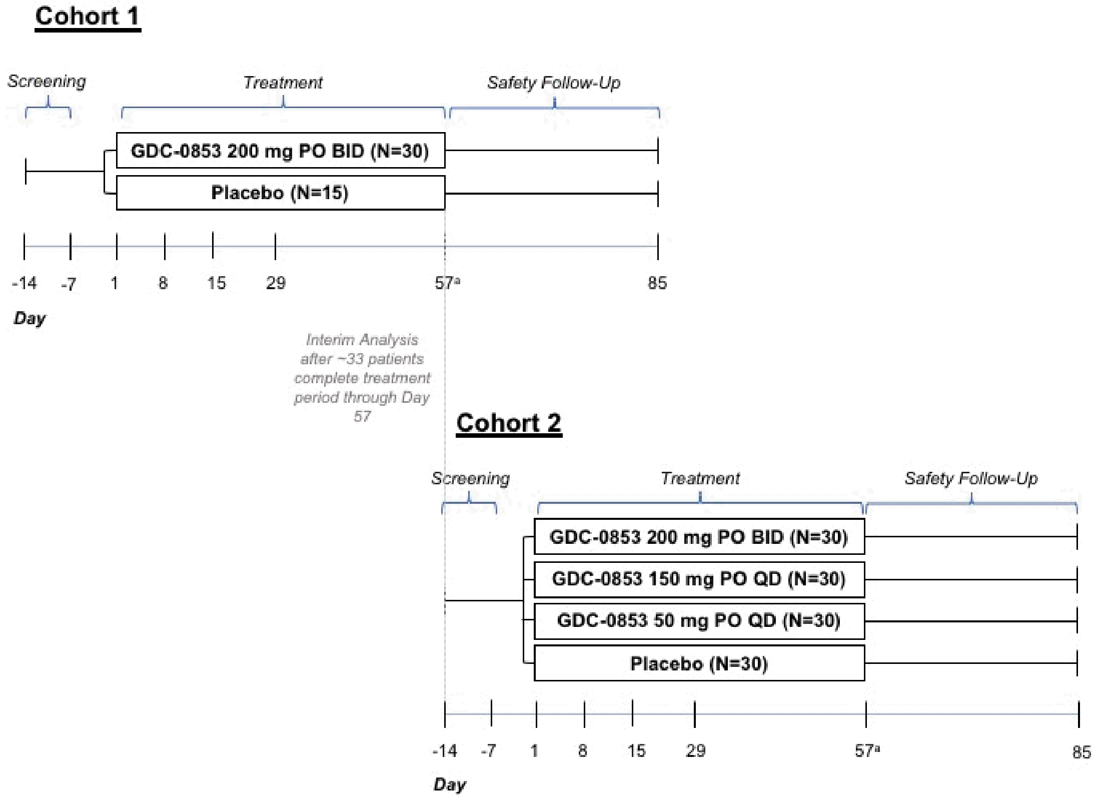

BID = twice daily; PO = orally; QD = daily.
[a] Last blinded dose = p.m. before Day 57 visit

3.1.1 Internal Monitoring Committee

For Cohort 2, periodic safety reviews and any interim analysis will be performed by the Sponsor's internal monitoring committee (IMC) as outlined in the IMC charter. This committee will be unblinded to treatment assignments and will include Sponsor representatives from the following functions: Clinical Science, Drug Safety, Biostatistics, and Statistical Programming and Analysis. The IMC members will not have direct contact with investigational staff or site monitors. The IMC may decide to unblind the study team to enable decision-making and potential interactions with regulatory bodies. The IMC may invite representatives from other functional areas on an ad-hoc basis when additional expertise is required (e.g., Clinical Pharmacology, Research, etc.) or additional Sponsor scientists to participate in data analyses and review.

At any time during the study, the Sponsor may choose to inactivate and suspend enrollment and further dosing for a given treatment arm (in Cohort 2) or reduce the dose due to safety concerns and as guided by the IMC. In Cohort 2, subsequently enrolled patients will be randomly allocated to the remaining active arms.

3.2 END OF STUDY AND LENGTH OF STUDY

The end of this study is defined as the date when all patients have completed the study completion visit or early termination visit or have otherwise been discontinued from the study. The total duration of this study for each subject is approximately 14 weeks (for both cohorts), including screening, treatment, and safety follow-up periods.

The total length of the study, from screening of the first patient to the end of the study, is expected to be approximately 19 months.

3.3 RATIONALE FOR STUDY DESIGN

3.3.1 Rationale for Fenebrutinib Dose and Schedule

In the proposed study for Cohort 1, patients randomly allocated to the fenebrutinib arm will receive 200 mg of fenebrutinib PO BID for 8 weeks. This dose is expected to be well tolerated and to substantially inhibit BTK activity, based on results from the Phase I studies. Dose-dependent target inhibition was demonstrated in the Phase I SAD and MAD studies with use of pharmacodynamic (PD) assays (phospho-BTK and basophil CD63 assays). On the basis of the pharmacokinetic (PK) and PK/PD models constructed using data from the relative bioavailability, SAD, and MAD healthy volunteer studies and of the tolerability of dosing up to 250 mg BID, the 200 mg BID dose is expected to provide a steady-state exposure achieving 90% maximal inhibitory concentration (IC_{90}) over the entire dosing interval in greater than 75% of patients. It should be noted that the extent of target engagement required for clinical efficacy is unknown. However, Cohort 1 is a pilot study, the dose tested in Cohort 1 will enable initial assessment of clinical efficacy in CSU.

Because the extent of target engagement required for clinical efficacy is unknown, doses for Cohort 2 were selected to evaluate a range of target engagement and to characterize the dose- and exposure–response relationships for safety and efficacy in order to select the optimal dose for further study.

The fenebrutinib dose levels proposed for Cohort 2 were selected based on target engagement (phospho-BTK and basophil CD63 assays) observed in the Phase 1 SAD and MAD studies as well as an in vitro assessment of FcεRI-dependent histamine release from LAD-2 mast cells. The rationale for the dose regimens is as follows:

- The 50-mg QD dose is expected to provide systemic exposures that will achieve 70% maximal inhibitory concentration (IC_{70}) based on the basophil CD63 assay for half of the dosing interval in approximately 50% of patients.
- The 150-mg QD dose is expected provide an exposure achieving IC_{70} based on the basophil CD63 assay for half of the dosing interval in approximately 90% of patients.

- The 200-mg BID dose is the Proof of Concept dose carried forward from Cohort 1. This dose is expected to provide a steady-state exposure achieving 90% maximal inhibitory concentration (IC_{90}) based on the basophil CD63 assay and approximately 80% maximal inhibitory concentration (IC_{80}) based on the LAD-2 mast cell FcεRI-dependent histamine release assay over the entire dosing interval in the majority of patients.

A fumaric acid tablet formulation of fenebrutinib will be used in this study. In the relative bioavailability Study GP29832, the tablet formulation decreased PK variability and appeared to improve absorption by mitigating pH-dependent solubility compared with the powder-in-capsule formulation. The proposed highest dose in both cohorts, 200 mg BID (fumaric acid tablet), is expected to achieve exposures (area under the concentration–time curve [AUC] and C_{max}) that are in the range of those observed following administration of 250 mg BID (powder-in-capsule) in Study GA29347 and 5-fold below the exposures at the no observed adverse effect level (NOAEL) of the nonclinical chronic toxicity studies (see the Fenebrutinib Investigator's Brochure).

Study GP29832 also characterized the effect of food and the effect of the PPI rabeprazole on the pharmacokinetics of fenebrutinib. A moderate-fat meal did not substantially change the systemic exposure following a single administration of 200 mg fenebrutinib, since the fumaric acid tablet formulation (fed:fasted geometric mean ratio 90% CI) for area under the concentration–time curve from time 0 to 24 hours (AUC_{0-24}) and C_{max} were 1.08 (0.97–1.22) and 0.85 (0.72–1.01), respectively, supporting administration without regard to food. Co-administration of multiple doses of rabeprazole with a single dose of 200 mg fenebrutinib with the fumaric acid tablet formulation decreased fenebrutinib systemic exposure (AUC_{0-24}) by approximately 32% and 44% in the fasted and fed states, respectively, compared with tablet alone. In order to evaluate PK in a relevant patient population, stable use of a PPI regimen will be permitted during the study.

3.3.2 Rationale for Patient Population

Patients enrolled in both cohorts of the study will have a CSU diagnosis for $\geq$ 6 months and will be refractory to treatment with a combination of H1 antihistamines consistent with standard of care (i.e., up to 4 times the approved dose per local treatment guidelines) as demonstrated by the presence of itch and hives for $>$6 consecutive weeks on this treatment at any time prior to enrollment. In addition, patients will have a UAS7 score of $\geq$16 during the 7 days prior to randomization despite current use of combination therapy.

While H1 antihistamines are the mainstay of therapy for CSU, some patients do not respond or respond only partially to these therapies, and these patients tend to experience more severe disease. This patient population was selected for this study because of the unmet medical need for more effective oral treatments.

3.3.3 Rationale for Control Group

A placebo-treated control group is required for this study in order to achieve its efficacy and safety objectives given the inherent variability in symptoms and the different rates of improvement in the placebo arms of prior studies. Patients in the placebo arm will continue to receive stable standard-of-care H1 *antihistamine* therapy throughout the study. In addition, the study will allow for rescue medications for persistent symptoms.

[redacted]

3.3.5 Rationale for Pharmacokinetic Sample Collection Schedule

The sampling schedule is designed to assess multiple pre-dose (prior to study drug administration in clinic) plasma fenebrutinib concentrations, which will enable the estimation of systemic fenebrutinib exposures and subsequent exposure-response analyses, both of which may be reported separately. Results will be used to inform dosing regimens for future studies of fenebrutinib.

3.3.6 Rationale for Efficacy Endpoints

The change in the UAS7 (see Table 2 for daily assessment of Urticaria Activity Score [UAS]) has been chosen as the primary efficacy endpoint as it has been used in pivotal trials in CSU to measure reduction in CSU disease severity. The UAS7 is a summation of the average daily (a.m./p.m.) scores on the UAS (range: 0 – 6), which is a composite diary score with numeric severity intensity ratings on a scale of 0–3 (0 = none to 3 = intense/severe) for two domains: the intensity of the itch and the number of wheals/hives (see Table 2). The UAS will be recorded by the patient twice daily (morning and evening) in the patient Urticaria Patient Daily eDiary. UAS7 scores range from 0 – 42 and the minimally important difference (MID) is considered to be a reduction from baseline of ≥ 9.5 to 10.5 points (Mathias et al. 2012). The baseline UAS7 is the sum of the daily scores on the UAS over the 7 days prior to randomization (Day 1 visit for both cohorts), and the UAS7 at Day 57 is the sum of daily scores on the UAS over the 7 days prior to the Day 57 visit (for both cohorts). The same principles of calculating baseline and Day 57 weekly scores will be applied to the other weekly outcomes unless otherwise stated.

The kinetics of response to fenebrutinib will also be carefully evaluated throughout the course of the study at regular intervals (at least every 1 – 2 weeks for a period of 8 weeks). In addition, disease recurrence or duration of treatment benefit after study drug is withdrawn during the safety follow-up period will be measured during this study. This will provide initial guidance for the duration of therapy in future studies.

Table 2 Twice Daily Patient Assessment of CSU Disease Activity (UAS Scale)

Score	Wheals (Hives)	Pruritus (Itch)
0	None	None
1	Mild (1–6 hives/12 hour)	Mild
2	Moderate (7–12 hives/12 hour)	Moderate
3	Intense (>12 hives/12 hour)	Severe

CSU = chronic spontaneous urticaria; UAS = Urticaria Activity Score.

4. MATERIALS AND METHODS

4.1 PATIENTS

Approximately 45 patients in Cohort 1 and approximately 120 patients in Cohort 2, aged 18 to 75 years old who have been diagnosed with refractory CSU and who remain symptomatic despite standard-of-care H1 antihistamine therapy (i.e., up to 4 times the approved dose per local treatment guidelines), will be enrolled in this study.

4.1.1 Inclusion Criteria

Patients in Cohort 1 and 2 must meet the following criteria for study entry:

- Willing to give written informed consent, adhere to the visit schedules, comply with the study drug regimen, and meet other study requirements
- Aged 18–75 years, inclusive
- Diagnosis of CSU refractory to H1 antihistamines at the time of randomization, as defined by all of the following:
  - The presence of itch and hives for > 6 consecutive weeks at any time prior to enrollment despite current use of H1 antihistamines, consistent with standard of care (i.e., up to 4 times the approved dose per local treatment guidelines) during this time period
  - UAS7 score ≥ 16 during the 7 days prior to randomization (Day 1)
  - Patients must have been on daily stable doses of H1 antihistamines, consistent with standard-of-care therapy (i.e., up to 4 times the approved dose per local treatment guidelines) for CSU starting at least 3 consecutive days immediately prior to the screening visit through Day 1 and must document current use on all visits.
  - CSU diagnosis for ≥ 6 months
- Willing and able to complete an Urticaria Patient Daily eDiary for the duration of the study
- Completion of 7 days of the Urticaria Patient Daily eDiary entries in the 7 days prior to randomization (7 of 7 days must be completed [i.e., must complete an entry every day] with up to 2 non-consecutive entries missed)

- No evidence of active or latent or inadequately treated infection with tuberculosis (TB) as defined by the following:
  - A negative QuantiFERON-TB-Gold® (QFT) performed (for German sites only: QFT is the preferred test) at the screening visit or within the 3 months prior to screening

    If QFT is unavailable, a negative Mantoux purified protein derivative (PPD) skin test as defined by the Centers for Disease Control and Prevention guidelines, may be performed at the screening visit or within the 3 months prior to screening -AND-

    Any additional procedures (e.g., chest X-Ray) only if required per local guidelines/standard of care to rule out latent or active TB

  NOTE: A documented negative screening for TB via the PPD test or a negative QFT within 3 months prior to screening and if required per local standard of care, a chest X-ray, is sufficient and no further screening with QFT is required.

  Patients with a history of Bacille Calmette-Guérin (BCG) vaccination should be screened using the QFT test, only.

  - An indeterminate QFT test should be repeated.
  - A positive QFT test or two successive indeterminate QFT results should be considered a positive diagnostic TB test.
  - An indeterminate QFT test followed by a negative QFT test should be considered a negative diagnostic TB test.
- Only for patients currently receiving PPIs or H2RAs: Treatment must be at a stable dose during the 2-week screening period prior to randomization and with a plan to remain at a stable dose for the duration of the study.
- For women of childbearing potential: Agreement to remain abstinent (refrain from heterosexual intercourse) or use contraceptive methods that result in a failure rate of $<$1% per year during the treatment period and for at least 4 weeks after the last dose of study drug (see Section 5.4.3.1). Women must refrain from donating eggs during this same period.
  - A woman is considered to be of childbearing potential if she is postmenarcheal, has not reached a postmenopausal state ($\geq$12 continuous months of amenorrhea with no identified cause other than menopause), and has not undergone surgical sterilization (removal of ovaries and/or uterus).
  - Examples of contraceptive methods with a failure rate of $<$1% per year include bilateral tubal ligation, male sterilization, established proper use of hormonal contraceptives that inhibit ovulation, hormone-releasing intrauterine devices, and copper intrauterine devices. Women using estrogen-containing hormonal contraceptives as a method of contraception must also use a barrier, such as a male condom, in conjunction with the hormonal contraceptives.

  - The reliability of sexual abstinence should be evaluated in relation to the duration of the clinical trial and the preferred and usual lifestyle of the patient. Periodic abstinence (e.g., calendar, ovulation, symptothermal, or post-ovulation methods) and withdrawal are not acceptable methods of contraception.

- For men: agreement to remain abstinent (refrain from heterosexual intercourse) or use contraceptive measures and agreement to refrain from donating sperm, as defined below (also see Section 5.4.3.2):
  - With female partners of childbearing potential or pregnant female partners, men must remain abstinent or use a condom during the treatment period and for at least 4 weeks after the last dose of study treatment to avoid exposing the embryo. Men must refrain from donating sperm during this same period.
  - The reliability of sexual abstinence should be evaluated in relation to the duration of the clinical trial and the preferred and usual lifestyle of the patient. Periodic abstinence (e.g., calendar, ovulation, symptothermal, or post-ovulation methods) and withdrawal are not acceptable methods of contraception.

4.1.2 Exclusion Criteria

Patients in Cohort 1 and 2 who meet any of the following criteria will be excluded from study entry:

- Treatment with omalizumab or other monoclonal antibody therapies used to treat CSU within 4 months prior to screening or primary nonresponse to omalizumab
- Use of a non-biologic investigational drug or participation in an investigational study with a non-biologic drug within 30 days prior to study drug administration on Day 1 (or within 5 half-lives of the investigational product, whichever is greater)
- Use of a biologic investigational therapy or participation in an investigational study involving biologic therapy within 90 days or 5 half-lives, whichever is greater, prior to study drug administration on Day 1
- Previous treatment with fenebrutinib or other BTK inhibitors
- Patients whose urticaria is solely due to physical urticaria
- Other diseases with symptoms of urticaria or angioedema, including urticarial vasculitis, urticaria pigmentosa, erythema multiforme, mastocytosis, hereditary or acquired angioedema, lymphoma, or leukemia
- Atopic dermatitis, bullous pemphigoid, dermatitis herpetiformis, or other skin disease associated with itch such as psoriasis
- Routine (daily or every other day during 5 or more consecutive days) doses of the following medications within 30 days prior to screening: systemic or cutaneous (topical) corticosteroids (prescription or over the counter), hydroxychloroquine, methotrexate, cyclosporine, or cyclophosphamide
- Prior utilization of IV steroids for treatment of laryngeal angioedema
- IVIG or plasmapheresis within 30 days prior to screening
- History of anaphylactic shock without clearly identifiable avoidable antigen (e.g., due to food allergy)

- Hypersensitivity to fenebrutinib or any component of the formulation
- Major surgery within 8 weeks prior to screening or surgery planned prior to end of study (12 weeks after randomization)
- Require any prohibited concomitant medications (see Section 4.4.3)
- History of live attenuated vaccine within 6 weeks prior to randomization or requirement to receive these vaccinations at any time during study drug treatment
    - Seasonal influenza and H1N1 vaccination is permitted if the inactivated vaccine formulation is administered.
- Evidence of clinically significant cardiac, neurologic, psychiatric, pulmonary, renal, hepatic, endocrine (including uncontrolled diabetes mellitus), metabolic, or GI disease that, in the investigator's opinion, would compromise the safety of the patient, interfere with the interpretation of the study results or otherwise preclude patient participation
    - Any items that are cause for uncertainty must be reviewed with the Medical Monitor.
- Uncontrolled disease states, such as asthma, psoriasis, or inflammatory bowel disease, where flares are commonly treated with oral or parenteral corticosteroids
- History of vasculitis
- Current liver disease
- Any known active infection (with the exception of fungal nail infections or oral herpes)
- History of recurrent bacterial, viral, mycobacterial or fungal infections (defined as $>$2 similar episodes requiring anti-microbial treatment within the previous 12 months), with the exception of recurrent oral or genital herpes (herpes simplex virus 1/herpes simplex virus 2) or uncomplicated urinary tract infections in females.
- Any history of opportunistic infections that, in the investigator or Sponsor's judgment, would raise safety concerns regarding the patient's participation in the study
- Any major episode of infection requiring hospitalization or treatment with IV antimicrobials within 8 weeks prior to and during screening or treatment with oral antimicrobials within 2 weeks prior to and during screening
    - Antimicrobials include antifungal, antibacterial, and antiviral agents.
- History of or currently active primary or secondary immunodeficiency, including known history of HIV infection
- Evidence of chronic and/or active hepatitis B or C
    - Positive hepatitis B surface antigen (HBsAg) or hepatitis C serology (regardless of treatment status)
    - Positive hepatitis B core antibody (HBcAb)

- History of cancer, including hematologic malignancy and solid tumors, within 10 years before screening
    - Basal or squamous cell carcinoma of the skin that has been excised and is considered cured and in situ carcinoma of the cervix treated with apparent success by curative therapy > 1 year prior to screening are not exclusionary.
- Women who are pregnant, nursing (breastfeeding), or intending to become pregnant during the study or within 4 weeks after completion of the study
- For women of childbearing potential (including those who have had a tubal ligation): positive serum pregnancy test result at screening or on Day 1.
    - A serum pregnancy test is needed on Day 1 only if the urine pregnancy test is positive (see Section 4.1.1 for definition of "childbearing potential").
- History of alcohol, drug (e.g., tetrahydrocannabinol [THC], marijuana), or chemical abuse within the 12 months prior to screening as determined by the investigator
- Need for systemic anti-coagulation with warfarin, other oral or injectable anti-coagulants, or anti-platelet agents other than NSAIDs, aspirin, and other salicylates
- History of non-gallstone–related pancreatitis or chronic pancreatitis
- History of hospitalizations or transfusion for a GI bleed
- History of cerebrovascular accident (CVA) within 10 years or any history of hemorrhagic CVA
- History of spontaneous intracranial hemorrhage or history of traumatic intracranial hemorrhage within 10 years
- Known bleeding diathesis
- Screening 12-lead ECG that demonstrates clinically relevant abnormalities that may affect patient safety or interpretation of study results, including
    - QT interval corrected using Fridericia's formula (QTcF) > 440 ms demonstrated by at least two ECGs > 30 minutes apart
- History of ventricular dysrhythmias or risk factors for ventricular dysrhythmias such as long QT syndrome and other genetic risk factors (e.g., Brugada syndrome), structural heart disease (e.g., severe left ventricular systolic dysfunction, severe left ventricular hypertrophy), coronary heart disease (symptomatic or with ischemia demonstrated by diagnostic testing, prior coronary artery bypass grafting, or coronary lesions > 70% diameter stenosis that have not been or cannot be re-vascularized), clinically significant electrolyte abnormalities (e.g., hypokalemia, hypomagnesemia, hypocalcemia), or family history of sudden unexplained death or cardiac ion channel mutations (e.g., congenital long QT syndrome)
- Current treatment with medications that are well known to prolong the QT interval (see https://crediblemeds.org/index.php/login/dlcheck) at doses that have a clinically meaningful effect on QT, as determined by the investigator; the investigator may contact the Sponsor for confirmation if needed

- Current treatment with astemizole, terfenadine, and/or ebastine
- Any condition possibly affecting oral drug absorption (e.g., gastrectomy, clinically significant diabetic gastroenteropathy, or certain types of bariatric surgery such as gastric bypass)
  - Procedures such as gastric banding, that simply divide the stomach into separate chambers, are not exclusionary.
- Any uncontrolled clinically significant laboratory abnormality that would affect safety, interpretation of study data, or the patient's participation in the study

The following exclusion criteria are based on screening laboratory tests. Laboratory tests may be repeated once during the screening period unless otherwise indicated (see Section 4.5.2.1):

- Creatinine $>$ 1.5 times the upper limit of normal (ULN; may be repeated if 1.5–2 $\times$ ULN)
- Creatinine clearance $<$ 70 mL/min (may be repeated if 60–69 mL/min) as estimated by the Cockcroft-Gault Equation
- ALT or AST $>$ 1.5 times ULN (may be repeated if 1.5–3 $\times$ ULN)
- Total bilirubin $>$ ULN (may be repeated if 1–3 $\times$ ULN)
- Hemoglobin $<$ 11 g/dL (may be repeated if 10–10.9 g/dL)
- ANC $< 1.5 \times 10^9$/L (may be repeated if $1.2–1.5 \times 10^9$/L)
- Platelet count $< 100 \times 10^9$/L (may be repeated if $80–100 \times 10^9$/L)
- IgG $<$ 500 mg/dL (should not be repeated)
- Abnormalities in hepatic synthetic function tests (e.g., PT, INR, PTT, albumin) judged by the investigator to be clinically significant

4.2 METHOD OF TREATMENT ASSIGNMENT AND BLINDING

4.2.1 Randomization and Blinding

This study is randomized, double-blinded, and placebo-controlled to minimize potential bias in treatment assignment, patient monitoring, and efficacy assessments. Random allocation will be conducted via an interactive voice or web-based response system (IxRS) and the Sponsor will provide the specifications of the randomization algorithm to the IxRS vendor. Patients in Cohort 1 will be randomly allocated to 200 mg PO BID fenebrutinib or placebo at an approximately 2:1 ratio using a stratified permuted blocks randomization scheme with stratification by country. Patients in Cohort 2 will be randomly allocated to each of the four treatment arms at an approximately 1:1:1:1 ratio, using a stratified permuted blocks randomization scheme with stratification by country.

Patients and study site personnel will be blinded to the individual treatment assignments throughout the study. Only standard and safety laboratory data results from the local laboratory (such as CBC, chemistries, and pregnancy testing) will be available to sites. Results of other assessments performed after randomization that might unblind investigators to the treatment patients received will not be provided to sites or to the

Sponsor's staff directly involved in study conduct [redacted]

Although PK samples must be collected from patients assigned to the comparator arm to maintain the blinding of treatment assignment, PK assay results for these patients are generally not needed for the safe conduct or proper interpretation of this trial. Sponsor personnel or a designee responsible for performing PK assays will be unblinded to patients' treatment assignments to identify appropriate PK samples to be analyzed. Samples from patients assigned to the placebo arm will not be analyzed except by request (e.g., to evaluate a possible error in dosing).

Patient and study site personnel will be blinded to treatment assignments throughout the study. During trial conduct, the Sponsor will monitor blinded clinical and safety data on safety and study conduct on an ongoing basis. For Cohort 1, if required for safety evaluations, Sponsor team personnel, but not the sites, not directly involved in the conduct of the study will have access to unblinded data. These Sponsor team personnel may include individuals with clinical and medical experience, biostatisticians, and individuals responsible for analyzing and interpreting the pharmacodynamics and pharmacokinetics of the study drug. For information on Cohort 2, see Section 3.1.1.

4.2.2 Unblinding

If unblinding is necessary for patient management (e.g., in the case of a serious adverse event for which patient management might be affected by knowledge of treatment assignment), the investigator will be able to break the treatment code by contacting the IxRS. Treatment codes should not be broken except in emergency situations. If the investigator wishes to know the identity of the study drug for any other reason, he or she should contact the Medical Monitor directly. The investigator should document and provide an explanation for any premature unblinding (e.g., accidental unblinding, unblinding due to a serious adverse event).

For regulatory reporting purposes, and if required by local health authorities, the Sponsor will break the treatment code for all serious, unexpected, suspected adverse reactions (see Section 5.7) that are considered by the investigator or Sponsor to be related to study drug.

4.3 STUDY TREATMENT

The investigational medicinal product (IMP) for this study is fenebrutinib.

4.3.1 Formulation, Packaging, and Handling

4.3.1.1 Fenebrutinib and Placebo

Fenebrutinib will be provided by the Sponsor as 50-mg dose strength tablets with corresponding matching placebo tablets, which will be indistinguishable in appearance. Study drug (fenebrutinib or placebo) will be dispensed at the Day 1 and 29 visits for both cohorts.

Tablets will be supplied in bottles (Cohort 1) and blister wallets (Cohort 2) for the treatment arm to which the patient is randomly allocated. Each bottle and blister wallet will be labeled per local regulatory requirements. Fenebrutinib and placebo tablets should be stored between 2°C and 8°C. Please refer to the pharmacy manual for detailed instructions on study drug storage and preparation.

For information on the formulation and handling of fenebrutinib, see the Fenebrutinib Investigator's Brochure.

4.3.1.2 Background Therapy: Standard-of-Care H1 Antihistamines for CSU

For information on the formulation, packaging, and administration of standard-of-care H1 antihistamines for CSU, see local prescribing information.

4.3.2 Dosage, Administration, and Compliance of Fenebrutinib and Placebo

4.3.2.1 Fenebrutinib and Placebo Dose and Administration

For Cohort 1, the fenebrutinib dose level is 200 mg (4 tablets) BID (total of 8 tablets each day) with matching placebo (see Section 3.1). For Cohort 2, the fenebrutinib dose levels are 50 mg QD, 150 mg QD, and 200 mg BID, with matching placebos (see Table 3).

Patients in both cohorts will take fenebrutinib/placebo on Day 1 and ending on the Day 56 visit. Although the Day 57 visit is the last day of the study treatment period, no study drug for Study GS39684 will be given on the Day 57 visit. *Patients will* return for the safety follow-up visit 4 weeks after the last dose of study drug.

For mandatory morning clinic visits (see Appendix 1 and Section 4.4.5), patients should be instructed that the morning dose of study drug will be taken in the clinic. On other clinic visit days, if the visit occurs in the morning, the patient should be instructed that the morning dose of study drug will be taken in the clinic.

Table 3 Fenebrutinib Dosing Regimen by Treatment Arm (Cohort 2)

Fenebrutinib Dose Arm	Number of Tablets	
	Fenebrutinib (a.m./p.m.)	Placebo (a.m./p.m.)
50 mg QD	1/0	3/4
150 mg QD	3/0	1/4
200 mg BID	4/4	0/0
Placebo	0/0	4/4

BID = twice daily; QD = once daily.

Fenebrutinib or placebo may be taken orally with or without food, except on certain days (see Appendix 1), when the morning dose of oral study drug will be administered at the morning (mandatory) clinic visit while fasting. The dates and times of the most recent prior meal, last dose of oral study drug (prior to clinic visit), and timing of oral study drug administration in clinic should be recorded at each clinic visit. Patients should be instructed that a missed dose should not be taken with the next scheduled dose.

In addition, any antacids (e.g., Pepto-Bismol®, Rolaids®) should be recorded as concomitant medications, including date and time of last administration. Administration of study drug should be staggered with antacid use (i.e., study drug should be taken 2 hours before or 2 hours after the antacid).

At the Day 1 and 29 visits, sufficient study medication tablets will be dispensed to complete dosing until the end of the study. When study medication is administered at the site, it will be administered under supervision of study personnel, and the amount of study medication dispensed must be recorded.

4.3.2.2 Fenebrutinib and Placebo Compliance

The following measures will be taken to assess patient compliance with study drug. For Cohort 1, patients will be directed to bring the study drug bottle to each visit after randomization. In addition, sites will be responsible for prepopulating the dates on the dosing label (affixed to the bottle) for when patients are scheduled to take study drug. The patients will record the times (a.m. or p.m.) that they take each dose in their eDiary. The number of tablets issued minus the number of tablets returned will be used to calculate the number of tablets taken and compliance.

For Cohort 2, sites will be responsible for prepopulating the dates on the blister wallets for when patients are scheduled to take study drug. The patients will record the times (a.m. or p.m.) that they take each dose in their eDiary. Patients will be instructed to return all blister wallets (used and unused) at each study visit for assessment of compliance and for medication disposal.

Compliance will be documented on the source record. Any overdose or incorrect administration of study drug should be noted on the Study Drug Administration electronic Case Report Form (eCRF). Adverse events associated with an overdose or incorrect administration of study drug should be recorded on the Adverse Event eCRF. If compliance is ≤ 80%, the investigator or designee is to counsel the patient and ensure steps are taken to improve compliance.

4.3.2.3 Background Therapy: Standard-of-Care H1 Antihistamines for CSU

The following H1 antihistamine medications are allowed:

- Cetirizine 10–40 mg QD
- Levocetirizine 5–20 mg QD
- Fexofenadine 180–720 mg QD
- Loratadine 10–40 mg QD
- Desloratadine 5–20 mg QD
- Rupatadine 10–40 mg QD
- Bilastine 20–80 mg QD

All patients will be allowed to take study-defined, second-generation, H1 antihistamine medications consistent with standard-of-care (i.e., up to 4 times the approved dose per local treatment guidelines) during the screening, treatment, and follow-up periods. Patients should remain on a stable H1 antihistamine regimen throughout the study period. Loratadine (10 mg) or cetirizine (10 mg) will be provided and used on an as-needed basis (maximum 1 per day) during screening, treatment, and follow-up periods). Therapies used for the treatment of CSU prior to enrollment will be collected as part of the patient's medical history.

Patients taking either LTRAs or H2 blockers for diseases other than CSU (e.g., asthma or gastroesophageal reflux disease, respectively) at screening will be permitted to continue their use at a stable dose during the study. These diseases must be recorded as part of the medical history collected during the screening period. Inhaled asthma controllers, including inhaled corticosteroids, are permitted during the study.

4.3.3 Investigational Medicinal Product Accountability

All IMPs required for completion of this study (fenebrutinib and placebo) will be provided by the Sponsor where required by local health authority regulations. The study site will acknowledge receipt of IMPs using the IxRS to confirm the shipment condition and content. Any damaged shipments will be replaced.

IMPs either will be disposed of at the study site according to the study site's institutional standard operating procedure or will be returned to the Sponsor with the appropriate documentation. The site's method of IMP destruction must be agreed to by the Sponsor.

The site must obtain written authorization from the Sponsor before any IMP is destroyed, and IMP destruction must be documented on the appropriate form.

Accurate records of all IMPs received at, dispensed from, returned to, and disposed of by the study site should be recorded on the Drug Inventory Log.

4.3.4 Post-Trial Access to Fenebrutinib

Currently, the Sponsor (Genentech, a member of the Roche Group) *does not have any plans to provide Genentech IMP fenebrutinib or any other study treatments to patients who have completed the study. The Sponsor may evaluate whether to continue providing fenebrutinib in accordance with* the Roche Global Policy on Continued Access to Investigational Medicinal Product, available at the following website:

http://www.roche.com/policy_continued_access_to_investigational_medicines.pdf

4.4 CONCOMITANT THERAPY AND ADDITIONAL RESTRICTIONS

Concomitant therapy includes any medication (e.g., prescription drugs, over-the-counter drugs, vaccines, herbal or homeopathic remedies, nutritional supplements) used by a patient from 14 days prior to initiation of study drug to the study completion/ discontinuation visit. All such medications (including standard-of-care H1 antihistamines for CSU) should be reported to the investigator and recorded on the Concomitant Medications eCRF.

4.4.1 Permitted Therapy

Patients who use oral contraceptives, hormone-replacement therapy, or other maintenance therapy should continue their use. All concomitant medications should be reported to the investigator and recorded on the appropriate eCRF. Patients will be encouraged to use the minimal dose required to control their symptoms.

For the purposes of this study, dietary supplements are defined as vitamins, minerals, purified food substances, and herbals with pharmaceutical properties.

Vitamins, minerals, and purified food substances are allowed in amounts not known to be associated with adverse effects (e.g., hypervitaminosis). Herbals with pharmaceutical properties are allowed only if there is acceptable evidence of no CYP3A inhibition or induction (refer to Section 4.4.3 for a list of prohibited concomitant medications, including herbal products). Otherwise, herbals with pharmaceutical properties must be discontinued for at least 4 weeks prior to the first dose of study medication, unless there are sufficient data available regarding the duration of an herbal medication's PK and PD effects to allow a shorter washout to be specified (e.g., 5 half-lives). Please direct any questions to the Medical Monitor.

4.4.2 Cautionary Therapy

4.4.2.1 Acid-Reducing Agents

Patients who use antacids (e.g., Pepto-Bismol®, Rolaids®) for symptomatic relief of heartburn should take fenebrutinib or matching placebo at least 2 hours before or 2 hours after antacid administration because gastric acid improves fenebrutinib absorption.

Patients may be treated with PPIs or H2RAs at up to the maximum recommended dose according to local labeling. The dose should remain stable for at least the 2 weeks before randomization and throughout the study.

At visits with scheduled PK assessments (see Appendix 1), any use of PPIs, H2RAs, and/or other antacids (e.g., Pepto-Bismol®, Rolaids®) should be recorded as concomitant medications, including the date and time of last administration.

4.4.2.2 Statins

Several lipid-lowering agents (statins) are metabolized by CYP3A (simvastatin, lovastatin) and/or transported by BCRP (rosuvastatin, atorvastatin), and thus may be affected by drug-drug interaction with fenebrutinib, therefore, dose adjustments of these medications should be considered (Kellick et al. 2014).

- Simvastatin: recommended maximum dose of 10 mg/day
- Lovastatin: recommended maximum dose of 20 mg/day
- Rosuvastatin: recommended maximum dose of 10 mg/day
- Atorvastatin: recommended maximum dose of 20 mg/day

The use of statins has been associated with myopathy, which can manifest as weakness, tenderness or muscle pain with elevations of creatine kinase (CK) above ten times the ULN. In severe cases, myopathy can cause rhabdomyolysis with or without acute kidney injury secondary to myoglobinuria, and rare fatalities due to rhabdomyolysis have occurred. The risk of myopathy is increased by elevated plasma levels of statins. Predisposing factors for myopathy include advanced age ($\geq$65 years), female gender, uncontrolled hypothyroidism, renal impairment, or the use of concomitant medications that increase the plasma levels of the statin.

4.4.2.3 CYP3A and BCRP-Mediated Drug Interactions

Preliminary data from a clinical drug-drug interaction study (Study GP39616) suggest that fenebrutinib can be classified as a mild inhibitor of CYP3A at clinically relevant doses. It is possible that fenebrutinib inhibition of CYP3A may alter the metabolism of CYP3A substrates and result in increased plasma concentrations of CYP3A substrates. Therefore, medications in the following categories (listed in detail in Appendix 5) should be used with caution in consultation with the Medical Monitor (or delegate) as necessary unless otherwise specified in Appendix 5:

- Sensitive CYP3A substrates

- CYP3A substrates with a narrow therapeutic window

The use of hormone-replacement therapy or hormonal contraceptives containing the CYP3A substrate ethinylestradiol (with the concomitant use of a barrier method) is permitted; however, these agents should be used with caution, and patients should be counseled regarding the potential risks and benefit of these medications per the local prescribing information. Ethinyl estradiol is metabolized by CYP3A so plasma concentrations of ethinyl estradiol are expected to increase in the presence of fenebrutinib (Zhang et al. 2007, Wang et al. 2004). Ethinyl estradiol is not a sensitive substrate of CYP3A (Drug Development and Drug Interactions: Table of Substrates, Inhibitors and Inducers, www.fda.gov). Therefore, the magnitude of increase in ethinyl estradiol plasma concentrations is expected to be less than the increase observed in midazolam concentrations (i.e., less than 2-fold) in Study GP39616, a drug-drug interaction study evaluating the effect of fenebrutinib on the PK of the sensitive CYP3A substrate midazolam. Minor increases in ethinyl estradiol concentrations are not generally associated with adverse events (e.g., Ortho Tri-Cyclen® USPI). Ethinyl estradiol efficacy is expected to be maintained, and ethinyl estradiol continues to be considered a reliable and effective form of contraception in combination with fenebrutinib.

Preliminary data from Study GP39616 also suggest that fenebrutinib can be classified as a moderately sensitive substrate of CYP3A at clinically relevant doses. There is a moderate potential for a drug-drug interaction with any medication that strongly inhibits or induces this enzyme. Therefore, medications in the following categories (listed in detail in Appendix 5) should be avoided for 7 days or 5 half-lives, whichever is longer, prior to the first dose of study drug and until the last dose of study drug. If use of one of these medications is necessary, the risks and benefits should be discussed with the Medical Monitor (or delegate) prior to concomitant administration with study drug:

- Strong CYP3A inhibitors
- Moderate or strong CYP3A inducers

Lastly, preliminary data from Study GP39616 suggest that fenebrutinib is a moderate inhibitor of the breast cancer resistance protein (BCRP) (also known as ABCG2) transporter protein at clinically relevant doses. There is a potential for increased plasma concentrations of drugs that are known to be substrates of the BCRP transporter. Plasma concentrations of the medications in the following category (listed in detail in Appendix 5) may increase; therefore, they should be used with caution in consultation with the Medical Monitor (or delegate) as necessary unless otherwise specified in Section 4.4.1.

The medications listed above and in Section 4.4.1 are not necessarily comprehensive. Thus, the investigator should consult the prescribing information for any concomitant medication as well as the Internet references provided below when determining whether a certain medication is metabolized by or strongly inhibits or induces CYP3A. The

investigator should contact the Medical Monitor if questions arise regarding medications not listed above.

https://www.fda.gov/Drugs/DevelopmentApprovalProcess/DevelopmentResources/DrugInteractionsLabeling/ucm093664.htm (Tables 3-1, 3-2, 3-3, and 5-1)

http://medicine.iupui.edu/clinpharm/ddis/table.aspx

4.4.3 Prohibited Therapy

Prior to the screening visit (Day −14 for both cohorts) as specified below, and during the study (not including the safety follow-up period if patient failed additional H1 antihistamine for worsened symptoms; see Section 3.1) the following medications and treatments will be restricted. Patients who receive these medications as therapy for CSU will be discontinued from the study treatment but will be followed for safety evaluation:

- Systemic or topical corticosteroids (prescription or over the counter), hydroxychloroquine, methotrexate, cyclosporine, or cyclophosphamide within 30 days prior to screening if used routinely (daily or every other day during 5 or more consecutive days) – The use of corticosteroids may be used for exacerbations
- Doxepin within 30 days prior to screening
- Omalizumab or other monoclonal antibody therapies used to treat CSU within 4 months prior to screening
- IVIG within 30 days prior to screening
- Plasmapheresis within 30 days prior to screening
- LTRAs within 1 day prior to screening
- Astemizole, terfenadine, and ebastine within 1 day prior to screening

4.4.3.1 Live or Attenuated Vaccinations

Immunization with a live or attenuated vaccine is prohibited within 6 weeks prior to study drug administration on Day 1 and for the duration of study participation, including the 4-week safety follow-up period after the administration of the last dose. See Section 5 for further details and precautions around vaccinations.

4.4.4 Prohibited Food

Use of the following foods is prohibited during the study and for at least 7 days prior to initiation of study treatment: furanocoumarin derivatives as found in grapefruit, Seville orange, pomegranate, or star fruit juice or products. Please refer to Appendix 5 for additional information.

4.4.5 Additional Restrictions

Patients in both cohorts should be fasting overnight for > 8 hours prior to the PK draw and/or fasting lipid panel on Days 1, 8, 29, 57, and 85 (see Appendix 1).

4.5 STUDY ASSESSMENTS

Please see Appendix 1 for the schedule of activities to be performed during the study.

4.5.1 Informed Consent Forms and Screening Log

Written informed consent for participation in the study must be obtained before performing any study-related procedures. Informed Consent Forms for enrolled patients and for patients who are not subsequently enrolled will be maintained at the study site.

All screening evaluations must be completed and reviewed to confirm that patients meet all eligibility criteria before enrollment. The investigator will maintain a screening log to record details of all patients screened and to confirm eligibility or record reasons for screening failure, as applicable.

4.5.2 Eligibility at Screening

At screening, patients who fail to meet any laboratory inclusion/exclusion criteria or other eligibility criteria may be retested or re-screened as per the instructions in Section 4.5.2.1 and Section 4.5.2.2, respectively.

4.5.2.1 Retesting: Laboratory Inclusion/Exclusion

If a patient does not meet certain laboratory inclusion/exclusion criteria at screening, the investigator may repeat the tests once within the screening period (see Section 4.1.2 for laboratory tests and levels that can be retested). If the patient meets the laboratory eligibility criteria on the second assessment, he or she will be permitted to enter the study. It will not be considered a retesting if blood samples have to be redrawn because of sample handling problems, breakage, sample integrity, or laboratory error.

4.5.2.2 Re-screening

Re-screening refers to repeating the entire screening process. Re-screening is required if a patient has not met eligibility criteria within the original screening visit. (Note: patients who have failed two laboratory testing attempts as described in Section 4.1.2 cannot be re-screened). Patients are allowed to be re-screened only once per cohort. Each patient must be re-consented before re-screening occurs. It will not be considered a re-screening if blood samples have to be redrawn because of sample handling problems, breakage, sample integrity, or laboratory error.

4.5.3 Medical History and Demographic Data

Comprehensive medical and surgical history, including a comprehensive review of the patient's CSU medical history, will be collected at the Day −14 visit for both cohorts. This review will include onset of symptoms, date of diagnosis, and therapies received for CSU. In addition, history of omalizumab (Xolair®) use and reason for discontinuation will be collected.

Concomitant medical usage will be collected at all visits, including unscheduled visits. Demographic data will include age, sex, and self-reported race/ethnicity.

4.5.4 Physical Examinations

A complete physical examination should be performed at the Day −14 visit for both cohorts and should include an evaluation of the head, eyes, ears, nose, and throat, and the cardiovascular, dermatological, musculoskeletal, respiratory, GI, genitourinary, and neurological systems. Any abnormality identified at baseline should be recorded on the General Medical History and Baseline Conditions eCRF.

Subsequent examinations may be limited to detect changes in symptoms of CSU as well as directed by patient complaints regarding adverse events. Changes from baseline abnormalities should be recorded in patient notes. New or worsened clinically significant abnormalities should be recorded as adverse events on the Adverse Event eCRF.

4.5.5 Vital Signs

Vital signs will include measurements of heart rate, systolic and diastolic blood pressure while the patient is in a seated position, and temperature. Vital signs will be assessed as outlined in the Schedule of Activities in Appendix 1 and during other unscheduled study visits when clinically indicated. The patients' height and weight will be measured once during the screening visit for both cohorts.

4.5.6 FricTest

For subjects who have a history of dermographism, a FricTest® will be performed at screening (Day −14), baseline (Day 1), and Days 57 and 85 for both cohorts. The FricTest® is a flat, plastic comb with four round-ended plastic pins, 3 mm in diameter and of different lengths. The FricTest® defines provocation thresholds and severity of dermographism (i.e., 4 pins inducing wheals defines severe dermographism).

4.5.7 Laboratory, Biomarker, and Other Biological Samples

Samples for the following laboratory tests will be sent to one or several central laboratories for analysis as per the Schedule of Activities in Appendix 1. Laboratory tests prior to randomization and dosing may be performed locally on Day 0, if central laboratory tests are not available due to sampling handling problems, breakage, or lab error.

- Hematology: hemoglobin, hematocrit, platelet count, RBC count, WBC count, percent and absolute differential counts (neutrophils, bands, lymphocytes, monocytes, eosinophils, basophils, other cells)
- Serum chemistry: sodium, potassium, chloride, bicarbonate, glucose, BUN, creatinine, calcium, phosphorus, magnesium, total and direct bilirubin, total protein, albumin, ALT, AST, LDH, alkaline phosphatase, creatine phosphokinase, CRP, lipase, and uric acid
- Urinalysis including dipstick (pH, specific gravity, glucose, protein, ketones, blood) and microscopic examination (sediment, RBCs, WBCs, casts, crystals, epithelial cells, bacteria)
- Coagulation: INR, activated PTT, PT, fibrinogen

- Fasting lipid panel
- Viral serology
- Hepatitis B: HBsAg, total HBcAb, and hepatitis B surface antibody
- Hepatitis C antibody
- [redacted]
- [redacted]
- The following samples will be sent to the Sponsor or a designee for analysis:
- [redacted]
- Plasma samples for PK analysis
- See the Schedule of Activities provided in Appendix 1 for specific timepoints.
- Samples for the following laboratory tests will be sent to the study site's local laboratory for analysis:
- Pregnancy test

  All women of childbearing potential (including those who have had a tubal ligation) will have a serum pregnancy test at screening. Urine pregnancy tests will be performed at specified subsequent visits. If a urine pregnancy test result is positive, it must be confirmed by a serum pregnancy test. Should a positive result be recorded at any time, the procedures detailed in Section 5.4.3 should be followed. If a local urine pregnancy test shows a positive result, then study drug will not be administered that day. Other study procedures should also be postponed and the result must be confirmed by a serum pregnancy test prior to proceeding.
- QFT or PPD (if QFT not available) and additional procedures (e.g., chest X-ray) to rule out latent or active TB per local guidelines

See the Schedule of Activities provided in Appendix 1 for specific timepoints.

[redacted]

[redacted]

[redacted]	[redacted]
[redacted]	[redacted]
[redacted]	[redacted]
[redacted]	[redacted]

[redacted]

For sampling procedures, storage conditions, and shipment instructions, see the laboratory manual.

Biological samples will be destroyed when the final clinical study report (CSR) has been completed, with the following exceptions:

- Plasma samples collected for PK analysis will be destroyed no later than 5 years after the final CSR has been completed.
- Blood, urine, and serum samples collected for biomarker analyses will be destroyed no later than 15 years after the final CSR has been completed.

When a patient withdraws from the study, samples collected prior to the date of withdrawal may still be analyzed, unless the patient specifically requests that the samples be destroyed or local laws require destruction of the samples. However, if samples have been tested prior to withdrawal, results from those tests will remain as part of the overall research data.

Data arising from sample analysis will be subject to the confidentiality standards described in Section 8.4.

4.5.8 Electrocardiograms

A single ECG recording, without artifacts, must be obtained at specified timepoints, as indicated in Appendix 1. The ECG intervals (e.g., PR, QRS, QT, QTcF, and RR) and heart rate from this ECG will be entered into the eCRF. Any morphologic waveform changes or other ECG abnormalities must be documented on the eCRF.

All ECG recordings must be performed using a standard high-quality, high-fidelity digital electrocardiograph machine equipped with computer-based interval measurements. Lead placement should be as consistent as possible. ECGs for each patient should be obtained from the same machine whenever possible. ECG recordings must be performed after the patient has been resting in a supine position for at least 10 minutes prior to beginning the ECG recording. All ECGs can be performed without specific restrictions (e.g., can be performed at any time of day, before or after dosing, fasting or fed) but are to be obtained prior to other procedures scheduled at that same time (e.g., vital sign measurements, blood draws). Body position should be consistently maintained for each ECG evaluation to prevent changes in heart rate. Circumstances

that may induce changes in heart rate, including environmental distractions (e.g., television, radio, conversation) should be avoided during the pre-ECG resting period and during ECG recording.

For safety monitoring purposes, the investigator must review, sign, and date all ECG tracings. Paper copies of ECG tracings will be kept as part of the patient's permanent study file at the site. If considered appropriate by the Sponsor, ECGs may be analyzed retrospectively at a central laboratory.

If at a particular post-dose timepoint the mean QTcF is > 500 ms or > 60 ms longer than the baseline value, another ECG must be recorded, ideally within the next 5 minutes, and ECG monitoring should continue until QTcF has stabilized on two successive ECGs. The Medical Monitor should be notified as soon as possible within 24 hours. Standard-of-care treatment may be instituted per the discretion of the investigator. If a PK sample is not scheduled for that timepoint, an unscheduled PK sample should be obtained. A decision on study drug discontinuation should be made, as described in Section 4.6.2. The investigator should also evaluate the patient for potential concurrent risk factors (e.g., electrolyte abnormalities, co-medications known to prolong the QT interval, severe bradycardia).

4.5.9 Patient-Reported Outcomes

Data from two patient reported outcomes (PRO) tools will be collected via questionnaires to document the treatment benefit of fenebrutinib: the Urticaria Patient Daily eDiary, [redacted]. The eDiary and [redacted] translated into the local language as required, will be completed in their entirety at specified timepoints during the study. [redacted]

Patients will use an electronic device to capture the Urticaria Patient Daily eDiary (see Appendix 3). The electronic device and/or instructions for completing the questionnaires electronically will be provided by the investigator staff. The data will be transmitted to a centralized database maintained by the electronic device vendor. The data will be available for access by appropriate study personnel.

[redacted]

4.5.9.1 Urticaria Patient Daily eDiary

The Urticaria Patient Daily eDiary includes the UAS, which will be used to calculate the UAS7. The eDiary comprises questions regarding largest hive size, sleep interference

score, activity interference question, rescue medication use, angioedema episodes, number of calls to doctor or nurse practitioner, and study medication compliance.

The eDiary is to be completed twice per day (a.m./p.m.) by the patient for the duration of the study. The eDiary will be given to the patient at the Day −14 visit for both cohorts.

4.5.9.2 Urticaria Activity Score

During the week prior to Day 1 (i.e., Week −1), UAS7 will be recorded twice daily for the purposes of enrollment eligibility for both cohorts. Subsequently, the UAS will be recorded twice daily using the Urticaria Patient Daily eDiary.

The UAS is a composite, eDiary-recorded score with numeric severity intensity ratings (0 = none to 3 = intense/severe) for a) the number of wheals (hives) and b) the intensity of the pruritus (itch) over the past 12 hours (twice daily; see Section 3.3.6). The daily UAS is calculated as the average of the morning and evening scores. The UAS7 will be calculated and is the weekly sum of the daily UAS, which is the composite score of the intensity of pruritus and the number of wheals. The maximum UAS7 value is 42; the intensity of the itch/pruritus and the number of wheals/hives are graded in Table 2.

[redacted]

[redacted]

[redacted]

[redacted]

[redacted]

[redacted]

[redacted]

[redacted]

[redacted]

[redacted]

4.6 PATIENT, TREATMENT, STUDY, AND SITE DISCONTINUATION

4.6.1 Patient Discontinuation

Patients have the right to voluntarily withdraw from the study at any time for any reason. In addition, the investigator must withdraw a patient from the study for the following, but not limited to, reasons:

- Patient withdrawal of consent at any time
- Any medical condition that the investigator or Sponsor determines may jeopardize the patient's safety if he or she continues in the study
- Investigator or Sponsor determines it is in the best interest of the patient

- The investigator may withdraw a patient from the study at any time due to patient non-compliance (e.g., drug compliance ≤ 80%, missed visits, missing Urticaria Patient Daily eDiary entries)

For patients who withdraw from the study, every effort should be made to complete an early termination visit including the assessments on the Schedule of Activities (see Appendix 1). The primary reason for withdrawal from the study should be documented on the appropriate eCRF. However, patients will not be followed for any reason after consent has been withdrawn. Patients who withdraw from the study will not be replaced.

If the patient discontinues the study prior to Day 85 visit, the patient will be asked to return to the clinic for a safety follow–up visit 4 weeks after the last dose of study drug. Patients who refuse to complete the safety follow–up period should undergo an early termination visit. Patients who discontinue during the safety follow-up period prior to completion of the 4-week safety follow-up will be asked to complete an early termination visit (see Appendix 1).

If a patient withdraws for reasons related to a serious adverse event, every attempt should be made to follow the patient until resolution of the event.

4.6.2 Study Treatment Discontinuation

Patients must discontinue study treatment if they experience any of the following:

- Pregnancy
- Malignancy
- Any serious infection or infection requiring treatment with an IV antimicrobial agent
- Any prohibited medication as defined in Section 4.4.3

Patients who discontinue study treatment prematurely (prior to Day 85 visit), including but not limited to the reasons listed above, will be asked to return to the clinic for a safety follow-up visit 4 weeks after the last dose of study drug. Patients who refuse to complete the safety follow up period should return to the clinic for an early termination visit.

The primary reason for study treatment discontinuation should be documented on the appropriate eCRF. Patients who discontinue study treatment prematurely will not be replaced.

4.6.3 Study and Site Discontinuation

The Sponsor has the right to terminate this study at any time. Reasons for terminating the study include, but are not limited to, the following:

- The Sponsor must terminate the study if the Sponsor believes that the incidence or severity of adverse events in this or other studies indicates a potential health hazard to patients.

- The Sponsor may terminate the study if patient enrollment or completion of the study unsatisfactory.

The Sponsor will notify the investigator if the Sponsor decides to discontinue the study.

The Sponsor has the right to close a site at any time. Reasons for closing a site may include, but are not limited to, the following:

- The Sponsor must close a site if corrective actions to improve site performance in the following areas do not yield significant improvement:
  - Excessively slow recruitment
  - Poor protocol adherence
  - Inaccurate or incomplete data recording
  - Non-compliance with the International Council on Harmonisation (ICH) guideline for Good Clinical Practice
- No study activity (i.e., all patients have completed the study and all obligations have been fulfilled).

5. ASSESSMENT OF SAFETY

5.1 SAFETY PLAN

The safety plan for patients in this study is based on nonclinical and clinical experience with fenebrutinib in completed and ongoing studies, as well as published literature, on other BTK inhibitors and BTK biology. The important potential safety risks for fenebrutinib are outlined below. Please refer to the Fenebrutinib Investigator's Brochure for a complete summary of safety information.

Several measures will be taken to ensure the safety of patients participating in this study. Eligibility criteria have been designed to exclude patients at higher risk for potential toxicities. Patients will undergo safety monitoring during the study, including monitoring of vital signs, physical examination, ECGs, and routine laboratory safety assessments (hematology, chemistry, and urinalysis) and assessment of the nature, frequency, and severity of adverse events. In addition, guidelines for managing potential adverse events, including criteria for treatment interruption or discontinuation, and enhanced safety reporting are provided below.

In addition, an unblinded IMC will monitor patient safety throughout the study for Cohort 2 (see Section 3.1.1).

5.1.1 Safety Plan for Potential Risks Associated with Fenebrutinib

5.1.1.1 Infections

Fenebrutinib is a reversible inhibitor of BTK, and the degree to which fenebrutinib antagonism of BTK signaling may suppress immune activity is unknown. On the basis of patients with XLA, a primary immunodeficiency caused by mutations in the BTK gene,

it is anticipated that inhibitors of BTK may raise the risk for certain bacterial infections (Lederman and Winkelstein 1985; Broides et al. 2006), enteroviral infections (Misbah et al. 1992; Ziegner et al. 2002), intestinal infections with giardia and *Campylobacter* species (Winkelstein et al. 2006; van den Bruele et al. 2010), or other opportunistic infections, which are cleared primarily by B-cell adaptive immune responses. This risk is likely independent of sex for patients exogenously administered fenebrutinib.

Effects on lymphocytes and immunoglobulins in rats and dogs were reversible and considered to be related to pharmacological activity involving BTK inhibition. See Section 1.2.2 for related primary nonclinical toxicity findings and the Fenebrutinib Investigator's Brochure for further details.

To date, no immune-challenge experiments (e.g., T-dependent antigen response test) have been conducted in animals. It is not known whether these effects on B cells and IgG concentrations in animals will translate to humans or whether such changes would have functional or deleterious impact on immune function.

Infections, including pneumonia and fatal influenza, have occurred in patients with B-cell malignancies treated with fenebrutinib. In studies with healthy subjects with single doses and with dosing for 14 days, self-limited Grade 1 events of nasopharyngitis were reported but did not lead to any change in study drug dosing. One healthy subject had asymptomatic bacteriuria, which resolved while study drug dosing continued. In blinded and open-label studies in autoimmune indications, serious, severe, and opportunistic infections have been reported, including one case of pulmonary tuberculosis as well as cases of pneumonia, bronchitis, cellulitis, enterobacter infection, herpes zoster, acute pyelonephritis, necrotizing soft tissue infection, colitis, periorbital cellulitis, and sinusitis. As a safety precaution, the study protocol contains exclusion criteria for infections and potential infection risk and guidelines for study drug management in the event of infection. Study subjects and patients should be monitored for fever and potential infectious complications, including opportunistic infections, and evaluated promptly.

Patients will be excluded from the study if they have a history of hospitalization due to an infection in the 8 weeks before screening, evidence of active or latent or inadequately treated infection with *Mycobacterium* TB, known active infection (current) or history of recurrent infection, or any known immunodeficiency including IgG < 500 mg/dL.

[redacted] All patients in the study should be monitored for fever and potential infectious complications, including opportunistic infections and TB, and should be evaluated promptly. Physicians or a health care provider should give patients advice to prevent potential transmission of and exposure to endemic infections according to local or Centers for Disease Control and Prevention guidelines. Patients should be advised to seek immediate medical attention if they develop signs and symptoms suggestive of an infection. All infections occurring during the study, including but not limited to respiratory infections, cutaneous

infections, urinary tract infections, systemic viral infections, and episodes of suspicious or febrile diarrhea, should be evaluated using serology or polymerase chain reaction, if available, and cultured, if feasible, and any identified organisms noted in the eCRF. Any serious infection, infection requiring IV antimicrobials, or any opportunistic infection is considered an adverse event of special interest and should be reported to the Sponsor as outlined in Section 5.4.2.

Guidelines for management of study treatment in the event that infections are observed in patients are provided in Section 5.1.2.

Please refer to the Fenebrutinib Investigator's Brochure for further details.

5.1.1.2 Vaccinations

The effect of fenebrutinib upon the efficacy of vaccinations is unknown. It is recommended that appropriate vaccinations per local guidelines be up to date before study participation. Patients will be excluded from study participation and will not be dosed with fenebrutinib if they have been vaccinated with live, attenuated vaccines (e.g., the intranasal live attenuated influenza vaccines, BCG, varicella) within 6 weeks before planned dosing. In addition, immunization with a live or attenuated vaccine is prohibited for the duration of study participation, including the 4-week safety follow-up period after the administration of the last dose.

In addition, current routine household contact with children or others who have been vaccinated with live vaccine components may pose a risk to the patient during study treatment with fenebrutinib. Some of these vaccines include varicella ("chickenpox") vaccine, oral polio vaccine, and the inhaled flu vaccine. Following vaccination with live component vaccines, the virus may be shed in bodily fluids, including stool, and there is a potential risk that the virus may be transmitted to the patient.

General guidelines for immunosuppressed patients suggest that exposure to vaccinated individuals should be avoided following vaccination with these vaccines for the stated time periods:

- Varicella or attenuated typhoid fever vaccination for 4 weeks following vaccination
- Oral polio vaccination for 6 weeks following vaccination
- Attenuated rotavirus vaccine for 10 days following vaccination
- FluMist® (inhaled flu vaccine) for 1 week following vaccination

Please refer to the Fenebrutinib Investigator's Brochure for further details.

5.1.1.3 Bleeding

No decrease in platelets, changes in coagulation parameters, or bleeding events were observed in nonclinical studies with fenebrutinib. Bleeding events, including non-serious NCI CTCAE v4.0 Grade 1 bruising and serious Grade ≥3 GI bleeding, have been reported in patients with hematological malignancies treated with fenebrutinib in

Study GO29089. The GI bleeding events have not been dose related, and the events occurred in patients who were taking concomitant NSAIDs and who had a history of gastroesophageal or peptic ulcer disease. The impact of BTK inhibition as a potential risk factor for bleeding is unknown. BTK is expressed in platelets and is involved in platelet function via GPVI/collagen receptor signaling and GP1b receptor signaling. Platelets from patients with XLA, a genetic deficiency of BTK, demonstrate decreased activation in response to submaximal collagen stimulation but normal response to thrombin; clinically, there is no reported bleeding propensity of patients with XLA.

Bruising or bleeding events related to fenebrutinib have not been reported in healthy subjects. Grade ≥ 2 bleeding events have been reported in blinded and open-label studies of fenebrutinib in autoimmune indications, including hematuria, purpura, hematoma, and uterine and vaginal bleeding.

It is unknown whether fenebrutinib will increase the risk of bleeding in patients, especially in those receiving anti-platelet or anticoagulant therapies. As a precautionary safety measure, patients will be excluded from study participation if they have a need for systemic anticoagulation with warfarin or other oral or injectable anticoagulants or anti-platelet agents (other than NSAIDs, aspirin, and other salicylates), any history of hospitalizations or transfusion for a GI bleed, any history of a hemorrhagic CVA, any history of spontaneous intracranial hemorrhage, traumatic intracranial hemorrhage within 10 years prior to the study, or a known bleeding diathesis. Patients should be advised to seek immediate medical attention if they develop signs and symptoms suggestive of clinically significant bleeding.

Bleeding events of moderate or greater severity are considered adverse events of special interest and should be reported to the Sponsor in an expedited manner as outlined in Section 5.4.2.

Guidelines for management of study treatment in the event that bleeding is observed in patients are provided in Section 5.1.2. Please refer to the Fenebrutinib Investigator's Brochure for further details.

5.1.1.4 Cytopenias

Cytopenias have been observed in patients with hematological malignancies who received fenebrutinib as well as in the ongoing, blinded Phase II studies and OLEs in autoimmune diseases. Cytopenias have included neutropenia, anemia, and thrombocytopenia; events have been monitorable and clinically manageable (see the Fenebrutinib Investigator's Brochure for further details).

Patients should be monitored regularly with hematology laboratory evaluations as outlined in the schedule of activities (see Appendix 1) and should receive appropriate supportive care as clinically indicated. Patients should be advised to seek immediate medical attention if they develop signs and symptoms suggestive of cytopenias

(e.g., persistent fever, bruising, bleeding, pallor). Cytopenias should be managed according to local clinical guidelines.

Guidelines for managing study treatment in the event that cytopenia is observed are provided in Section 5.1.2. Please refer to the Fenebrutinib Investigator's Brochure for further details.

5.1.1.5 Gastrointestinal Effects

Body weight gain and food consumption changes have been observed in animals, including nonsignificant increases in male Wistar-Han rats administered ≥2 mg/kg/day (4.3 μM•hr) for 6 months, and significant reductions in rats administered 100 mg/kg/day (1438 μM•hr) and dogs administered 25 mg/kg (180 μM•hr) for 4 weeks. These effects on body weight gain and food consumption were reversible following discontinuation of fenebrutinib dosing.

NCI CTCAE v4.0 Grade 1 diarrhea, nausea, and abdominal pain have been reported in patients with B-cell malignancies; however, the events have resolved and have not led to study drug discontinuation. Healthy subjects in the MAD Study GA29347 reported events of mild self-limited nausea. Across studies with immune indications receiving blinded or open-label treatment, approximately 5% of enrolled patients have reported nausea, vomiting, diarrhea, or other gastrointestinal symptoms.

Throughout the study, patients will be monitored for GI side effects.

Guidelines for management of study treatment in the event of GI side effects in patients are provided in Section 5.1.1.5. Please refer to the Fenebrutinib Investigator's Brochure for further details.

5.1.1.6 Hepatotoxicity

Evidence of hepatobiliary injury was observed in animals administered relatively high doses of fenebrutinib in repeat-dose toxicity studies. Dose-dependent increases in ALT, AST, and/or bilirubin have been observed in rats administered ≥6 mg/kg/day (≥17 μM•hr) and dogs administered ≥10 mg/kg/day (≥36 μM•hr), with corresponding microscopic changes in the liver of dogs administered 25 mg/kg/day (180 μM•hr). The hepatotoxicity findings in dogs were associated with moribundity in two high-dose animals. The NOAEL for these findings was considered to be 10 mg/kg (36 μM•hr) in dogs, the most sensitive species, given the absence of fenebrutinib–related hepatotoxicity at this dose when administered for 9 months. This exposure provides a 5-fold safety multiple above the study dose of 200 mg BID (projected $AUC_{0\text{-}24}$ of 6.7 μM•hr). The hepatotoxicity findings were fully reversible and considered monitorable by changes in plasma transaminases and bilirubin that occurred at doses lower than those producing histopathology findings (see the Fenebrutinib Investigator's Brochure for further details).

In clinical studies in autoimmune indications, including in patients with CSU, cases of Grade 3 (or severe) transaminase elevations, including cases that were considered

serious by the investigator have been reported. These transaminase elevations have occurred in both the randomized clinical studies, which remain blinded to the Sponsor in terms of treatment assignment, and the open-label extension. The transaminase levels returned to normal after discontinuation of the study treatment. There have been no observed AEs of liver enzyme elevation in clinical studies to date in healthy subjects or patients with hematological malignancies. To date, no cases of transaminase elevations have led to clinical jaundice or bilirubin $> 2 \times$ ULN (Hy's Law). All transaminase elevations have been reversible when dosing of blinded study medication/placebo was withheld.

As a safety risk-mitigation measure, to be eligible for the study, AST and/or ALT levels should be no more than $1.5 \times$ ULN, and total bilirubin levels should be normal at screening. Safety monitoring for potential hepatotoxicity includes baseline and routine evaluations of AST/ALT and total bilirubin levels throughout the study as outlined in the schedule of activities (see Appendix 1).

Laboratory results of either an AST or ALT $> 5 \times$ ULN or an AST or ALT $> 3 \times$ ULN in combination with a total bilirubin $> 2 \times$ ULN, of which at least 35% is direct bilirubin or there is clinical jaundice, are considered adverse events of special interest and should be reported to the Sponsor in an expedited manner as outlined in Section 5.4.2.

Guidelines for the management of study treatment in the event of hepatotoxicity in patients are provided in Section 5.1.2. Please refer to the Fenebrutinib Investigator's Brochure for further details.

5.1.1.7 Cardiovascular Effects

Fenebrutinib is considered to have a low potential to cause QT interval prolongation or to directly affect other cardiovascular parameters, at therapeutic exposures. A minimal increase in QTc (7 ms or 3%) interval was noted at 45 mg/kg in the single-dose cardiovascular safety pharmacology study in telemetry-instrumented dogs. Based on extrapolated/interpolated toxicokinetic data, the unbound C_{max} at 45 mg/kg (considered a NOAEL) and no-observed–effect level of 15 mg/kg were 23-fold and 7-fold higher, respectively, than the mean unbound C_{max} in humans at the 200-mg BID dose. There have been no fenebrutinib–related changes in ECG parameters in the 4-week or 9-month dog toxicity studies.

Analysis of ECG data from the SAD and MAD studies in healthy subjects did not demonstrate any significant increase in either QRS interval or QTcF intervals. However, cardiac safety will be evaluated in all patients at baseline and throughout this study, with routine monitoring of vital signs (including heart rate and blood pressure), routine safety ECGs, and collection of adverse events (see Section 5.1.1.7 and Section 5.2.1).

Management of patients with sustained QTcF prolongation (QTcF that is >500 ms or >60 ms longer than the baseline value) should include recording another ECG, ideally within the next 5 minutes, and ECG monitoring should continue until QTcF has stabilized

on two successive ECGs. The Medical Monitor should be notified as soon as possible within 24 hours. Standard-of-care treatment may be instituted per the discretion of the investigator. If a PK sample is not scheduled for that timepoint, an unscheduled PK sample should be obtained. A decision on study drug discontinuation should be made, as described in Section 4.6.2. The investigator should also evaluate the patient for potential concurrent risk factors (e.g., electrolyte abnormalities, co-medications known to prolong the QT interval, severe bradycardia).

Guidelines for management of study treatment in the event in the event of cardiovascular effects in patients are provided in Section 5.1.2. Please refer to the Fenebrutinib Investigator's Brochure for further details.

5.1.1.8 Vascular Inflammation

Vascular inflammation (vasculitis) was observed in dogs administered fenebrutinib at ≥10 mg/kg/day (≥56 μM•hr) in the 4-week toxicity study, and these changes were not completely reversed by the end of the 4-week recovery period. There was no consistent correlation with any clinical biomarkers. However, in the 9-month toxicity study in dogs, no fenebrutinib–related vascular inflammation was observed up to the highest dose of 10 mg/kg/day (36 μM•hr), which is considered to be the NOAEL (AUC) for the canine vascular inflammation findings. This exposure provides a 5-fold safety multiple above the study dose of 200 mg BID (projected $AUC_{0\text{-}24}$ of 6.7 μM•hr). The translatability of these findings to humans is unknown; however, Beagle dogs are susceptible to spontaneous development of polyarteritis syndrome (Snyder et al. 1995) and may be more sensitive to any drug-induced effects. Further, there are several examples of approved therapies for which there is no correlation between the finding of vasculitis in dogs or rats at clinically relevant exposures and adverse outcomes in patients (FDA 2011). Guidelines for management of study treatment in the event of vasculitis in patients are provided in Section 5.1.2. Please refer to the Fenebrutinib Investigator's Brochure for further details.

5.1.1.9 Malignancy

The impact of BTK inhibition on the development of malignancies is not known; however, malignancies have been identified as a potential concern for immunomodulatory agents. Malignancies have been reported in patients with XLA, including lymphoreticular malignancies, gastric and colorectal adenocarcinoma, and squamous cell carcinoma of the lung.

Patients with a history of cancer, including hematologic malignancy and solid tumors, within 10 years before screening will be excluded from the study. Basal or squamous cell carcinoma of the skin that has been excised and is considered cured and in situ carcinoma of the cervix treated with apparent success by curative therapy more than 1 year prior to screening are not exclusionary.

All malignancies are adverse events of special interest and should be reported to the Sponsor in an expedited manner as outlined in Section 5.2.3.

Guidelines for management of study treatment in the event of malignancies in patients are provided in Section 5.1.2. Please refer to the Fenebrutinib Investigator's Brochure for further details.

5.1.2 Management of Patients Who Experience Specific Adverse Events

5.1.2.1 Management of Specific Adverse Events

Guidelines for management of specific adverse events are outlined in Table 5.

Table 5 Guidelines for Management of Patients Who Experience Specific Adverse Events

Event	Action to be Taken [a]
**Infection [b]**	
Serious infection, opportunistic infection, or any infection requiring treatment with an IV antimicrobial agent	Discontinue study treatment and report event as an adverse event of special interest.
Self-limited infections that require treatment	Withhold study treatment during antimicrobial therapy. Study treatment may resume after consultation with the Medical Monitor.
Bleeding	Bleeding events of moderate or greater severity are considered adverse events of special interest and should be reported to the Sponsor in an expedited manner.<br>For serious bleeding events or bleeding events requiring transfusion, radiologic endoscopic, or elective operative intervention, withhold study treatment and consult with the Medical Monitor.
**Gastrointestinal effects**	
Nausea, vomiting, and/or diarrhea	Manage according to site institutional guidelines.<br>Consider administration of study treatment with food as a possible mitigation strategy.
**Malignancy**	
Any malignancy	Discontinue study treatment, with the exception of non-serious local and resectable basal or squamous cell carcinoma of the skin. Report event as an adverse event of special interest to the Sponsor in an expedited manner.
**Hepatotoxicity**	
AST or ALT 3.0–5.0 × ULN	Withhold study treatment and consult with the Medical Monitor.
AST or ALT > 3 × ULN in combination with a total bilirubin > 2 × ULN, of which at least 35% is direct bilirubin, or clinical jaundice	Discontinue study treatment. Report event(s) as adverse event of special interest (Hy's law) to the Sponsor in an expedited manner.

Table 5 Guidelines for Management of Patients Who Experience Specific Adverse Events (cont.)

Event	Action to be Taken [a]
AST or ALT > 5 × ULN	Discontinue study treatment. Any elevation of an AST or ALT > 5 × ULN should be reported as an adverse event of special interest to the Sponsor in an expedited manner.
**Cardiovascular effects**	
Sustained (at least two ECG measurements > 30 minutes apart) QTcF that is > 500 ms or > 60 ms longer than the baseline value	Unless there is a clear alternative cause other than study drug, discontinue study treatment. [c]
Sustained absolute QTcF that is > 515 ms	Unless there is a clear alternative cause other than study drug, discontinue study treatment. [c]
An episode of torsades de pointes or a new ECG finding of clinical concern	Unless there is a clear alternative cause other than study drug, discontinue study treatment. [c]
**Vascular inflammation**	
Vasculitis	Discontinue study treatment and consult with the Medical Monitor.

IV = intravenous; QTcF = QT interval corrected using Fridericia's formula; ULN = upper limit of normal.

Note: "Study treatment" includes study drug (fenebrutinib or placebo).

a Any patient who discontinues study treatment should enter safety follow-up, if possible.

b Appropriate laboratory investigations, including but not limited to cultures, should be performed to establish the etiology of any serious infection.

c In rare circumstances, it may be acceptable to resume study drug, provided that any ECG abnormalities have resolved and that the patient is appropriately monitored. Clinical judgment should be applied.

5.1.2.2 Management of Increases in QT Interval

Study drug should be discontinued in patients who develop any of the following:

- Sustained (at least two ECG measurements > 30 minutes apart) QTcF that is > 500 ms or > 60 ms longer than the baseline value
- Sustained absolute QTcF that is > 515 ms
- An episode of torsades de pointes or a new ECG finding of clinical concern

Of note, if there is a new intraventricular conduction block, the increase in QRS complex duration should be subtracted from the QTcF change, because this represents an increase in QTcF unrelated to alterations in repolarization. Also of note, it is not uncommon to record arrhythmias, such as non-sustained ventricular tachycardia, supraventricular tachycardia, pauses, or atrial fibrillation, in healthy volunteers receiving placebo during periods of extended ECG monitoring. Therefore, it is critical that expert cardiology advice be sought to confirm any ECG changes and to ascertain the likelihood of a drug-induced arrhythmia versus the background occurrence of this arrhythmia. In such a situation, saving all available ECG data is highly suggested.

Management of patients with sustained QTcF prolongation should include close monitoring, with ECGs repeated at least hourly until two successive ECGs show resolution of the findings, correction of any electrolyte abnormalities, and possible discontinuation of other concomitant medications that are known to prolong the QT interval. Consultation with a cardiologist or electrophysiologist is recommended to help in the management of such patients.

5.2 SAFETY PARAMETERS AND DEFINITIONS

Safety assessments will consist of monitoring and recording adverse events, including serious adverse events and adverse events of special interest, performing protocol-specified safety laboratory assessments, measuring protocol-specified vital signs, and conducting other protocol-specified tests that are deemed critical to the safety evaluation of the study.

Certain types of events require immediate reporting to the Sponsor, as outlined in Section 5.4.

5.2.1 Adverse Events

According to the ICH guideline for Good Clinical Practice, an adverse event is any untoward medical occurrence in a clinical investigation subject administered a pharmaceutical product, regardless of causal attribution. An adverse event can therefore be any of the following:

- Any unfavorable and unintended sign (including an abnormal laboratory finding), symptom, or disease temporally associated with the use of a medicinal product, whether or not considered related to the medicinal product
- Any new disease or exacerbation of an existing disease (a worsening in the character, frequency, or severity of a known condition), except as described in Section 5.3.5.10
- Recurrence of an intermittent medical condition (e.g., headache) not present at baseline
- Any deterioration in a laboratory value or other clinical test (e.g., ECG, X-ray) that is associated with symptoms or leads to a change in study treatment or concomitant treatment or discontinuation from study drug
- Adverse events that are related to a protocol-mandated intervention, including those that occur prior to assignment of study treatment (e.g., screening invasive procedures such as biopsies)

5.2.2 Serious Adverse Events (Immediately Reportable to the Sponsor)

A serious adverse event is any adverse event that meets any of the following criteria:

- Is fatal (i.e., the adverse event actually causes or leads to death)

- Is life threatening (i.e., the adverse event, in the view of the investigator, places the patient at immediate risk of death)
    - This does not include any adverse event that had it occurred in a more severe form or was allowed to continue might have caused death.
- Requires or prolongs inpatient hospitalization (see Section 5.3.5.11)
- Results in persistent or significant disability/incapacity (i.e., the adverse event results in substantial disruption of the patient's ability to conduct normal life functions)
- Is a congenital anomaly/birth defect in a neonate/infant born to a mother exposed to study drug
- Is a significant medical event in the investigator's judgment (e.g., may jeopardize the patient or may require medical/surgical intervention to prevent one of the outcomes listed above)

The terms "severe" and "serious" are not synonymous. Severity refers to the intensity of an adverse event (e.g., rated as mild, moderate, or severe; see Section 5.3.3); the event itself may be of relatively minor medical significance (such as severe headache without any further findings).

Severity and seriousness need to be independently assessed for each adverse event recorded on the eCRF.

Serious adverse events are required to be reported by the investigator to the Sponsor immediately (i.e., no more than 24 hours after learning of the event; see Section 5.4.2 for reporting instructions).

5.2.3 Adverse Events of Special Interest (Immediately Reportable to the Sponsor)

Adverse events of special interest are required to be reported by the investigator to the Sponsor immediately (i.e., no more than 24 hours after learning of the event; see Section 5.4.2 for reporting instructions). Adverse events of special interest for this study include the following:

- Any serious infection, any infections requiring IV antimicrobials and any opportunistic infections
- Bleeding events of moderate or greater severity
- All malignancies
- Adverse events of special interest for general drug development
    - A laboratory result of AST or ALT $> 5 \times$ ULN
    - Cases of potential drug-induced liver injury that include an ALT or AST $> 3 \times$ ULN in combination with a total bilirubin $> 2 \times$ ULN, of which at least 35% is direct bilirubin or there is clinical jaundice, as defined by Hy's law (see Section 5.1.1.6)

- Suspected transmission of an infectious agent by the study drug, as defined below:

  Any organism, virus, or infectious particle (e.g., prion protein transmitting transmissible spongiform encephalopathy), pathogenic or non-pathogenic, is considered an infectious agent. A transmission of an infectious agent may be suspected from clinical symptoms or laboratory findings that indicate an infection in a patient exposed to a medicinal product. This term applies only when a contamination of the study drug is suspected.

5.3 METHODS AND TIMING FOR CAPTURING AND ASSESSING SAFETY PARAMETERS

The investigator is responsible for ensuring that all adverse events (see Section 5.2.1 for definition) are recorded on the Adverse Event eCRF and reported to the Sponsor in accordance with instructions provided in this Section and in Section 5.4 – Section 5.6.

For each adverse event recorded on the Adverse Event eCRF, the investigator will make an assessment of seriousness (see Section 5.2.2), severity (see Section 5.3.3), and causality (see Section 5.3.4).

5.3.1 Adverse Event Reporting Period

Investigators will seek information on adverse events at each patient contact. All adverse events, whether reported by the patient or noted by study personnel, will be recorded in the patient's medical record and on the Adverse Event eCRF.

After informed consent has been obtained **but prior to initiation of study drug**, only serious adverse events caused by a protocol-mandated intervention (e.g., invasive procedures such as biopsy sample collection, discontinuation of medications) should be reported (see Section 5.4.2 for instructions for reporting serious adverse events).

After initiation of study drug, all adverse events will be reported until 4 weeks after the last dose of study drug the patients receives. After this period, the investigator should report any serious adverse events that are believed to be related to prior study drug treatment (see Section 5.4.2).

5.3.2 Eliciting Adverse Event Information

A consistent methodology of non-directive questioning should be adopted for eliciting adverse event information at all patient evaluation timepoints. Examples of non-directive questions include the following:

"How have you felt since your last clinic visit?"

"Have you had any new or changed health problems since you were last here?"

5.3.3 Assessment of Severity of Adverse Events

Table 6 provides the adverse event grading scale for severity.

Table 6 Adverse Event Severity Grading Scale

Severity	Description
Mild	Discomfort noticed, but no disruption of normal daily activity
Moderate	Discomfort sufficient to reduce or affect normal daily activity
Severe	Incapacitating with inability to work or to perform normal daily activity

Note: Regardless of severity, some events may also meet seriousness criteria. Refer to definition of a serious adverse event (see Section 5.2.2).

5.3.4 Assessment of Causality of Adverse Events

Investigators should use their knowledge of the patient, the circumstances surrounding the event, and an evaluation of any potential alternative causes to determine whether an adverse event is considered to be related to the study drug, indicating "yes" or "no" accordingly. The following guidance should be taken into consideration (see also Table 7):

- Temporal relationship of event onset to the initiation of study drug
- Course of the event, with special consideration of the effects of dose reduction, discontinuation of study drug, or reintroduction of study drug (as applicable)
- Known association of the event with the study drug or with similar treatments
- Known association of the event with the disease under study
- Presence of risk factors in the patient or use of concomitant medications known to increase the occurrence of the event
- Presence of non-treatment–related factors that are known to be associated with the occurrence of the event

Table 7 Causal Attribution Guidance

Is the adverse event suspected to be caused by the study drug on the basis of facts, evidence, science-based rationales, and clinical judgment?	
YES	There is a plausible temporal relationship between the onset of the adverse event and administration of the study drug, and the adverse event cannot be readily explained by the patient's clinical state, intercurrent illness, or concomitant therapies; and/or the adverse event follows a known pattern of response to the study drug; and/or the adverse event abates or resolves upon discontinuation of the study drug or dose reduction and, if applicable, reappears upon re-challenge.
NO	An adverse event will be considered related, unless it fulfills the criteria specified below.<br>Evidence exists that the adverse event has an etiology other than the study drug (e.g., preexisting medical condition, underlying disease, intercurrent illness, or concomitant medication); and/or the adverse event has no plausible temporal relationship to administration of the study drug (e.g., cancer diagnosed 2 days after first dose of study drug).

For patients receiving combination therapy, causality will be assessed individually for each protocol-mandated therapy.

5.3.5 Procedures for Recording Adverse Events

Investigators should use correct medical terminology/concepts when recording adverse events on the Adverse Event eCRF. Avoid colloquialisms and abbreviations.

Only one adverse event term should be recorded in the event field on the Adverse Event eCRF.

5.3.5.1 Diagnosis versus Signs and Symptoms

A diagnosis (if known) should be recorded on the Adverse Event eCRF rather than individual signs and symptoms (e.g., record only liver failure or hepatitis rather than jaundice, asterixis, and elevated transaminases). However, if a constellation of signs and/or symptoms cannot be medically characterized as a single diagnosis or syndrome at the time of reporting, each individual event should be recorded on the Adverse Event eCRF. If a diagnosis is subsequently established, all previously reported adverse events based on signs and symptoms should be nullified and replaced by one adverse event report based on the single diagnosis, with a starting date that corresponds to the starting date of the first symptom of the eventual diagnosis.

5.3.5.2 Adverse Events That Are Secondary to Other Events

In general, adverse events that are secondary to other events (e.g., cascade events or clinical sequelae) should be identified by their primary cause, with the exception of severe or serious secondary events. A medically significant secondary adverse event that is separated in time from the initiating event should be recorded as an independent event on the Adverse Event eCRF. For example:

- If vomiting results in mild dehydration with no additional treatment in a healthy adult, only vomiting should be reported on the eCRF.
- If vomiting results in severe dehydration, both events should be reported separately on the eCRF.
- If a severe GI hemorrhage leads to renal failure, both events should be reported separately on the eCRF.
- If dizziness leads to a fall and consequent fracture, all three events should be reported separately on the eCRF.
- If neutropenia is accompanied by an infection, both events should be reported separately on the eCRF.

All adverse events should be recorded separately on the Adverse Event eCRF if it is unclear as to whether the events are associated.

5.3.5.3 Persistent or Recurrent Adverse Events

A persistent adverse event is one that extends continuously, without resolution, between patient evaluation timepoints. Such events should be recorded only once on the Adverse Event eCRF. The initial severity (intensity or grade) of the event will be recorded at the time the event is first reported. If a persistent adverse event becomes

more severe, the most extreme severity should also be recorded on the Adverse Event eCRF. If the event becomes serious, it should be reported to the Sponsor immediately (i.e., no more than 24 hours after learning that the event became serious; see Section 5.4.2 for reporting instructions). The Adverse Event eCRF should be updated by changing the event from "non-serious" to "serious," providing the date that the event became serious and completing all data fields related to serious adverse events.

A recurrent adverse event is one that resolves between patient evaluation timepoints and subsequently recurs. Each recurrence of an adverse event should be recorded as a separate event on the Adverse Event eCRF.

5.3.5.4 Abnormal Laboratory Values

Not every laboratory abnormality qualifies as an adverse event. A laboratory test result must be reported as an adverse event if it meets any of the following criteria:

- Is accompanied by clinical symptoms
- Results in a change in study treatment (e.g., dosage modification, treatment interruption, or treatment discontinuation)
- Results in a medical intervention (e.g., potassium supplementation for hypokalemia) or a change in concomitant therapy
- Is clinically significant in the investigator’s judgment

It is the investigator’s responsibility to review all laboratory findings. Medical and scientific judgment should be exercised in deciding whether an isolated laboratory abnormality should be classified as an adverse event.

If a clinically significant laboratory abnormality is a sign of a disease or syndrome (e.g., alkaline phosphatase and bilirubin 5 × ULN associated with cholestasis), only the diagnosis (i.e., cholestasis) should be recorded on the Adverse Event eCRF.

If a clinically significant laboratory abnormality is not a sign of a disease or syndrome, the abnormality itself should be recorded on the Adverse Event eCRF, along with a descriptor indicating whether the test result is above or below the normal range (e.g., "elevated potassium," as opposed to "abnormal potassium"). If the laboratory abnormality can be characterized by a precise clinical term per standard definitions, the clinical term should be recorded as the adverse event. For example, an elevated serum potassium level of 7.0 mEq/L should be recorded as "hyperkalemia."

Observations of the same clinically significant laboratory abnormality from visit to visit should only be recorded once on the Adverse Event eCRF (see Section 5.3.5.3 for details on recording persistent adverse events).

5.3.5.5 Abnormal Vital Sign Values

Not every vital sign abnormality qualifies as an adverse event. A vital sign result must be reported as an adverse event if it meets any of the following criteria:

- Is accompanied by clinical symptoms
- Results in a change in study treatment (e.g., dosage modification, treatment interruption, or treatment discontinuation)
- Results in a medical intervention or a change in concomitant therapy
- Is clinically significant in the investigator's judgment

It is the investigator's responsibility to review all vital sign findings. Medical and scientific judgment should be exercised in deciding whether an isolated vital sign abnormality should be classified as an adverse event.

If a clinically significant vital sign abnormality is a sign of a disease or syndrome (e.g., high blood pressure), only the diagnosis (i.e., hypertension) should be recorded on the Adverse Event eCRF.

Observations of the same clinically significant vital sign abnormality from visit to visit should only be recorded once on the Adverse Event eCRF (see Section 5.3.5.3 for details on recording persistent adverse events).

5.3.5.6 Abnormal Liver Function Tests

The finding of an elevated ALT or AST ($>3\times$ baseline value) in combination with either an elevated total bilirubin ($>2\times$ ULN) or clinical jaundice in the absence of cholestasis or other causes of hyperbilirubinemia is considered to be an indicator of severe liver injury (as defined by Hy's law). Therefore, investigators must report as an adverse event the occurrence of either of the following:

- Treatment-emergent ALT or AST $>3\times$ baseline value in combination with total bilirubin $>2\times$ ULN (of which $\geq$35% is direct bilirubin)
- Treatment-emergent ALT or AST $>3\times$ baseline value in combination with clinical jaundice

The most appropriate diagnosis or (if a diagnosis cannot be established) the abnormal laboratory values should be recorded on the Adverse Event eCRF (see Section 5.3.5.2) and reported to the Sponsor immediately (i.e., no more than 24 hours after learning of the event), either as a serious adverse event or an adverse event of special interest (see Section 5.4.2).

5.3.5.7 Deaths

All deaths that occur during the protocol-specified adverse event reporting period (see Section 5.3.1), regardless of relationship to study drug, must be recorded on the Adverse Event eCRF and immediately reported to the Sponsor (see Section 5.4.2). This includes death attributed to progression of chronic spontaneous urticaria.

Death should be considered an outcome and not a distinct event. The event or condition that caused or contributed to the fatal outcome should be recorded as the single medical concept on the Adverse Event eCRF. Generally, only one such event should be reported. If the cause of death is unknown and cannot be ascertained at the time of reporting, **"unexplained death"** should be recorded on the Adverse Event eCRF. If the cause of death later becomes available (e.g., after autopsy), "unexplained death" should be replaced by the established cause of death. The term **"sudden death"** should not be used unless combined with the presumed cause of death (e.g., "sudden cardiac death").

If the death is attributed to angioedema of CSU, "chronic spontaneous urticaria angioedema" should be recorded on the Adverse Event eCRF.

Deaths that occur after the adverse event reporting period should be reported as described in Section 5.6.

5.3.5.8 Preexisting Medical Conditions

A preexisting medical condition is one that is present at the screening visit for this study. Such conditions should be recorded on the General Medical History and Baseline Conditions eCRF.

A preexisting medical condition should be recorded as an adverse event only if the frequency, severity, or character of the condition worsens during the study. When recording such events on the Adverse Event eCRF, it is important to convey the concept that the preexisting condition has changed by including applicable descriptors (e.g., "more frequent headaches").

5.3.5.9 Lack of Efficacy or Worsening of Chronic Spontaneous Urticaria

Medical occurrences or symptoms of deterioration that are anticipated as part of CSU should be recorded as an adverse event if judged by the investigator to have unexpectedly worsened in severity or frequency or changed in nature compared to baseline at any time during the study. When recording an unanticipated worsening of CSU on the Adverse Event eCRF, it is important to convey the concept that the condition has changed by including applicable descriptors (e.g., "accelerated CSU").

5.3.5.10 Hospitalization or Prolonged Hospitalization

Any adverse event that results in hospitalization (i.e., inpatient admission to a hospital) or prolonged hospitalization should be documented and reported as a serious adverse event (per the definition of serious adverse event in Section 5.2.2), except as outlined below.

An event that leads to hospitalization under the following circumstances should not be reported as an adverse event or a serious adverse event:

- Hospitalization for respite care

- Hospitalization for a preexisting condition, provided that all of the following criteria are met:
  - The hospitalization was planned prior to the study or was scheduled during the study when elective surgery became necessary because of the expected normal progression of the disease
  - The patient has not experienced an adverse event

An event that leads to hospitalization under the following circumstances is not considered to be a serious adverse event, but should be reported as an adverse event instead:

- Hospitalization that was necessary because of patient requirement for outpatient care outside of normal outpatient clinic operating hours. For this scenario, record the underlying medical condition which resulted in hospitalization on the Adverse Event eCRF.

5.3.5.11 Adverse Events Associated with an Overdose or Error in Drug Administration

An overdose is the accidental or intentional use of a drug in an amount higher than the dose being studied. An overdose or incorrect administration of study treatment is not itself an adverse event, but it may result in an adverse event. All adverse events associated with an overdose or incorrect administration of study drug should be recorded on the Adverse Event eCRF. If the associated adverse event fulfills seriousness criteria, the event should be reported to the Sponsor immediately (i.e., no more than 24 hours after learning of the event; see Section 5.4.2).

No safety data related to overdosing of GDC-0853 are available.

5.3.5.12 Patient-Reported Outcome Data

Adverse event reports will not be derived from PRO data by the Sponsor, and safety analyses will not be performed using PRO data. However, if any PRO responses suggestive of a possible adverse event are identified during site review of the PRO data, the investigator will determine whether the criteria for an adverse event have been met and, if so, will report the event on the Adverse Event eCRF.

5.4 IMMEDIATE REPORTING REQUIREMENTS FROM INVESTIGATOR TO SPONSOR

Certain events require immediate reporting to allow the Sponsor to take appropriate measures to address potential new risks in a clinical trial. The investigator must report such events to the Sponsor immediately; under no circumstances should reporting take place more than 24 hours after the investigator learns of the event. The following is a list of events that the investigator must report to the Sponsor within 24 hours after learning of the event, regardless of relationship to study drug:

- Serious adverse events (see Section 5.4.2 for further details)

- Adverse events of special interest (see Section 5.4.2 for further details)
- Pregnancies (see Section 5.4.3 for further details)

The investigator must report new significant follow-up information for these events to the Sponsor immediately (i.e., no more than 24 hours after becoming aware of the information). New significant information includes the following:

- New signs or symptoms or a change in the diagnosis
- Significant new diagnostic test results
- Change in causality based on new information
- Change in the event's outcome, including recovery
- Additional narrative information on the clinical course of the event

Investigators must also comply with local requirements for reporting serious adverse events to the local health authority and Institutional Review Board or Ethics Committee (IRB/EC).

5.4.1 Emergency Medical Contacts

Medical Monitor Contact Information

Medical Monitor contact information:

Medical Monitor: [redacted]

Telephone No.: [redacted]

5.4.2 Reporting Requirements for Serious Adverse Events and Adverse Events of Special Interest

5.4.2.1 Events That Occur prior to Study Drug Initiation

After informed consent has been obtained but prior to initiation of study drug, only serious adverse events caused by a protocol-mandated intervention should be reported. The Serious Adverse Event/Adverse Event of Special Interest Reporting Form provided to investigators should be completed and submitted to the Sponsor or its designee immediately (i.e., no more than 24 hours after learning of the event), either by faxing or by scanning and emailing the form using the fax number or email address provided to investigators.

5.4.2.2 Events That Occur after Study Drug Initiation

After initiation of study drug, serious adverse events and adverse events of special interest will be reported until 4 weeks after the last dose of study drug. Investigators should record all case details that can be gathered immediately (i.e., within 24 hours after learning of the event) on the Adverse Event eCRF and submit the report via the electronic data capture (EDC) system. A report will be generated and sent to Safety Risk Management by the EDC system.

In the event that the EDC system is unavailable, the Serious Adverse Event/Adverse Event of Special Interest Reporting Form provided to investigators should be completed and submitted to the Sponsor or its designee immediately (i.e., no more than 24 hours after learning of the event), either by faxing or by scanning and emailing the form using the fax number or email address provided to investigators. Once the EDC system is available, all information will need to be entered and submitted via the EDC system.

Instructions for reporting serious adverse events that occur >4 weeks after the last dose of study treatment are provided in Section 5.6.

5.4.3 Reporting Requirements for Pregnancies

5.4.3.1 Pregnancies in Female Patients

Female patients of childbearing potential will be instructed to immediately inform the investigator if they become pregnant during the study or within 4 weeks after the last dose of study drug. A Clinical Trial Pregnancy Reporting Form should be completed and submitted to the Sponsor or its designee immediately (i.e., no more than 24 hours after learning of the pregnancy), either by faxing or by scanning and emailing the form using the fax number or email address provided to investigators. Pregnancy should not be recorded on the Adverse Event eCRF. The investigator should discontinue study drug and counsel the patient, discussing the risks of the pregnancy and the possible effects on the fetus. Monitoring of the patient should continue until conclusion of the pregnancy. Any serious adverse events associated with the pregnancy (e.g., an event in the fetus, an event in the mother during or after the pregnancy, or a congenital anomaly/birth defect in the child) should be reported on the Adverse Event eCRF. In addition, the investigator will submit a Clinical Trial Pregnancy Reporting Form when updated information on the course and outcome of the pregnancy becomes available.

5.4.3.2 Pregnancies in Female Partners of Male Patients

Male patients will be instructed through the Informed Consent Form to immediately inform the investigator if their partner becomes pregnant during the study or within 4 weeks after the last dose of study drug. A Clinical Trial Pregnancy Reporting Form should be completed and submitted to the Sponsor or its designee immediately (i.e., no more than 24 hours after learning of the pregnancy), either by faxing or by scanning and emailing the form using the fax number or email address provided to investigators. Attempts should be made to collect and report details of the course and outcome of any pregnancy in the partner of a male patient exposed to study drug. When permitted by the site, the pregnant partner would need to sign an Authorization for Use and Disclosure of Pregnancy Health Information to allow for follow-up on her pregnancy. If the authorization has been signed, the investigator should submit a Clinical Trial Pregnancy Reporting Form when updated information on the course and outcome of the pregnancy becomes available. An investigator who is contacted by the male patient or his pregnant partner may provide information on the risks of the pregnancy and the possible effects on the fetus, to support an informed decision in cooperation with the treating physician and/or obstetrician.

5.4.3.3 Congenital Anomalies/Birth Defects and Abortions

Any congenital anomaly/birth defect in a child born to a female patient exposed to study drug or the female partner of a male patient exposed to study drug should be classified as a serious adverse event, recorded on the Adverse Event eCRF, and reported to the Sponsor immediately (i.e., no more than 24 hours after learning of the event; see Section 5.4.2). Any abortion should be reported in the same fashion (as the Sponsor considers abortions to be medically significant).

5.5 FOLLOW-UP OF PATIENTS AFTER ADVERSE EVENTS

5.5.1 Investigator Follow-Up

The investigator should follow each adverse event until the event has resolved to baseline grade or better, the event is assessed as stable by the investigator, the patient is lost to follow-up, or the patient withdraws consent. Every effort should be made to follow all serious adverse events considered to be related to study drug or trial-related procedures until a final outcome can be reported.

During the study period, resolution of adverse events (with dates) should be documented on the Adverse Event eCRF and in the patient's medical record to facilitate source data verification.

All pregnancies reported during the study should be followed until pregnancy outcome.

5.5.2 Sponsor Follow-Up

For serious adverse events, adverse events of special interest, and pregnancies, the Sponsor or a designee may follow up by telephone, fax, email, and/or a monitoring visit to obtain additional case details and outcome information (e.g., from hospital discharge summaries, consultant reports, autopsy reports) in order to perform an independent medical assessment of the reported case.

5.6 ADVERSE EVENTS THAT OCCUR AFTER THE ADVERSE EVENT REPORTING PERIOD

The Sponsor should be notified if the investigator becomes aware of any serious adverse event that occurs after the end of the adverse event reporting period (defined as 4 weeks after the last dose of study drug; see Section 5.3.1), if the event is believed to be related to prior study drug treatment. These events should be reported through use of the Adverse Event eCRF. However, if the EDC system is not available, the investigator should report these events directly to the Sponsor or its designee, either by faxing or by scanning and emailing the Serious Adverse Event/Adverse Event of Special Interest Reporting Form using the fax number or email address provided to investigators.

5.7 EXPEDITED REPORTING TO HEALTH AUTHORITIES, INVESTIGATORS, INSTITUTIONAL REVIEW BOARDS, AND ETHICS COMMITTEES

The Sponsor will promptly evaluate all serious adverse events and adverse events of special interest against cumulative product experience to identify and expeditiously communicate possible new safety findings to investigators, IRBs, ECs, and applicable health authorities based on applicable legislation.

To determine reporting requirements for single adverse event cases, the Sponsor will assess the expectedness of these events using the following reference document:

- Fenebrutinib Investigator's Brochure

The Sponsor will compare the severity of each event and the cumulative event frequency reported for the study with the severity and frequency reported in the applicable reference document.

Reporting requirements will also be based on the investigator's assessment of causality and seriousness, with allowance for upgrading by the Sponsor as needed.

6. STATISTICAL CONSIDERATIONS AND ANALYSIS PLAN

The primary and secondary efficacy analyses will be based on a modified intent-to-treat (mITT) approach. All patients who received at least one dose of study drug will be included in the mITT population, with patients grouped according to the treatment assigned at randomization. Safety analyses will be conducted on the safety-evaluable population, defined as all patients who received at least one dose of study drug, with patients grouped according to the actual treatment received.

For each cohort, the final analysis of data from the 8-week, placebo-controlled period will be performed when the following two criteria have been met: 1) All patients in the cohort have either completed the Day 57 visit or discontinued from the placebo-controlled period prematurely and 2) all data from the placebo-controlled period in the cohort are in the database and have been cleaned and verified. Patients and study site personnel will remain blinded to the individual treatment assignment until after the study is completed (i.e., after all patients in both cohorts have either completed the safety follow-up period or discontinued early from the study), the database is locked, and the study analyses are final for both cohorts.

The focus of the trial is estimation and generation of hypotheses to be confirmed in future trials; therefore, Type I error control is not addressed.

6.1 DETERMINATION OF SAMPLE SIZE

6.1.1 Cohort 1: Pilot Assessment

The purpose of this cohort is to evaluate the efficacy of fenebrutinib at 200 mg PO BID compared with placebo in improving the UAS7. Point and interval estimates of the

change from baseline of the UAS7 within each treatment group as well as of the difference in change from baseline of the UAS7 between treatment groups will be presented.

The cohort will enroll approximately 45 patients. Patients will be randomized in a 2:1 ratio to receive treatment with either fenebrutinib or placebo. The sample size of approximately 30 patients in the fenebrutinib arm and 15 patients in the placebo arm provides approximately 80% power to detect an 11-point difference in the UAS7 change from baseline at Day 57 between treatment groups under the following assumptions:

- The absolute change from baseline at Day 57 is normally distributed with a standard deviation of 13.
- Two-sided alpha is 0.10.
- Dropout rate at Day 57 is 10%, leading to a 10% loss of information.

6.1.2 Cohort 2: Dose-Ranging Assessment

The purpose of this cohort is estimation and hypothesis generation regarding the dose-ranging effects of fenebrutinib compared with placebo in improving the UAS7. Point and interval estimates of the change from baseline of the UAS7 within each treatment group as well as of the difference in change from baseline of the UAS7 between treatment groups vs placebo will be presented.

The cohort will enroll approximately 120 patients. Patients will be randomly allocated in a 1:1:1:1 ratio to receive treatment with one of three dose levels of fenebrutinib or placebo. The sample size of approximately 30 in each arm provides approximately 90% power to detect an 11-point difference in the UAS7 change from baseline at Day 57 between treatment groups, under the following assumptions:

- The absolute change from baseline at Day 57 is normally distributed with a standard deviation of 13
- Two-sided alpha is 0.10
- Dropout rate at Day 57 is 10%, leading to a 10% loss of information.

The overall sample size may be adjusted depending on the outcome of a planned interim analysis for Cohort 1 (see Section 6.8), which will include an evaluation of these assumptions.

6.2 SUMMARIES OF CONDUCT OF STUDY

The number of patients who enroll, discontinue, or complete the study will be summarized. Reasons for premature study withdrawal will be listed and summarized. Enrollment and major protocol deviations will be listed and evaluated for their potential effects on the interpretation of study results.

6.3 SUMMARIES OF TREATMENT GROUP COMPARABILITY

Baseline demographics, disease characteristics, and exposure to study drug will be summarized by treatment group using descriptive statistics. For categorical endpoints, the descriptive statistics will include counts and proportions. For continuous endpoints, the descriptive statistics will include the number of observations, mean, standard deviation, median, minimum, and maximum.

6.4 EFFICACY ANALYSES

Statistical analysis will be conducted for each cohort separately. Statistical testing will be performed as a two-sided test with a statistical significance level of 0.10. No multiplicity adjustments will be performed to control overall Type I error, and positive tests will be viewed as hypothesis generating rather than confirmatory.

Continuous longitudinal efficacy endpoints will be analyzed using a mixed model for repeated measures (MMRM) and descriptive statistics as appropriate. An unstructured covariance pattern will be specified to model the within-subject errors. Parameters will be estimated with the use of restricted maximum likelihood, and the Kenward-Roger method will be used for calculating the denominator degrees of freedom. The MMRM method assumes that data are missing at random. That is, MMRM assumes that given the statistical model and given the observed values of the endpoint, missing data are independent of the unobserved values (O'Kelly and Ratitch 2014). High correlation between successive observations on a subject allows data from subjects who dropped out to make a contribution to estimation of the effects at the final timepoint.

All MMRM models will include country, treatment group, visit, and visit by treatment group interaction as covariates.

Time-to-event endpoints will be analyzed using a Cox proportional hazards model. Categorical endpoints will be analyzed using an appropriate statistical method, such as Cochran-Mantel-Haenszel test or Fisher's exact test.

Details of all statistical methods will be provided in the Data Analysis Plans (DAP).

6.4.1 <u>Primary Efficacy Endpoint</u>

The primary efficacy endpoint is the change from baseline in the UAS7 at Day 57 (Week 8).

The UAS is to be recorded twice daily (i.e., morning and evening) using an eDiary that will be provided to each patient. Scores ranging from 0 (none) to 3 (severe) will be entered for each of the two UAS domains consisting of number of wheals (hives) and intensity of pruritus (itch) resulting in a total possible score of 0 to 6 (see Table 2). The daily UAS is calculated as the average of the morning and evening scores. When either the morning or evening score is missing, the non-missing UAS for that day (morning or evening) will be used as the daily UAS, and when both the morning and evening UAS

are missing, the daily UAS will be deemed missing. The UAS7 is the sum of the daily UAS over the 7 days prior to the timepoint of interest. The baseline UAS7 will be calculated as the sum of daily UAS values over the week (7 days) prior to Day 1.

When one or more daily UAS values is missing, over the week prior to a timepoint of interest, rules for deriving UAS7 will be as follows:

- If a patient has at least 4 completed daily scores on the UAS (both domains) over the 7 days prior to the timepoint of interest, the UAS7 will be defined as the average of the available daily scores, multiplied by 7.
- If a patient has fewer than 4 completed daily scores on the UAS over the 7 days prior to the timepoint of interest, then the UAS7 will be considered missing for that timepoint.

The primary endpoint will be analyzed using a MMRM model as specified in Section 6.4. Additional model covariates will include baseline UAS7 and its interaction with visit. Missing data will be handled by the model under the missing-at-random assumption without need for imputation.

As a sensitivity analysis, an analysis-of-covariance (ANCOVA) model adjusted for country and baseline UAS7 will be fit. Missing Day 57 data will be imputed by last observation carried forward.

6.4.2 Secondary Efficacy Endpoints

The secondary efficacy endpoints are as follows:

- Proportion of patients who are well controlled (UAS7 $\leq$6) at Day 57
- Change from baseline in the UAS7 at Day 29 (Week 4)

These endpoints will be analyzed as specified in Section 6.4.

6.4.3 Exploratory Efficacy Endpoints

The exploratory efficacy endpoints include the following:

- Change from baseline in the weekly itch score at Day 29
- Change from baseline in the weekly itch score at Day 57
- Change from baseline in the weekly hives score at Day 57
- Proportion of patients who are well controlled (UAS7 $\leq$6) at Day 29
- Proportion of patients who achieve complete response (UAS7 $=$0) at Day 29
- Proportion of patients who achieve complete response (UAS7 $=$0) at Day 57
- Proportion of patients achieving MID in UAS7 at Day 57 (reduction from baseline $\geq$11 points)
- Proportion of patients achieving MID in the weekly itch score at Day 57 (reduction from baseline $\geq$5 points)

- Time to achieving MID in UAS7 (reduction from baseline ≥ 11 points)
- [redacted]

Further details on the analysis of exploratory endpoints will be included in the DAP.

6.5 SAFETY ANALYSES

Adverse events will be graded according to the adverse event severity grading scale described in Section 5.3.3. Summaries of adverse events, serious adverse events, deaths, adverse events of special interest, adverse events that lead to discontinuation, ECG findings, laboratory test results, and vital sign measurements will be presented.

6.6 PHARMACOKINETIC ANALYSES

The PK endpoints are as follows:

- Plasma concentration data for fenebrutinib will be tabulated and summarized by visits. Descriptive summary statistics will include the arithmetic mean, median, range, SD, and coefficient of variation, as appropriate

[redacted]

The PK analyses will include patients with sufficient data to enable estimation of key parameters (e.g., AUC, t_{max}, C_{max}, $t_{1/2}$), with patients grouped according to treatment received. [redacted]

Additional PK analyses will be conducted as appropriate.

[redacted]

[redacted]

6.8 INTERIM ANALYSIS

6.8.1 Cohort 1: Planned Interim Analysis

An interim analysis will be performed after approximately 33 patients have completed their 8–week treatment period. The purpose of this analysis is to assess the efficacy of the 200-mg fenebrutinib BID daily arm compared with the placebo, to guide internal decision-making around issues such as ungating of Cohort 2, adequacy of sample sizes for safety and/or efficacy analyses in Cohort 2, or to inform further development decisions. Summaries of safety and efficacy data by treatment groups will be prepared and reviewed by Sponsor personnel who do not have direct contact with investigational staff, monitors, and patients. Further details of the interim analysis will be specified in the DAP prior to the conduct of the interim analysis. Access to treatment assignment information will follow the Sponsor's standard procedures.

6.8.2 Cohort 2: Optional Interim Analysis

Given the hypothesis-generating nature of this study, the Sponsor may choose to conduct up to two interim efficacy analyses. The decision to conduct an optional interim analysis and the timing of the analysis will be documented in the Sponsor's trial master file prior to the conduct of the interim analysis. The interim analysis will be performed and interpreted by members of the IMC. Access to treatment assignment information will follow the Sponsor's standard procedures.

7. DATA COLLECTION AND MANAGEMENT

7.1 DATA QUALITY ASSURANCE

The Sponsor will be responsible for data management of this study, including quality checking of the data. Data entered manually will be collected via EDC through use of eCRFs. Sites will be responsible for data entry into the EDC system. In the event of discrepant data, the Sponsor will request data clarification from the sites, which the sites will resolve electronically in the EDC system.

The Sponsor will produce an EDC Study Specification document that describes the quality checking to be performed on the data. Central laboratory data will be sent directly to the Sponsor, using the Sponsor's standard procedures to handle and process the electronic transfer of these data.

eCRFs and correction documentation will be maintained in the EDC system's audit trail. System backups for data stored by the Sponsor and records retention for the study data will be consistent with the Sponsor's standard procedures.

PRO data will be collected through the use of an electronic device provided by a vendor. The device is designed for entry of data in a way that is attributable, secure, and accurate, in compliance with U.S. Food and Drug Administration (FDA) regulations for electronic records (21 CFR Part 11). The electronic data are available for view access only via secure access to a web server method. Only identified and trained users may view the data, and their actions become part of the audit trail. The Sponsor will have view access only. System backups for data stored by the Sponsor and records retention for the study data will be consistent with the Sponsor's standard procedures.

7.2 ELECTRONIC CASE REPORT FORMS

eCRFs are to be completed through use of a Sponsor-designated EDC system. Sites will receive training and have access to a manual for appropriate eCRF completion. eCRFs will be submitted electronically to the Sponsor and should be handled in accordance with instructions from the Sponsor.

All eCRFs should be completed by designated, trained site staff. eCRFs should be reviewed and electronically signed and dated by the investigator or a designee.

At the end of the study, the investigator will receive patient data for his or her site in a readable format on a compact disc that must be kept with the study records. Acknowledgement of receipt of the compact disc is required.

7.3 ELECTRONIC PATIENT-REPORTED OUTCOME DATA

Patients will use an electronic device to capture PRO data. The data will be transmitted via wireless or web automatically after entry or uploaded by site staff at the appropriate frequency to a centralized database maintained by the electronic device vendor.

Once the study is complete, the data, audit trail, and trial and system documentation will be archived. The investigator will receive patient data for the site in both human- and machine-readable formats on an archival-quality compact disc that must be kept with the study records as source data. Acknowledgement of receipt of the compact disc is required. In addition, the Sponsor will receive all data in a machine-readable format on a compact disc.

7.4 SOURCE DATA DOCUMENTATION

Study monitors will perform ongoing source data verification to confirm that critical protocol data (i.e., source data) entered into the eCRFs by authorized site personnel are accurate, complete, and verifiable from source documents.

Source documents (paper or electronic) are those in which patient data are recorded and documented for the first time. They include, but are not limited to, hospital records, clinical and office charts, laboratory notes, memoranda, patient-reported outcomes, evaluation checklists, pharmacy dispensing records, recorded data from automated instruments, copies of transcriptions that are certified after verification as being accurate

and complete, microfiche, photographic negatives, microfilm or magnetic media, X-rays, patient files, and records kept at pharmacies, laboratories, and medico-technical departments involved in a clinical trial.

Before study initiation, the types of source documents that are to be generated will be clearly defined in the Trial Monitoring Plan. This includes any protocol data to be entered directly into the eCRFs (i.e., no prior written or electronic record of the data) and considered source data.

Source documents that are required to verify the validity and completeness of data entered into the eCRFs must not be obliterated or destroyed and must be retained per the policy for retention of records described in Section 7.6.

To facilitate source data verification, the investigators and institutions must provide the Sponsor direct access to applicable source documents and reports for trial-related monitoring, Sponsor audits, and IRB/EC review. The study site must also allow inspection by applicable health authorities.

7.5 USE OF COMPUTERIZED SYSTEMS

When clinical observations are entered directly into a study site's computerized medical record system (i.e., in lieu of original hardcopy records), the electronic record can serve as the source document if the system has been validated in accordance with health authority requirements pertaining to computerized systems used in clinical research. An acceptable computerized data collection system allows preservation of the original entry of data. If original data are modified, the system should maintain a viewable audit trail that shows the original data as well as the reason for the change, name of the person making the change, and date of the change.

7.6 RETENTION OF RECORDS

Records and documents pertaining to the conduct of this study and the distribution of IMP, including eCRFs, electronic PRO data (if applicable), Informed Consent Forms, laboratory test results, and medication inventory records, must be retained by the Principal Investigator for at least 15 years after completion or discontinuation of the study or for the length of time required by relevant national or local health authorities, whichever is longer. After that period of time, the documents may be destroyed, subject to local regulations.

No records may be disposed of without the written approval of the Sponsor. Written notification should be provided to the Sponsor prior to transferring any records to another party or moving them to another location.

Roche will retain study data for 25 years after the final Clinical Study Report has been completed or for the length of time required by relevant national or local health authorities, whichever is longer.

8. ETHICAL CONSIDERATIONS

8.1 COMPLIANCE WITH LAWS AND REGULATIONS

This study will be conducted in full conformance with the ICH E6 guideline for Good Clinical Practice and the principles of the Declaration of Helsinki, or the laws and regulations of the country in which the research is conducted, whichever affords the greater protection to the individual. The study will comply with the requirements of the ICH E2A guideline (Clinical Safety Data Management: Definitions and Standards for Expedited Reporting). Studies conducted in the United States or under a U.S. Investigational New Drug (IND) application will comply with U.S. FDA regulations and applicable local, state, and federal laws. Studies conducted in the European Union or European Economic Area will comply with the E.U. Clinical Trial Directive (2001/20/EC).

8.2 INFORMED CONSENT

The Sponsor's sample Informed Consent Form (and ancillary sample Informed Consent Forms, if applicable) will be provided to each site. If applicable, it will be provided in a certified translation of the local language. The Sponsor or its designee must review and approve any proposed deviations from the Sponsor's sample Informed Consent Forms or any alternate consent forms proposed by the site (collectively, the "Consent Forms") before IRB/EC submission. The final IRB/EC–approved Consent Forms must be provided to the Sponsor for health authority submission purposes according to local requirements.

If applicable, the Informed Consent Form will contain separate Sections for any optional procedures. The investigator or authorized designee will explain to each patient the objectives, methods, and potential risks associated with each optional procedure. Patients will be told that they are free to refuse to participate and may withdraw their consent at any time for any reason. A separate, specific signature will be required to document a patient's agreement to participate in optional procedures. Patients who decline to participate will not provide a separate signature.

The Consent Forms must be signed and dated by the patient or the patient's legally authorized representative before his or her participation in the study. The case history or clinical records for each patient shall document the informed consent process and that written informed consent was obtained prior to participation in the study.

The Consent Forms should be revised whenever there are changes to study procedures or when new information becomes available that may affect the willingness of the patient to participate. The final revised IRB/EC-approved Consent Forms must be provided to the Sponsor for health authority submission purposes.

Patients must be re-consented to the most current version of the Consent Forms (or to a significant new information/findings addendum in accordance with applicable laws and IRB/EC policy) during their participation in the study. For any updated or revised

Consent Forms, the case history or clinical records for each patient shall document the informed consent process and that written informed consent was obtained using the updated/revised Consent Forms for continued participation in the study.

A copy of each signed Consent Form must be provided to the patient or the patient's legally authorized representative. All signed and dated Consent Forms must remain in each patient's study file or in the site file and must be available for verification by study monitors at any time.

8.3 INSTITUTIONAL REVIEW BOARD OR ETHICS COMMITTEE

This protocol, the Informed Consent Forms, any information to be given to the patient, and relevant supporting information must be submitted to the IRB/EC by the Principal Investigator and reviewed and approved by the IRB/EC before the study is initiated. In addition, any patient recruitment materials must be approved by the IRB/EC.

The Principal Investigator is responsible for providing written summaries of the status of the study to the IRB/EC annually or more frequently in accordance with the requirements, policies, and procedures established by the IRB/EC. Investigators are also responsible for promptly informing the IRB/EC of any protocol amendments (see Section 9.6).

In addition to the requirements for reporting all adverse events to the Sponsor, investigators must comply with requirements for reporting serious adverse events to the local health authority and IRB/EC. Investigators may receive written IND safety reports or other safety-related communications from the Sponsor. Investigators are responsible for ensuring that such reports are reviewed and processed in accordance with health authority requirements and the policies and procedures established by their IRB/EC, and archived in the site's study file.

8.4 CONFIDENTIALITY

The Sponsor maintains confidentiality standards by coding each patient enrolled in the study through assignment of a unique patient identification number. This means that patient names are not included in data sets that are transmitted to any Sponsor location.

Patient medical information obtained by this study is confidential and may be disclosed to third parties only as permitted by the Informed Consent Form (or separate authorization for use and disclosure of personal health information) signed by the patient, unless permitted or required by law.

Medical information may be given to a patient's personal physician or other appropriate medical personnel responsible for the patient's welfare, for treatment purposes.

Given the complexity and exploratory nature of the analyses, data derived from exploratory biomarker specimens will generally not be provided to study investigators or patients unless required by law. The aggregate results of any conducted research will

be available in accordance with the effective Sponsor policy on study data publication (see Section 9.5).

Data generated by this study must be available for inspection upon request by representatives of national and local health authorities, Sponsor monitors, representatives, and collaborators, and the IRB/EC for each study site, as appropriate.

8.5 FINANCIAL DISCLOSURE

Investigators will provide the Sponsor with sufficient, accurate financial information in accordance with local regulations to allow the Sponsor to submit complete and accurate financial certification or disclosure statements to the appropriate health authorities. Investigators are responsible for providing information on financial interests during the course of the study and for 1 year after completion of the study (i.e., last patient, last visit).

9. STUDY DOCUMENTATION, MONITORING, AND ADMINISTRATION

9.1 STUDY DOCUMENTATION

The investigator must maintain adequate and accurate records to enable the conduct of the study to be fully documented, including, but not limited to, the protocol, protocol amendments, Informed Consent Forms, and documentation of IRB/EC and governmental approval. In addition, at the end of the study, the investigator will receive the patient data, including an audit trail containing a complete record of all changes to data.

9.2 PROTOCOL DEVIATIONS

The investigator should document and explain any protocol deviations. The investigator should promptly report any deviations that might have an impact on patient safety and data integrity to the Sponsor and to the IRB/EC in accordance with established IRB/EC policies and procedures. The Sponsor will review all protocol deviations and assess whether any represent a serious breach of Good Clinical Practice guidelines and require reporting to health authorities. As per the Sponsor's standard operating procedures, prospective requests to deviate from the protocol, including requests to waive protocol eligibility criteria, are not allowed.

9.3 SITE INSPECTIONS

Site visits will be conducted by the Sponsor or an authorized representative for inspection of study data, subjects' medical records, and eCRFs. The investigator will permit national and local health authorities; Sponsor monitors, representatives, and collaborators; and the IRBs/ECs to inspect facilities and records relevant to this study.

9.4 ADMINISTRATIVE STRUCTURE

This trial is sponsored by Genentech. This pilot study will be conducted at study sites experienced in conducting clinical trials in CSU. Data will be recorded via an EDC system from Medidata Solutions (New York, NY) using eCRFs (see Section 7.2). The contract research organization will be responsible for submission to IRB/ECs for approval of the study protocol, patient recruitment, data collection, and reporting. An IxRS will be used to assign patients to treatment groups and to manage ongoing investigational product requests and shipments.

9.5 PUBLICATION OF DATA AND PROTECTION OF TRADE SECRETS

Regardless of the outcome of a trial, the Sponsor is dedicated to openly providing information on the trial to healthcare professionals and to the public, both at scientific congresses and in peer-reviewed journals. The Sponsor will comply with all requirements for publication of study results. For more information, refer to the Roche Global Policy on Sharing of Clinical Trials Data at the following Web site:

www.roche.com/roche_global_policy_on_sharing_of_clinical_study_information.pdf

The results of this study may be published or presented at scientific congresses. For all clinical trials in patients involving an IMP for which a marketing authorization application has been filed or approved in any country, the Sponsor aims to submit a journal manuscript reporting primary clinical trial results within 6 months after the availability of the respective Clinical Study Report. In addition, for all clinical trials in patients involving an IMP for which a marketing authorization application has been filed or approved in any country, the Sponsor aims to publish results from analyses of additional endpoints and exploratory data that are clinically meaningful and statistically sound.

The investigator must agree to submit all manuscripts or abstracts to the Sponsor prior to submission for publication or presentation. This allows the Sponsor to protect proprietary information and to provide comments based on information from other studies that may not yet be available to the investigator.

In accordance with standard editorial and ethical practice, the Sponsor will generally support publication of multicenter trials only in their entirety and not as individual center data. In this case, a coordinating investigator will be designated by mutual agreement.

Authorship will be determined by mutual agreement and in line with International Committee of Medical Journal Editors authorship requirements. Any formal publication of the study in which contribution of Sponsor personnel exceeded that of conventional monitoring will be considered as a joint publication by the investigator and the appropriate Sponsor personnel.

Any inventions and resulting patents, improvements, and/or know-how originating from the use of data from this study will become and remain the exclusive and unburdened property of the Sponsor, except where agreed otherwise.

9.6 PROTOCOL AMENDMENTS

Any protocol amendments will be prepared by the Sponsor. Protocol amendments will be submitted to the IRB/EC and to regulatory authorities in accordance with local regulatory requirements.

Approval must be obtained from the IRB/EC and regulatory authorities (as locally required) before implementation of any changes, except for changes necessary to eliminate an immediate hazard to patients or changes that involve logistical or administrative aspects only (e.g., change in Medical Monitor or contact information).

10. REFERENCES

Altrichter S, Peter HJ, Pisarevskaja D, et al. IgE mediated autoallergy against thyroid peroxidase–a novel pathomechanism of chronic spontaneous urticaria? PLoS ONE 2011;6:e14794.

Broides A, Yang W, Conley ME. Genotype/phenotype correlations in X-linked agammaglobulinemia. Clin Immunol 2006;118:195–200.

Bruton OC. Agammaglobulinemia. Pediatrics 1952;9:722–8.

Bugatti S, Vitolo B, Caporali R, et al. B cells in rheumatoid arthritis: from pathogenic players to disease biomarkers. Biomed Res Int 2014;2014:681678. doi: 10.1155/2014/681678

Conley ME, Broides A, Hernandez-Trujillo V, et al. Genetic anaylis of patients with defects in early B-cell development. Immunol Rev 2005;203:216–34.

Di Paolo JA, Huang T, Balazs M, et al. Specific BTK Inhibition suppresses B cell and myeloid cell-mediated arthritis. Nat Chem Biol 2011;7:41–50.

[FDA] U.S. Food and Drug Administration. Pharmacology review of Incivek (telaprevir). 2011. Available from: http://www.accessdata.fda.gov/drugsatfda_docs/nda/2011/201917Orig1s000TOC.cfm

Ferrer M. Immunological events in chronic spontaneous urticaria. Clin Transl Allergy 2015;5:360. doi: 10.1186/s13601-015-0074-7

Fiebiger E, Maurer D, Holub H, et al. Serum IgG autoantibodies directed against the α chain of FcεRI: a selective marker and pathogenetic factor for a distinct subset of chronic urticaria patients? J Clin Invest 1995;96:2606–12. doi: 10.1172/JCI118325

Greaves M. Chronic urticaria. J Allergy Clin Immunol 2000;105:664–72.

Greaves M. Chronic idiopathic urticaria. Curr Opin Allergy Clin Immunol 2003;3:363–8.

Hata D, Kawakami Y, Inagaki N, et al. Involvement of Bruton's tyrosine kinase in FcεRI-dependent mast cell degranulation and cytokine production. J Exp Med. 1998 Apr 20;187:1235–47.

Inman WH, Vessey MP, Westerholm B, et al. Thromboembolic disease and the steroidal content of oral contraceptives. A report to the Committee on Safety of Drugs. Br Med J 1970;2:203–9.

Iyer AS, Morales JL, Huang W, et al. Absence of Tec family kinases interleukin-2 inducible T cell kinase (Itk) and Bruton’s tyrosine kinase (Btk) severely impairs FcεRI-dependent mast cell responses. J Bio Chem 2011;286:9503–13. doi: 10.1074/jbc.M110.1656131

Kaplan AP. Chronic urticaria and angioedema. N Engl J Med 2002;346:175–9.

Kaveri SV, Maddur MS, Hegde P, et al. Intravenous immunoglobulins in immunodeficiences: more that mere replacement therapy. Clin Exp Immunol 2011;164(Suppl 2):2–5. doi: 10.1111/j.1365-2249.2011.04387.x

Kay AB, Ying S, Ardelean E, et al. Elevations in vascular markers and eosinophils in chronic spontaneous urticarial weals with low-level persistence in uninvolved skin. Br J Dermatol 2014;171:505–11. doi: 10.1111/bjd.12991

Kellick KA, Bottorff M, Toth PP, et al. A clinician's guide to statin drug-drug interactions. J Clin Lipidol 2014 May-Jun;8(3 Suppl):S30-46.

Kern F, Lichtenstein LM. Defective histamine release in chronic urticaria. J Clin Invest 1976;57:1369–77.

Kozel MA, Sabroe RA. Chronic urticaria, aetiology, management and current and future treatment options. Drugs 2004;64:2515–36.

[redacted]

Lederman HM, Winkelstein JA. X-linked agammaglobulinema: an analysis of 96 patients. Medicine (Baltimore) 1985;64:145–56.

Liu L, Di Paolo J, Barbosa J, et al. Antiarthritis effect of a novel Bruton's tyrosine kinase (BTK) inhibitor in rat collagen-induced arthritis and mechanism-based pharmacokinetic/pharmacodynamic modeling: relationships between inhibition of BTK phosphorylation and efficacy. J Pharmacol Exp Ther 2011;338:154–63.

Luquin E, Kaplan AP, Ferrer M. Increased responsiveness of basophils of patients with chronic urticaria to sera but hypo-responsiveness to other stimuli. Clin Exp Allergy 2005;35:456–60. doi: 10.1111/j.1365-2222.2005.02212.x

Mathias SD, Crosby RD, Zazzali JL, et al. Evaluating the minimally important difference of the urticaria activity score and other measures of disease activity in patients with chronic idiopathic urticaria. Ann Allergy Asthma Immunol 2012;108:20–4. doi: 10.1016/j.anai.2011.09.008

Maurer M, Weller K, Bindslev-Jensen C, et al. Unmet clinical needs in chronic spontaneous urticaria. A GA2LEN task force report. Allergy 2011;66:317–30.

McGirt LY, Vasagar K, Gober LM, et al. Successful treatment of recalcitrant chronic idiopathic urticaria with sulfasalazine. Arch Dermatol 2006;142:1337–42.

Medicinal Products Act in the version published on 12 December 2005 (Federal Law Gazette [BGBl.]) Part I p. 3394, last amended by Article 2a of the Law of 27 March 2014 (Federal Law Gazette I p. 261).

Misbah SA, Spickett GP, Ryba PC, et al. Chronic enteroviral meningoencephalitis in agammaglobulinemia: case report and literature review. J Clin Immunol 1992;12:266–70.

Niiro H, Clark EA. Regulation of B-cell fate by antigen-receptor signals. Nat Rev Immunol 2002;2:945–56.

O'Kelly M, Ratitch B. Clinical trials with missing data: a guide for practitioners. Chichester, UK: John Wiley & Sons, 2014.

Powell RJ, Leech SC, Till S, et al. BSACI guideline for the management of chronic urticaria and angioedema. Clin Exp Allergy 2015;45:547–65. doi: 10.1111/cea.12494

Puri K, Di Paolo J, Gold M. B-cell receptor signaling inhibitors for treatment of autoimmune inflammatory diseases and B-cell malignancies. Int Rev Immunol 2013;32:397–427.

Reth M, Nielsen P. Signaling circuits in early B-cell development. Adv Immunol 2014;122:129–75. doi: 10.1016/B978-0-12-800267-4.00004-3

Saini SS. Chronic spontaneuos urticaria: etiology and pathogensis. Immunol Allergy Clin North Am 2014;34:33–52. doi: 10.1016/j.iac.2013.09.012

Sochorová K, Horvath R, Rozkova D, et al. Impaired toll-like receptor 8-mediated IL-6 and TNF-α production in antigen-presenting cells from patients with X-linked agammaglobulinemia. Blood 2007;109:2553–6.

Snyder PW, Kazacos EA, Scott-Moncrieff JC, et al. Pathologic features of naturally occurring juvenile polyarteritis in beagle dogs. Vet Pathol 1995;32:337–45.

Tilles SA. Approach to therapy in chronic urticaria: when Benadryl is not enough. Allergy Asthma Proc 2005;26:9–12.

Tong LJ, Balakrishnan G, Kochan J. et al. Assessment of autoimmunity in patients with chronic urticaria. J Allergy Clin Immunol 1997;99:461–5.

Tsukada S, Saffran DC, Rawlings DJ, et al. Deficient expression of a B cell cytoplasmic tyrosine kinase in human X-linked agammaglobulinemia. Cell 1993;72:279–90. doi:10.1016/0092-8674(93)90667-F

van den Bruele T, Mourad-Baars PE, Claas EC, et al. Campylobacter jejuni bacteremia and Helicobacter pylori in a patient with X-linked agammaglobulinemia. Eur J Clin Microbiol Infect Dis 2010;29:1315–9.

Vetrie D, Vořechovský I, Sideras P, et al. The gene involved in X-linked agammaglobulinaemia is a member of the src family of protein-tyrosine kinases. Nature 1993;361:226–33. doi:10.1038/361226a0

Wang B, Sanchez RI, Franklin RB, et al. The involvement of CYP3A4 and CYP2C9 in the metabolism of 17 alpha-ethinylestradiol. Drug Metab Dispos 2004;32:1209–12.

Whang JA, Chang BY. Bruton's tyrosine kinase inhibitors for the treatment of rheumatoid arthritis. Drug Discov Today 2014;19:1200–4. doi: 10.1016/j.drudis.2014.03.028

Winkelstein JA, Marino MC, Lederman HM, et al. X-linked agammaglobulinema: report on a United States registry of 201 patients. Medicine (Baltimore) 2006;85:193–202.

Ying S, Kikuchi Y, Meng Q, et al. TH1/TH2 cytokines and inflammatory cells in skin biopsy specimens from patients with chronic idiopathic urticaria: comparison with the allergen-induced late-phase cutaneous reaction. J Allergy Clin Immunol 2002;109:694–700.

Zhang H, Cui D, Wang B, et al. Pharmacokinetic drug interactions involving 17alpha-ethinylestradiol: a new look at an old drug. Clin Pharmacokinet 2007;46:133–57.

Ziegner UH, Kobayashi RH, Cunningham-Rundles C, et al. Progressive neurodegeneration in patients with primary immunodeficiency disease on IVIG treatment. Clin Immunol 2002;102:19–24.

Zweiman B, Valenzano M, Atkins PC. Modulation of serum histamine releasing activity in chronic idiopathic urticaria. Immunopharmacology 1998;39:225–34.

Appendix 1
Schedule of Activities (Cohorts 1 and 2)

	Screening [a]		Treatment Period						Safety Follow Up [b]	Early Term.	Unscheduled Visit
Week			0	1	2	4	6 [c]	8	12		
Day (± days)	−14 (−4/+2)	−7	1 [d]	8 [d] (±1)	15 (±1)	29 [d] (±2)	43 (±2)	57 [d, e] (±2)	85 [d] (±2)		
Informed consent	x										
Demographic data	x										
General medical history and baseline conditions	x										
Inclusion/exclusion criteria	x	x	x								
Randomization			x								
Train patient to complete Urticaria Patient Daily eDiary (including UAS) [f]	x	x									
Distribute eDiary to patient [g]	x										
Urticaria Patient Daily eDiary g		x	x	x	x	x	x	x	x	x	
[redacted]			x			x		x	x		
Concomitant medications [i]	x	x	x	x	x	x	x	x	x	x	x
Adverse events			x	x	x	x	x	x	x	x	x
Vital signs [j]	x	x	x	x	x	x		x	x	x	x [k]
Height	x										
Weight	x		x			x		x	x	x	x [k]
Complete physical examination [l]	x								x		
Limited physical examination [m]		x	x	x	x	x		x		x	x [k]
ECG [n]	x		x	x					x	x	x [k]
Hepatitis Screening [o]	x										
QFT (PPD if QFT not available)	x										

Appendix 1
Schedule of Activities (Cohorts 1 and 2) (cont.)

			Treatment Period						Safety Follow Up [b]		
Week	Screening [a]		0	1	2	4	6 [c]	8	12		
Day (± days)	−14 (−4/+2)	−7	1 [d]	8 [d] (±1)	15 (±1)	29 [d] (±2)	43 (±2)	57 [d, e] (±2)	85 [d] (±2)	Early Term.	Unscheduled Visit
Chest X-ray [p]	x										
Fenebrutinib/placebo administration in clinic [q, r]			x	x	x	x					
Drug Dispensing			x			x					
Hematology [s]	x	x	x	x	x	x		x	x	x	x [k]
Chemistry [t]	x	x	x	x	x	x		x	x	x	x [k]
Fasting Lipid Panel			x			x		x	x		
Coagulation studies [u]	x									x	x [k]
Pregnancy test [v]	x		x	x	x	x		x	x	x	
Urinalysis [w]	x		x					x	x	x	
[redacted]	x								x	x	
[redacted]	x								x	x	
FricTest (for patients with dermographism only)	x		x					x	x	x	
Plasma PK assessment [y]			x	x				x		x	x [k]
[redacted]			x								
[redacted]	x		x					x	x	x	
[redacted]	x		x					x	x	x	
[redacted]			x					x			
Phone call [bb]							x				

Appendix 1
Schedule of Activities (Cohorts 1 and 2) (cont.)

BID = twice a day; eCRF = electronic case report form; eDiary = electronic diary (patient reported outcomes); HBcAb = hepatitis B core antibody; HBsAb = hepatitis B surface antibody; HBsAg = hepatitis B surface antigen; HCV Ab = hepatitis C antibody; PD = pharmacodynamic; PK = pharmacokinetic; PPD = purified protein derivative; PPI = proton pump inhibitor; QFT = QuantiFERON-TB Gold®; QTcF = QT interval corrected using Fridericia's formula; TB = tuberculosis; [redacted] Term. = Termination; UAS = Urticaria Activity Score; UAS7 = Urticaria Activity Score over 7 days; [redacted]

[a] Laboratory tests prior to randomization and dosing may be performed locally on Day 0, if central laboratory tests are not available due to sampling handling problems, breakage, or lab error.

[b] Safety follow-up visit 4 weeks after the last dose of study drug if the patient discontinues the study and/or study treatment (per Sections 4.6.1 and 4.6.2) prior to Day 85 visit.

[c] Phone call instead of a clinic visit.

[d] Morning clinic visit is required for visits on Days 1, 8, 29, 57, and 85; for other study visits, morning visits are recommended. For mandatory morning visits, the patient should be fasting (overnight, > 8 hours) prior to the first PK blood draw and/or fasting lipid panel.

[e] Day 57 visit is the last day of the study treatment period; however, no study drug will be taken at the Day 57 visit. The last dose of blinded study drug will be the p.m. dose on Day 56 or the day before the Day 57 visit if it does not occur on Day 57.

[f] Patients should be trained to use the eDiary at Day –14. At Day –7, staff should query patients for any questions they may have concerning the use of the eDiary and ensure patients understand correct usage before randomization.

[g] Patient is to complete the eDiary twice daily, approximately every 12 hours (a.m./p.m.), every day for the duration of the study. The eDiary includes the UAS7 (itch score, number of hives) and other patient reported outcomes.

[redacted]

[i] Includes any medication (e.g., prescription drugs, over-the-counter drugs, vaccines, herbal or homeopathic remedies, vitamins, and nutritional or dietary supplements) used by a patient from 12 weeks prior to initiation of study drug until 4 weeks after the last dose of study drug. In addition, at each clinic visit, any use of PPIs, H2 receptor antagonists, and/or other antacids (e.g., Pepto-Bismol®, Rolaids®) should be recorded as concomitant medications, including the date, dose, and time of last administration.

[j] Includes respiratory rate, pulse rate, temperature, and systolic and diastolic blood pressures while the patient is in a seated position for at least 5 minutes.

[k] This procedure is optional per the investigator's discretion.

[l] A complete physical examination should include an evaluation of the head, eyes, ears, nose, and throat and the cardiovascular, dermatological, musculoskeletal, respiratory, gastrointestinal, and neurological systems. Patients should be screened for dermographism. Any abnormality identified at baseline should be recorded on the General Medical History and Baseline Condition eCRF.

[m] Perform a limited, symptom-directed examination at specified timepoints or as clinically indicated. Record new or worsened clinically significant abnormalities on the Adverse Event eCRF.

[n] Interpretable digital ECG recording (e.g., without artifacts) will be obtained. The ECG intervals (e.g., PR, QRS, QT, QTcF, and RR) and heart rate from the ECGs will be entered into the eCRF; ECGs for each patient should be obtained from the same machine whenever possible. ECGs can be performed without specific restrictions (e.g., can be any time of day, before or after dosing, fasting or fed) but are to be obtained prior to other procedures scheduled at that same time (e.g., vital sign measurements, blood draws). ECGs must be performed after the patient has been resting in a supine position for at least 10 minutes prior to beginning the ECG recording. Environmental distractions (e.g., television, radio, conversation) should be avoided during the pre-ECG resting period and during ECG recording.

[o] HBsAg, HBsAb, HBcAb, and HCV Ab.

[p] Performed only if required by local guidelines to rule out active TB infection.

[q] On mandatory morning clinic visit days during the treatment period (Days 1, 8, 29, and 57), patients should be instructed that the morning dose of study drug will be taken in the clinic. On other clinic visit days, if the visit occurs in the morning, the patient should be instructed that the morning dose of study drug will be taken in the clinic. The morning dose should be taken after all pre-dose assessments are complete (i.e., ECG, questionnaires, and PK and biomarker sample collection).

[e] Patients will take fenebrutinib/placebo BID approximately every 12 hours starting on Day 1 and ending on Day 56 (pm) or the day before (pm) the Day 57 visit if it does not occur on Day 57. One dose (a total of 4 tablets) of fenebrutinib/placebo should be taken with water by mouth BID (a total of 8 tablets each day). The dates and times of the most recent prior meal, last dose of oral study drug (prior to clinic visit), and timing of study drug administration in clinic should be recorded at each clinic visit.

[s] Includes WBC count, RBC count, hemoglobin, hematocrit, platelet count, and WBC differential (i.e., neutrophils, eosinophils, basophils, monocytes, lymphocytes, and other cells if present).

[t] Includes sodium, potassium, chloride, bicarbonate, glucose, BUN or urea, creatinine, total protein, albumin, phosphorus, calcium, total and direct bilirubin, alkaline phosphatase, ALT, AST, uric acid, LDH, CRP, and lipase.

[u] Includes PT, PTT, INR, and fibrinogen.

[v] All women of childbearing potential, including those who have had a tubal ligation, will have a serum pregnancy test at screening. Urine pregnancy tests will be performed locally at specified subsequent visits. If a urine pregnancy test result is positive, it must be confirmed by a serum pregnancy test (performed locally).

[w] Includes dipstick, including pH, specific gravity, glucose, protein, ketones, blood, and microscopic examination (e.g., sediment, RBCs, WBCs, casts, crystals, epithelial cells, bacteria if present).

[redacted]

[y] Collect PK samples prior to drug administration, and the patient should be fasting overnight for $>$8 hours.

[redacted]

[bb] Site staff to remind patients to take the study drug and to complete their eDiaries.

Appendix 2
Childbearing Potential, Pregnancy Testing, and Contraception

For Women

All women of childbearing potential (including those who have had a tubal ligation) will have a serum pregnancy test at screening and a urine pregnancy test on Study Day 1 prior to administration of study drug and monthly at appropriate clinic visits. If a urine pregnancy test result is positive, study drug will not be administered until pregnancy is ruled out. The result must be confirmed by a serum pregnancy test (conducted by the local laboratory). Refer to Section 5.4.3 of the protocol for management of a patient with a confirmed pregnancy.

All female patients are considered to be of childbearing potential unless they meet one of the following criteria:

- The patient has been postmenopausal (non−therapy-induced amenorrhea) for at least 12 continuous months with no other identified cause.
- The patient had a surgical bilateral oophorectomy (with or without hysterectomy) more than 6 weeks prior to enrollment.
- The patient had a hysterectomy.

Female patients of reproductive or childbearing potential who are unwilling to use a method of contraception that results in a failure rate of < 1 % per year or remain abstinent (refrain from heterosexual intercourse), and refrain from donating eggs during the treatment period and for at least 4 weeks after the last dose of study drug will be excluded from study participation.

Abstinence is acceptable only if it is in line with the preferred and usual lifestyle of the patient. Periodic abstinence (e.g., calendar, ovulation, symptothermal, or post-ovulation methods) and withdrawal are not acceptable methods of contraception.

Examples of contraceptive methods with a failure rate of < 1% per year include the following:

- Sterilization, bilateral surgical tubal ligation
- Intrauterine device
- Combined oral contraceptive pill [1]
- Contraceptive transdermal patch (estrogen and progestin containing)[1]
- Hormonal vaginal device
- Progestogen-only hormonal contraception associated with inhibition of ovulation
- Implants for contraception
- Injections for contraception (with prolonged release)

- Sole sexual partner consisting of surgically sterilized male partner with appropriate postsurgical verification of the absence of spermatozoa in the ejaculate. Patients may provide verbal confirmation that the partner completed appropriate follow-up after vasectomy. Sites are not required to obtain partner medical records.

[1] Women using estrogen-containing hormonal contraceptives as a method of contraception must also use a barrier such as a male condom in conjunction with the hormonal contraceptives.

- **For Men**:
- All men must agree to remain abstinent (refrain from heterosexual intercourse) or use contraceptive measures, and agreement to refrain from donating sperm, as defined below:
- With female partners of childbearing potential or pregnant female partners, men must remain abstinent or use a condom during the treatment period and for at least 4 weeks after the last dose of fenebrutinib to avoid exposing the embryo. Men must refrain from donating sperm during this same period.

For Men and Women

The reliability of sexual abstinence should be evaluated in relation to the duration of the clinical trial and the preferred and usual lifestyle of the patient. Periodic abstinence (e.g., calendar, ovulation, symptothermal, and post-ovulation methods) and withdrawal are not acceptable methods of contraception.

Appendix 3
Urticaria Patient Daily eDiary

<u>General Instructions</u>

Please answer each question to the best of your ability.

There are no right or wrong answers.

For each question, please choose the response that describes your experience.

Please pay close attention to the timeframe of interest. Some questions ask about the **<u>past 12 hours</u>**, while others ask about the **<u>past 24 hours</u>**.

<u>Instructions for Counting the Number of Hives and Measuring the Size of the Largest Hive</u>

Count each hive separately even if you have more than one hive grouped together with other hives.

Please use the ruler that you have been given to measure the size of your largest hive. If you need help, please have someone else take this measurement for you. **Please do not measure a group of hives as one hive.**

Today's Date

		-				-				

Day **Month** **Year**

Please complete this Section <u>every morning</u> throughout the duration of the study. (Please circle only one response.)

1. Thinking about the **<u>past 12 hours</u>**, please record the severity of itch and the number of hives you may have had associated with your skin condition. **<u>Please count each hive separately</u>** even if you have more than one hive grouped together with other hives.

Itch (severity)	Hives (number)
0 = none<br>1 = mild<br>2 = moderate<br>3 = severe	0 = none<br>1 = between 1 and 6 hives<br>2 = between 7 and 12 hives<br>3 = greater than 12 hives

This next question asks you to estimate the size of your largest hive in centimeters (cm). Please use the ruler that you have been provided with to make this measurement. If your largest hive is located on your back or in a place that is hard to reach, please have someone else take this measurement for you. When measuring the largest hive size, **<u>please do not measure a group of hives as one hive.</u>**

Largest Hive (size)
0 = none<br>1 = less than 1.25 centimeter (cm)<br>2 = between 1.25 centimeter (cm) and 2.5 centimeters (cm)<br>3 = greater than 2.5 centimeters (cm)

Appendix 3
Urticaria Patient Daily eDiary (cont.)

Today's Date

		-				-				

Day **Month** **Year**

Please complete this Section <u>every evening</u> throughout the duration of the study. (Please circle only one response.)

2. Thinking about the **<u>past 12 hours</u>**, please record the severity of itch and the number of hives you may have had associated with your skin condition. **<u>Please count each hive separately</u>** even if you have more one than one hive grouped together with other hives.

Itch (severity)	Hives (number)
0 = none<br>1 = mild<br>2 = moderate<br>3 = severe	0 = none<br>1 = between 1 and 6 hives<br>2 = between 7 and 12 hives<br>3 = greater than 12 hives

This next question asks you to estimate the size of your largest hive in centimeters (cm). Please use the ruler that you have been provided with to make this measurement. If your largest hive is located on your back or in a place that is hard to reach, please have someone else take this measurement for you. When measuring the largest hive size, **<u>please do not measure a group of hives as one hive.</u>**

Largest Hive (size)
0 = none<br>1 = less than 1.25 centimeter (cm)<br>2 = between 1.25 centimeter (cm) and 2.5 centimeters (cm)<br>3 = greater than 2.5 centimeters (cm)

Today's Date

		–				–				

Day **Month** **Year**

Please complete this Section once each day throughout the duration of the study (preferably at the same time each day).

(Please circle only one response.)

3. Please rate how much your hives or itch interfered with your sleep during the **past 24 hours**.

   0 No interference

   1 Mild, little interference with sleep

   2 Moderate, awoke occasionally, some interference with sleep

   3 Substantial, woke up often, severe interference with sleep

4. Please rate how much your hives or itch interfered with your daily activities during **the past 24 hours**. This could include work, school, sports, hobbies, and activities with friends and family.

   0 No interference

   1 Mild, little interference with daily activities

   2 Moderate, some interference with daily activities

   3 Substantial, severe interference with daily activities

These next questions are about your symptoms and how you managed them during the past 24 hours.

5. During the **<u>past 24 hours</u>**, did you use loratadine or cetirizine in order to control symptoms of your skin condition such as itch or hives?

   0 = No

   1 = Yes

6a. During the **<u>past 24 hours</u>**, did you have any rapid swelling on your face, (especially your eyelids or lips), inside your mouth (including your throat or tongue), or elsewhere on your body? This rapid swelling, also called angioedema, is at a deeper level <u>under</u> your skin than hives.

   0 = No **(GO TO Question 7)**   1 = Yes

6b. If Yes, how did you treat this rapid swelling? **(Circle all that apply.)**

   0 Did nothing (GO TO Question 7)

   1 Took some prescription or non-prescription medication

   2 Called my doctor, nurse or nurse practitioner

   3 Went to see my doctor, nurse, or nurse practitioner

   4 Went to the emergency room at the hospital

   5 Was hospitalized

7. During the **<u>past 24 hours</u>**, did you or someone else call your doctor, nurse or nurse practitioner because of your skin condition?

   0 = No   1 = Yes

Appendix 4
Urticaria [redacted]

[redacted]

[redacted]

[redacted]

[redacted]

[redacted]

[redacted] [redacted] [redacted] [redacted] [redacted]

[redacted]

[redacted] [redacted] [redacted] [redacted] [redacted]

[redacted]

[redacted] [redacted] [redacted] [redacted] [redacted]

[redacted]

[redacted] [redacted] [redacted] [redacted] [redacted]

Appendix 5
Concomitant Medications (Including Foods and Herbal Products)

Class	Expected Interaction	Recommendation	Examples of Drugs in this Class [a]
Antacids	Decreased fenebrutinib absorption due to increased gastric pH	Take fenebrutinib 2 hours before or 2 hours after antacid	• Pepto-Bismol, Rolaids
Moderate or strong CYP3A inhibitors	Increased fenebrutinib plasma concentrations due to inhibition of metabolism	Avoid for 7 days or 5 half-lives (whichever is longer) prior to first dose of study drug and during the treatment period	• Antimicrobials (clarithromycin, erythromycin, itraconazole, ketoconazole, telithromycin, troleandomycin, voriconazole, posaconazole)<br>• Antidepressants (nefazodone)<br>• Antihypertensive/cardiac (verapamil, diltiazem)<br>• Other (grapefruit juice, Seville orange juice, pomegranate, star fruit)
CYP3A inducers	Decreased fenebrutinib plasma concentrations due to increased metabolism	Avoid for 7 days or 5 half-lives (whichever is longer) prior to first dose of study drug and during the treatment period	• Antimicrobials (rifampin, rifapentine, rifabutin)<br>• Antidepressants (St. John's wort, hyperforin)<br>• Antiepileptics (carbamazepine, phenytoin, phenobarbital, hyperforin)<br>• Diabetes (pioglitazone, troglitazone)<br>• Other (modafinil, bosentan)

Appendix 5
Concomitant Medications (Including Foods and Herbal Products) (cont.)

Class	Expected Interaction	Recommendation	Examples of Drugs in this Class [a]
Sensitive and narrow therapeutic window CYP3A substrates	Potential for increased plasma concentrations of CYP3A substrates due to inhibition of metabolism by fenebrutinib	Use with caution and monitor for adverse events related to CYP3A substrates as directed by product labeling; consult with the Medical Monitor as needed	• Antiemetic/prokinetic (aprepitant, cisapride)<br>• Antihistamine (astemizole, terfenadine)<br>• Antihypertensive/cardiac (dronedarone, eplerenone, felodipine, nisoldipine, quinidine, ticagrelor, vardenafil)<br>• Benzodiazepines (alprazolam, diazepam, midazolam)<br>• Lipid-lowering (simvastatin [recommended maximum dose: 10 mg/day], lovastatin [recommended maximum dose: 20 mg/day])<br>• Migraine (eletriptan, ergotamine)<br>• Steroids (budesonide, fluticasone)<br>• Other (alfentanil, buspirone, conivaptan, darifenacin, dasatinib, dihydroergotamine, fentanyl, lurasidone, pimozide, quetiapine, sildenafil, tolvaptan, triazolam)
BCRP substrates with a narrow therapeutic index	Potential for increased plasma concentrations of BCRP substrates due to inhibition of transport by fenebrutinib	Use with caution and monitor for adverse events related to BCRP substrates as directed by product labeling; consult with the Medical Monitor as needed	• Antihypertensive (prazosin)<br>• Anti-inflammatory (sulfasalazine)<br>• Lipid-lowering (rosuvastatin [recommended maximum dose: 10 mg/day], atorvastatin [recommended maximum dose: 20 mg/day]<br>• Muscle relaxants (dantrolene)<br>• Steroids (estrone-3-sulfate)

[a] The following list is not comprehensive. Please refer to the following websites for additional information and consult the Medical Monitor if necessary:

U.S. FDA Table of Substrates, Inhibitors, and Inducers (Tables 3-1, 3-2, 3-3, and 5-1) (http://www.fda.gov/Drugs/DevelopmentApprovalProcess/DevelopmentResources/DrugInteractionsLabeling/ucm093664.htm)

Indiana University Department of Medicine P450 Interaction Table (http://medicine.iupui.edu/clinpharm/ddis/clinical-table)

PROTOCOL

**TITLE:**	**A PHASE II, MULTICENTER, RANDOMIZED, DOUBLE-BLIND, PLACEBO-CONTROLLED PILOT AND DOSE-RANGING STUDY OF GDC-0853 IN PATIENTS WITH REFRACTORY CHRONIC SPONTANEOUS URTICARIA (CSU)**
**PROTOCOL NUMBER:**	GS39684
**VERSION NUMBER:**	5 (Germany)
**EUDRACT NUMBER:**	2016-004624-35
**TEST PRODUCT:**	Fenebrutinib (GDC-0853, RO7010939)
**MEDICAL MONITOR:**	[redacted]
**SPONSOR:**	Genentech, Inc.
**DATE FINAL:**	Version 1: 13 December 2016
**DATES AMENDED:**	Version 2 (Germany Only): 11 April 2017<br>Version 3: 19 December 2017<br>Version 4: 9 August 2018<br>Version 5 (Germany): See electronic date stamp below.

PROTOCOL AMENDMENT APPROVAL

Approver's Name	Title	Date and Time (UTC)
[redacted]	Company Signatory	01-Feb-2019 22:02:39

CONFIDENTIAL

The information contained in this document, especially any unpublished data, is the property of Genentech, Inc. (or under its control) and therefore is provided to you in confidence as an investigator, potential investigator, or consultant, for review by you, your staff, and an applicable Ethics Committee or Institutional Review Board. It is understood that this information will not be disclosed to others without written authorization from Genentech except to the extent necessary to obtain informed consent from persons to whom the drug may be administered.

PROTOCOL AMENDMENT, VERSION 5 (GERMANY): RATIONALE

Protocol GS39684 has been amended to remove the option for patients to enter the open-label extension (OLE) Study GS40868 after completing the 8-week treatment period. Study GS40868 has been withdrawn in Germany so the option for patients to roll over in the OLE study is not available.

The following changes have been made:

- OLE language has been removed throughout the protocol. Figure 1, "Study Schema," has been updated. Section 3.1.2 has been deleted and the appropriate sections have been revised (Sections 3.1, 3.1.2, and 4.3.2.1 and Appendix 1).
- Post-trial access to fenebrutinib has been updated for the situation in which continued access to the study drug is unlikely per the Roche Global Policy on Continued Access to Investigation Medicinal Product and there are no plans for an extension study (Section 4.3.4).
- An administrative error for the Urticaria Patient Daily eDiary has been corrected (Appendix 3).

Additional minor changes have been made to improve clarity and consistency. Substantive new information appears in italics. This amendment represents cumulative changes to the original protocol.

TABLE OF CONTENTS

PROTOCOL ACCEPTANCE FORM 10

PROTOCOL SYNOPSIS 11

1. BACKGROUND 24
   1.1 Background on Chronic Spontaneous Urticaria 24
   1.2 Background on Bruton's Tyrosine Kinase and Fenebrutinib 25
   1.2.1 Bruton's Tyrosine Kinase 25
   1.2.2 Nonclinical Experience with Fenebrutinib 26
   1.2.3 Clinical Experience with Fenebrutinib 27
   1.3 Study Rationale and Benefit-Risk Assessment 29
   1.3.1 Infections 30
   1.3.2 Bleeding 30
   1.3.3 Cytopenias 31
   1.3.4 Hepatotoxicity 31
   1.3.5 Cardiovascular Effects 31
   1.3.6 Malignancy 31

2. OBJECTIVES AND ENDPOINTS 32

3. STUDY DESIGN 33
   3.1 Description of the Study 33
   3.1.1 Internal Monitoring Committee 36
   3.2 End of Study and Length of Study 37
   3.3 Rationale for Study Design 37
   3.3.1 Rationale for Fenebrutinib Dose and Schedule 37
   3.3.2 Rationale for Patient Population 38
   3.3.3 Rationale for Control Group 39
   [redacted]
   3.3.5 Rationale for Pharmacokinetic Sample Collection Schedule 39
   3.3.6 Rationale for Efficacy Endpoints 39

4. MATERIALS AND METHODS 40

4.1 Patients ... 40
4.1.1 Inclusion Criteria ... 40
4.1.2 Exclusion Criteria ... 42
4.2 Method of Treatment Assignment and Blinding ... 45
4.2.1 Randomization and Blinding ... 45
4.2.2 Unblinding ... 46
4.3 Study Treatment ... 46
4.3.1 Formulation, Packaging, and Handling ... 47
4.3.1.1 Fenebrutinib and Placebo ... 47
4.3.1.2 Background Therapy: Standard-of-Care H1 Antihistamines for CSU ... 47
4.3.2 Dosage, Administration, and Compliance of Fenebrutinib and Placebo ... 47
4.3.2.1 Fenebrutinib and Placebo Dose and Administration ... 47
4.3.2.2 Fenebrutinib and Placebo Compliance ... 48
4.3.2.3 Background Therapy: Standard-of-Care H1 Antihistamines for CSU ... 49
4.3.3 Investigational Medicinal Product Accountability ... 49
4.3.4 Post-Trial Access to Fenebrutinib ... 50
4.4 Concomitant Therapy and Additional Restrictions ... 50
4.4.1 Permitted Therapy ... 50
4.4.2 Cautionary Therapy ... 51
4.4.2.1 Acid-Reducing Agents ... 51
4.4.2.2 Statins ... 51
4.4.2.3 CYP3A and BCRP-Mediated Drug Interactions ... 51
4.4.3 Prohibited Therapy ... 53
4.4.3.1 Live or Attenuated Vaccinations ... 53
4.4.4 Prohibited Food ... 53
4.4.5 Additional Restrictions ... 53
4.5 Study Assessments ... 54
4.5.1 Informed Consent Forms and Screening Log ... 54
4.5.2 Eligibility at Screening ... 54
4.5.2.1 Retesting: Laboratory Inclusion/Exclusion ... 54

4.5.2.2 Re-Screening ... 54
4.5.3 Medical History and Demographic Data ... 54
4.5.4 Physical Examinations ... 55
4.5.5 Vital Signs ... 55
4.5.6 FricTest ... 55
4.5.7 Laboratory, Biomarker, and Other Biological Samples ... 55
4.5.8 Electrocardiograms ... 57
4.5.9 Patient-Reported Outcomes ... 58
4.5.9.1 Urticaria Patient Daily eDiary ... 58
4.5.9.2 Urticaria Activity Score ... 59
[redacted]
4.6 Patient, Treatment, Study, and Site Discontinuation ... 60
4.6.1 Patient Discontinuation ... 60
4.6.2 Study Treatment Discontinuation ... 61
4.6.3 Study and Site Discontinuation ... 61

5. ASSESSMENT OF SAFETY ... 62
5.1 Safety Plan ... 62
5.1.1 Safety Plan for Potential Risks Associated with Fenebrutinib ... 62
5.1.1.1 Infections ... 62
5.1.1.2 Vaccinations ... 64
5.1.1.3 Bleeding ... 64
5.1.1.4 Cytopenias ... 65
5.1.1.5 Gastrointestinal Effects ... 66
5.1.1.6 Hepatotoxicity ... 66
5.1.1.7 Cardiovascular Effects ... 67
5.1.1.8 Vascular Inflammation ... 68
5.1.1.9 Malignancy ... 68
5.1.2 Management of Patients Who Experience Specific Adverse Events ... 69
5.1.2.1 Management of Specific Adverse Events ... 69

5.1.2.2 Management of Increases in QT Interval 70
5.2 Safety Parameters and Definitions 71
5.2.1 Adverse Events 71
5.2.2 Serious Adverse Events (Immediately Reportable to the Sponsor) 71
5.2.3 Adverse Events of Special Interest (Immediately Reportable to the Sponsor).. 72
5.3 Methods and Timing for Capturing and Assessing Safety Parameters... 73
5.3.1 Adverse Event Reporting Period 73
5.3.2 Eliciting Adverse Event Information 73
5.3.3 Assessment of Severity of Adverse Events 73
5.3.4 Assessment of Causality of Adverse Events 74
5.3.5 Procedures for Recording Adverse Events............................ 75
5.3.5.1 Diagnosis versus Signs and Symptoms................................. 75
5.3.5.2 Adverse Events That Are Secondary to Other Events.. 75
5.3.5.3 Persistent or Recurrent Adverse Events................................ 75
5.3.5.4 Abnormal Laboratory Values 76
5.3.5.5 Abnormal Vital Sign Values 77
5.3.5.6 Abnormal Liver Function Tests 77
5.3.5.7 Deaths 77
5.3.5.8 Preexisting Medical Conditions... 78
5.3.5.9 Lack of Efficacy or Worsening of Chronic Spontaneous Urticaria 78
5.3.5.10 Hospitalization or Prolonged Hospitalization.......................... 78
5.3.5.11 Adverse Events Associated with an Overdose or Error in Drug Administration 79
5.3.5.12 Patient-Reported Outcome Data 79
5.4 Immediate Reporting Requirements from Investigator to Sponsor.. 79
5.4.1 Emergency Medical Contacts 80
5.4.2 Reporting Requirements for Serious Adverse Events and Adverse Events of Special Interest 80
5.4.2.1 Events That Occur prior to Study Drug Initiation.................... 80

5.4.2.2	Events That Occur after Study Drug Initiation	80
5.4.3	Reporting Requirements for Pregnancies	81
5.4.3.1	Pregnancies in Female Patients	81
5.4.3.2	Pregnancies in Female Partners of Male Patients	81
5.4.3.3	Congenital Anomalies/Birth Defects and Abortions	82
5.5	Follow-Up of Patients after Adverse Events	82
5.5.1	Investigator Follow-Up	82
5.5.2	Sponsor Follow-Up	82
5.6	Adverse Events That Occur after the Adverse Event Reporting Period	82
5.7	Expedited Reporting to Health Authorities, Investigators, Institutional Review Boards, and Ethics Committees	83
6.	STATISTICAL CONSIDERATIONS AND ANALYSIS PLAN	83
6.1	Determination of Sample Size	83
6.1.1	Cohort 1: Pilot Assessment	83
6.1.2	Cohort 2: Dose-Ranging Assessment	84
6.2	Summaries of Conduct of Study	84
6.3	Summaries of Treatment Group Comparability	85
6.4	Efficacy Analyses	85
6.4.1	Primary Efficacy Endpoint	85
6.4.2	Secondary Efficacy Endpoints	86
6.4.3	Exploratory Efficacy Endpoints	86
6.5	Safety Analyses	87
6.6	Pharmacokinetic Analyses	87
[redacted]	[redacted]	
6.8	Interim Analysis	88
6.8.1	Cohort 1: Planned Interim Analysis	88
6.8.2	Cohort 2: Optional Interim Analysis	88
7.	DATA COLLECTION AND MANAGEMENT	88
7.1	Data Quality Assurance	88
7.2	Electronic Case Report Forms	89
7.3	Electronic Patient-Reported Outcome Data	89

7.4 Source Data Documentation ... 89
7.5 Use of Computerized Systems ... 90
7.6 Retention of Records ... 90

8. ETHICAL CONSIDERATIONS ... 91
8.1 Compliance with Laws and Regulations ... 91
8.2 Informed Consent ... 91
8.3 Institutional Review Board or Ethics Committee ... 92
8.4 Confidentiality ... 92
8.5 Financial Disclosure ... 93

9. STUDY DOCUMENTATION, MONITORING, AND ADMINISTRATION ... 93
9.1 Study Documentation ... 93
9.2 Protocol Deviations ... 93
9.3 Site Inspections ... 93
9.4 Administrative Structure ... 94
9.5 Publication of Data and Protection of Trade Secrets ... 94
9.6 Protocol Amendments ... 95

10. REFERENCES ... 96

LIST OF TABLES

Table 1 Objectives and Corresponding Endpoints ... 32
Table 2 Twice Daily Patient Assessment of CSU Disease Activity (UAS Scale) ... 40
Table 3 Fenebrutinib Dosing Regimen by Treatment Arm (Cohort 2) ... 48
[redacted]
Table 5 Guidelines for Management of Patients Who Experience Specific Adverse Events ... 69
Table 6 Adverse Event Severity Grading Scale ... 74
Table 7 Causal Attribution Guidance ... 74

LIST OF FIGURES

Figure 1 Study Schema ... 36

LIST OF APPENDICES

Appendix 1 Schedule of Activities (Cohorts 1 and 2) 100
Appendix 2 Childbearing Potential, Pregnancy Testing, and Contraception.. 105
Appendix 3 Urticaria Patient Daily eDiary 107
[redacted]
Appendix 5 Concomitant Medications (Including Foods and Herbal Products)... 113

PROTOCOL AMENDMENT ACCEPTANCE FORM

**TITLE:**	**A PHASE II, MULTICENTER, RANDOMIZED, DOUBLE-BLIND, PLACEBO-CONTROLLED PILOT AND DOSE-RANGING STUDY OF GDC-0853 IN PATIENTS WITH REFRACTORY CHRONIC SPONTANEOUS URTICARIA (CSU)**
**PROTOCOL NUMBER:**	GS39684
**VERSION NUMBER:**	5 (Germany)
**EUDRACT NUMBER:**	2016-004624-35
**TEST PRODUCT:**	Fenebrutinib (GDC-0853, RO7010939)
**MEDICAL MONITOR:**	[redacted]
**SPONSOR:**	Genentech, Inc.

I agree to conduct the study in accordance with the current protocol.

____

Principal Investigator's Name (print)

____ ____________________

Principal Investigator's Signature Date

Please return a copy of the signed form as instructed by the CRO. Please retain the original for your study files.

PROTOCOL SYNOPSIS

**TITLE:**	**A PHASE II, MULTICENTER, RANDOMIZED, DOUBLE-BLIND, PLACEBO-CONTROLLED PILOT AND DOSE-RANGING STUDY OF GDC-0853 IN PATIENTS WITH REFRACTORY CHRONIC SPONTANEOUS URTICARIA (CSU)**
**PROTOCOL NUMBER:**	GS39684
**VERSION NUMBER:**	5 (Germany)
**EUDRACT NUMBER:**	2016-004624-35
**TEST PRODUCT:**	Fenebrutinib (GDC-0853, RO7010939)
**PHASE:**	Phase II
**INDICATION:**	Refractory Chronic Spontaneous Urticaria (CSU)
**SPONSOR:**	Genentech, Inc.

Objectives and Endpoints

This pilot and dose-ranging study will evaluate the efficacy, safety, and pharmacokinetics of fenebrutinib compared with placebo in patients with chronic spontaneous urticaria (CSU) refractory to anti-histamines (up to 4 times the approved dose per local treatment guidelines). Specific objectives and corresponding endpoints for the study are outlined below.

Objectives and Corresponding Endpoints

Objectives	Corresponding Endpoints
**Efficacy Objective:**	
• To evaluate the efficacy of fenebrutinib compared with placebo in patients with CSU who are refractory to anti-histamines	**Primary Endpoint:**<br>• Change from baseline in the UAS7 at Day 57 (Week 8)<br>**Secondary Endpoints:**<br>• Proportion of patients who are well controlled (UAS7 ≤ 6) at Day 57<br>• Change from baseline in the UAS7 at Day 29 (Week 4)<br>**Exploratory Endpoints:**<br>• Change from baseline in the weekly itch score at Day 29<br>• Change from baseline in the weekly itch score at Day 57<br>• Change from baseline in the weekly hives score at Day 57<br>• Proportion of patients who are well controlled (UAS7 ≤ 6) at Day 29<br>• Proportion of patients who achieve complete response (UAS7 = 0) at Day 29<br>• Proportion of patients who achieve complete response (UAS7 = 0) at Day 57<br>• Proportion of patients achieving MID in UAS7 at Day 57 (reduction from baseline ≥ 11 points)<br>• Proportion of patients achieving MID in the weekly itch score at Day 57 (reduction from baseline ≥ 5 points)

Objectives and Corresponding Endpoints (Continued)

Objectives	Corresponding Endpoints
	Exploratory Endpoints (Continued):<br>• Time to achieving MID in UAS7 (reduction from baseline ≥ 11 points)<br>[redacted]
**Safety Objective:**	
• To evaluate the safety of fenebrutinib compared with placebo	• The nature, frequency, timing, and severity of adverse events<br>• Change from baseline in targeted vital signs, physical examination findings, ECGs, and clinical laboratory results following fenebrutinib administration
**Pharmacokinetic Objective:**	
• To characterize the pharmacokinetics of fenebrutinib in patients	• Plasma concentrations of fenebrutinib at specified timepoints
[redacted]	
[redacted]	[redacted]
[redacted]	[redacted]
[redacted]	[redacted]
[redacted]	
[redacted]	[redacted]

AUC_{0-t} = area under the concentration–time curve from time 0 to time t [redacted] CL/F = apparent clearance; C_{max} = maximum concentration observed; C_{trough} = steady-state concentration at the end of a dosing interval; CSU = chronic spontaneous urticaria; MID = minimally important difference; [redacted] PK = pharmacokinetic; $t_{1/2}$ = half-life; t_{max} = time to maximum concentration; UAS7 = Urticaria Activity Score over 7 days; [redacted]

Study Design

Description of Study

This pilot and dose-ranging study is a multicenter, randomized, double-blind, placebo-controlled, parallel-group study of the efficacy and safety of fenebrutinib as add-on therapy for the treatment of adult patients 18 – 75 years old who have been diagnosed with CSU and who remain symptomatic despite treatment with H1 antihistamines (including doses up to 4 times the approved dose per local treatment guidelines). The study will consist of two cohorts. Cohort 1 will enroll approximately 45 patients across multiple sites. After screening, eligible patients will

be randomly allocated in a 2:1 ratio to receive fenebrutinib 200 mg orally (PO) twice daily (BID) or matching placebo for 8 weeks and will maintain stable doses of standard-of-care H1 antihistamine therapy throughout the study. On the basis of results from an interim analysis of Cohort 1, a dose-ranging cohort, Cohort 2, will be opened and approximately 120 patients will be randomly allocated in a 1:1:1:1 ratio to receive 50 mg PO daily (QD), 150 mg PO QD, 200 mg PO BID of fenebrutinib, or placebo, respectively, for 8 weeks and will maintain stable doses of standard-of-care H1 antihistamine therapy (background therapy) throughout the study.

Both cohorts will consist of 3 distinct study periods over a time-period of 14 weeks as outlined below:

- Screening period: Day −14 to Day −1
- Treatment Period: Day 1 to Day 57 (Week 0 to Week 8)
- Follow-Up Period: Day 57 to Day 85 (Week 8 to Week 12)

Patients in both cohorts will have a screening period of approximately 2 weeks to establish their eligibility for the study and baseline symptom scores. For the duration of the screening period, patients must maintain stable doses of their pre-screening combination therapy with standard-of-care H1 antihistamines (i.e., up to 4 times the approved dose per local treatment guidelines). The screening period will consist of visits at Day −14 and Day −7. Patients must meet all of the following criteria to enter the screening period:

- Documented treatment with a regimen that includes standard-of-care H1 antihistamine for CSU at Day −14 and for at least the 3 consecutive days immediately prior to Day −14
- Willing and able to complete a symptom electronic diary (Urticaria Patient Daily eDiary) twice daily throughout the screening period to establish the patient’s Urticaria Activity Score over 7 days (UAS7) score.

To be eligible for randomization in both cohorts, for the 7 days prior to randomization, patients must meet all of the following:

- Seven consecutive days of entries in the Urticaria Patient Daily eDiary, and
- UAS7 symptom score of ≥16 (range: 0–42)

Only in exceptional circumstances, when information concerning eligibility is outstanding (e.g., delayed laboratory results), will a longer screening period be permitted up to 3 business days. Upon approval from the Medical Monitor, patients may be re-screened or may be retested during the screening period. Circumstances that may permit re-screening or retesting include, but are not limited to, a laboratory test result that does not meet eligibility requirements.

On Day 1, eligible patients in Cohort 1 will be randomly allocated in a 2:1 ratio to receive fenebrutinib 200 mg orally (PO) twice daily (BID) or placebo for 8 weeks. Eligible patients in Cohort 2 will be will be randomly allocated in a 1:1:1:1 ratio to receive 50 mg PO daily (QD), 150 mg PO QD, 200 mg PO BID of fenebrutinib, or placebo, respectively, for 8 weeks. The primary efficacy endpoint will be at Day 57 (Week 8). Throughout the treatment period, patients must maintain stable doses of their pre-randomization H1 antihistamine therapy.

After completion of the 8-week treatment period, patients in both cohorts will enter a 4-week safety follow-up period to allow for further characterization of the pharmacokinetics and pharmacodynamics of fenebrutinib, and collection of additional efficacy and safety data. During safety follow-up period, no study treatment will be given; patients must maintain stable doses of their pre-randomization CSU H1 antihistamine treatment (background therapy). In the safety follow-up period, patients may add up to one additional H1 antihistamine therapy in case of worsened symptoms. The goal of allowing additional H1 antihistamine therapy after the treatment period is to reduce patient dropout for improved safety evaluation.

In addition to their daily background therapy, for the duration of the study all patients will be able to use a single approved dose of loratadine (10 mg maximum) or cetirizine (10 mg maximum) within a 24-hour period as rescue medication if symptoms worsen. If a patient needs rescue therapy and is already on background treatment with cetirizine or loratadine, the patient may receive 10 mg more of the same drug only if the total daily dose remains below 4 times the approved dose. Otherwise, the alternate rescue medication may be used. Patients should record the use of this medication in their eDiary. Patients receiving proton-pump inhibitors

(PPIs) or H2 receptor antagonists (H2RAs) should be stabilized on a regimen beginning at least 2 weeks prior to randomization and continuing throughout the study.

Internal Monitoring Committee

For Cohort 2, periodic safety reviews and any interim analysis will be performed by the Sponsor's internal monitoring committee (IMC) as outlined in the IMC charter. This committee will be unblinded to treatment assignments and will include Sponsor representatives from the following functions: Clinical Science, Drug Safety, Biostatistics, and Statistical Programming and Analysis. The IMC members will not have direct contact with investigational staff or site monitors. The IMC may decide to unblind the study team to enable decision-making and potential interactions with regulatory bodies. The IMC may invite representatives from other functional areas on an ad-hoc basis when additional expertise is required (e.g., Clinical Pharmacology, Research, etc.) or additional Sponsor scientists to participate in data analyses and review.

At any time during the study, the Sponsor may choose to inactivate and suspend enrollment and further dosing for a given treatment arm (in Cohort 2) or reduce the dose due to safety concerns and as guided by the IMC. In Cohort 2, subsequently enrolled patients will be randomly allocated to the remaining active arms.

Number of Patients

Approximately 45 patients, aged 18 to 75 years old who have been diagnosed with refractory CSU and who remain symptomatic despite standard-of-care H1 antihistamine (i.e., up to 4 times the approved dose per local treatment guidelines), will be enrolled in Cohort 1. On the basis of results from an interim analysis of Cohort 1, a dose-ranging cohort, Cohort 2, will be opened and approximately 120 patients will be enrolled.

Target Population

<u>Inclusion Criteria</u>

Patients in Cohort 1 and 2 must meet the following criteria for study entry:

- Willing to give written informed consent, adhere to the visit schedules, comply with the study drug regimen, and meet other study requirements
- Aged 18 – 75 years, inclusive
- Diagnosis of CSU refractory to H1 antihistamines at the time of randomization, as defined by all of the following:
  - The presence of itch and hives for > 6 consecutive weeks at any time prior to enrollment despite current use of H1 antihistamine, consistent with standard of care (i.e., up to 4 times the approved dose per local treatment guidelines) during this time period
  - UAS7 score ≥ 16 during the 7 days prior to randomization (Day 1)
  - Patients must have been on daily stable doses of H1 antihistamine, consistent with standard of care (i.e., up to 4 times the approved dose per local treatment guidelines) treatment for CSU starting at least 3 consecutive days immediately prior to the screening visit through Day 1and must document current use on all visits.
  - CSU diagnosis for ≥ 6 months
- Willing and able to complete an Urticaria Patient Daily eDiary for the duration of the study
- Completion of 7 days of the Urticaria Patient Daily eDiary entries in the 7 days prior to randomization (7 of 7 days must be completed [i.e., must complete an entry every day] with up to 2 non-consecutive entries missed)
- No evidence of active or latent or inadequately treated infection with tuberculosis (TB) as defined by the following:
  - A negative QuantiFERON-TB-Gold® (QFT) performed at the screening visit or within the 3 months prior to screening (for German sites only: QFT is the preferred test)

    If QFT is unavailable, a negative Mantoux purified protein derivative (PPD) skin test as defined by the Centers for Disease Control and Prevention guidelines, may be performed at the screening visit or within the 3 months prior to screening -AND-

Any additional procedures (e.g., chest X-Ray) required per local guidelines to rule out latent or active TB

NOTE: A documented negative screening for TB via the PPD test or a negative QFT within 3 months prior to screening (and if required per local standard of care, a chest X-ray), is sufficient and no further screening with QFT is required.

Patients with a history of Bacille Calmette-Guérin (BCG) vaccination should be screened using the QFT test, only.

- An indeterminate QFT test should be repeated.
- A positive QFT test or two successive indeterminate QFT results should be considered a positive diagnostic TB test.
- An indeterminate QFT test followed by a negative QFT test should be considered a negative diagnostic TB test.

- Only for patients currently receiving PPIs or H2RAs: Treatment must be at a stable dose during the 2 week screening period prior to randomization and with a plan to remain at a stable dose for the duration of the study
- For women of childbearing potential: Agreement to remain abstinent (refrain from heterosexual intercourse) or use contraceptive methods that result in a failure rate of $<1\%$ per year during the treatment period and for at least 4 weeks after the last dose of study drug. Women must refrain from donating eggs during this same period.

  A woman is considered to be of childbearing potential if she is postmenarcheal, has not reached a postmenopausal state (≥ 12 continuous months of amenorrhea with no identified cause other than menopause), and has not undergone surgical sterilization (removal of ovaries and/or uterus).

  Examples of contraceptive methods with a failure rate of $<1\%$ per year include bilateral tubal ligation, male sterilization, established proper use of hormonal contraceptives that inhibit ovulation, hormone-releasing intrauterine devices, and copper intrauterine devices. Women using estrogen-containing hormonal contraceptives as a method of contraception must also use a barrier, such as a male condom, in conjunction with the hormonal contraceptives.

  The reliability of sexual abstinence should be evaluated in relation to the duration of the clinical trial and the preferred and usual lifestyle of the patient. Periodic abstinence (e.g., calendar, ovulation, symptothermal, or post-ovulation methods) and withdrawal are not acceptable methods of contraception.

- For men: agreement to remain abstinent (refrain from heterosexual intercourse) or use contraceptive measures and agreement to refrain from donating sperm, as defined below:

  With female partners of childbearing potential or pregnant female partners, men must remain abstinent or use a condom during the treatment period and for at least 4 weeks after the last dose of study treatment to avoid exposing the embryo. Men must refrain from donating sperm during this same period.

  The reliability of sexual abstinence should be evaluated in relation to the duration of the clinical trial and the preferred and usual lifestyle of the patient. Periodic abstinence (e.g., calendar, ovulation, symptothermal, or post-ovulation methods) and withdrawal are not acceptable methods of contraception.

Exclusion Criteria

Patients in Cohort 1 and 2 who meet any of the following criteria will be excluded from study entry:

- Treatment with omalizumab or other monoclonal antibody therapies used to treat CSU within 4 months prior to screening or primary nonresponse to omalizumab
- Use of a non-biologic investigational drug or participation in an investigational study with a non-biologic drug within 30 days prior to study drug administration on Day 1 (or within 5 half-lives of the investigational product, whichever is greater)

- Use of a biologic investigational therapy or participation in an investigational study involving biologic therapy within 90 days or 5 half-lives, whichever is greater, prior to study drug administration on Day 1
- Previous treatment with fenebrutinib or other Bruton's tyrosine kinase (BTK) inhibitors
- Patients whose urticaria is solely due to physical urticaria
- Other diseases with symptoms of urticaria or angioedema, including urticarial vasculitis, urticaria pigmentosa, erythema multiforme, mastocytosis, hereditary or acquired angioedema, lymphoma, or leukemia
- Atopic dermatitis, bullous pemphigoid, dermatitis herpetiformis, or other skin disease associated with itch such as psoriasis
- Routine (daily or every other day during 5 or more consecutive days) doses of the following medications within 30 days prior to screening: systemic or cutaneous (topical) corticosteroids (prescription or over the counter), hydroxychloroquine, methotrexate, cyclosporine, or cyclophosphamide
- Prior utilization of intravenous (IV) steroids for treatment of laryngeal angioedema
- IV immunoglobulin G (IVIG) or plasmapheresis within 30 days prior to screening
- History of anaphylactic shock without clearly identifiable avoidable antigen (e.g., due to food allergy)
- Hypersensitivity to fenebrutinib or any component of the formulation
- Major surgery, within 8 weeks prior to screening or surgery planned prior to end of study (12 weeks after randomization)
- Require any prohibited concomitant medications
- History of live attenuated vaccine within 6 weeks prior to randomization or requirement to receive these vaccinations at any time during study drug treatment

    Seasonal influenza and H1N1 vaccination is permitted if the inactivated vaccine formulation is administered.
- Evidence of clinically significant cardiac, neurologic, psychiatric, pulmonary, renal, hepatic, endocrine (including uncontrolled diabetes mellitus), metabolic, or gastrointestinal (GI) disease that, in the investigator's opinion, would compromise the safety of the patient, interfere with the interpretation of the study results, or otherwise preclude patient participation.

    Any items that are cause for uncertainty must be reviewed with the Medical Monitor.
- Uncontrolled disease states, such as asthma, psoriasis, or inflammatory bowel disease, where flares are commonly treated with oral or parenteral corticosteroids
- History of vasculitis
- Current liver disease
- Any known active infection (with the exception of fungal nail infections or oral herpes)
- History of recurrent bacterial, viral, mycobacterial or fungal infections (defined as >2 similar episodes requiring anti-microbial treatment within the previous 12 months), with the exception of recurrent oral or genital herpes (herpes simplex virus 1/herpes simplex virus 2) or uncomplicated urinary tract infections in females.
- Any history of opportunistic infections that, in the investigator or Sponsor's judgment, would raise safety concerns regarding the patient's participation in the study
- Any major episode of infection requiring hospitalization or treatment with IV antimicrobials within 8 weeks prior to and during screening or treatment with oral antimicrobials within 2 weeks prior to and during screening

    Antimicrobials include antifungal, antibacterial, and antiviral agents.
- History of or currently active primary or secondary immunodeficiency, including known history of HIV infection

- Evidence of chronic and/or active hepatitis B or C
  - Positive hepatitis B surface antigen (HBsAg) or hepatitis C serology (regardless of treatment status)
  - Positive hepatitis B core antibody (HBcAb)
- History of cancer, including hematologic malignancy and solid tumors, within 10 years before screening
  - Basal or squamous cell carcinoma of the skin that has been excised and is considered cured and in situ carcinoma of the cervix treated with apparent success by curative therapy > 1 year prior to screening are not exclusionary.
- Women who are pregnant, nursing (breastfeeding), or intending to become pregnant during the study or within 4 weeks after completion of the study
- For women of childbearing potential (including those who have had a tubal ligation): positive serum pregnancy test result at screening or on Day 1.
  - A serum pregnancy test is needed on Day 1 only if the urine pregnancy test is positive.
- History of alcohol, drug(e.g., tetrahydrocannabinol [THC], marijuana), or chemical abuse within the 12 months prior to screening as determined by the investigator
- Need for systemic anti-coagulation with warfarin, other oral or injectable anti-coagulants, or anti-platelet agents other than non-steroidal anti-inflammatory drugs (NSAIDs), aspirin, and other salicylates
- History of non-gallstone–related pancreatitis or chronic pancreatitis
- History of hospitalizations or transfusion for a GI bleed
- History of cerebrovascular accident (CVA) within 10 years or any history of hemorrhagic CVA
- History of spontaneous intracranial hemorrhage or history of traumatic intracranial hemorrhage within 10 years
- Known bleeding diathesis
- Screening 12-lead ECG that demonstrates clinically relevant abnormalities that may affect patient safety or interpretation of study results, including
  - QT interval corrected using Fridericia's formula (QTcF) > 440 ms demonstrated by at least two ECGs > 30 minutes apart
- History of ventricular dysrhythmias or risk factors for ventricular dysrhythmias such as long QT syndrome and other genetic risk factors (e.g., Brugada syndrome), structural heart disease (e.g., severe left ventricular systolic dysfunction, severe left ventricular hypertrophy), coronary heart disease (symptomatic, or with ischemia demonstrated by diagnostic testing, prior coronary artery bypass grafting, or coronary lesions > 70% diameter stenosis that have not been or cannot be re-vascularized), clinically significant electrolyte abnormalities (e.g., hypokalemia, hypomagnesemia, hypocalcemia), or family history of sudden unexplained death or cardiac ion channel mutations (e.g., congenital long QT syndrome)
- Current treatment with medications that are well known to prolong the QT interval (see https://crediblemeds.org/index.php/login/dlcheck) at doses that have a clinically meaningful effect on QT, as determined by the investigator; the investigator may contact the Sponsor for confirmation if needed
- Current treatment with astemizole, terfenadine, and/or ebastine
- Any condition possibly affecting oral drug absorption (e.g., gastrectomy, clinically significant diabetic gastroenteropathy, or certain types of bariatric surgery such as gastric bypass)
  - Procedures such as gastric banding, that simply divide the stomach into separate chambers, are not exclusionary.
- Any uncontrolled clinically significant laboratory abnormality that would affect safety, interpretation of study data, or the patient's participation in the study
- Subjects who live in detention on court order or on regulatory action as per local and national law (see §40 subSection 1 sentence 3 no. 4 Arzneimittelgesetz).

The following exclusion criteria are based on screening laboratory tests. Laboratory tests may be repeated once during the screening period unless otherwise indicated:

- Creatinine $>$ 1.5 times the upper limit of normal (ULN; may be repeated if 1.5–2 $\times$ ULN)
- Creatinine clearance $<$ 70 mL/min (may be repeated if 60–69 mL/min) as estimated by the Cockcroft-Gault Equation
- ALT or AST $>$ 1.5 times ULN (may be repeated if 1.5–3 $\times$ ULN)
- Total bilirubin $>$ ULN (may be repeated if 1–3 $\times$ ULN)
- Hemoglobin $<$ 11 g/dL (may be repeated if 10–10.9 g/dL)
- ANC $< 1.5 \times 10^9$/L (may be repeated if 1.2–1.5×10^9/L)
- Platelet count $< 100 \times 10^9$/L (may be repeated if 80–100×10^9/L)
- IgG $<$ 500 mg/dL (should not be repeated)
- Abnormalities in hepatic synthetic function tests (e.g., PT, INR, PTT, albumin) judged by the investigator to be clinically significant

End of Study

The end of this study is defined as the date when all patients have completed the study completion visit or early termination visit or have otherwise been discontinued from the study.

Length of Study

The total duration of this study for each subject is approximately 14 weeks, including screening, treatment, and safety follow-up periods.

The total length of the study, from screening of the first patient to the end of the study, is expected to be approximately 19 months.

Investigational Medicinal Products

The investigational medicinal product for this study is fenebrutinib. For Cohort 1, patients will receive fenebrutinib 200 mg PO BID or placebo for 8 weeks. For Cohort 2, will receive fenebrutinib 50 mg QD, 150 mg QD, 200 mg BID, or placebo for 8 weeks.

Non-Investigational Medicinal Products

Throughout the treatment period, patients must maintain stable doses of their pre-randomization H1 antihistamine therapy. In addition to their daily background therapy, for the duration of the study all patients will be able to use a single approved dose of loratadine (10 mg maximum) or cetirizine (10 mg maximum) within a 24-hour period as rescue medication if symptoms worsen. If a patient needs rescue therapy and is already on background treatment with cetirizine or loratadine, the patient may receive 10 mg more of the same drug only if the total daily dose remains below 4 times the approved dose. Otherwise, the alternate rescue medication may be used.

Statistical Methods

Primary Analysis

The primary efficacy endpoint is the change from baseline in the UAS7 at Day 57 (Week 8).

The Urticaria Activity Score (UAS) is to be recorded twice daily (i.e., morning and evening) using an eDiary that will be provided to each patient. Scores ranging from 0 (none) to 3 (severe) will be entered for each of the two UAS domains consisting of number of wheals (hives) and intensity of pruritus (itch) resulting in a total possible score of 0 to 6. The daily UAS is calculated as the average of the morning and evening scores. When either the morning or evening score is missing, the non-missing UAS for that day (morning or evening) will be used as the daily UAS, and when both the morning and evening UAS are missing, the daily UAS will be deemed missing. The UAS7 is the sum of the daily UAS over the 7 days prior to the time point of interest. The baseline UAS7 will be calculated as the sum of daily UAS values over the week (7 days) prior to Day 1.

When one or more daily UAS values is missing, over the week prior to a timepoint of interest, rules for deriving UAS7 will be as follows:

- If a patient has at least 4 completed daily scores on the UAS (both domains) over the 7 days prior to the time point of interest, the UAS7 will be defined as the average of the available daily scores, multiplied by 7.
- If a patient has fewer than 4 completed daily scores on the UAS over the 7 days prior to the time point of interest, then the UAS7 will be considered missing for that time point.

The primary endpoint will be analyzed using a mixed model for repeated measures model. Additional model covariates will include baseline UAS7 and its interaction with visit. Missing data will be handled by the model under the missing-at-random assumption without need for imputation. As a sensitivity analysis, an analysis of covariance (ANCOVA) model adjusted for country and baseline UAS7 will be fit, and missing Day 57 data will be imputed by last observation carried forward.

Determination of Sample Size

Cohort 1: Pilot Assessment

The purpose of this cohort is to evaluate the efficacy of fenebrutinib compared with placebo in improving the UAS7. Point and interval estimates of the change from baseline of the UAS7 within each treatment group as well as of the difference in change from baseline of the UAS7 between treatment groups will be presented.

The cohort will enroll approximately 45 patients. Patients will be randomized in a 2:1 ratio to receive treatment with either fenebrutinib or placebo. The sample size of approximately 30 patients in the fenebrutinib arm and 15 patients in the placebo arm provides approximately 80% power to detect an 11-point difference in the UAS7 change from baseline at Day 57 between treatment groups under the following assumptions:

- The absolute change from baseline at Day 57 is normally distributed with a standard deviation of 13.
- Two-sided alpha is 0.10.
- Drop out at Day 57 is 10%, leading to a 10% loss of information.

Cohort 2: Dose-Ranging Assessment

The purpose of this cohort is estimation and hypothesis generation regarding the dose-ranging effects of fenebrutinib compared with placebo in improving the UAS7. Point and interval estimates of the change from baseline of the UAS7 within each treatment group as well as of the difference in change from baseline of the UAS7 between treatment groups vs placebo will be presented.

The cohort will enroll approximately 120 patients. Patients will be randomly allocated in a 1:1:1:1 ratio to receive treatment with one of three dose levels of fenebrutinib or placebo. The sample size of approximately 30 in each arm provides approximately 90% power to detect an 11-point difference in the UAS7 change from baseline at Day 57 between treatment groups, under the following assumptions:

- The absolute change from baseline at Day 57 is normally distributed with a standard deviation of 13
- Two-sided alpha is 0.10
- Dropout rate at Day 57 is 10%, leading to a 10% loss of information.

The overall sample size may be adjusted depending on the outcome of a planned interim analysis for Cohort 1, which will include an evaluation of these assumptions.

Interim Analysis

Cohort 1: Planned Interim Analysis

An interim analysis will be performed after approximately 33 patients have completed their 8–week treatment period. The purpose of this analysis is to assess the efficacy of the 200-mg fenebrutinib BID daily arm compared with the placebo, to guide internal decision-making around

issues such as ungating of Cohort 2, adequacy of sample sizes for safety and/or efficacy analyses in Cohort 2, or to inform further development decisions. Summaries of safety and efficacy data by treatment groups will be prepared and reviewed by Sponsor personnel who do not have direct contact with investigational staff, monitors, and patients. Further details of the interim analysis will be specified in the data analysis plan (DAP) prior to the conduct of the interim analysis. Access to treatment assignment information will follow the Sponsor's standard procedures.

Cohort 2: Optional Interim Analysis

Given the hypothesis-generating nature of this study, the Sponsor may choose to conduct up to two interim efficacy analyses. The decision to conduct an optional interim analysis and the timing of the analysis will be documented in the Sponsor's trial master file prior to the conduct of the interim analysis. The interim analysis will be performed and interpreted by members of the IMC. Access to treatment assignment information will follow the Sponsor's standard procedures.

LIST OF ABBREVIATIONS AND DEFINITIONS OF TERMS

Abbreviation	Definition
ANCOVA	analysis of covariance
AUC	area under the concentration-time curve
AUC_{0-24}	area under the concentration-time curve from time 0 to 24 hours
AUC_{0-t}	area under the concentration-time curve from time 0 to time t
BCG	Bacille Calmette-Guérin
BCR	B-cell receptor
BCRP	breast cancer resistance protein
BID	twice a day
BTK	Bruton's tyrosine kinase
C_{max}	maximum observed concentration
C_{trough}	steady-state concentration at the end of a dosing interval
CIU	chronic idiopathic urticaria
CL/F	apparent clearance
CRP	C-reactive protein
CSR	clinical study report
CSU	chronic spontaneous urticaria
CTCAE	Common Terminology Criteria for Adverse Events
CVA	cerebrovascular accident
DAP	data analysis plan
DLAE	dose-limiting adverse events
DLT	dose-limiting toxicity
EC	Ethics Committee
eCRF	electronic Case Report Form
EDC	electronic data capture
FcεRI	high affinity IgE receptor
FDA	Food and Drug Administration
GI	gastrointestinal
H2RA	H2 receptor antagonist
HBcAb	hepatitis B core antibody
HBsAg	hepatitis B surface antigen
IC_{70}	70% maximal inhibitory concentration
IC_{80}	80% maximal inhibitory concentration
IC_{90}	90% maximal inhibitory concentration
ICH	International Conference on Harmonisation
Ig	immunoglobulin

Abbreviation	Definition
IL-1	interleukin 1
IL-6	interleukin 6
IMC	Internal monitoring committee
IMP	investigational medicinal product
IND	Investigational New Drug (application)
IRB	Institutional Review Board
IV	intravenous
IVIG	intravenous immunoglobulin G
IxRS	interactive voice or web-based response system
LTRA	leukotriene receptor antagonist
MAD	multiple-ascending dose
MID	minimally important difference
mITT	modified intent-to-treat
MMRM	mixed model for repeated measures
NCI	National Cancer Institute
NOAEL	no observed adverse effect level
NSAID	non-steroidal anti-inflammatory drug
PD	pharmacodynamic
PK	pharmacokinetic
PO	by mouth, orally
PPD	Purified Protein Derivative
PPI	proton-pump inhibitor
PRO	patient-reported outcome
QD	once a day
QFT	QuantiFERON-TB-Gold
QTcF	QT interval corrected using Fridericia's formula
RA	rheumatoid arthritis
SAD	single-ascending dose
SLE	systemic lupus erythematosus
$t_{1/2}$	half-life
t_{max}	time to maximum concentration
TB	tuberculosis
[redacted]	[redacted]
THC	tetrahydrocannabinol
TNF-α	tumor necrosis factor alpha
UAS	Urticaria Activity Score
UAS7	Urticaria Activity Score over 7 days

Abbreviation	Definition
[redacted]	[redacted]
XLA	X-linked agammaglobulinemia

1. BACKGROUND

1.1 BACKGROUND ON CHRONIC SPONTANEOUS URTICARIA

Chronic spontaneous urticaria (CSU, also referred to as chronic idiopathic urticaria [CIU]) is defined by the presence of wheals (hives), angioedema, or both for at least 6 weeks without an obvious cause (Greaves 2003). Previous estimates of the prevalence of CSU were approximately 0.1%, which persists in 20% of CSU patients 2 decades after diagnosis (Greaves 2000; Saini 2014). More recent evidence indicates that the point prevalence of the disease is approximately 1% (Maurer et al. 2011). Affected patients experience frequent pruritic hives with associated erythema and/or episodes of angioedema. CSU is reported to be associated with angioedema in approximately 50% of cases (McGirt et al. 2006). The classic urticaria description is a wheal and flare with a pale elevated lesion and surrounding erythema, ranging in size from a few millimeters to a few centimeters across, usually occurring in groups and often coalescing to form large confluent lesions.

The etiology of CSU is not clear. There are several theories including one proposing an infectious origin and another related to an autoimmune origin (Kaplan 2002). Some studies have found that approximately 30%−60% of patients with CSU have an autoimmune component as evidenced by the presence of a positive autologous serum skin test (Fiebiger et al. 1995; Tong et al. 1997; Zweiman et al. 1998). Another hypothesis regarding the etiology of CSU is that of a specific IgE antibody targeted to an endogenous antigen (Altrichter et al. 2011). Crosslinking of this IgE antibody docked in the high affinity IgE receptor (FcεRI) could result in the activation of skin mast cells and release of chemical mediators, such as histamine, that lead to the wheal and flare formation of a hive. In fact, recent findings in a study of more than 450 patients with CSU indicate that greater than 50% of CSU patients have IgE antibodies directed against thyroperoxidase (Altrichter et al. 2011). While an autoimmune etiology can be found in a large percentage of patients, many patients do not have an identified autoimmune etiology despite having a similar disease presentation (Ferrer 2015).

The final common pathway in CSU is the abnormal activation of mast cells and basophils in the skin. In patients with CSU, increased numbers of mast cells can be found in both affected and unaffected skin (Kay et al. 2014). Furthermore, mast cells from CSU patients are more sensitive, have lower thresholds for activation, and respond more robustly by releasing more histamine and other inflammatory mediators. Similarly, increased numbers of basophils have been seen in the lesional and non-lesional skin of patients with CSU (Ying et al. 2002). In patients with CSU, there is a paradoxical basopenia thought to be due to increased recruitment of basophils to diseased skin. The peripheral basopenia is inversely correlated with severity of disease activity. In comparison with healthy controls, studies have shown that blood basophils of CSU patients have a reduced capacity to release histamine following IgE stimulation. This paradoxical reduction is attributed to prior in vivo activation in the skin (Kern and Lichtenstein 1976). However, when basophils from CSU patients are incubated with

serum from other CSU patients or even normal sera, they release more histamine than basophils from healthy donors. Collectively, these data suggest that basophil signaling and activation are dysregulated in patients with CSU (Luquin et al. 2005).

Roughly half of patients with CSU achieve symptomatic control with H1 antihistamine therapy at approved doses. In some cases, the dose of antihistamine is increased (up to 4 times the approved dose per local treatment guidelines) and additional therapies, such as leukotriene receptor antagonists (LTRAs), are used although increased doses of antihistamines and LTRAs are not approved for the treatment of CSU. CSU can be a debilitating condition because of a lack of clinical response as well as the unpredictable course of the disease, both of which can have a profound negative influence on the patient's quality of life (Tilles 2005).

Patients may remain symptomatic despite ongoing H1 antihistamine treatment (up to 4 times the approved dose per local treatment guidelines; Powell et al. 2015), and for this group of patients, therapies such as immunosuppressants (including cyclosporine, corticosteroids, intravenous immunoglobulin G [IVIG], and methotrexate) and plasmapheresis have been used (Kozel and Sabroe 2004). These agents have variable success and may be associated with severe adverse effects. More recently, omalizumab was approved for treatment of refractory CSU/CIU.

1.2 BACKGROUND ON BRUTON'S TYROSINE KINASE AND FENEBRUTINIB

1.2.1 Bruton's Tyrosine Kinase

Discovery of the genetic basis for primary immunodeficiencies has been the source of new therapeutic targets in immunomodulatory therapies (Puri et al. 2013; Bugatti et al. 2014; Whang and Chang 2014). In humans, mutations in the gene for Bruton's tyrosine kinase (BTK), which is located on the X chromosome, can result in the development of an immunodeficiency state characterized by a significant absence of circulating B cells (Bruton 1952; Tsukada et al. 1993; Vetrie et al. 1993; Conley et al. 2005) and very low immunoglobulin levels due to a defect in B-cell differentiation at the pro– to pre–B cell stage that precludes assembly of the B-cell receptor (BCR) complex and immunoglobulin gene expression (Reth and Nielsen 2014). Affected male patients have a primary immune deficiency, X-linked agammaglobulinemia (XLA), and are susceptible to recurrent infections starting shortly after birth. Patients with XLA can live relatively normal lives on a standard therapy of intravenous (IV) immunoglobulin, which suggests that BTK can be safely inhibited, especially in people with established immune systems. IV immunoglobulin replacement therapy lowers the rate of infection, reduces hospitalization rates for patients with XLA, and has greatly improved the long-term prognosis of these patients.

BTK is essential for the differentiation and activity of B cells during immune system ontogeny and normal adaptive immune responses. BTK is activated by phosphatidylinositol 3-kinase–dependent plasma membrane recruitment and

phosphorylation on tyrosine Y551 by the Src-family kinase Lyn. Autophosphorylation and activation also occurs on tyrosine Y223 in a BTK-specific manner. Once activated, BTK induces PLCγ2- and Ca^{2+}-dependent signaling, which leads to the activation of NF-κB– and NFAT-dependent pathways leading to cellular activation and differentiation (Niiro and Clark 2002). In addition, BTK is important in FcεRI signaling in both basophils and mast cells, the key cell types in the pathogenesis of CSU. BTK null mice have impaired FcεRI signaling resulting in decreased histamine and inflammatory cytokine release (Hata et al. 1998; Iyer et al. 2011).

1.2.2 Nonclinical Experience with Fenebrutinib

Fenebrutinib (also referred to as GDC-0853 or RO7010939) is a highly selective, orally administered, reversible inhibitor of BTK that is being developed by Genentech, Inc. as a potential therapeutic for autoimmune diseases, including CSU. Fenebrutinib has undergone extensive investigation in nonclinical in vitro and in vivo studies to characterize its pharmacological, metabolic, and toxicological properties (see the Fenebrutinib Investigator's Brochure for further details).

In vitro cell-based experiments suggest that antagonism of BTK leads to inhibition of BCR-dependent B-cell proliferation and a reduction of inflammatory cytokine production from myeloid cells (including tumor necrosis factor alpha [TNF-α], interleukin 1 [IL-1], and interleukin 6 [IL-6]) by preventing signaling through the FcγRIII receptor (Di Paolo et al. 2011; Liu et al. 2011). Fenebrutinib effectively blocks BCR- and CD40-mediated activation and proliferation of B cells. BTK in B cells also plays a role in TLR4-mediated B-cell proliferation and class switching. In monocytes, fenebrutinib inhibits TLR4- and immune complex-mediated inflammatory cytokine production, including TNF-α, which contributes to disease pathogenesis in rheumatoid arthritis (RA).

As described above, the pathophysiology of CSU is not completely understood. A key step is FcεRI-activation and release of histamine and inflammatory cytokines from mast cells and basophils, leading to the wheal and flare formation of a hive as well as angioedema. In support of the importance of BTK in FcεRI signaling and the pathogenesis of CSU, BTK null mice have impaired FcεRI signaling, resulting in decreased histamine and inflammatory cytokine release (Hata et al. 1998; Iyer et al. 2011).

Consistent with these findings, in vitro experiments with human mast cell lines demonstrated that fenebrutinib could effectively inhibit the release of histamine induced by cross-linking of IgE bound to FcεRI on the surface of mast cells. In addition, in a Phase Ib study in healthy volunteers, oral administration of fenebrutinib inhibited ex vivo basophil activation as measured by diminished cell surface expression of CD63 after cross-linking of IgE. As such, fenebrutinib inhibits the activity of two specific cell types that play key roles in disease pathology in CSU.

The fenebrutinib safety profile has been assessed in repeat-dose, general toxicology studies (once a day [QD] oral dosing) ranging from 1 week to 9 months in rats and dogs; in vitro and in vivo genetic toxicology studies; in vitro phototoxicity evaluation; in vitro and in vivo safety pharmacology studies of the central nervous, respiratory, and cardiovascular systems; and embryo-fetal development (Seg II) studies in rats and rabbits. Overall, fenebrutinib was well tolerated for up to 6 months in rats (up to 104 μM•hr) and up to 9 months in dogs (up to 36 μM•hr). Notable findings identified in nonclinical toxicology studies include vascular inflammation (≥56 μM•hr) in dogs, hepatotoxicity (180 μM•hr) in dogs and rats, and a minimal increase in corrected QT interval (QTc; 7 ms or 3%; extrapolated unbound maximum observed concentration [C_{max}] of 3.17 μM) in dogs. Fetal malformations in rats (at 627 μM•hr) and rabbits (≥10.6 μM•hr) warrant the continued use of highly effective contraception in clinical trials. On the basis of the nonclinical and clinical safety data to date, fenebrutinib is expected to be well tolerated at the doses and duration administered in the current study.

1.2.3 Clinical Experience with Fenebrutinib

As of 12 March 2018, fenebrutinib or placebo has been administered to 1099 subjects (i.e., 333 healthy subjects, 24 patients with hematological malignancies, 576 patients with rheumatoid arthritis, 129 patients with systemic lupus erythematosus, and 37 patients with chronic spontaneous urticaria) at doses with a range of 0.5–600 mg and has been generally well tolerated with no safety concerns that have led to a change in the conduct of the studies.

Study GO29089 is a Phase I, open-label study in which fenebrutinib has been evaluated in patients with relapsed or refractory B-cell non-Hodgkin's lymphoma or chronic lymphocytic leukemia. In order to focus on the autoimmune indications, Genentech elected to stop development of fenebrutinib in oncology, and the Phase I study is continuing without further patient enrollment. Enrollment was stopped after completion of the 400-mg dose level at which time 24 patients had been enrolled in 3 cohorts: 100 mg, 200 mg, and 400 mg fenebrutinib daily. Seven patients remain in the study, and all have undergone intrapatient dose escalation to 400 mg QD fenebrutinib. The mean duration of daily dosing for these 7 patients has been 21 months (range of 18–23 months). Fenebrutinib was well tolerated with no dose-limiting toxicities (DLTs), maximum tolerated dose was not reached, and adverse events have been generally non-serious National Cancer Institute Common Terminology Criteria for Adverse Events, Version 4.0 (NCI CTCAE v4.0) Grade 1 or Grade 2 events that have been clinically manageable. The adverse events regardless of causality reported in ≥15% of patients include fatigue, nausea, diarrhea, headache, abdominal pain, dizziness, cough, and thrombocytopenia. As of August 2016, 11 serious adverse events had been reported in 5 patients, of whom 2 had a fatal outcome (i.e., complications of H1N1 influenza and influenza pneumonia).

Study GP29318 was a two-part, single-ascending dose (SAD) study to assess the safety, tolerability, and pharmacokinetics of fenebrutinib administered to 93 healthy subjects. In Part 1, the single-dose–escalation portion, 71 subjects were randomized to panels of 8 subjects (6:2 active:placebo ratio) per dose group (0.5 – 600 mg), with 53 subjects receiving active fenebrutinib. In Part 2, 100 mg fenebrutinib was administered to 40 subjects in the open-label food and pilot rabeprazole effect study. There were no serious adverse events and no withdrawals due to adverse events during the conduct of Study GP29318. In Part 1 of the study, there were no dose-limiting adverse events (DLAEs) at single doses up to 600 mg fenebrutinib. All adverse events were mild in intensity (Grade 1; Toxicity Grading Scale for Healthy Adult and Adolescent Volunteers Enrolled in Preventive Vaccine Clinical Trials) and transient. No adverse events increased in intensity or frequency with dose escalation. There were two treatment-emergent adverse events of mild self-limited headache reported as related to fenebrutinib administration. There were no trends in safety laboratory findings, vital sign changes, physical examination findings, or ECG changes. There were no trends in hepatic laboratory changes following single doses of fenebrutinib in healthy subjects. Administration of fenebrutinib inhibited ex vivo basophil activation as demonstrated by diminished CD63 cell surface expression after cross-linking of IgE. Refer to the Fenebrutinib Investigator's Brochure for further information on Study GP29318, including pharmacokinetics.

Study GA29347 was a multiple-ascending dose (MAD) study to assess the safety, tolerability, and pharmacokinetics of multiple doses of fenebrutinib administered to 30 healthy subjects for 14 days. Forty subjects were randomized to panels of 8 subjects (6:2 active:placebo) per dose group, at doses of 20 mg twice a day (BID), 60 mg BID, 150 mg BID, 250 mg BID, or 500 mg QD for 14 days, with 30 subjects receiving active fenebrutinib. The study drug was well tolerated. There were no serious adverse events and no withdrawals due to adverse events during the conduct of the study. All adverse events were mild in intensity (Grade 1) and transient, with no relationship to dose. Adverse events included skin reactions (i.e., rash, contact dermatitis, and skin irritation from ECG leads), nausea, headache, insomnia, toothache, tinnitus, and asymptomatic bacteriuria. There were no trends in safety laboratory, vital sign, physical examination, or ECG findings. Similar to the SAD study, dose-dependent inhibition of CD63 expression was observed following fenebrutinib administration, with sustained inhibition over the duration of dosing.

Study GP29832 was a relative bioavailability study designed to evaluate the effects of formulation, food, and proton-pump inhibitor (PPI) or methotrexate co-administration on the pharmacokinetics of fenebrutinib in healthy subjects. Fenebrutinib was well tolerated when administered to 48 healthy subjects at the 200-mg dose level.

Study GA29350 is a multicenter Phase II dose ranging study comparing the efficacy and safety of fenebrutinib versus placebo and adalimumab in patients with RA who have had an inadequate response to previous methotrexate therapy and versus placebo in

patients with an inadequate response to previous tumor necrosis factor therapy. The study began enrollment in September 2016, and the total planned enrollment is approximately 580 patients.

Study GA30044 is the first study investigating fenebrutinib in systemic lupus erythematosus (SLE). This is a multicenter, Phase II, randomized, double-blind, placebo-controlled, parallel-group, dose-ranging study designed to evaluate the efficacy and safety of fenebrutinib in patients with moderate-to-severe active SLE in combination with standard-of-care therapy.

The drug-drug interaction Study GP39616 has recently concluded. While final results are not yet available, preliminary data suggests:

- Fenebrutinib is a Breast Cancer Resistance Protein (BCRP) inhibitor. Consequently, fenebrutinib may alter transport of BCRP substrates and result in increased plasma concentrations of BCRP substrates.

The study also confirmed the following:

- Fenebrutinib is a mild inhibitor of CYP3A at clinically relevant doses. Consequently, fenebrutinib may alter metabolism of CYP3A substrates and result in increased plasma concentrations of CYP3A substrates.
- Fenebrutinib is a moderately sensitive substrate of CYP3A at clinically relevant doses. There is a moderate potential for levels of fenebrutinib to increase in patients taking concomitant medications that inhibit CYP3A and decrease in patients taking medications that induce CYP3A.

Refer to the Fenebrutinib Investigator's Brochure for detailed background information on fenebrutinib as well as for additional details on nonclinical and clinical studies.

1.3 STUDY RATIONALE AND BENEFIT-RISK ASSESSMENT

Omalizumab, an anti-IgE monoclonal antibody, has demonstrated efficacy in treating patients with CSU, highlighting the key role of IgE in CSU pathogenesis. Inhibition of IgE-mediated FcεRI signaling by BTK inhibition offers a promising mechanism for the treatment of CSU. In preclinical in vitro studies, BTK inhibition was able to prevent the release of histamine and inflammatory cytokines from mast cells after FcεRI engagement and cross-linking. In addition, in the healthy volunteer SAD and MAD studies, oral administration of fenebrutinib was able to inhibit ex vivo basophil activation as demonstrated by diminished CD63 cell surface expression. As such, fenebrutinib inhibits two key pathogenic cell types in CSU. The aim of this study is to determine if fenebrutinib can effectively treat patients with CSU, as measured by reduction in disease activity.

Humans with XLA lack functional BTK but can live relatively normal lives on a standard therapy of IV immunoglobulin (Kaveri et al. 2011), suggesting that BTK can be safely inhibited in patients with CSU who have functional immune systems. Clinical experience

with fenebrutinib to date has not generated safety concerns that would preclude further evaluation in patients with CSU. In the SAD (Study GP29318), MAD (Study GA29347), relative bioavailability (Study GP29832), and oncology (Study GO29089) studies, fenebrutinib was well tolerated with no DLAEs or DLTs. In the oncology study, there were 2 deaths due to complications of confirmed influenza (i.e., H1N1 influenza and influenza pneumonia).

Several measures will be taken to ensure the safety of patients participating in this study based on the potential risks for fenebrutinib based on nonclinical and clinical studies and published literature (see Section 5.1.1). Eligibility criteria have been designed to exclude patients at higher risk for potential toxicities. In addition, the sites selected for this study will be specialty dermatology and immunology clinics with experience in treating CSU.

1.3.1 Infections

Fenebrutinib is a targeted immunomodulator; however, as a reversible inhibitor, the degree to which fenebrutinib antagonism of BTK signaling may suppress immune activity is unknown. Patients participating in this study may be at risk for infections, including opportunistic infections. Therefore, patients at high risk for infection will be excluded (see Section 5.1.1.1). Patients will be carefully monitored throughout the study for infections. Fenebrutinib will be discontinued in any patient who develops a serious infection, opportunistic infection, or any infection requiring treatment with an IV antimicrobial agent.

1.3.2 Bleeding

BTK is expressed in platelets and is involved in platelet function via GPVI/collagen receptor signaling and GP1b receptor signaling. Platelets from patients with XLA demonstrate decreased activation in response to submaximal collagen stimulation but normal response to thrombin; clinically, there is no reported bleeding propensity in patients with XLA. In the fenebrutinib clinical study involving oncology patients (GO29089), 2 patients experienced Grade ≥ 3 gastrointestinal (GI) bleeding. These events were not dose related and occurred in patients on non-steroidal anti-inflammatory drugs (NSAIDs)/acetylsalicylic acid with a history of gastroesophageal or peptic ulcer disease.

It is unknown whether fenebrutinib will increase the risk of bleeding in patients with CSU who receive antiplatelet or anticoagulant therapies. Therefore, the eligibility criteria exclude patients at high risk for bleeding complications.

1.3.3 Cytopenias

Neutropenia, anemia, and thrombocytopenia have been observed in patients with hematologic malignancies who received fenebrutinib. No clinically significant changes in cell counts were observed in the healthy volunteer studies. Events have been monitorable and clinically manageable. Cell counts will be monitored regularly throughout the study.

1.3.4 Hepatotoxicity

Evidence of hepatobiliary injury was observed in animals administered relatively high doses of fenebrutinib in repeat-dose toxicity studies. In clinical studies to date, in autoimmune indications, enrolling over 800 patients, multiple cases of treatment emergent Grade 3 (or severe) elevations of ALT, some of which were considered serious adverse events, have been observed in the randomized clinical studies, which remain blinded to the Sponsor in terms of treatment assignment, as well as in open label extensions of fenebrutinib. These cases have been seen in blinded studies in CSU. None of the cases of transaminase elevations resulted in clinical jaundice or bilirubin $> 2 \times$ ULN (Hy's Law). All transaminase elevations have been reversible when dosing of blinded study medication/placebo was withheld. These findings have not been seen in single dose and multiple dosing for 14 days in healthy subjects and QD dosing in patients with hematological malignancies. To minimize this risk, exclusion criteria have been defined for abnormal liver enzyme and function tests and current liver disease (see Section 4.1.2). For further information on nonclinical and clinical findings of hepatotoxicity, please see the Fenebrutinib Investigator's Brochure.

1.3.5 Cardiovascular Effects

Fenebrutinib is considered to have a low potential to cause QT interval prolongation or to directly affect other cardiovascular parameters at therapeutic exposures. A minimal increase in corrected QT (QTc; 7 ms or 3%) interval was noted at 45 mg/kg in the single-dose cardiovascular safety pharmacology study in telemetry-instrumented dogs. Cardiac safety will be evaluated in all patients at baseline and throughout the study, with routine monitoring of vital signs, including heart rate and blood pressure, collection of ECGs, and reporting of cardiac adverse events.

1.3.6 Malignancy

The impact of BTK inhibition on the development of malignancies is not known; however, malignancies are considered a potential concern for all immunomodulatory agents. Patients with a history of cancer within 10 years of screening will be excluded from study participation, except for basal or squamous cell carcinoma of the skin that has been excised and is considered cured and in situ carcinoma of the cervix treated with apparent success by curative therapy more than 1 year prior to screening.

Overall, fenebrutinib has been well tolerated in Phase I healthy subjects and an oncology study. On the basis of the compelling mechanism for BTK inhibition in CSU,

the benefit-risk ratio for this study is deemed acceptable. The safety profile of fenebrutinib will be further characterized in this Phase II study. A robust safety monitoring plan that describes the potential risks for fenebrutinib and the risk-mitigation strategies to minimize risks for the patients in this trial is provided in Section 5.

Please refer to the most recent Fenebrutinib Investigator's Brochure for additional details on clinical and nonclinical studies and additional safety information.

2. OBJECTIVES AND ENDPOINTS

This pilot and dose-ranging study will evaluate the efficacy, safety, and pharmacokinetics of fenebrutinib compared with placebo in patients with CSU refractory to anti-histamines (up to 4 times the approved dose per local treatment guidelines). Specific objectives and corresponding endpoints for the study are outlined in Table 1.

Table 1 Objectives and Corresponding Endpoints

Objectives	Corresponding Endpoints
**Efficacy Objective:**	
• To evaluate the efficacy of fenebrutinib compared with placebo in patients with CSU who are refractory to anti-histamines	**Primary Endpoint:**<br>• Change from baseline in the UAS7 at Day 57 (Week 8)<br>**Secondary Endpoints:**<br>• Proportion of patients who are well controlled (UAS7 $\leq$6) at Day 57<br>• Change from baseline in the UAS7 at Day 29 (Week 4)<br>**Exploratory Endpoint:**<br>• Change from baseline in the weekly itch score at Day 29<br>• Change from baseline in the weekly itch score at Day 57<br>• Change from baseline in the weekly hives score at Day 57<br>• Proportion of patients who are well controlled (UAS7 $\leq$6) at Day 29<br>• Proportion of patients who achieve complete response (UAS7 = 0) at Day 29<br>• Proportion of patients who achieve complete response (UAS7 = 0) at Day 57<br>• Proportion of patients achieving MID in UAS7 at Day 57 (reduction from baseline $\geq$11 points)<br>• Proportion of patients achieving MID in the weekly itch score at Day 57 (reduction from baseline $\geq$5 points)<br>• Time to achieving MID in UAS7 (reduction from baseline $\geq$11 points)<br>[redacted]

Table 1 Objectives and Corresponding Endpoints (cont.)

Objectives	Corresponding Endpoints
**Safety Objective:**	
• To evaluate the safety of fenebrutinib compared with placebo	• The nature, frequency, timing, and severity of adverse events<br>• Change from baseline in targeted vital signs, physical examination findings, ECGs, and clinical laboratory results following fenebrutinib administration
**Pharmacokinetic Objective:**	
• To characterize the pharmacokinetics of fenebrutinib in patients	• Plasma concentrations of fenebrutinib at specified timepoints
[redacted]	
[redacted]	[redacted]
[redacted]	[redacted]
[redacted]	[redacted]
[redacted]	
[redacted]	[redacted]

AUC_{0-t} = area under the concentration–time curve from time 0 to time t; [redacted] CL/F = apparent clearance; C_{max} = maximum concentration observed; C_{trough} = steady-state concentration at the end of a dosing interval; CSU = chronic spontaneous urticaria; Ig = immunoglobulin; MID = minimally important difference [redacted] PK = pharmacokinetic; $t_{1/2}$ = half-life; t_{max} = time to maximum concentration; UAS7 = Urticaria Activity Score over 7 days; [redacted]

3. STUDY DESIGN

3.1 DESCRIPTION OF THE STUDY

This pilot and dose-ranging study is a multicenter, randomized, double-blind, placebo-controlled, parallel-group study of the efficacy and safety of fenebrutinib as

add-on therapy for the treatment of adult patients 18 – 75 years old who have been diagnosed with CSU and who remain symptomatic despite treatment with H1 antihistamines (including doses up to 4 times the approved dose per local treatment guidelines). The study will consist of two cohorts. Cohort 1 will enroll approximately 45 patients across multiple sites. After screening, eligible patients will be randomly allocated in a 2:1 ratio to receive fenebrutinib 200 mg orally (PO) twice daily (BID) or matching placebo for 8 weeks and will maintain stable doses of standard-of-care H1 antihistamine therapy throughout the study. On the basis of results from an interim analysis of Cohort 1, a dose-ranging cohort, Cohort 2, will be opened and approximately 120 patients will be randomly allocated in a 1:1:1:1 ratio to receive 50 mg PO daily (QD), 150 mg PO QD, 200 mg PO BID of fenebrutinib, or placebo, respectively, for 8 weeks and will maintain stable doses of standard-of-care H1 antihistamine therapy (background therapy) throughout the study.

Both cohorts will consist of 3 distinct study periods over a time-period of 14 weeks as outlined below (see Figure 1):

- Screening period: Day –14 to Day –1
- Treatment Period: Day 1 to Day 57 (Week 0 to Week 8)
- Follow-Up Period: Day 57 to Day 85 (Week 8 to Week 12)

Patients in both cohorts will have a screening period of approximately 2 weeks to establish their eligibility for the study and baseline symptom scores. For the duration of the screening period, patients must maintain stable doses of their pre-screening combination therapy with standard-of-care H1 antihistamines (i.e., up to 4 times the approved dose per local treatment guidelines). The screening period will consist of visits at Day –14 and Day –7. Patients must meet all of the following criteria to enter the screening period:

- Documented treatment with a regimen that includes standard-of-care H1 antihistamine for CSU at Day –14 and for at least the 3 consecutive days immediately prior to Day –14 (see Section 4.4.1 for list of H1 antihistamines available for use in this study)
- Willing and able to complete a symptom electronic diary (Urticaria Patient Daily eDiary) twice daily throughout the screening period to establish the patient's Urticaria Activity Score over 7 days (UAS7) score.

To be eligible for randomization in both cohorts, for the 7 days prior to randomization, patients must meet all of the following:

- Seven consecutive days of entries in the Urticaria Patient Daily eDiary, and
- UAS7 symptom score of ≥ 16 (range: 0 – 42)

Only in exceptional circumstances, when information concerning eligibility is outstanding (e.g., delayed laboratory results), will a longer screening period be permitted up to

3 business days. Upon approval from the Medical Monitor, patients may be re-screened or maybe retested during the screening period (see Section 4.5.2.2 and Section 4.5.2.1, respectively). Circumstances that may permit re-screening or retesting include, but are not limited to, a laboratory test result that does not meet eligibility requirements.

The primary efficacy endpoint will be at Day 57 (Week 8). Throughout the treatment period, patients must maintain stable doses of their pre-randomization H1 antihistamine therapy.

After completion of the 8-week treatment period, patients in both cohorts will enter a 4-week safety follow-up period to allow for further characterization of the pharmacokinetics and pharmacodynamics of fenebrutinib and collection of additional efficacy and safety data. During safety follow-up period, no study treatment will be given; patients must maintain stable doses of their pre-randomization CSU H1 antihistamine treatment (background therapy). In the safety follow-up period, patients may add up to one additional H1 antihistamine therapy in case of worsened symptoms (see Section 4.4.3). The goal of allowing additional H1 antihistamine therapy after the treatment period is to reduce patient dropout for improved safety evaluation.

In addition to their daily background therapy, for the duration of the study, all patients will be able to use a single approved dose of loratadine (10 mg maximum) or cetirizine (10 mg maximum) within a 24-hour period as rescue medication if symptoms worsen. If a patient needs rescue therapy and is already on background treatment with cetirizine or loratadine, the patient may receive 10 mg more of the same drug only if the total daily dose remains below 4 times the approved dose. Otherwise, the alternate rescue medication may be used. Patients should record the use of this medication in their eDiary. Patients receiving PPIs or H2 receptor antagonists (H2RAs) should be stabilized on a regimen beginning at least 2 weeks prior to randomization and continuing throughout the study (see Section 4.4.2.1).

Figure 1 Study Schema

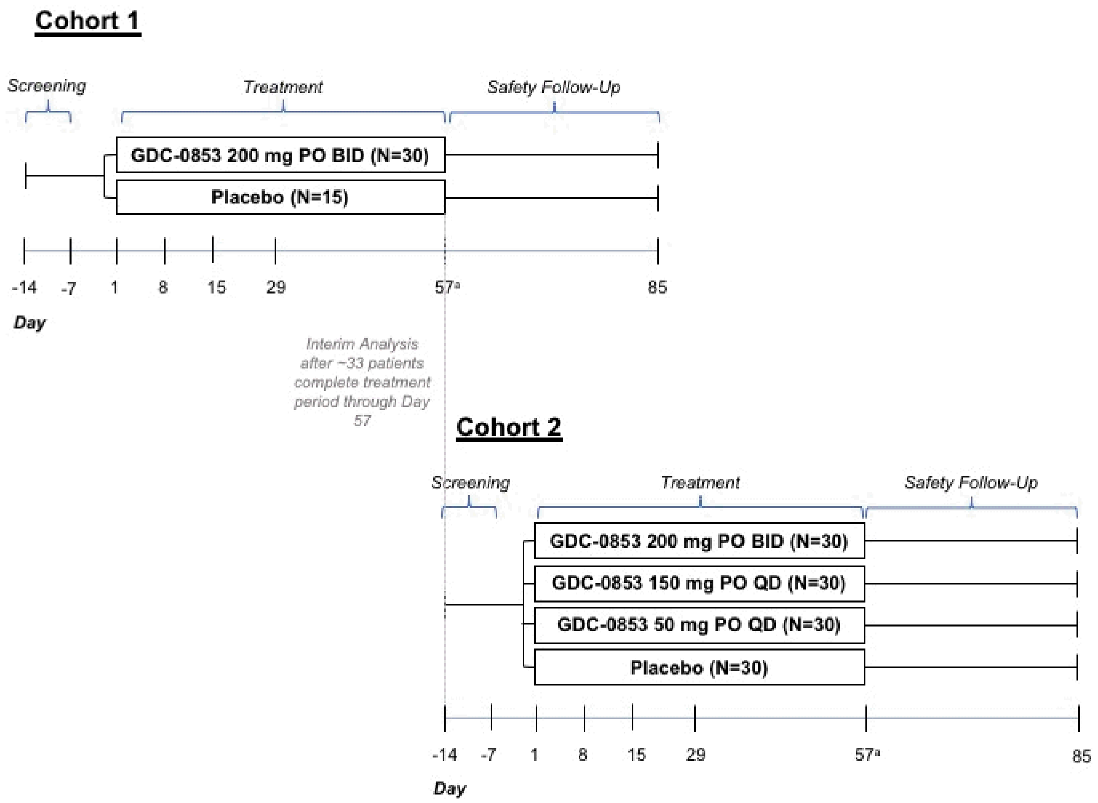

BID = twice daily; PO = orally; QD = daily.

[a] Last blinded dose = p.m. before Day 57 visit

3.1.1 Internal Monitoring Committee

For Cohort 2, periodic safety reviews and any interim analysis will be performed by the Sponsor's internal monitoring committee (IMC) as outlined in the IMC charter. This committee will be unblinded to treatment assignments and will include Sponsor representatives from the following functions: Clinical Science, Drug Safety, Biostatistics, and Statistical Programming and Analysis. The IMC members will not have direct contact with investigational staff or site monitors. The IMC may decide to unblind the study team to enable decision-making and potential interactions with regulatory bodies. The IMC may invite representatives from other functional areas on an ad-hoc basis when additional expertise is required (e.g., Clinical Pharmacology, Research, etc.) or additional Sponsor scientists to participate in data analyses and review.

At any time during the study, the Sponsor may choose to inactivate and suspend enrollment and further dosing for a given treatment arm (in Cohort 2) or reduce the dose due to safety concerns and as guided by the IMC. In Cohort 2, subsequently enrolled patients will be randomly allocated to the remaining active arms.

3.2 END OF STUDY AND LENGTH OF STUDY

The end of this study is defined as the date when all patients have completed the study completion visit or early termination visit or have otherwise been discontinued from the study. The total duration of this study for each subject is approximately 14 weeks (for both cohorts), including screening, treatment, and safety follow-up periods.

The total length of the study, from screening of the first patient to the end of the study, is expected to be approximately 19 months.

3.3 RATIONALE FOR STUDY DESIGN

3.3.1 Rationale for Fenebrutinib Dose and Schedule

In the proposed study for Cohort 1, patients randomly allocated to the fenebrutinib arm will receive 200 mg of fenebrutinib PO BID for 8 weeks. This dose is expected to be well tolerated and to substantially inhibit BTK activity, based on results from the Phase I studies. Dose-dependent target inhibition was demonstrated in the Phase I SAD and MAD studies with use of pharmacodynamic (PD) assays (phospho-BTK and basophil CD63 assays). On the basis of the pharmacokinetic (PK) and PK/PD models constructed using data from the relative bioavailability, SAD, and MAD healthy volunteer studies and of the tolerability of dosing up to 250 mg BID, the 200 mg BID dose is expected to provide a steady-state exposure achieving 90% maximal inhibitory concentration (IC_{90}) over the entire dosing interval in greater than 75% of patients. It should be noted that the extent of target engagement required for clinical efficacy is unknown. However, Cohort 1 is a pilot study, the dose tested in Cohort 1 will enable initial assessment of clinical efficacy in CSU.

Because the extent of target engagement required for clinical efficacy is unknown, doses for Cohort 2 were selected to evaluate a range of target engagement and to characterize the dose- and exposure–response relationships for safety and efficacy in order to select the optimal dose for further study.

The fenebrutinib dose levels proposed for Cohort 2 were selected based on target engagement (phospho-BTK and basophil CD63 assays) observed in the Phase 1 SAD and MAD studies as well as an in vitro assessment of FcεRI-dependent histamine release from LAD-2 mast cells. The rationale for the dose regimens is as follows:

- The 50-mg QD dose is expected to provide systemic exposures that will achieve 70% maximal inhibitory concentration (IC_{70}) based on the basophil CD63 assay for half of the dosing interval in approximately 50% of patients.
- The 150-mg QD dose is expected provide an exposure achieving IC_{70} based on the basophil CD63 assay for half of the dosing interval in approximately 90% of patients.

- The 200-mg BID dose is the Proof of Concept dose carried forward from Cohort 1. This dose is expected to provide a steady-state exposure achieving 90% maximal inhibitory concentration (IC_{90}) based on the basophil CD63 assay and approximately 80% maximal inhibitory concentration (IC_{80}) based on the LAD-2 mast cell FcεRI-dependent histamine release assay over the entire dosing interval in the majority of patients.

A fumaric acid tablet formulation of fenebrutinib will be used in this study. In the relative bioavailability Study GP29832, the tablet formulation decreased PK variability and appeared to improve absorption by mitigating pH-dependent solubility compared with the powder-in-capsule formulation. The proposed highest dose in both cohorts, 200 mg BID (fumaric acid tablet), is expected to achieve exposures (area under the concentration–time curve [AUC] and C_{max}) that are in the range of those observed following administration of 250 mg BID (powder-in-capsule) in Study GA29347 and 5-fold below the exposures at the no observed adverse effect level (NOAEL) of the nonclinical chronic toxicity studies (see the Fenebrutinib Investigator's Brochure).

Study GP29832 also characterized the effect of food and the effect of the PPI rabeprazole on the pharmacokinetics of fenebrutinib. A moderate-fat meal did not substantially change the systemic exposure following a single administration of 200 mg fenebrutinib, since the fumaric acid tablet formulation (fed:fasted geometric mean ratio 90% CI) for area under the concentration–time curve from time 0 to 24 hours (AUC_{0-24}) and C_{max} were 1.08 (0.97–1.22) and 0.85 (0.72–1.01), respectively, supporting administration without regard to food. Co-administration of multiple doses of rabeprazole with a single dose of 200 mg fenebrutinib with the fumaric acid tablet formulation decreased fenebrutinib systemic exposure (AUC_{0-24}) by approximately 32% and 44% in the fasted and fed states, respectively, compared with tablet alone. In order to evaluate PK in a relevant patient population, stable use of a PPI regimen will be permitted during the study.

3.3.2 Rationale for Patient Population

Patients enrolled in both cohorts of the study will have a CSU diagnosis for $\geq$ 6 months and will be refractory to treatment with a combination of H1 antihistamines consistent with standard of care (i.e., up to 4 times the approved dose per local treatment guidelines) as demonstrated by the presence of itch and hives for $>$6 consecutive weeks on this treatment at any time prior to enrollment. In addition, patients will have a UAS7 score of $\geq$16 during the 7 days prior to randomization despite current use of combination therapy.

While H1 antihistamines are the mainstay of therapy for CSU, some patients do not respond or respond only partially to these therapies, and these patients tend to experience more severe disease. This patient population was selected for this study because of the unmet medical need for more effective oral treatments.

3.3.3 Rationale for Control Group

A placebo-treated control group is required for this study in order to achieve its efficacy and safety objectives given the inherent variability in symptoms and the different rates of improvement in the placebo arms of prior studies. Patients in the placebo arm will continue to receive stable standard-of-care H1 *antihistamine* therapy throughout the study. In addition, the study will allow for rescue medications for persistent symptoms.

[redacted]

3.3.5 Rationale for Pharmacokinetic Sample Collection Schedule

The sampling schedule is designed to assess multiple pre-dose (prior to study drug administration in clinic) plasma fenebrutinib concentrations, which will enable the estimation of systemic fenebrutinib exposures and subsequent exposure-response analyses, both of which may be reported separately. Results will be used to inform dosing regimens for future studies of fenebrutinib.

3.3.6 Rationale for Efficacy Endpoints

The change in the UAS7 (see Table 2 for daily assessment of Urticaria Activity Score [UAS]) has been chosen as the primary efficacy endpoint as it has been used in pivotal trials in CSU to measure reduction in CSU disease severity. The UAS7 is a summation of the average daily (a.m./p.m.) scores on the UAS (range: 0–6), which is a composite diary score with numeric severity intensity ratings on a scale of 0–3 (0 = none to 3 = intense/severe) for two domains: the intensity of the itch and the number of wheals/hives (see Table 2). The UAS will be recorded by the patient twice daily (morning and evening) in the patient Urticaria Patient Daily eDiary. UAS7 scores range from 0–42 and the minimally important difference (MID) is considered to be a reduction from baseline of ≥9.5 to 10.5 points (Mathias et al. 2012). The baseline UAS7 is the sum of the daily scores on the UAS over the 7 days prior to randomization (Day 1 visit for both cohorts), and the UAS7 at Day 57 is the sum of daily scores on the UAS over the 7 days prior to the Day 57 visit (for both cohorts). The same principles of calculating baseline and Day 57 weekly scores will be applied to the other weekly outcomes unless otherwise stated.

The kinetics of response to fenebrutinib will also be carefully evaluated throughout the course of the study at regular intervals (at least every 1–2 weeks for a period of 8 weeks). In addition, disease recurrence or duration of treatment benefit after study drug is withdrawn during the safety follow-up period will be measured during this study. This will provide initial guidance for the duration of therapy in future studies.

Table 2 **Twice Daily Patient Assessment of CSU Disease Activity (UAS Scale)**

Score	Wheals (Hives)	Pruritus (Itch)
0	None	None
1	Mild (1–6 hives/12 hour)	Mild
2	Moderate (7–12 hives/12 hour)	Moderate
3	Intense (> 12 hives/12 hour)	Severe

CSU = chronic spontaneous urticaria; UAS = Urticaria Activity Score.

4. MATERIALS AND METHODS

4.1 PATIENTS

Approximately 45 patients in Cohort 1 and approximately 120 patients in Cohort 2, aged 18 to 75 years old who have been diagnosed with refractory CSU and who remain symptomatic despite standard-of-care H1 antihistamine therapy (i.e., up to 4 times the approved dose per local treatment guidelines), will be enrolled in this study.

4.1.1 Inclusion Criteria

Patients in Cohort 1 and 2 must meet the following criteria for study entry:

- Willing to give written informed consent, adhere to the visit schedules, comply with the study drug regimen, and meet other study requirements
- Aged 18–75 years, inclusive
- Diagnosis of CSU refractory to H1 antihistamines at the time of randomization, as defined by all of the following:
  - The presence of itch and hives for > 6 consecutive weeks at any time prior to enrollment despite current use of H1 antihistamines, consistent with standard of care (i.e., up to 4 times the approved dose per local treatment guidelines) during this time period
  - UAS7 score ≥ 16 during the 7 days prior to randomization (Day 1)
  - Patients must have been on daily stable doses of H1 antihistamines, consistent with standard-of-care therapy (i.e., up to 4 times the approved dose per local treatment guidelines) for CSU starting at least 3 consecutive days immediately prior to the screening visit through Day 1 and must document current use on all visits.
  - CSU diagnosis for ≥ 6 months
- Willing and able to complete an Urticaria Patient Daily eDiary for the duration of the study
- Completion of 7 days of the Urticaria Patient Daily eDiary entries in the 7 days prior to randomization (7 of 7 days must be completed [i.e., must complete an entry every day] with up to 2 non-consecutive entries missed)

- No evidence of active or latent or inadequately treated infection with tuberculosis (TB) as defined by the following:
  - A negative QuantiFERON-TB-Gold® (QFT) performed (for German sites only: QFT is the preferred test) at the screening visit or within the 3 months prior to screening

    If QFT is unavailable, a negative Mantoux purified protein derivative (PPD) skin test as defined by the Centers for Disease Control and Prevention guidelines, may be performed at the screening visit or within the 3 months prior to screening -AND-

    Any additional procedures (e.g., chest X-Ray) only if required per local guidelines/standard of care to rule out latent or active TB

  NOTE: A documented negative screening for TB via the PPD test or a negative QFT within 3 months prior to screening and if required per local standard of care, a chest X-ray, is sufficient and no further screening with QFT is required.

  Patients with a history of Bacille Calmette-Guérin (BCG) vaccination should be screened using the QFT test, only.
  - An indeterminate QFT test should be repeated.
  - A positive QFT test or two successive indeterminate QFT results should be considered a positive diagnostic TB test.
  - An indeterminate QFT test followed by a negative QFT test should be considered a negative diagnostic TB test.
- Only for patients currently receiving PPIs or H2RAs: Treatment must be at a stable dose during the 2-week screening period prior to randomization and with a plan to remain at a stable dose for the duration of the study.
- For women of childbearing potential: Agreement to remain abstinent (refrain from heterosexual intercourse) or use contraceptive methods that result in a failure rate of $<1\%$ per year during the treatment period and for at least 4 weeks after the last dose of study drug (see Section 5.4.3.1). Women must refrain from donating eggs during this same period.
  - A woman is considered to be of childbearing potential if she is postmenarcheal, has not reached a postmenopausal state (≥ 12 continuous months of amenorrhea with no identified cause other than menopause), and has not undergone surgical sterilization (removal of ovaries and/or uterus).
  - Examples of contraceptive methods with a failure rate of $<1\%$ per year include bilateral tubal ligation, male sterilization, established proper use of hormonal contraceptives that inhibit ovulation, hormone-releasing intrauterine devices, and copper intrauterine devices. Women using estrogen-containing hormonal contraceptives as a method of contraception must also use a barrier, such as a male condom, in conjunction with the hormonal contraceptives.

- The reliability of sexual abstinence should be evaluated in relation to the duration of the clinical trial and the preferred and usual lifestyle of the patient. Periodic abstinence (e.g., calendar, ovulation, symptothermal, or post-ovulation methods) and withdrawal are not acceptable methods of contraception.

- For men: agreement to remain abstinent (refrain from heterosexual intercourse) or use contraceptive measures and agreement to refrain from donating sperm, as defined below (also see Section 5.4.3.2):
    - With female partners of childbearing potential or pregnant female partners, men must remain abstinent or use a condom during the treatment period and for at least 4 weeks after the last dose of study treatment to avoid exposing the embryo. Men must refrain from donating sperm during this same period.
    - The reliability of sexual abstinence should be evaluated in relation to the duration of the clinical trial and the preferred and usual lifestyle of the patient. Periodic abstinence (e.g., calendar, ovulation, symptothermal, or post-ovulation methods) and withdrawal are not acceptable methods of contraception.

4.1.2 Exclusion Criteria

Patients in Cohort 1 and 2 who meet any of the following criteria will be excluded from study entry:

- Treatment with omalizumab or other monoclonal antibody therapies used to treat CSU within 4 months prior to screening or primary nonresponse to omalizumab
- Use of a non-biologic investigational drug or participation in an investigational study with a non-biologic drug within 30 days prior to study drug administration on Day 1 (or within 5 half-lives of the investigational product, whichever is greater)
- Use of a biologic investigational therapy or participation in an investigational study involving biologic therapy within 90 days or 5 half-lives, whichever is greater, prior to study drug administration on Day 1
- Previous treatment with fenebrutinib or other BTK inhibitors
- Patients whose urticaria is solely due to physical urticaria
- Other diseases with symptoms of urticaria or angioedema, including urticarial vasculitis, urticaria pigmentosa, erythema multiforme, mastocytosis, hereditary or acquired angioedema, lymphoma, or leukemia
- Atopic dermatitis, bullous pemphigoid, dermatitis herpetiformis, or other skin disease associated with itch such as psoriasis
- Routine (daily or every other day during 5 or more consecutive days) doses of the following medications within 30 days prior to screening: systemic or cutaneous (topical) corticosteroids (prescription or over the counter), hydroxychloroquine, methotrexate, cyclosporine, or cyclophosphamide
- Prior utilization of IV steroids for treatment of laryngeal angioedema
- IVIG or plasmapheresis within 30 days prior to screening
- History of anaphylactic shock without clearly identifiable avoidable antigen (e.g., due to food allergy)

- Hypersensitivity to fenebrutinib or any component of the formulation
- Major surgery within 8 weeks prior to screening or surgery planned prior to end of study (12 weeks after randomization)
- Require any prohibited concomitant medications (see Section 4.4.3)
- History of live attenuated vaccine within 6 weeks prior to randomization or requirement to receive these vaccinations at any time during study drug treatment
    - Seasonal influenza and H1N1 vaccination is permitted if the inactivated vaccine formulation is administered.
- Evidence of clinically significant cardiac, neurologic, psychiatric, pulmonary, renal, hepatic, endocrine (including uncontrolled diabetes mellitus), metabolic, or GI disease that, in the investigator's opinion, would compromise the safety of the patient, interfere with the interpretation of the study results or otherwise preclude patient participation
    - Any items that are cause for uncertainty must be reviewed with the Medical Monitor.
- Uncontrolled disease states, such as asthma, psoriasis, or inflammatory bowel disease, where flares are commonly treated with oral or parenteral corticosteroids
- History of vasculitis
- Current liver disease
- Any known active infection (with the exception of fungal nail infections or oral herpes)
- History of recurrent bacterial, viral, mycobacterial or fungal infections (defined as $>$2 similar episodes requiring anti-microbial treatment within the previous 12 months), with the exception of recurrent oral or genital herpes (herpes simplex virus 1/herpes simplex virus 2) or uncomplicated urinary tract infections in females.
- Any history of opportunistic infections that, in the investigator or Sponsor's judgment, would raise safety concerns regarding the patient's participation in the study
- Any major episode of infection requiring hospitalization or treatment with IV antimicrobials within 8 weeks prior to and during screening or treatment with oral antimicrobials within 2 weeks prior to and during screening
    - Antimicrobials include antifungal, antibacterial, and antiviral agents.
- History of or currently active primary or secondary immunodeficiency, including known history of HIV infection
- Evidence of chronic and/or active hepatitis B or C
    - Positive hepatitis B surface antigen (HBsAg) or hepatitis C serology (regardless of treatment status)
    - Positive hepatitis B core antibody (HBcAb)

- History of cancer, including hematologic malignancy and solid tumors, within 10 years before screening
    - Basal or squamous cell carcinoma of the skin that has been excised and is considered cured and in situ carcinoma of the cervix treated with apparent success by curative therapy > 1 year prior to screening are not exclusionary.
- Women who are pregnant, nursing (breastfeeding), or intending to become pregnant during the study or within 4 weeks after completion of the study
- For women of childbearing potential (including those who have had a tubal ligation): positive serum pregnancy test result at screening or on Day 1.
    - A serum pregnancy test is needed on Day 1 only if the urine pregnancy test is positive (see Section 4.1.1 for definition of “childbearing potential”).
- History of alcohol, drug (e.g., tetrahydrocannabinol [THC], marijuana), or chemical abuse within the 12 months prior to screening as determined by the investigator
- Need for systemic anti-coagulation with warfarin, other oral or injectable anti-coagulants, or anti-platelet agents other than NSAIDs, aspirin, and other salicylates
- History of non-gallstone–related pancreatitis or chronic pancreatitis
- History of hospitalizations or transfusion for a GI bleed
- History of cerebrovascular accident (CVA) within 10 years or any history of hemorrhagic CVA
- History of spontaneous intracranial hemorrhage or history of traumatic intracranial hemorrhage within 10 years
- Known bleeding diathesis
- Screening 12-lead ECG that demonstrates clinically relevant abnormalities that may affect patient safety or interpretation of study results, including
    - QT interval corrected using Fridericia’s formula (QTcF) > 440 ms demonstrated by at least two ECGs > 30 minutes apart
- History of ventricular dysrhythmias or risk factors for ventricular dysrhythmias such as long QT syndrome and other genetic risk factors (e.g., Brugada syndrome), structural heart disease (e.g., severe left ventricular systolic dysfunction, severe left ventricular hypertrophy), coronary heart disease (symptomatic or with ischemia demonstrated by diagnostic testing, prior coronary artery bypass grafting, or coronary lesions > 70% diameter stenosis that have not been or cannot be re-vascularized), clinically significant electrolyte abnormalities (e.g., hypokalemia, hypomagnesemia, hypocalcemia), or family history of sudden unexplained death or cardiac ion channel mutations (e.g., congenital long QT syndrome)
- Current treatment with medications that are well known to prolong the QT interval (see https://crediblemeds.org/index.php/login/dlcheck) at doses that have a clinically meaningful effect on QT, as determined by the investigator; the investigator may contact the Sponsor for confirmation if needed

- Current treatment with astemizole, terfenadine, and/or ebastine
- Any condition possibly affecting oral drug absorption (e.g., gastrectomy, clinically significant diabetic gastroenteropathy, or certain types of bariatric surgery such as gastric bypass)
  - Procedures such as gastric banding, that simply divide the stomach into separate chambers, are not exclusionary.
- Any uncontrolled clinically significant laboratory abnormality that would affect safety, interpretation of study data, or the patient's participation in the study
- Subjects who live in detention on court order or on regulatory action as per local and national law (see §40 subSection 1 sentence 3 no. 4 Arzneimittelgesetz; Medicinal Products Act).

The following exclusion criteria are based on screening laboratory tests. Laboratory tests may be repeated once during the screening period unless otherwise indicated (see Section 4.5.2.1):

- Creatinine $>$ 1.5 times the upper limit of normal (ULN; may be repeated if 1.5–2 $\times$ ULN)
- Creatinine clearance $<$ 70 mL/min (may be repeated if 60–69 mL/min) as estimated by the Cockcroft-Gault Equation
- ALT or AST $>$ 1.5 times ULN (may be repeated if 1.5–3 $\times$ ULN)
- Total bilirubin $>$ ULN (may be repeated if 1–3 $\times$ ULN)
- Hemoglobin $<$ 11 g/dL (may be repeated if 10–10.9 g/dL)
- ANC $<$ 1.5×10^9/L (may be repeated if 1.2–1.5×10^9/L)
- Platelet count $<$ 100×10^9/L (may be repeated if 80–100×10^9/L)
- IgG $<$ 500 mg/dL (should not be repeated)
- Abnormalities in hepatic synthetic function tests (e.g., PT, INR, PTT, albumin) judged by the investigator to be clinically significant

4.2 METHOD OF TREATMENT ASSIGNMENT AND BLINDING

4.2.1 Randomization and Blinding

This study is randomized, double-blinded, and placebo-controlled to minimize potential bias in treatment assignment, patient monitoring, and efficacy assessments. Random allocation will be conducted via an interactive voice or web-based response system (IxRS) and the Sponsor will provide the specifications of the randomization algorithm to the IxRS vendor. Patients in Cohort 1 will be randomly allocated to 200 mg PO BID fenebrutinib or placebo at an approximately 2:1 ratio using a stratified permuted blocks randomization scheme with stratification by country. Patients in Cohort 2 will be randomly allocated to each of the four treatment arms at an approximately 1:1:1:1 ratio, using a stratified permuted blocks randomization scheme with stratification by country.

Patients and study site personnel will be blinded to the individual treatment assignments throughout the study. Only standard and safety laboratory data results from the local

laboratory (such as CBC, chemistries, and pregnancy testing) will be available to sites. Results of other assessments performed after randomization that might unblind investigators to the treatment patients received will not be provided to sites or to the Sponsor's staff directly involved in study conduct [redacted]

Although PK samples must be collected from patients assigned to the comparator arm to maintain the blinding of treatment assignment, PK assay results for these patients are generally not needed for the safe conduct or proper interpretation of this trial. Sponsor personnel or a designee responsible for performing PK assays will be unblinded to patients' treatment assignments to identify appropriate PK samples to be analyzed. Samples from patients assigned to the placebo arm will not be analyzed except by request (e.g., to evaluate a possible error in dosing).

Patient and study site personnel will be blinded to treatment assignments throughout the study. During trial conduct, the Sponsor will monitor blinded clinical and safety data on safety and study conduct on an ongoing basis. For Cohort 1, if required for safety evaluations, Sponsor team personnel, but not the sites, not directly involved in the conduct of the study will have access to unblinded data. These Sponsor team personnel may include individuals with clinical and medical experience, biostatisticians, and individuals responsible for analyzing and interpreting the pharmacodynamics and pharmacokinetics of the study drug. For information on Cohort 2, see Section 3.1.1.

4.2.2 Unblinding

If unblinding is necessary for patient management (e.g., in the case of a serious adverse event for which patient management might be affected by knowledge of treatment assignment), the investigator will be able to break the treatment code by contacting the IxRS. Treatment codes should not be broken except in emergency situations. If the investigator wishes to know the identity of the study drug for any other reason, he or she should contact the Medical Monitor directly. The investigator should document and provide an explanation for any premature unblinding (e.g., accidental unblinding, unblinding due to a serious adverse event).

For regulatory reporting purposes, and if required by local health authorities, the Sponsor will break the treatment code for all serious, unexpected, suspected adverse reactions (see Section 5.7) that are considered by the investigator or Sponsor to be related to study drug.

4.3 STUDY TREATMENT

The investigational medicinal product (IMP) for this study is fenebrutinib.

4.3.1 Formulation, Packaging, and Handling

4.3.1.1 Fenebrutinib and Placebo

Fenebrutinib will be provided by the Sponsor as 50-mg dose strength tablets with corresponding matching placebo tablets, which will be indistinguishable in appearance. Study drug (fenebrutinib or placebo) will be dispensed at the Day 1 and 29 visits for both cohorts.

Tablets will be supplied in bottles (Cohort 1) and blister wallets (Cohort 2) for the treatment arm to which the patient is randomly allocated. Each bottle and blister wallet will be labeled per local regulatory requirements. Fenebrutinib and placebo tablets should be stored between 2°C and 8°C. Please refer to the pharmacy manual for detailed instructions on study drug storage and preparation.

For information on the formulation and handling of fenebrutinib, see the Fenebrutinib Investigator's Brochure.

4.3.1.2 Background Therapy: Standard-of-Care H1 Antihistamines for CSU

For information on the formulation, packaging, and administration of standard-of-care H1 antihistamines for CSU, see local prescribing information.

4.3.2 Dosage, Administration, and Compliance of Fenebrutinib and Placebo

4.3.2.1 Fenebrutinib and Placebo Dose and Administration

For Cohort 1, the fenebrutinib dose level is 200 mg (4 tablets) BID (total of 8 tablets each day) with matching placebo (see Section 3.1). For Cohort 2, the fenebrutinib dose levels are 50 mg QD, 150 mg QD, and 200 mg BID, with matching placebos (see Table 3).

Patients in both cohorts will take fenebrutinib/placebo on Day 1 and ending on the Day 56 visit. Although the Day 57 visit is the last day of the study treatment period, no study drug for Study GS39684 will be given on the Day 57 visit. *Patients will* return for the safety follow-up visit 4 weeks after the last dose of study drug.

For mandatory morning clinic visits (see Appendix 1 and Section 4.4.5), patients should be instructed that the morning dose of study drug will be taken in the clinic. On other clinic visit days, if the visit occurs in the morning, the patient should be instructed that the morning dose of study drug will be taken in the clinic.

Table 3 Fenebrutinib Dosing Regimen by Treatment Arm (Cohort 2)

	Number of Tablets	
Fenebrutinib Dose Arm	Fenebrutinib (a.m./p.m.)	Placebo (a.m./p.m.)
50 mg QD	1/0	3/4
150 mg QD	3/0	1/4
200 mg BID	4/4	0/0
Placebo	0/0	4/4

BID = twice daily; QD = once daily.

Fenebrutinib or placebo may be taken orally with or without food, except on certain days (see Appendix 1), when the morning dose of oral study drug will be administered at the morning (mandatory) clinic visit while fasting. The dates and times of the most recent prior meal, last dose of oral study drug (prior to clinic visit), and timing of oral study drug administration in clinic should be recorded at each clinic visit. Patients should be instructed that a missed dose should not be taken with the next scheduled dose.

In addition, any antacids (e.g., Pepto-Bismol®, Rolaids®) should be recorded as concomitant medications, including date and time of last administration. Administration of study drug should be staggered with antacid use (i.e., study drug should be taken 2 hours before or 2 hours after the antacid).

At the Day 1 and 29 visits, sufficient study medication tablets will be dispensed to complete dosing until the end of the study. When study medication is administered at the site, it will be administered under supervision of study personnel, and the amount of study medication dispensed must be recorded.

4.3.2.2 Fenebrutinib and Placebo Compliance

The following measures will be taken to assess patient compliance with study drug. For Cohort 1, patients will be directed to bring the study drug bottle to each visit after randomization. In addition, sites will be responsible for prepopulating the dates on the dosing label (affixed to the bottle) for when patients are scheduled to take study drug. The patients will record the times (a.m. or p.m.) that they take each dose in their eDiary. The number of tablets issued minus the number of tablets returned will be used to calculate the number of tablets taken and compliance.

For Cohort 2, sites will be responsible for prepopulating the dates on the blister wallets for when patients are scheduled to take study drug. The patients will record the times (a.m. or p.m.) that they take each dose in their eDiary. Patients will be instructed to return all blister wallets (used and unused) at each study visit for assessment of compliance and for medication disposal.

Compliance will be documented on the source record. Any overdose or incorrect administration of study drug should be noted on the Study Drug Administration electronic Case Report Form (eCRF). Adverse events associated with an overdose or incorrect administration of study drug should be recorded on the Adverse Event eCRF. If compliance is ≤ 80%, the investigator or designee is to counsel the patient and ensure steps are taken to improve compliance.

4.3.2.3 Background Therapy: Standard-of-Care H1 Antihistamines for CSU

The following H1 antihistamine medications are allowed:

- Cetirizine 10–40 mg QD
- Levocetirizine 5–20 mg QD
- Fexofenadine 180–720 mg QD
- Loratadine 10–40 mg QD
- Desloratadine 5–20 mg QD
- Rupatadine 10–40 mg QD
- Bilastine 20–80 mg QD

All patients will be allowed to take study-defined, second-generation, H1 antihistamine medications consistent with standard-of-care (i.e., up to 4 times the approved dose per local treatment guidelines) during the screening, treatment, and follow-up periods. Patients should remain on a stable H1 antihistamine regimen throughout the study period. Loratadine (10 mg) or cetirizine (10 mg) will be provided and used on an as-needed basis (maximum 1 per day) during screening, treatment, and follow-up periods). Therapies used for the treatment of CSU prior to enrollment will be collected as part of the patient's medical history.

Patients taking either LTRAs or H2 blockers for diseases other than CSU (e.g., asthma or gastroesophageal reflux disease, respectively) at screening will be permitted to continue their use at a stable dose during the study. These diseases must be recorded as part of the medical history collected during the screening period. Inhaled asthma controllers, including inhaled corticosteroids, are permitted during the study.

4.3.3 <u>Investigational Medicinal Product Accountability</u>

All IMPs required for completion of this study (fenebrutinib and placebo) will be provided by the Sponsor where required by local health authority regulations. The study site will acknowledge receipt of IMPs using the IxRS to confirm the shipment condition and content. Any damaged shipments will be replaced.

IMPs either will be disposed of at the study site according to the study site's institutional standard operating procedure or will be returned to the Sponsor with the appropriate documentation. The site's method of IMP destruction must be agreed to by the Sponsor.

The site must obtain written authorization from the Sponsor before any IMP is destroyed, and IMP destruction must be documented on the appropriate form.

Accurate records of all IMPs received at, dispensed from, returned to, and disposed of by the study site should be recorded on the Drug Inventory Log.

4.3.4 Post-Trial Access to Fenebrutinib

Currently, the Sponsor (Genentech, a member of the Roche Group) *does not have any plans to provide Genentech IMP fenebrutinib or any other study treatments to patients who have completed the study. The Sponsor may evaluate whether to continue providing fenebrutinib in accordance with* the Roche Global Policy on Continued Access to Investigational Medicinal Product, available at the following website:

http://www.roche.com/policy_continued_access_to_investigational_medicines.pdf

4.4 CONCOMITANT THERAPY AND ADDITIONAL RESTRICTIONS

Concomitant therapy includes any medication (e.g., prescription drugs, over-the-counter drugs, vaccines, herbal or homeopathic remedies, nutritional supplements) used by a patient from 14 days prior to initiation of study drug to the study completion/ discontinuation visit. All such medications (including standard-of-care H1 antihistamines for CSU) should be reported to the investigator and recorded on the Concomitant Medications eCRF.

4.4.1 Permitted Therapy

Patients who use oral contraceptives, hormone-replacement therapy, or other maintenance therapy should continue their use. All concomitant medications should be reported to the investigator and recorded on the appropriate eCRF. Patients will be encouraged to use the minimal dose required to control their symptoms.

For the purposes of this study, dietary supplements are defined as vitamins, minerals, purified food substances, and herbals with pharmaceutical properties.

Vitamins, minerals, and purified food substances are allowed in amounts not known to be associated with adverse effects (e.g., hypervitaminosis). Herbals with pharmaceutical properties are allowed only if there is acceptable evidence of no CYP3A inhibition or induction (refer to Section 4.4.3 for a list of prohibited concomitant medications, including herbal products). Otherwise, herbals with pharmaceutical properties must be discontinued for at least 4 weeks prior to the first dose of study medication, unless there are sufficient data available regarding the duration of an herbal medication's PK and PD effects to allow a shorter washout to be specified (e.g., 5 half-lives). Please direct any questions to the Medical Monitor.

4.4.2 Cautionary Therapy

4.4.2.1 Acid-Reducing Agents

Patients who use antacids (e.g., Pepto-Bismol®, Rolaids®) for symptomatic relief of heartburn should take fenebrutinib or matching placebo at least 2 hours before or 2 hours after antacid administration because gastric acid improves fenebrutinib absorption.

Patients may be treated with PPIs or H2RAs at up to the maximum recommended dose according to local labeling. The dose should remain stable for at least the 2 weeks before randomization and throughout the study.

At visits with scheduled PK assessments (see Appendix 1), any use of PPIs, H2RAs, and/or other antacids (e.g., Pepto-Bismol®, Rolaids®) should be recorded as concomitant medications, including the date and time of last administration.

4.4.2.2 Statins

Several lipid-lowering agents (statins) are metabolized by CYP3A (simvastatin, lovastatin) and/or transported by BCRP (rosuvastatin, atorvastatin), and thus may be affected by drug-drug interaction with fenebrutinib, therefore, dose adjustments of these medications should be considered (Kellick et al. 2014).

- Simvastatin: recommended maximum dose of 10 mg/day
- Lovastatin: recommended maximum dose of 20 mg/day
- Rosuvastatin: recommended maximum dose of 10 mg/day
- Atorvastatin: recommended maximum dose of 20 mg/day

The use of statins has been associated with myopathy, which can manifest as weakness, tenderness or muscle pain with elevations of creatine kinase (CK) above ten times the ULN. In severe cases, myopathy can cause rhabdomyolysis with or without acute kidney injury secondary to myoglobinuria, and rare fatalities due to rhabdomyolysis have occurred. The risk of myopathy is increased by elevated plasma levels of statins. Predisposing factors for myopathy include advanced age ($\geq$65 years), female gender, uncontrolled hypothyroidism, renal impairment, or the use of concomitant medications that increase the plasma levels of the statin.

4.4.2.3 CYP3A and BCRP-Mediated Drug Interactions

Preliminary data from a clinical drug-drug interaction study (Study GP39616) suggest that fenebrutinib can be classified as a mild inhibitor of CYP3A at clinically relevant doses. It is possible that fenebrutinib inhibition of CYP3A may alter the metabolism of CYP3A substrates and result in increased plasma concentrations of CYP3A substrates. Therefore, medications in the following categories (listed in detail in Appendix 5) should be used with caution in consultation with the Medical Monitor (or delegate) as necessary unless otherwise specified in Appendix 5:

- Sensitive CYP3A substrates

- CYP3A substrates with a narrow therapeutic window

The use of hormone-replacement therapy or hormonal contraceptives containing the CYP3A substrate ethinylestradiol (with the concomitant use of a barrier method) is permitted; however, these agents should be used with caution, and patients should be counseled regarding the potential risks and benefit of these medications per the local prescribing information. Ethinyl estradiol is metabolized by CYP3A so plasma concentrations of ethinyl estradiol are expected to increase in the presence of fenebrutinib (Zhang et al. 2007, Wang et al. 2004). Ethinyl estradiol is not a sensitive substrate of CYP3A (Drug Development and Drug Interactions: Table of Substrates, Inhibitors and Inducers, www.fda.gov). Therefore, the magnitude of increase in ethinyl estradiol plasma concentrations is expected to be less than the increase observed in midazolam concentrations (i.e., less than 2-fold) in Study GP39616, a drug-drug interaction study evaluating the effect of fenebrutinib on the PK of the sensitive CYP3A substrate midazolam. Minor increases in ethinyl estradiol concentrations are not generally associated with adverse events (e.g., Ortho Tri-Cyclen® USPI). Ethinyl estradiol efficacy is expected to be maintained, and ethinyl estradiol continues to be considered a reliable and effective form of contraception in combination with fenebrutinib.

Preliminary data from Study GP39616 also suggest that fenebrutinib can be classified as a moderately sensitive substrate of CYP3A at clinically relevant doses. There is a moderate potential for a drug-drug interaction with any medication that strongly inhibits or induces this enzyme. Therefore, medications in the following categories (listed in detail in Appendix 5) should be avoided for 7 days or 5 half-lives, whichever is longer, prior to the first dose of study drug and until the last dose of study drug. If use of one of these medications is necessary, the risks and benefits should be discussed with the Medical Monitor (or delegate) prior to concomitant administration with study drug:

- Strong CYP3A inhibitors
- Moderate or strong CYP3A inducers

Lastly, preliminary data from Study GP39616 suggest that fenebrutinib is a moderate inhibitor of the breast cancer resistance protein (BCRP) (also known as ABCG2) transporter protein at clinically relevant doses. There is a potential for increased plasma concentrations of drugs that are known to be substrates of the BCRP transporter. Plasma concentrations of the medications in the following category (listed in detail in Appendix 5) may increase; therefore, they should be used with caution in consultation with the Medical Monitor (or delegate) as necessary unless otherwise specified in Section 4.4.1.

The medications listed above and in Section 4.4.1 are not necessarily comprehensive. Thus, the investigator should consult the prescribing information for any concomitant medication as well as the Internet references provided below when determining whether a certain medication is metabolized by or strongly inhibits or induces CYP3A. The

investigator should contact the Medical Monitor if questions arise regarding medications not listed above.

https://www.fda.gov/Drugs/DevelopmentApprovalProcess/DevelopmentResources/DrugInteractionsLabeling/ucm093664.htm (Tables 3-1, 3-2, 3-3, and 5-1)

http://medicine.iupui.edu/clinpharm/ddis/table.aspx

4.4.3 Prohibited Therapy

Prior to the screening visit (Day −14 for both cohorts) as specified below, and during the study (not including the safety follow-up period if patient failed additional H1 antihistamine for worsened symptoms; see Section 3.1) the following medications and treatments will be restricted. Patients who receive these medications as therapy for CSU will be discontinued from the study treatment but will be followed for safety evaluation:

- Systemic or topical corticosteroids (prescription or over the counter), hydroxychloroquine, methotrexate, cyclosporine, or cyclophosphamide within 30 days prior to screening if used routinely (daily or every other day during 5 or more consecutive days) – The use of corticosteroids may be used for exacerbations
- Doxepin within 30 days prior to screening
- Omalizumab or other monoclonal antibody therapies used to treat CSU within 4 months prior to screening
- IVIG within 30 days prior to screening
- Plasmapheresis within 30 days prior to screening
- LTRAs within 1 day prior to screening
- Astemizole, terfenadine, and ebastine within 1 day prior to screening

4.4.3.1 Live or Attenuated Vaccinations

Immunization with a live or attenuated vaccine is prohibited within 6 weeks prior to study drug administration on Day 1 and for the duration of study participation, including the 4-week safety follow-up period after the administration of the last dose. See Section 5 for further details and precautions around vaccinations.

4.4.4 Prohibited Food

Use of the following foods is prohibited during the study and for at least 7 days prior to initiation of study treatment: furanocoumarin derivatives as found in grapefruit, Seville orange, pomegranate, or star fruit juice or products. Please refer to Appendix 5 for additional information.

4.4.5 Additional Restrictions

Patients in both cohorts should be fasting overnight for > 8 hours prior to the PK draw and/or fasting lipid panel on Days 1, 8, 29, 57, and 85 (see Appendix 1).

4.5 STUDY ASSESSMENTS

Please see Appendix 1 for the schedule of activities to be performed during the study.

4.5.1 Informed Consent Forms and Screening Log

Written informed consent for participation in the study must be obtained before performing any study-related procedures. Informed Consent Forms for enrolled patients and for patients who are not subsequently enrolled will be maintained at the study site.

All screening evaluations must be completed and reviewed to confirm that patients meet all eligibility criteria before enrollment. The investigator will maintain a screening log to record details of all patients screened and to confirm eligibility or record reasons for screening failure, as applicable.

4.5.2 Eligibility at Screening

At screening, patients who fail to meet any laboratory inclusion/exclusion criteria or other eligibility criteria may be retested or re-screened as per the instructions in Section 4.5.2.1 and Section 4.5.2.2, respectively.

4.5.2.1 Retesting: Laboratory Inclusion/Exclusion

If a patient does not meet certain laboratory inclusion/exclusion criteria at screening, the investigator may repeat the tests once within the screening period (see Section 4.1.2 for laboratory tests and levels that can be retested). If the patient meets the laboratory eligibility criteria on the second assessment, he or she will be permitted to enter the study. It will not be considered a retesting if blood samples have to be redrawn because of sample handling problems, breakage, sample integrity, or laboratory error.

4.5.2.2 Re-screening

Re-screening refers to repeating the entire screening process. Re-screening is required if a patient has not met eligibility criteria within the original screening visit. (Note: patients who have failed two laboratory testing attempts as described in Section 4.1.2 cannot be re-screened). Patients are allowed to be re-screened only once per cohort. Each patient must be re-consented before re-screening occurs. It will not be considered a re-screening if blood samples have to be redrawn because of sample handling problems, breakage, sample integrity, or laboratory error.

4.5.3 Medical History and Demographic Data

Comprehensive medical and surgical history, including a comprehensive review of the patient's CSU medical history, will be collected at the Day −14 visit for both cohorts. This review will include onset of symptoms, date of diagnosis, and therapies received for CSU. In addition, history of omalizumab (Xolair®) use and reason for discontinuation will be collected.

Concomitant medical usage will be collected at all visits, including unscheduled visits. Demographic data will include age, sex, and self-reported race/ethnicity.

4.5.4 Physical Examinations

A complete physical examination should be performed at the Day −14 visit for both cohorts and should include an evaluation of the head, eyes, ears, nose, and throat, and the cardiovascular, dermatological, musculoskeletal, respiratory, GI, genitourinary, and neurological systems. Any abnormality identified at baseline should be recorded on the General Medical History and Baseline Conditions eCRF.

Subsequent examinations may be limited to detect changes in symptoms of CSU as well as directed by patient complaints regarding adverse events. Changes from baseline abnormalities should be recorded in patient notes. New or worsened clinically significant abnormalities should be recorded as adverse events on the Adverse Event eCRF.

4.5.5 Vital Signs

Vital signs will include measurements of heart rate, systolic and diastolic blood pressure while the patient is in a seated position, and temperature. Vital signs will be assessed as outlined in the Schedule of Activities in Appendix 1 and during other unscheduled study visits when clinically indicated. The patients' height and weight will be measured once during the screening visit for both cohorts.

4.5.6 FricTest

For subjects who have a history of dermographism, a FricTest® will be performed at screening (Day −14), baseline (Day 1), and Days 57 and 85 for both cohorts. The FricTest® is a flat, plastic comb with four round-ended plastic pins, 3 mm in diameter and of different lengths. The FricTest® defines provocation thresholds and severity of dermographism (i.e., 4 pins inducing wheals defines severe dermographism).

4.5.7 Laboratory, Biomarker, and Other Biological Samples

Samples for the following laboratory tests will be sent to one or several central laboratories for analysis as per the Schedule of Activities in Appendix 1. Laboratory tests prior to randomization and dosing may be performed locally on Day 0, if central laboratory tests are not available due to sampling handling problems, breakage, or lab error.

- Hematology: hemoglobin, hematocrit, platelet count, RBC count, WBC count, percent and absolute differential counts (neutrophils, bands, lymphocytes, monocytes, eosinophils, basophils, other cells)
- Serum chemistry: sodium, potassium, chloride, bicarbonate, glucose, BUN, creatinine, calcium, phosphorus, magnesium, total and direct bilirubin, total protein, albumin, ALT, AST, LDH, alkaline phosphatase, creatine phosphokinase, CRP, lipase, and uric acid
- Urinalysis including dipstick (pH, specific gravity, glucose, protein, ketones, blood) and microscopic examination (sediment, RBCs, WBCs, casts, crystals, epithelial cells, bacteria)
- Coagulation: INR, activated PTT, PT, fibrinogen

- Fasting lipid panel
- Viral serology
- Hepatitis B: HBsAg, total HBcAb, and hepatitis B surface antibody
- Hepatitis C antibody
- [redacted]
- [redacted]
- The following samples will be sent to the Sponsor or a designee for analysis:
- [redacted]
- Plasma samples for PK analysis
- See the Schedule of Activities provided in Appendix 1 for specific timepoints.
- Samples for the following laboratory tests will be sent to the study site's local laboratory for analysis:
- Pregnancy test

  All women of childbearing potential (including those who have had a tubal ligation) will have a serum pregnancy test at screening. Urine pregnancy tests will be performed at specified subsequent visits. If a urine pregnancy test result is positive, it must be confirmed by a serum pregnancy test. Should a positive result be recorded at any time, the procedures detailed in Section 5.4.3 should be followed. If a local urine pregnancy test shows a positive result, then study drug will not be administered that day. Other study procedures should also be postponed and the result must be confirmed by a serum pregnancy test prior to proceeding.
- QFT or PPD (if QFT not available) and additional procedures (e.g., chest X-ray) to rule out latent or active TB per local guidelines

See the Schedule of Activities provided in Appendix 1 for specific timepoints.

[redacted]

[redacted]

[redacted]	[redacted]
[redacted]	[redacted]
[redacted]	[redacted]
[redacted]	[redacted]

[redacted]

For sampling procedures, storage conditions, and shipment instructions, see the laboratory manual.

Biological samples will be destroyed when the final clinical study report (CSR) has been completed, with the following exceptions:

- Plasma samples collected for PK analysis will be destroyed no later than 5 years after the final CSR has been completed.
- Blood, urine, and serum samples collected for biomarker analyses will be destroyed no later than 15 years after the final CSR has been completed.

When a patient withdraws from the study, samples collected prior to the date of withdrawal may still be analyzed, unless the patient specifically requests that the samples be destroyed or local laws require destruction of the samples. However, if samples have been tested prior to withdrawal, results from those tests will remain as part of the overall research data.

Data arising from sample analysis will be subject to the confidentiality standards described in Section 8.4.

4.5.8 Electrocardiograms

A single ECG recording, without artifacts, must be obtained at specified timepoints, as indicated in Appendix 1. The ECG intervals (e.g., PR, QRS, QT, QTcF, and RR) and heart rate from this ECG will be entered into the eCRF. Any morphologic waveform changes or other ECG abnormalities must be documented on the eCRF.

All ECG recordings must be performed using a standard high-quality, high-fidelity digital electrocardiograph machine equipped with computer-based interval measurements. Lead placement should be as consistent as possible. ECGs for each patient should be obtained from the same machine whenever possible. ECG recordings must be performed after the patient has been resting in a supine position for at least 10 minutes prior to beginning the ECG recording. All ECGs can be performed without specific restrictions (e.g., can be performed at any time of day, before or after dosing, fasting or fed) but are to be obtained prior to other procedures scheduled at that same time (e.g., vital sign measurements, blood draws). Body position should be consistently maintained for each ECG evaluation to prevent changes in heart rate. Circumstances

that may induce changes in heart rate, including environmental distractions (e.g., television, radio, conversation) should be avoided during the pre-ECG resting period and during ECG recording.

For safety monitoring purposes, the investigator must review, sign, and date all ECG tracings. Paper copies of ECG tracings will be kept as part of the patient's permanent study file at the site. If considered appropriate by the Sponsor, ECGs may be analyzed retrospectively at a central laboratory.

If at a particular post-dose timepoint the mean QTcF is > 500 ms or > 60 ms longer than the baseline value, another ECG must be recorded, ideally within the next 5 minutes, and ECG monitoring should continue until QTcF has stabilized on two successive ECGs. The Medical Monitor should be notified as soon as possible within 24 hours. Standard-of-care treatment may be instituted per the discretion of the investigator. If a PK sample is not scheduled for that timepoint, an unscheduled PK sample should be obtained. A decision on study drug discontinuation should be made, as described in Section 4.6.2. The investigator should also evaluate the patient for potential concurrent risk factors (e.g., electrolyte abnormalities, co-medications known to prolong the QT interval, severe bradycardia).

4.5.9 Patient-Reported Outcomes

Data from two patient reported outcomes (PRO) tools will be collected via questionnaires to document the treatment benefit of fenebrutinib: the Urticaria Patient Daily eDiary, [redacted] The eDiary and [redacted] translated into the local language as required, will be completed in their entirety at specified timepoints during the study.

[redacted]

Patients will use an electronic device to capture the Urticaria Patient Daily eDiary (see Appendix 3). The electronic device and/or instructions for completing the questionnaires electronically will be provided by the investigator staff. The data will be transmitted to a centralized database maintained by the electronic device vendor. The data will be available for access by appropriate study personnel.

[redacted]

4.5.9.1 Urticaria Patient Daily eDiary

The Urticaria Patient Daily eDiary includes the UAS, which will be used to calculate the UAS7. The eDiary comprises questions regarding largest hive size, sleep interference

score, activity interference question, rescue medication use, angioedema episodes, number of calls to doctor or nurse practitioner, and study medication compliance.

The eDiary is to be completed twice per day (a.m./p.m.) by the patient for the duration of the study. The eDiary will be given to the patient at the Day −14 visit for both cohorts.

4.5.9.2 Urticaria Activity Score

During the week prior to Day 1 (i.e., Week −1), UAS7 will be recorded twice daily for the purposes of enrollment eligibility for both cohorts. Subsequently, the UAS will be recorded twice daily using the Urticaria Patient Daily eDiary.

The UAS is a composite, eDiary-recorded score with numeric severity intensity ratings (0 = none to 3 = intense/severe) for a) the number of wheals (hives) and b) the intensity of the pruritus (itch) over the past 12 hours (twice daily; see Section 3.3.6). The daily UAS is calculated as the average of the morning and evening scores. The UAS7 will be calculated and is the weekly sum of the daily UAS, which is the composite score of the intensity of pruritus and the number of wheals. The maximum UAS7 value is 42; the intensity of the itch/pruritus and the number of wheals/hives are graded in Table 2.

[redacted]

[redacted]

[redacted]

[redacted]

[redacted]

[redacted]

[redacted]

[redacted]

[redacted]

[redacted]

[redacted]

[redacted]

4.6 PATIENT, TREATMENT, STUDY, AND SITE DISCONTINUATION

4.6.1 Patient Discontinuation

Patients have the right to voluntarily withdraw from the study at any time for any reason. In addition, the investigator must withdraw a patient from the study for the following, but not limited to, reasons:

- Patient withdrawal of consent at any time
- Any medical condition that the investigator or Sponsor determines may jeopardize the patient's safety if he or she continues in the study
- Investigator or Sponsor determines it is in the best interest of the patient

- The investigator may withdraw a patient from the study at any time due to patient non-compliance (e.g., drug compliance ≤ 80%, missed visits, missing Urticaria Patient Daily eDiary entries)

For patients who withdraw from the study, every effort should be made to complete an early termination visit including the assessments on the Schedule of Activities (see Appendix 1). The primary reason for withdrawal from the study should be documented on the appropriate eCRF. However, patients will not be followed for any reason after consent has been withdrawn. Patients who withdraw from the study will not be replaced.

If the patient discontinues the study prior to Day 85 visit, the patient will be asked to return to the clinic for a safety follow–up visit 4 weeks after the last dose of study drug. Patients who refuse to complete the safety follow–up period should undergo an early termination visit. Patients who discontinue during the safety follow-up period prior to completion of the 4-week safety follow-up will be asked to complete an early termination visit (see Appendix 1).

If a patient withdraws for reasons related to a serious adverse event, every attempt should be made to follow the patient until resolution of the event.

4.6.2 Study Treatment Discontinuation

Patients must discontinue study treatment if they experience any of the following:

- Pregnancy
- Malignancy
- Any serious infection or infection requiring treatment with an IV antimicrobial agent
- Any prohibited medication as defined in Section 4.4.3

Patients who discontinue study treatment prematurely (prior to Day 85 visit), including but not limited to the reasons listed above, will be asked to return to the clinic for a safety follow-up visit 4 weeks after the last dose of study drug. Patients who refuse to complete the safety follow up period should return to the clinic for an early termination visit.

The primary reason for study treatment discontinuation should be documented on the appropriate eCRF. Patients who discontinue study treatment prematurely will not be replaced.

4.6.3 Study and Site Discontinuation

The Sponsor has the right to terminate this study at any time. Reasons for terminating the study include, but are not limited to, the following:

- The Sponsor must terminate the study if the Sponsor believes that the incidence or severity of adverse events in this or other studies indicates a potential health hazard to patients.

- The Sponsor may terminate the study if patient enrollment or completion of the study unsatisfactory.

The Sponsor will notify the investigator if the Sponsor decides to discontinue the study.

The Sponsor has the right to close a site at any time. Reasons for closing a site may include, but are not limited to, the following:

- The Sponsor must close a site if corrective actions to improve site performance in the following areas do not yield significant improvement:
  - Excessively slow recruitment
  - Poor protocol adherence
  - Inaccurate or incomplete data recording
  - Non-compliance with the International Council on Harmonisation (ICH) guideline for Good Clinical Practice
- No study activity (i.e., all patients have completed the study and all obligations have been fulfilled).

5. ASSESSMENT OF SAFETY

5.1 SAFETY PLAN

The safety plan for patients in this study is based on nonclinical and clinical experience with fenebrutinib in completed and ongoing studies, as well as published literature, on other BTK inhibitors and BTK biology. The important potential safety risks for fenebrutinib are outlined below. Please refer to the Fenebrutinib Investigator's Brochure for a complete summary of safety information.

Several measures will be taken to ensure the safety of patients participating in this study. Eligibility criteria have been designed to exclude patients at higher risk for potential toxicities. Patients will undergo safety monitoring during the study, including monitoring of vital signs, physical examination, ECGs, and routine laboratory safety assessments (hematology, chemistry, and urinalysis) and assessment of the nature, frequency, and severity of adverse events. In addition, guidelines for managing potential adverse events, including criteria for treatment interruption or discontinuation, and enhanced safety reporting are provided below.

In addition, an unblinded IMC will monitor patient safety throughout the study for Cohort 2 (see Section 3.1.1).

5.1.1 Safety Plan for Potential Risks Associated with Fenebrutinib

5.1.1.1 Infections

Fenebrutinib is a reversible inhibitor of BTK, and the degree to which fenebrutinib antagonism of BTK signaling may suppress immune activity is unknown. On the basis of patients with XLA, a primary immunodeficiency caused by mutations in the BTK gene,

it is anticipated that inhibitors of BTK may raise the risk for certain bacterial infections (Lederman and Winkelstein 1985; Broides et al. 2006), enteroviral infections (Misbah et al. 1992; Ziegner et al. 2002), intestinal infections with giardia and *Campylobacter* species (Winkelstein et al. 2006; van den Bruele et al. 2010), or other opportunistic infections, which are cleared primarily by B-cell adaptive immune responses. This risk is likely independent of sex for patients exogenously administered fenebrutinib.

Effects on lymphocytes and immunoglobulins in rats and dogs were reversible and considered to be related to pharmacological activity involving BTK inhibition. See Section 1.2.2 for related primary nonclinical toxicity findings and the Fenebrutinib Investigator's Brochure for further details.

To date, no immune-challenge experiments (e.g., T-dependent antigen response test) have been conducted in animals. It is not known whether these effects on B cells and IgG concentrations in animals will translate to humans or whether such changes would have functional or deleterious impact on immune function.

Infections, including pneumonia and fatal influenza, have occurred in patients with B-cell malignancies treated with fenebrutinib. In studies with healthy subjects with single doses and with dosing for 14 days, self-limited Grade 1 events of nasopharyngitis were reported but did not lead to any change in study drug dosing. One healthy subject had asymptomatic bacteriuria, which resolved while study drug dosing continued. In blinded and open-label studies in autoimmune indications, serious, severe, and opportunistic infections have been reported, including one case of pulmonary tuberculosis as well as cases of pneumonia, bronchitis, cellulitis, enterobacter infection, herpes zoster, acute pyelonephritis, necrotizing soft tissue infection, colitis, periorbital cellulitis, and sinusitis. As a safety precaution, the study protocol contains exclusion criteria for infections and potential infection risk and guidelines for study drug management in the event of infection. Study subjects and patients should be monitored for fever and potential infectious complications, including opportunistic infections, and evaluated promptly.

Patients will be excluded from the study if they have a history of hospitalization due to an infection in the 8 weeks before screening, evidence of active or latent or inadequately treated infection with *Mycobacterium* TB, known active infection (current) or history of recurrent infection, or any known immunodeficiency including IgG < 500 mg/dL.

[redacted] All patients in the study should be monitored for fever and potential infectious complications, including opportunistic infections and TB, and should be evaluated promptly. Physicians or a health care provider should give patients advice to prevent potential transmission of and exposure to endemic infections according to local or Centers for Disease Control and Prevention guidelines. Patients should be advised to seek immediate medical attention if they develop signs and symptoms suggestive of an infection. All infections occurring during the study, including but not limited to respiratory infections, cutaneous

infections, urinary tract infections, systemic viral infections, and episodes of suspicious or febrile diarrhea, should be evaluated using serology or polymerase chain reaction, if available, and cultured, if feasible, and any identified organisms noted in the eCRF. Any serious infection, infection requiring IV antimicrobials, or any opportunistic infection is considered an adverse event of special interest and should be reported to the Sponsor as outlined in Section 5.4.2.

Guidelines for management of study treatment in the event that infections are observed in patients are provided in Section 5.1.2.

Please refer to the Fenebrutinib Investigator's Brochure for further details.

5.1.1.2 Vaccinations

The effect of fenebrutinib upon the efficacy of vaccinations is unknown. It is recommended that appropriate vaccinations per local guidelines be up to date before study participation. Patients will be excluded from study participation and will not be dosed with fenebrutinib if they have been vaccinated with live, attenuated vaccines (e.g., the intranasal live attenuated influenza vaccines, BCG, varicella) within 6 weeks before planned dosing. In addition, immunization with a live or attenuated vaccine is prohibited for the duration of study participation, including the 4-week safety follow-up period after the administration of the last dose.

In addition, current routine household contact with children or others who have been vaccinated with live vaccine components may pose a risk to the patient during study treatment with fenebrutinib. Some of these vaccines include varicella ("chickenpox") vaccine, oral polio vaccine, and the inhaled flu vaccine. Following vaccination with live component vaccines, the virus may be shed in bodily fluids, including stool, and there is a potential risk that the virus may be transmitted to the patient.

General guidelines for immunosuppressed patients suggest that exposure to vaccinated individuals should be avoided following vaccination with these vaccines for the stated time periods:

- Varicella or attenuated typhoid fever vaccination for 4 weeks following vaccination
- Oral polio vaccination for 6 weeks following vaccination
- Attenuated rotavirus vaccine for 10 days following vaccination
- FluMist® (inhaled flu vaccine) for 1 week following vaccination

Please refer to the Fenebrutinib Investigator's Brochure for further details.

5.1.1.3 Bleeding

No decrease in platelets, changes in coagulation parameters, or bleeding events were observed in nonclinical studies with fenebrutinib. Bleeding events, including non-serious NCI CTCAE v4.0 Grade 1 bruising and serious Grade ≥3 GI bleeding, have been reported in patients with hematological malignancies treated with fenebrutinib in Study

GO29089. The GI bleeding events have not been dose related, and the events occurred in patients who were taking concomitant NSAIDs and who had a history of gastroesophageal or peptic ulcer disease. The impact of BTK inhibition as a potential risk factor for bleeding is unknown. BTK is expressed in platelets and is involved in platelet function via GPVI/collagen receptor signaling and GP1b receptor signaling. Platelets from patients with XLA, a genetic deficiency of BTK, demonstrate decreased activation in response to submaximal collagen stimulation but normal response to thrombin; clinically, there is no reported bleeding propensity of patients with XLA.

Bruising or bleeding events related to fenebrutinib have not been reported in healthy subjects. Grade ≥ 2 bleeding events have been reported in blinded and open-label studies of fenebrutinib in autoimmune indications, including hematuria, purpura, hematoma, and uterine and vaginal bleeding.

It is unknown whether fenebrutinib will increase the risk of bleeding in patients, especially in those receiving anti-platelet or anticoagulant therapies. As a precautionary safety measure, patients will be excluded from study participation if they have a need for systemic anticoagulation with warfarin or other oral or injectable anticoagulants or anti-platelet agents (other than NSAIDs, aspirin, and other salicylates), any history of hospitalizations or transfusion for a GI bleed, any history of a hemorrhagic CVA, any history of spontaneous intracranial hemorrhage, traumatic intracranial hemorrhage within 10 years prior to the study, or a known bleeding diathesis. Patients should be advised to seek immediate medical attention if they develop signs and symptoms suggestive of clinically significant bleeding.

Bleeding events of moderate or greater severity are considered adverse events of special interest and should be reported to the Sponsor in an expedited manner as outlined in Section 5.4.2.

Guidelines for management of study treatment in the event that bleeding is observed in patients are provided in Section 5.1.2. Please refer to the Fenebrutinib Investigator's Brochure for further details.

5.1.1.4 Cytopenias

Cytopenias have been observed in patients with hematological malignancies who received fenebrutinib as well as in the ongoing, blinded Phase II studies and OLEs in autoimmune diseases. Cytopenias have included neutropenia, anemia, and thrombocytopenia; events have been monitorable and clinically manageable (see the Fenebrutinib Investigator's Brochure for further details).

Patients should be monitored regularly with hematology laboratory evaluations as outlined in the schedule of activities (see Appendix 1) and should receive appropriate supportive care as clinically indicated. Patients should be advised to seek immediate medical attention if they develop signs and symptoms suggestive of cytopenias

(e.g., persistent fever, bruising, bleeding, pallor). Cytopenias should be managed according to local clinical guidelines.

Guidelines for managing study treatment in the event that cytopenia is observed are provided in Section 5.1.2. Please refer to the Fenebrutinib Investigator's Brochure for further details.

5.1.1.5 Gastrointestinal Effects

Body weight gain and food consumption changes have been observed in animals, including nonsignificant increases in male Wistar-Han rats administered ≥2 mg/kg/day (4.3 μM•hr) for 6 months, and significant reductions in rats administered 100 mg/kg/day (1438 μM•hr) and dogs administered 25 mg/kg (180 μM•hr) for 4 weeks. These effects on body weight gain and food consumption were reversible following discontinuation of fenebrutinib dosing.

NCI CTCAE v4.0 Grade 1 diarrhea, nausea, and abdominal pain have been reported in patients with B-cell malignancies; however, the events have resolved and have not led to study drug discontinuation. Healthy subjects in the MAD Study GA29347 reported events of mild self-limited nausea. Across studies with immune indications receiving blinded or open-label treatment, approximately 5% of enrolled patients have reported nausea, vomiting, diarrhea, or other gastrointestinal symptoms.

Throughout the study, patients will be monitored for GI side effects.

Guidelines for management of study treatment in the event of GI side effects in patients are provided in Section 5.1.1.5. Please refer to the Fenebrutinib Investigator's Brochure for further details.

5.1.1.6 Hepatotoxicity

Evidence of hepatobiliary injury was observed in animals administered relatively high doses of fenebrutinib in repeat-dose toxicity studies. Dose-dependent increases in ALT, AST, and/or bilirubin have been observed in rats administered ≥6 mg/kg/day (≥17 μM•hr) and dogs administered ≥10 mg/kg/day (≥36 μM•hr), with corresponding microscopic changes in the liver of dogs administered 25 mg/kg/day (180 μM•hr). The hepatotoxicity findings in dogs were associated with moribundity in two high-dose animals. The NOAEL for these findings was considered to be 10 mg/kg (36 μM•hr) in dogs, the most sensitive species, given the absence of fenebrutinib–related hepatotoxicity at this dose when administered for 9 months. This exposure provides a 5-fold safety multiple above the study dose of 200 mg BID (projected $AUC_{0\text{-}24}$ of 6.7 μM•hr). The hepatotoxicity findings were fully reversible and considered monitorable by changes in plasma transaminases and bilirubin that occurred at doses lower than those producing histopathology findings (see the Fenebrutinib Investigator's Brochure for further details).

In clinical studies in autoimmune indications, including in patients with CSU, cases of Grade 3 (or severe) transaminase elevations, including cases that were considered

serious by the investigator have been reported. These transaminase elevations have occurred in both the randomized clinical studies, which remain blinded to the Sponsor in terms of treatment assignment, and the open-label extension. The transaminase levels returned to normal after discontinuation of the study treatment. There have been no observed AEs of liver enzyme elevation in clinical studies to date in healthy subjects or patients with hematological malignancies. To date, no cases of transaminase elevations have led to clinical jaundice or bilirubin $>2\times$ULN (Hy's Law). All transaminase elevations have been reversible when dosing of blinded study medication/placebo was withheld.

As a safety risk-mitigation measure, to be eligible for the study, AST and/or ALT levels should be no more than $1.5\times$ULN, and total bilirubin levels should be normal at screening. Safety monitoring for potential hepatotoxicity includes baseline and routine evaluations of AST/ALT and total bilirubin levels throughout the study as outlined in the schedule of activities (see Appendix 1).

Laboratory results of either an AST or ALT $>5\times$ULN or an AST or ALT $>3\times$ULN in combination with a total bilirubin $>2\times$ULN, of which at least 35% is direct bilirubin or there is clinical jaundice, are considered adverse events of special interest and should be reported to the Sponsor in an expedited manner as outlined in Section 5.4.2.

Guidelines for the management of study treatment in the event of hepatotoxicity in patients are provided in Section 5.1.2. Please refer to the Fenebrutinib Investigator's Brochure for further details.

5.1.1.7 Cardiovascular Effects

Fenebrutinib is considered to have a low potential to cause QT interval prolongation or to directly affect other cardiovascular parameters, at therapeutic exposures. A minimal increase in QTc (7 ms or 3%) interval was noted at 45 mg/kg in the single-dose cardiovascular safety pharmacology study in telemetry-instrumented dogs. Based on extrapolated/interpolated toxicokinetic data, the unbound C_{max} at 45 mg/kg (considered a NOAEL) and no-observed–effect level of 15 mg/kg were 23-fold and 7-fold higher, respectively, than the mean unbound C_{max} in humans at the 200-mg BID dose. There have been no fenebrutinib–related changes in ECG parameters in the 4-week or 9-month dog toxicity studies.

Analysis of ECG data from the SAD and MAD studies in healthy subjects did not demonstrate any significant increase in either QRS interval or QTcF intervals. However, cardiac safety will be evaluated in all patients at baseline and throughout this study, with routine monitoring of vital signs (including heart rate and blood pressure), routine safety ECGs, and collection of adverse events (see Section 5.1.1.7 and Section 5.2.1).

Management of patients with sustained QTcF prolongation (QTcF that is >500 ms or >60 ms longer than the baseline value) should include recording another ECG, ideally within the next 5 minutes, and ECG monitoring should continue until QTcF has stabilized

on two successive ECGs. The Medical Monitor should be notified as soon as possible within 24 hours. Standard-of-care treatment may be instituted per the discretion of the investigator. If a PK sample is not scheduled for that timepoint, an unscheduled PK sample should be obtained. A decision on study drug discontinuation should be made, as described in Section 4.6.2. The investigator should also evaluate the patient for potential concurrent risk factors (e.g., electrolyte abnormalities, co-medications known to prolong the QT interval, severe bradycardia).

Guidelines for management of study treatment in the event in the event of cardiovascular effects in patients are provided in Section 5.1.2. Please refer to the Fenebrutinib Investigator's Brochure for further details.

5.1.1.8 Vascular Inflammation

Vascular inflammation (vasculitis) was observed in dogs administered fenebrutinib at ≥ 10 mg/kg/day (≥ 56 μM • hr) in the 4-week toxicity study, and these changes were not completely reversed by the end of the 4-week recovery period. There was no consistent correlation with any clinical biomarkers. However, in the 9-month toxicity study in dogs, no fenebrutinib–related vascular inflammation was observed up to the highest dose of 10 mg/kg/day (36 μM • hr), which is considered to be the NOAEL (AUC) for the canine vascular inflammation findings. This exposure provides a 5-fold safety multiple above the study dose of 200 mg BID (projected AUC_{0-24} of 6.7 μM • hr). The translatability of these findings to humans is unknown; however, Beagle dogs are susceptible to spontaneous development of polyarteritis syndrome (Snyder et al. 1995) and may be more sensitive to any drug-induced effects. Further, there are several examples of approved therapies for which there is no correlation between the finding of vasculitis in dogs or rats at clinically relevant exposures and adverse outcomes in patients (FDA 2011). Guidelines for management of study treatment in the event of vasculitis in patients are provided in Section 5.1.2. Please refer to the Fenebrutinib Investigator's Brochure for further details.

5.1.1.9 Malignancy

The impact of BTK inhibition on the development of malignancies is not known; however, malignancies have been identified as a potential concern for immunomodulatory agents. Malignancies have been reported in patients with XLA, including lymphoreticular malignancies, gastric and colorectal adenocarcinoma, and squamous cell carcinoma of the lung.

Patients with a history of cancer, including hematologic malignancy and solid tumors, within 10 years before screening will be excluded from the study. Basal or squamous cell carcinoma of the skin that has been excised and is considered cured and in situ carcinoma of the cervix treated with apparent success by curative therapy more than 1 year prior to screening are not exclusionary.

All malignancies are adverse events of special interest and should be reported to the Sponsor in an expedited manner as outlined in Section 5.2.3.

Guidelines for management of study treatment in the event of malignancies in patients are provided in Section 5.1.2. Please refer to the Fenebrutinib Investigator's Brochure for further details.

5.1.2 Management of Patients Who Experience Specific Adverse Events

5.1.2.1 Management of Specific Adverse Events

Guidelines for management of specific adverse events are outlined in Table 5.

Table 5 Guidelines for Management of Patients Who Experience Specific Adverse Events

Event	Action to be Taken [a]
**Infection [b]**	
Serious infection, opportunistic infection, or any infection requiring treatment with an IV antimicrobial agent	Discontinue study treatment and report event as an adverse event of special interest.
Self-limited infections that require treatment	Withhold study treatment during antimicrobial therapy. Study treatment may resume after consultation with the Medical Monitor.
Bleeding	Bleeding events of moderate or greater severity are considered adverse events of special interest and should be reported to the Sponsor in an expedited manner.<br>For serious bleeding events or bleeding events requiring transfusion, radiologic endoscopic, or elective operative intervention, withhold study treatment and consult with the Medical Monitor.
**Gastrointestinal effects**	
Nausea, vomiting, and/or diarrhea	Manage according to site institutional guidelines.<br>Consider administration of study treatment with food as a possible mitigation strategy.
**Malignancy**	
Any malignancy	Discontinue study treatment, with the exception of non-serious local and resectable basal or squamous cell carcinoma of the skin. Report event as an adverse event of special interest to the Sponsor in an expedited manner.
**Hepatotoxicity**	
AST or ALT 3.0–5.0 × ULN	Withhold study treatment and consult with the Medical Monitor.
AST or ALT > 3 × ULN in combination with a total bilirubin > 2 × ULN, of which at least 35% is direct bilirubin, or clinical jaundice	Discontinue study treatment. Report event(s) as adverse event of special interest (Hy's law) to the Sponsor in an expedited manner.

Table 5 Guidelines for Management of Patients Who Experience Specific Adverse Events (cont.)

Event	Action to be Taken [a]
AST or ALT > 5 × ULN	Discontinue study treatment. Any elevation of an AST or ALT > 5 × ULN should be reported as an adverse event of special interest to the Sponsor in an expedited manner.
**Cardiovascular effects**	
Sustained (at least two ECG measurements > 30 minutes apart) QTcF that is > 500 ms or > 60 ms longer than the baseline value	Unless there is a clear alternative cause other than study drug, discontinue study treatment. [c]
Sustained absolute QTcF that is > 515 ms	Unless there is a clear alternative cause other than study drug, discontinue study treatment. [c]
An episode of torsades de pointes or a new ECG finding of clinical concern	Unless there is a clear alternative cause other than study drug, discontinue study treatment. [c]
**Vascular inflammation**	
Vasculitis	Discontinue study treatment and consult with the Medical Monitor.

IV = intravenous; QTcF = QT interval corrected using Fridericia's formula; ULN = upper limit of normal.

Note: "Study treatment" includes study drug (fenebrutinib or placebo).

[a] Any patient who discontinues study treatment should enter safety follow-up, if possible.

[b] Appropriate laboratory investigations, including but not limited to cultures, should be performed to establish the etiology of any serious infection.

[c] In rare circumstances, it may be acceptable to resume study drug, provided that any ECG abnormalities have resolved and that the patient is appropriately monitored. Clinical judgment should be applied.

5.1.2.2 Management of Increases in QT Interval

Study drug should be discontinued in patients who develop any of the following:

- Sustained (at least two ECG measurements > 30 minutes apart) QTcF that is > 500 ms or > 60 ms longer than the baseline value
- Sustained absolute QTcF that is > 515 ms
- An episode of torsades de pointes or a new ECG finding of clinical concern

Of note, if there is a new intraventricular conduction block, the increase in QRS complex duration should be subtracted from the QTcF change, because this represents an increase in QTcF unrelated to alterations in repolarization. Also of note, it is not uncommon to record arrhythmias, such as non-sustained ventricular tachycardia, supraventricular tachycardia, pauses, or atrial fibrillation, in healthy volunteers receiving placebo during periods of extended ECG monitoring. Therefore, it is critical that expert cardiology advice be sought to confirm any ECG changes and to ascertain the likelihood of a drug-induced arrhythmia versus the background occurrence of this arrhythmia. In such a situation, saving all available ECG data is highly suggested.

Management of patients with sustained QTcF prolongation should include close monitoring, with ECGs repeated at least hourly until two successive ECGs show resolution of the findings, correction of any electrolyte abnormalities, and possible discontinuation of other concomitant medications that are known to prolong the QT interval. Consultation with a cardiologist or electrophysiologist is recommended to help in the management of such patients.

5.2 SAFETY PARAMETERS AND DEFINITIONS

Safety assessments will consist of monitoring and recording adverse events, including serious adverse events and adverse events of special interest, performing protocol-specified safety laboratory assessments, measuring protocol-specified vital signs, and conducting other protocol-specified tests that are deemed critical to the safety evaluation of the study.

Certain types of events require immediate reporting to the Sponsor, as outlined in Section 5.4.

5.2.1 Adverse Events

According to the ICH guideline for Good Clinical Practice, an adverse event is any untoward medical occurrence in a clinical investigation subject administered a pharmaceutical product, regardless of causal attribution. An adverse event can therefore be any of the following:

- Any unfavorable and unintended sign (including an abnormal laboratory finding), symptom, or disease temporally associated with the use of a medicinal product, whether or not considered related to the medicinal product
- Any new disease or exacerbation of an existing disease (a worsening in the character, frequency, or severity of a known condition), except as described in Section 5.3.5.10
- Recurrence of an intermittent medical condition (e.g., headache) not present at baseline
- Any deterioration in a laboratory value or other clinical test (e.g., ECG, X-ray) that is associated with symptoms or leads to a change in study treatment or concomitant treatment or discontinuation from study drug
- Adverse events that are related to a protocol-mandated intervention, including those that occur prior to assignment of study treatment (e.g., screening invasive procedures such as biopsies)

5.2.2 Serious Adverse Events (Immediately Reportable to the Sponsor)

A serious adverse event is any adverse event that meets any of the following criteria:

- Is fatal (i.e., the adverse event actually causes or leads to death)

- Is life threatening (i.e., the adverse event, in the view of the investigator, places the patient at immediate risk of death)
    - This does not include any adverse event that had it occurred in a more severe form or was allowed to continue might have caused death.
- Requires or prolongs inpatient hospitalization (see Section 5.3.5.11)
- Results in persistent or significant disability/incapacity (i.e., the adverse event results in substantial disruption of the patient's ability to conduct normal life functions)
- Is a congenital anomaly/birth defect in a neonate/infant born to a mother exposed to study drug
- Is a significant medical event in the investigator's judgment (e.g., may jeopardize the patient or may require medical/surgical intervention to prevent one of the outcomes listed above)

The terms "severe" and "serious" are not synonymous. Severity refers to the intensity of an adverse event (e.g., rated as mild, moderate, or severe; see Section 5.3.3); the event itself may be of relatively minor medical significance (such as severe headache without any further findings).

Severity and seriousness need to be independently assessed for each adverse event recorded on the eCRF.

Serious adverse events are required to be reported by the investigator to the Sponsor immediately (i.e., no more than 24 hours after learning of the event; see Section 5.4.2 for reporting instructions).

5.2.3 Adverse Events of Special Interest (Immediately Reportable to the Sponsor)

Adverse events of special interest are required to be reported by the investigator to the Sponsor immediately (i.e., no more than 24 hours after learning of the event; see Section 5.4.2 for reporting instructions). Adverse events of special interest for this study include the following:

- Any serious infection, any infections requiring IV antimicrobials and any opportunistic infections
- Bleeding events of moderate or greater severity
- All malignancies
- Adverse events of special interest for general drug development
    - A laboratory result of AST or ALT $> 5 \times$ ULN
    - Cases of potential drug-induced liver injury that include an ALT or AST $> 3 \times$ ULN in combination with a total bilirubin $> 2 \times$ ULN, of which at least 35% is direct bilirubin or there is clinical jaundice, as defined by Hy's law (see Section 5.1.1.6)

- Suspected transmission of an infectious agent by the study drug, as defined below:

  Any organism, virus, or infectious particle (e.g., prion protein transmitting transmissible spongiform encephalopathy), pathogenic or non-pathogenic, is considered an infectious agent. A transmission of an infectious agent may be suspected from clinical symptoms or laboratory findings that indicate an infection in a patient exposed to a medicinal product. This term applies only when a contamination of the study drug is suspected.

5.3 METHODS AND TIMING FOR CAPTURING AND ASSESSING SAFETY PARAMETERS

The investigator is responsible for ensuring that all adverse events (see Section 5.2.1 for definition) are recorded on the Adverse Event eCRF and reported to the Sponsor in accordance with instructions provided in this Section and in Section 5.4 – Section 5.6.

For each adverse event recorded on the Adverse Event eCRF, the investigator will make an assessment of seriousness (see Section 5.2.2), severity (see Section 5.3.3), and causality (see Section 5.3.4).

5.3.1 Adverse Event Reporting Period

Investigators will seek information on adverse events at each patient contact. All adverse events, whether reported by the patient or noted by study personnel, will be recorded in the patient's medical record and on the Adverse Event eCRF.

After informed consent has been obtained **but prior to initiation of study drug**, only serious adverse events caused by a protocol-mandated intervention (e.g., invasive procedures such as biopsy sample collection, discontinuation of medications) should be reported (see Section 5.4.2 for instructions for reporting serious adverse events).

After initiation of study drug, all adverse events will be reported until 4 weeks after the last dose of study drug the patients receives. After this period, the investigator should report any serious adverse events that are believed to be related to prior study drug treatment (see Section 5.4.2).

5.3.2 Eliciting Adverse Event Information

A consistent methodology of non-directive questioning should be adopted for eliciting adverse event information at all patient evaluation timepoints. Examples of non-directive questions include the following:

"How have you felt since your last clinic visit?"

"Have you had any new or changed health problems since you were last here?"

5.3.3 Assessment of Severity of Adverse Events

Table 6 provides the adverse event grading scale for severity.

Table 6 Adverse Event Severity Grading Scale

Severity	Description
Mild	Discomfort noticed, but no disruption of normal daily activity
Moderate	Discomfort sufficient to reduce or affect normal daily activity
Severe	Incapacitating with inability to work or to perform normal daily activity

Note: Regardless of severity, some events may also meet seriousness criteria. Refer to definition of a serious adverse event (see Section 5.2.2).

5.3.4 Assessment of Causality of Adverse Events

Investigators should use their knowledge of the patient, the circumstances surrounding the event, and an evaluation of any potential alternative causes to determine whether an adverse event is considered to be related to the study drug, indicating "yes" or "no" accordingly. The following guidance should be taken into consideration (see also Table 7):

- Temporal relationship of event onset to the initiation of study drug
- Course of the event, with special consideration of the effects of dose reduction, discontinuation of study drug, or reintroduction of study drug (as applicable)
- Known association of the event with the study drug or with similar treatments
- Known association of the event with the disease under study
- Presence of risk factors in the patient or use of concomitant medications known to increase the occurrence of the event
- Presence of non-treatment–related factors that are known to be associated with the occurrence of the event

Table 7 Causal Attribution Guidance

Is the adverse event suspected to be caused by the study drug on the basis of facts, evidence, science-based rationales, and clinical judgment?	
YES	There is a plausible temporal relationship between the onset of the adverse event and administration of the study drug, and the adverse event cannot be readily explained by the patient's clinical state, intercurrent illness, or concomitant therapies; and/or the adverse event follows a known pattern of response to the study drug; and/or the adverse event abates or resolves upon discontinuation of the study drug or dose reduction and, if applicable, reappears upon re-challenge.
NO	An adverse event will be considered related, unless it fulfills the criteria specified below.<br>Evidence exists that the adverse event has an etiology other than the study drug (e.g., preexisting medical condition, underlying disease, intercurrent illness, or concomitant medication); and/or the adverse event has no plausible temporal relationship to administration of the study drug (e.g., cancer diagnosed 2 days after first dose of study drug).

For patients receiving combination therapy, causality will be assessed individually for each protocol-mandated therapy.

5.3.5 Procedures for Recording Adverse Events

Investigators should use correct medical terminology/concepts when recording adverse events on the Adverse Event eCRF. Avoid colloquialisms and abbreviations.

Only one adverse event term should be recorded in the event field on the Adverse Event eCRF.

5.3.5.1 Diagnosis versus Signs and Symptoms

A diagnosis (if known) should be recorded on the Adverse Event eCRF rather than individual signs and symptoms (e.g., record only liver failure or hepatitis rather than jaundice, asterixis, and elevated transaminases). However, if a constellation of signs and/or symptoms cannot be medically characterized as a single diagnosis or syndrome at the time of reporting, each individual event should be recorded on the Adverse Event eCRF. If a diagnosis is subsequently established, all previously reported adverse events based on signs and symptoms should be nullified and replaced by one adverse event report based on the single diagnosis, with a starting date that corresponds to the starting date of the first symptom of the eventual diagnosis.

5.3.5.2 Adverse Events That Are Secondary to Other Events

In general, adverse events that are secondary to other events (e.g., cascade events or clinical sequelae) should be identified by their primary cause, with the exception of severe or serious secondary events. A medically significant secondary adverse event that is separated in time from the initiating event should be recorded as an independent event on the Adverse Event eCRF. For example:

- If vomiting results in mild dehydration with no additional treatment in a healthy adult, only vomiting should be reported on the eCRF.
- If vomiting results in severe dehydration, both events should be reported separately on the eCRF.
- If a severe GI hemorrhage leads to renal failure, both events should be reported separately on the eCRF.
- If dizziness leads to a fall and consequent fracture, all three events should be reported separately on the eCRF.
- If neutropenia is accompanied by an infection, both events should be reported separately on the eCRF.

All adverse events should be recorded separately on the Adverse Event eCRF if it is unclear as to whether the events are associated.

5.3.5.3 Persistent or Recurrent Adverse Events

A persistent adverse event is one that extends continuously, without resolution, between patient evaluation timepoints. Such events should be recorded only once on the Adverse Event eCRF. The initial severity (intensity or grade) of the event will be recorded at the time the event is first reported. If a persistent adverse event becomes

more severe, the most extreme severity should also be recorded on the Adverse Event eCRF. If the event becomes serious, it should be reported to the Sponsor immediately (i.e., no more than 24 hours after learning that the event became serious; see Section 5.4.2 for reporting instructions). The Adverse Event eCRF should be updated by changing the event from "non-serious" to "serious," providing the date that the event became serious and completing all data fields related to serious adverse events.

A recurrent adverse event is one that resolves between patient evaluation timepoints and subsequently recurs. Each recurrence of an adverse event should be recorded as a separate event on the Adverse Event eCRF.

5.3.5.4 Abnormal Laboratory Values

Not every laboratory abnormality qualifies as an adverse event. A laboratory test result must be reported as an adverse event if it meets any of the following criteria:

- Is accompanied by clinical symptoms
- Results in a change in study treatment (e.g., dosage modification, treatment interruption, or treatment discontinuation)
- Results in a medical intervention (e.g., potassium supplementation for hypokalemia) or a change in concomitant therapy
- Is clinically significant in the investigator's judgment

It is the investigator's responsibility to review all laboratory findings. Medical and scientific judgment should be exercised in deciding whether an isolated laboratory abnormality should be classified as an adverse event.

If a clinically significant laboratory abnormality is a sign of a disease or syndrome (e.g., alkaline phosphatase and bilirubin 5 × ULN associated with cholestasis), only the diagnosis (i.e., cholestasis) should be recorded on the Adverse Event eCRF.

If a clinically significant laboratory abnormality is not a sign of a disease or syndrome, the abnormality itself should be recorded on the Adverse Event eCRF, along with a descriptor indicating whether the test result is above or below the normal range (e.g., "elevated potassium," as opposed to "abnormal potassium"). If the laboratory abnormality can be characterized by a precise clinical term per standard definitions, the clinical term should be recorded as the adverse event. For example, an elevated serum potassium level of 7.0 mEq/L should be recorded as "hyperkalemia."

Observations of the same clinically significant laboratory abnormality from visit to visit should only be recorded once on the Adverse Event eCRF (see Section 5.3.5.3 for details on recording persistent adverse events).

5.3.5.5 Abnormal Vital Sign Values

Not every vital sign abnormality qualifies as an adverse event. A vital sign result must be reported as an adverse event if it meets any of the following criteria:

- Is accompanied by clinical symptoms
- Results in a change in study treatment (e.g., dosage modification, treatment interruption, or treatment discontinuation)
- Results in a medical intervention or a change in concomitant therapy
- Is clinically significant in the investigator's judgment

It is the investigator's responsibility to review all vital sign findings. Medical and scientific judgment should be exercised in deciding whether an isolated vital sign abnormality should be classified as an adverse event.

If a clinically significant vital sign abnormality is a sign of a disease or syndrome (e.g., high blood pressure), only the diagnosis (i.e., hypertension) should be recorded on the Adverse Event eCRF.

Observations of the same clinically significant vital sign abnormality from visit to visit should only be recorded once on the Adverse Event eCRF (see Section 5.3.5.3 for details on recording persistent adverse events).

5.3.5.6 Abnormal Liver Function Tests

The finding of an elevated ALT or AST ($>3 \times$ baseline value) in combination with either an elevated total bilirubin ($>2 \times$ ULN) or clinical jaundice in the absence of cholestasis or other causes of hyperbilirubinemia is considered to be an indicator of severe liver injury (as defined by Hy's law). Therefore, investigators must report as an adverse event the occurrence of either of the following:

- Treatment-emergent ALT or AST $>3 \times$ baseline value in combination with total bilirubin $>2 \times$ ULN (of which $\geq$35% is direct bilirubin)
- Treatment-emergent ALT or AST $>3 \times$ baseline value in combination with clinical jaundice

The most appropriate diagnosis or (if a diagnosis cannot be established) the abnormal laboratory values should be recorded on the Adverse Event eCRF (see Section 5.3.5.2) and reported to the Sponsor immediately (i.e., no more than 24 hours after learning of the event), either as a serious adverse event or an adverse event of special interest (see Section 5.4.2).

5.3.5.7 Deaths

All deaths that occur during the protocol-specified adverse event reporting period (see Section 5.3.1), regardless of relationship to study drug, must be recorded on the Adverse Event eCRF and immediately reported to the Sponsor (see Section 5.4.2). This includes death attributed to progression of chronic spontaneous urticaria.

Death should be considered an outcome and not a distinct event. The event or condition that caused or contributed to the fatal outcome should be recorded as the single medical concept on the Adverse Event eCRF. Generally, only one such event should be reported. If the cause of death is unknown and cannot be ascertained at the time of reporting, **"unexplained death"** should be recorded on the Adverse Event eCRF. If the cause of death later becomes available (e.g., after autopsy), "unexplained death" should be replaced by the established cause of death. The term **"sudden death"** should not be used unless combined with the presumed cause of death (e.g., "sudden cardiac death").

If the death is attributed to angioedema of CSU, "chronic spontaneous urticaria angioedema" should be recorded on the Adverse Event eCRF.

Deaths that occur after the adverse event reporting period should be reported as described in Section 5.6.

5.3.5.8 Preexisting Medical Conditions

A preexisting medical condition is one that is present at the screening visit for this study. Such conditions should be recorded on the General Medical History and Baseline Conditions eCRF.

A preexisting medical condition should be recorded as an adverse event only if the frequency, severity, or character of the condition worsens during the study. When recording such events on the Adverse Event eCRF, it is important to convey the concept that the preexisting condition has changed by including applicable descriptors (e.g., "more frequent headaches").

5.3.5.9 Lack of Efficacy or Worsening of Chronic Spontaneous Urticaria

Medical occurrences or symptoms of deterioration that are anticipated as part of CSU should be recorded as an adverse event if judged by the investigator to have unexpectedly worsened in severity or frequency or changed in nature compared to baseline at any time during the study. When recording an unanticipated worsening of CSU on the Adverse Event eCRF, it is important to convey the concept that the condition has changed by including applicable descriptors (e.g., "accelerated CSU").

5.3.5.10 Hospitalization or Prolonged Hospitalization

Any adverse event that results in hospitalization (i.e., inpatient admission to a hospital) or prolonged hospitalization should be documented and reported as a serious adverse event (per the definition of serious adverse event in Section 5.2.2), except as outlined below.

An event that leads to hospitalization under the following circumstances should not be reported as an adverse event or a serious adverse event:

- Hospitalization for respite care

- Hospitalization for a preexisting condition, provided that all of the following criteria are met:
  - The hospitalization was planned prior to the study or was scheduled during the study when elective surgery became necessary because of the expected normal progression of the disease
  - The patient has not experienced an adverse event

An event that leads to hospitalization under the following circumstances is not considered to be a serious adverse event, but should be reported as an adverse event instead:

- Hospitalization that was necessary because of patient requirement for outpatient care outside of normal outpatient clinic operating hours. For this scenario, record the underlying medical condition which resulted in hospitalization on the Adverse Event eCRF.

5.3.5.11 Adverse Events Associated with an Overdose or Error in Drug Administration

An overdose is the accidental or intentional use of a drug in an amount higher than the dose being studied. An overdose or incorrect administration of study treatment is not itself an adverse event, but it may result in an adverse event. All adverse events associated with an overdose or incorrect administration of study drug should be recorded on the Adverse Event eCRF. If the associated adverse event fulfills seriousness criteria, the event should be reported to the Sponsor immediately (i.e., no more than 24 hours after learning of the event; see Section 5.4.2).

No safety data related to overdosing of GDC-0853 are available.

5.3.5.12 Patient-Reported Outcome Data

Adverse event reports will not be derived from PRO data by the Sponsor, and safety analyses will not be performed using PRO data. However, if any PRO responses suggestive of a possible adverse event are identified during site review of the PRO data, the investigator will determine whether the criteria for an adverse event have been met and, if so, will report the event on the Adverse Event eCRF.

5.4 IMMEDIATE REPORTING REQUIREMENTS FROM INVESTIGATOR TO SPONSOR

Certain events require immediate reporting to allow the Sponsor to take appropriate measures to address potential new risks in a clinical trial. The investigator must report such events to the Sponsor immediately; under no circumstances should reporting take place more than 24 hours after the investigator learns of the event. The following is a list of events that the investigator must report to the Sponsor within 24 hours after learning of the event, regardless of relationship to study drug:

- Serious adverse events (see Section 5.4.2 for further details)

- Adverse events of special interest (see Section 5.4.2 for further details)
- Pregnancies (see Section 5.4.3 for further details)

The investigator must report new significant follow-up information for these events to the Sponsor immediately (i.e., no more than 24 hours after becoming aware of the information). New significant information includes the following:

- New signs or symptoms or a change in the diagnosis
- Significant new diagnostic test results
- Change in causality based on new information
- Change in the event's outcome, including recovery
- Additional narrative information on the clinical course of the event

Investigators must also comply with local requirements for reporting serious adverse events to the local health authority and Institutional Review Board or Ethics Committee (IRB/EC).

5.4.1 Emergency Medical Contacts

Medical Monitor Contact Information

Medical Monitor contact information:

Medical Monitor: [redacted]

Telephone No.: [redacted]

5.4.2 Reporting Requirements for Serious Adverse Events and Adverse Events of Special Interest

5.4.2.1 Events That Occur prior to Study Drug Initiation

After informed consent has been obtained but prior to initiation of study drug, only serious adverse events caused by a protocol-mandated intervention should be reported. The Serious Adverse Event/Adverse Event of Special Interest Reporting Form provided to investigators should be completed and submitted to the Sponsor or its designee immediately (i.e., no more than 24 hours after learning of the event), either by faxing or by scanning and emailing the form using the fax number or email address provided to investigators.

5.4.2.2 Events That Occur after Study Drug Initiation

After initiation of study drug, serious adverse events and adverse events of special interest will be reported until 4 weeks after the last dose of study drug. Investigators should record all case details that can be gathered immediately (i.e., within 24 hours after learning of the event) on the Adverse Event eCRF and submit the report via the electronic data capture (EDC) system. A report will be generated and sent to Safety Risk Management by the EDC system.

In the event that the EDC system is unavailable, the Serious Adverse Event/Adverse Event of Special Interest Reporting Form provided to investigators should be completed and submitted to the Sponsor or its designee immediately (i.e., no more than 24 hours after learning of the event), either by faxing or by scanning and emailing the form using the fax number or email address provided to investigators. Once the EDC system is available, all information will need to be entered and submitted via the EDC system.

Instructions for reporting serious adverse events that occur > 4 weeks after the last dose of study treatment are provided in Section 5.6.

5.4.3 Reporting Requirements for Pregnancies

5.4.3.1 Pregnancies in Female Patients

Female patients of childbearing potential will be instructed to immediately inform the investigator if they become pregnant during the study or within 4 weeks after the last dose of study drug. A Clinical Trial Pregnancy Reporting Form should be completed and submitted to the Sponsor or its designee immediately (i.e., no more than 24 hours after learning of the pregnancy), either by faxing or by scanning and emailing the form using the fax number or email address provided to investigators. Pregnancy should not be recorded on the Adverse Event eCRF. The investigator should discontinue study drug and counsel the patient, discussing the risks of the pregnancy and the possible effects on the fetus. Monitoring of the patient should continue until conclusion of the pregnancy. Any serious adverse events associated with the pregnancy (e.g., an event in the fetus, an event in the mother during or after the pregnancy, or a congenital anomaly/birth defect in the child) should be reported on the Adverse Event eCRF. In addition, the investigator will submit a Clinical Trial Pregnancy Reporting Form when updated information on the course and outcome of the pregnancy becomes available.

5.4.3.2 Pregnancies in Female Partners of Male Patients

Male patients will be instructed through the Informed Consent Form to immediately inform the investigator if their partner becomes pregnant during the study or within 4 weeks after the last dose of study drug. A Clinical Trial Pregnancy Reporting Form should be completed and submitted to the Sponsor or its designee immediately (i.e., no more than 24 hours after learning of the pregnancy), either by faxing or by scanning and emailing the form using the fax number or email address provided to investigators. Attempts should be made to collect and report details of the course and outcome of any pregnancy in the partner of a male patient exposed to study drug. When permitted by the site, the pregnant partner would need to sign an Authorization for Use and Disclosure of Pregnancy Health Information to allow for follow-up on her pregnancy. If the authorization has been signed, the investigator should submit a Clinical Trial Pregnancy Reporting Form when updated information on the course and outcome of the pregnancy becomes available. An investigator who is contacted by the male patient or his pregnant partner may provide information on the risks of the pregnancy and the possible effects on the fetus, to support an informed decision in cooperation with the treating physician and/or obstetrician.

5.4.3.3 Congenital Anomalies/Birth Defects and Abortions

Any congenital anomaly/birth defect in a child born to a female patient exposed to study drug or the female partner of a male patient exposed to study drug should be classified as a serious adverse event, recorded on the Adverse Event eCRF, and reported to the Sponsor immediately (i.e., no more than 24 hours after learning of the event; see Section 5.4.2). Any abortion should be reported in the same fashion (as the Sponsor considers abortions to be medically significant).

5.5 FOLLOW-UP OF PATIENTS AFTER ADVERSE EVENTS

5.5.1 <u>Investigator Follow-Up</u>

The investigator should follow each adverse event until the event has resolved to baseline grade or better, the event is assessed as stable by the investigator, the patient is lost to follow-up, or the patient withdraws consent. Every effort should be made to follow all serious adverse events considered to be related to study drug or trial-related procedures until a final outcome can be reported.

During the study period, resolution of adverse events (with dates) should be documented on the Adverse Event eCRF and in the patient's medical record to facilitate source data verification.

All pregnancies reported during the study should be followed until pregnancy outcome.

5.5.2 <u>Sponsor Follow-Up</u>

For serious adverse events, adverse events of special interest, and pregnancies, the Sponsor or a designee may follow up by telephone, fax, email, and/or a monitoring visit to obtain additional case details and outcome information (e.g., from hospital discharge summaries, consultant reports, autopsy reports) in order to perform an independent medical assessment of the reported case.

5.6 ADVERSE EVENTS THAT OCCUR AFTER THE ADVERSE EVENT REPORTING PERIOD

The Sponsor should be notified if the investigator becomes aware of any serious adverse event that occurs after the end of the adverse event reporting period (defined as 4 weeks after the last dose of study drug; see Section 5.3.1), if the event is believed to be related to prior study drug treatment. These events should be reported through use of the Adverse Event eCRF. However, if the EDC system is not available, the investigator should report these events directly to the Sponsor or its designee, either by faxing or by scanning and emailing the Serious Adverse Event/Adverse Event of Special Interest Reporting Form using the fax number or email address provided to investigators.

5.7 EXPEDITED REPORTING TO HEALTH AUTHORITIES, INVESTIGATORS, INSTITUTIONAL REVIEW BOARDS, AND ETHICS COMMITTEES

The Sponsor will promptly evaluate all serious adverse events and adverse events of special interest against cumulative product experience to identify and expeditiously communicate possible new safety findings to investigators, IRBs, ECs, and applicable health authorities based on applicable legislation.

To determine reporting requirements for single adverse event cases, the Sponsor will assess the expectedness of these events using the following reference document:

- Fenebrutinib Investigator's Brochure

The Sponsor will compare the severity of each event and the cumulative event frequency reported for the study with the severity and frequency reported in the applicable reference document.

Reporting requirements will also be based on the investigator's assessment of causality and seriousness, with allowance for upgrading by the Sponsor as needed.

6. STATISTICAL CONSIDERATIONS AND ANALYSIS PLAN

The primary and secondary efficacy analyses will be based on a modified intent-to-treat (mITT) approach. All patients who received at least one dose of study drug will be included in the mITT population, with patients grouped according to the treatment assigned at randomization. Safety analyses will be conducted on the safety-evaluable population, defined as all patients who received at least one dose of study drug, with patients grouped according to the actual treatment received.

For each cohort, the final analysis of data from the 8-week, placebo-controlled period will be performed when the following two criteria have been met: 1) All patients in the cohort have either completed the Day 57 visit or discontinued from the placebo-controlled period prematurely and 2) all data from the placebo-controlled period in the cohort are in the database and have been cleaned and verified. Patients and study site personnel will remain blinded to the individual treatment assignment until after the study is completed (i.e., after all patients in both cohorts have either completed the safety follow-up period or discontinued early from the study), the database is locked, and the study analyses are final for both cohorts.

The focus of the trial is estimation and generation of hypotheses to be confirmed in future trials; therefore, Type I error control is not addressed.

6.1 DETERMINATION OF SAMPLE SIZE

6.1.1 Cohort 1: Pilot Assessment

The purpose of this cohort is to evaluate the efficacy of fenebrutinib at 200 mg PO BID compared with placebo in improving the UAS7. Point and interval estimates of the

change from baseline of the UAS7 within each treatment group as well as of the difference in change from baseline of the UAS7 between treatment groups will be presented.

The cohort will enroll approximately 45 patients. Patients will be randomized in a 2:1 ratio to receive treatment with either fenebrutinib or placebo. The sample size of approximately 30 patients in the fenebrutinib arm and 15 patients in the placebo arm provides approximately 80% power to detect an 11-point difference in the UAS7 change from baseline at Day 57 between treatment groups under the following assumptions:

- The absolute change from baseline at Day 57 is normally distributed with a standard deviation of 13.
- Two-sided alpha is 0.10.
- Dropout rate at Day 57 is 10%, leading to a 10% loss of information.

6.1.2 Cohort 2: Dose-Ranging Assessment

The purpose of this cohort is estimation and hypothesis generation regarding the dose-ranging effects of fenebrutinib compared with placebo in improving the UAS7. Point and interval estimates of the change from baseline of the UAS7 within each treatment group as well as of the difference in change from baseline of the UAS7 between treatment groups vs placebo will be presented.

The cohort will enroll approximately 120 patients. Patients will be randomly allocated in a 1:1:1:1 ratio to receive treatment with one of three dose levels of fenebrutinib or placebo. The sample size of approximately 30 in each arm provides approximately 90% power to detect an 11-point difference in the UAS7 change from baseline at Day 57 between treatment groups, under the following assumptions:

- The absolute change from baseline at Day 57 is normally distributed with a standard deviation of 13
- Two-sided alpha is 0.10
- Dropout rate at Day 57 is 10%, leading to a 10% loss of information.

The overall sample size may be adjusted depending on the outcome of a planned interim analysis for Cohort 1 (see Section 6.8), which will include an evaluation of these assumptions.

6.2 SUMMARIES OF CONDUCT OF STUDY

The number of patients who enroll, discontinue, or complete the study will be summarized. Reasons for premature study withdrawal will be listed and summarized. Enrollment and major protocol deviations will be listed and evaluated for their potential effects on the interpretation of study results.

6.3 SUMMARIES OF TREATMENT GROUP COMPARABILITY

Baseline demographics, disease characteristics, and exposure to study drug will be summarized by treatment group using descriptive statistics. For categorical endpoints, the descriptive statistics will include counts and proportions. For continuous endpoints, the descriptive statistics will include the number of observations, mean, standard deviation, median, minimum, and maximum.

6.4 EFFICACY ANALYSES

Statistical analysis will be conducted for each cohort separately. Statistical testing will be performed as a two-sided test with a statistical significance level of 0.10. No multiplicity adjustments will be performed to control overall Type I error, and positive tests will be viewed as hypothesis generating rather than confirmatory.

Continuous longitudinal efficacy endpoints will be analyzed using a mixed model for repeated measures (MMRM) and descriptive statistics as appropriate. An unstructured covariance pattern will be specified to model the within-subject errors. Parameters will be estimated with the use of restricted maximum likelihood, and the Kenward-Roger method will be used for calculating the denominator degrees of freedom. The MMRM method assumes that data are missing at random. That is, MMRM assumes that given the statistical model and given the observed values of the endpoint, missing data are independent of the unobserved values (O'Kelly and Ratitch 2014). High correlation between successive observations on a subject allows data from subjects who dropped out to make a contribution to estimation of the effects at the final timepoint.

All MMRM models will include country, treatment group, visit, and visit by treatment group interaction as covariates.

Time-to-event endpoints will be analyzed using a Cox proportional hazards model. Categorical endpoints will be analyzed using an appropriate statistical method, such as Cochran-Mantel-Haenszel test or Fisher's exact test.

Details of all statistical methods will be provided in the Data Analysis Plans (DAP).

6.4.1 Primary Efficacy Endpoint

The primary efficacy endpoint is the change from baseline in the UAS7 at Day 57 (Week 8).

The UAS is to be recorded twice daily (i.e., morning and evening) using an eDiary that will be provided to each patient. Scores ranging from 0 (none) to 3 (severe) will be entered for each of the two UAS domains consisting of number of wheals (hives) and intensity of pruritus (itch) resulting in a total possible score of 0 to 6 (see Table 2). The daily UAS is calculated as the average of the morning and evening scores. When either the morning or evening score is missing, the non-missing UAS for that day (morning or evening) will be used as the daily UAS, and when both the morning and evening UAS

are missing, the daily UAS will be deemed missing. The UAS7 is the sum of the daily UAS over the 7 days prior to the timepoint of interest. The baseline UAS7 will be calculated as the sum of daily UAS values over the week (7 days) prior to Day 1.

When one or more daily UAS values is missing, over the week prior to a timepoint of interest, rules for deriving UAS7 will be as follows:

- If a patient has at least 4 completed daily scores on the UAS (both domains) over the 7 days prior to the timepoint of interest, the UAS7 will be defined as the average of the available daily scores, multiplied by 7.
- If a patient has fewer than 4 completed daily scores on the UAS over the 7 days prior to the timepoint of interest, then the UAS7 will be considered missing for that timepoint.

The primary endpoint will be analyzed using a MMRM model as specified in Section 6.4. Additional model covariates will include baseline UAS7 and its interaction with visit. Missing data will be handled by the model under the missing-at-random assumption without need for imputation.

As a sensitivity analysis, an analysis-of-covariance (ANCOVA) model adjusted for country and baseline UAS7 will be fit. Missing Day 57 data will be imputed by last observation carried forward.

6.4.2 Secondary Efficacy Endpoints

The secondary efficacy endpoints are as follows:

- Proportion of patients who are well controlled (UAS7 ≤6) at Day 57
- Change from baseline in the UAS7 at Day 29 (Week 4)

These endpoints will be analyzed as specified in Section 6.4.

6.4.3 Exploratory Efficacy Endpoints

The exploratory efficacy endpoints include the following:

- Change from baseline in the weekly itch score at Day 29
- Change from baseline in the weekly itch score at Day 57
- Change from baseline in the weekly hives score at Day 57
- Proportion of patients who are well controlled (UAS7 ≤6) at Day 29
- Proportion of patients who achieve complete response (UAS7 =0) at Day 29
- Proportion of patients who achieve complete response (UAS7 =0) at Day 57
- Proportion of patients achieving MID in UAS7 at Day 57 (reduction from baseline ≥11 points)
- Proportion of patients achieving MID in the weekly itch score at Day 57 (reduction from baseline ≥5 points)

- Time to achieving MID in UAS7 (reduction from baseline ≥ 11 points)
- [redacted]

Further details on the analysis of exploratory endpoints will be included in the DAP.

6.5 SAFETY ANALYSES

Adverse events will be graded according to the adverse event severity grading scale described in Section 5.3.3. Summaries of adverse events, serious adverse events, deaths, adverse events of special interest, adverse events that lead to discontinuation, ECG findings, laboratory test results, and vital sign measurements will be presented.

6.6 PHARMACOKINETIC ANALYSES

The PK endpoints are as follows:

- Plasma concentration data for fenebrutinib will be tabulated and summarized by visits. Descriptive summary statistics will include the arithmetic mean, median, range, SD, and coefficient of variation, as appropriate

[redacted]

The PK analyses will include patients with sufficient data to enable estimation of key parameters (e.g., AUC, t_{max}, C_{max}, $t_{1/2}$), with patients grouped according to treatment received. [redacted]

Additional PK analyses will be conducted as appropriate.

[redacted]

[redacted]

6.8 INTERIM ANALYSIS

6.8.1 Cohort 1: Planned Interim Analysis

An interim analysis will be performed after approximately 33 patients have completed their 8–week treatment period. The purpose of this analysis is to assess the efficacy of the 200-mg fenebrutinib BID daily arm compared with the placebo, to guide internal decision-making around issues such as ungating of Cohort 2, adequacy of sample sizes for safety and/or efficacy analyses in Cohort 2, or to inform further development decisions. Summaries of safety and efficacy data by treatment groups will be prepared and reviewed by Sponsor personnel who do not have direct contact with investigational staff, monitors, and patients. Further details of the interim analysis will be specified in the DAP prior to the conduct of the interim analysis. Access to treatment assignment information will follow the Sponsor's standard procedures.

6.8.2 Cohort 2: Optional Interim Analysis

Given the hypothesis-generating nature of this study, the Sponsor may choose to conduct up to two interim efficacy analyses. The decision to conduct an optional interim analysis and the timing of the analysis will be documented in the Sponsor's trial master file prior to the conduct of the interim analysis. The interim analysis will be performed and interpreted by members of the IMC. Access to treatment assignment information will follow the Sponsor's standard procedures.

7. DATA COLLECTION AND MANAGEMENT

7.1 DATA QUALITY ASSURANCE

The Sponsor will be responsible for data management of this study, including quality checking of the data. Data entered manually will be collected via EDC through use of eCRFs. Sites will be responsible for data entry into the EDC system. In the event of discrepant data, the Sponsor will request data clarification from the sites, which the sites will resolve electronically in the EDC system.

The Sponsor will produce an EDC Study Specification document that describes the quality checking to be performed on the data. Central laboratory data will be sent directly to the Sponsor, using the Sponsor's standard procedures to handle and process the electronic transfer of these data.

eCRFs and correction documentation will be maintained in the EDC system's audit trail. System backups for data stored by the Sponsor and records retention for the study data will be consistent with the Sponsor's standard procedures.

PRO data will be collected through the use of an electronic device provided by a vendor. The device is designed for entry of data in a way that is attributable, secure, and accurate, in compliance with U.S. Food and Drug Administration (FDA) regulations for electronic records (21 CFR Part 11). The electronic data are available for view access only via secure access to a web server method. Only identified and trained users may view the data, and their actions become part of the audit trail. The Sponsor will have view access only. System backups for data stored by the Sponsor and records retention for the study data will be consistent with the Sponsor's standard procedures.

7.2 ELECTRONIC CASE REPORT FORMS

eCRFs are to be completed through use of a Sponsor-designated EDC system. Sites will receive training and have access to a manual for appropriate eCRF completion. eCRFs will be submitted electronically to the Sponsor and should be handled in accordance with instructions from the Sponsor.

All eCRFs should be completed by designated, trained site staff. eCRFs should be reviewed and electronically signed and dated by the investigator or a designee.

At the end of the study, the investigator will receive patient data for his or her site in a readable format on a compact disc that must be kept with the study records. Acknowledgement of receipt of the compact disc is required.

7.3 ELECTRONIC PATIENT-REPORTED OUTCOME DATA

Patients will use an electronic device to capture PRO data. The data will be transmitted via wireless or web automatically after entry or uploaded by site staff at the appropriate frequency to a centralized database maintained by the electronic device vendor.

Once the study is complete, the data, audit trail, and trial and system documentation will be archived. The investigator will receive patient data for the site in both human- and machine-readable formats on an archival-quality compact disc that must be kept with the study records as source data. Acknowledgement of receipt of the compact disc is required. In addition, the Sponsor will receive all data in a machine-readable format on a compact disc.

7.4 SOURCE DATA DOCUMENTATION

Study monitors will perform ongoing source data verification to confirm that critical protocol data (i.e., source data) entered into the eCRFs by authorized site personnel are accurate, complete, and verifiable from source documents.

Source documents (paper or electronic) are those in which patient data are recorded and documented for the first time. They include, but are not limited to, hospital records, clinical and office charts, laboratory notes, memoranda, patient-reported outcomes, evaluation checklists, pharmacy dispensing records, recorded data from automated instruments, copies of transcriptions that are certified after verification as being accurate

and complete, microfiche, photographic negatives, microfilm or magnetic media, X-rays, patient files, and records kept at pharmacies, laboratories, and medico-technical departments involved in a clinical trial.

Before study initiation, the types of source documents that are to be generated will be clearly defined in the Trial Monitoring Plan. This includes any protocol data to be entered directly into the eCRFs (i.e., no prior written or electronic record of the data) and considered source data.

Source documents that are required to verify the validity and completeness of data entered into the eCRFs must not be obliterated or destroyed and must be retained per the policy for retention of records described in Section 7.6.

To facilitate source data verification, the investigators and institutions must provide the Sponsor direct access to applicable source documents and reports for trial-related monitoring, Sponsor audits, and IRB/EC review. The study site must also allow inspection by applicable health authorities.

7.5 USE OF COMPUTERIZED SYSTEMS

When clinical observations are entered directly into a study site's computerized medical record system (i.e., in lieu of original hardcopy records), the electronic record can serve as the source document if the system has been validated in accordance with health authority requirements pertaining to computerized systems used in clinical research. An acceptable computerized data collection system allows preservation of the original entry of data. If original data are modified, the system should maintain a viewable audit trail that shows the original data as well as the reason for the change, name of the person making the change, and date of the change.

7.6 RETENTION OF RECORDS

Records and documents pertaining to the conduct of this study and the distribution of IMP, including eCRFs, electronic PRO data (if applicable), Informed Consent Forms, laboratory test results, and medication inventory records, must be retained by the Principal Investigator for at least 15 years after completion or discontinuation of the study or for the length of time required by relevant national or local health authorities, whichever is longer. After that period of time, the documents may be destroyed, subject to local regulations.

No records may be disposed of without the written approval of the Sponsor. Written notification should be provided to the Sponsor prior to transferring any records to another party or moving them to another location.

Roche will retain study data for 25 years after the final Clinical Study Report has been completed or for the length of time required by relevant national or local health authorities, whichever is longer.

8. ETHICAL CONSIDERATIONS

8.1 COMPLIANCE WITH LAWS AND REGULATIONS

This study will be conducted in full conformance with the ICH E6 guideline for Good Clinical Practice and the principles of the Declaration of Helsinki, or the laws and regulations of the country in which the research is conducted, whichever affords the greater protection to the individual. The study will comply with the requirements of the ICH E2A guideline (Clinical Safety Data Management: Definitions and Standards for Expedited Reporting). Studies conducted in the United States or under a U.S. Investigational New Drug (IND) application will comply with U.S. FDA regulations and applicable local, state, and federal laws. Studies conducted in the European Union or European Economic Area will comply with the E.U. Clinical Trial Directive (2001/20/EC).

8.2 INFORMED CONSENT

The Sponsor’s sample Informed Consent Form (and ancillary sample Informed Consent Forms, if applicable) will be provided to each site. If applicable, it will be provided in a certified translation of the local language. The Sponsor or its designee must review and approve any proposed deviations from the Sponsor's sample Informed Consent Forms or any alternate consent forms proposed by the site (collectively, the "Consent Forms") before IRB/EC submission. The final IRB/EC–approved Consent Forms must be provided to the Sponsor for health authority submission purposes according to local requirements.

If applicable, the Informed Consent Form will contain separate Sections for any optional procedures. The investigator or authorized designee will explain to each patient the objectives, methods, and potential risks associated with each optional procedure. Patients will be told that they are free to refuse to participate and may withdraw their consent at any time for any reason. A separate, specific signature will be required to document a patient's agreement to participate in optional procedures. Patients who decline to participate will not provide a separate signature.

The Consent Forms must be signed and dated by the patient or the patient’s legally authorized representative before his or her participation in the study. The case history or clinical records for each patient shall document the informed consent process and that written informed consent was obtained prior to participation in the study.

The Consent Forms should be revised whenever there are changes to study procedures or when new information becomes available that may affect the willingness of the patient to participate. The final revised IRB/EC-approved Consent Forms must be provided to the Sponsor for health authority submission purposes.

Patients must be re-consented to the most current version of the Consent Forms (or to a significant new information/findings addendum in accordance with applicable laws and IRB/EC policy) during their participation in the study. For any updated or revised

Consent Forms, the case history or clinical records for each patient shall document the informed consent process and that written informed consent was obtained using the updated/revised Consent Forms for continued participation in the study.

A copy of each signed Consent Form must be provided to the patient or the patient's legally authorized representative. All signed and dated Consent Forms must remain in each patient's study file or in the site file and must be available for verification by study monitors at any time.

8.3 INSTITUTIONAL REVIEW BOARD OR ETHICS COMMITTEE

This protocol, the Informed Consent Forms, any information to be given to the patient, and relevant supporting information must be submitted to the IRB/EC by the Principal Investigator and reviewed and approved by the IRB/EC before the study is initiated. In addition, any patient recruitment materials must be approved by the IRB/EC.

The Principal Investigator is responsible for providing written summaries of the status of the study to the IRB/EC annually or more frequently in accordance with the requirements, policies, and procedures established by the IRB/EC. Investigators are also responsible for promptly informing the IRB/EC of any protocol amendments (see Section 9.6).

In addition to the requirements for reporting all adverse events to the Sponsor, investigators must comply with requirements for reporting serious adverse events to the local health authority and IRB/EC. Investigators may receive written IND safety reports or other safety-related communications from the Sponsor. Investigators are responsible for ensuring that such reports are reviewed and processed in accordance with health authority requirements and the policies and procedures established by their IRB/EC, and archived in the site's study file.

8.4 CONFIDENTIALITY

The Sponsor maintains confidentiality standards by coding each patient enrolled in the study through assignment of a unique patient identification number. This means that patient names are not included in data sets that are transmitted to any Sponsor location.

Patient medical information obtained by this study is confidential and may be disclosed to third parties only as permitted by the Informed Consent Form (or separate authorization for use and disclosure of personal health information) signed by the patient, unless permitted or required by law.

Medical information may be given to a patient's personal physician or other appropriate medical personnel responsible for the patient's welfare, for treatment purposes.

Given the complexity and exploratory nature of the analyses, data derived from exploratory biomarker specimens will generally not be provided to study investigators or patients unless required by law. The aggregate results of any conducted research will

be available in accordance with the effective Sponsor policy on study data publication (see Section 9.5).

Data generated by this study must be available for inspection upon request by representatives of national and local health authorities, Sponsor monitors, representatives, and collaborators, and the IRB/EC for each study site, as appropriate.

8.5 FINANCIAL DISCLOSURE

Investigators will provide the Sponsor with sufficient, accurate financial information in accordance with local regulations to allow the Sponsor to submit complete and accurate financial certification or disclosure statements to the appropriate health authorities. Investigators are responsible for providing information on financial interests during the course of the study and for 1 year after completion of the study (i.e., last patient, last visit).

9. STUDY DOCUMENTATION, MONITORING, AND ADMINISTRATION

9.1 STUDY DOCUMENTATION

The investigator must maintain adequate and accurate records to enable the conduct of the study to be fully documented, including, but not limited to, the protocol, protocol amendments, Informed Consent Forms, and documentation of IRB/EC and governmental approval. In addition, at the end of the study, the investigator will receive the patient data, including an audit trail containing a complete record of all changes to data.

9.2 PROTOCOL DEVIATIONS

The investigator should document and explain any protocol deviations. The investigator should promptly report any deviations that might have an impact on patient safety and data integrity to the Sponsor and to the IRB/EC in accordance with established IRB/EC policies and procedures. The Sponsor will review all protocol deviations and assess whether any represent a serious breach of Good Clinical Practice guidelines and require reporting to health authorities. As per the Sponsor's standard operating procedures, prospective requests to deviate from the protocol, including requests to waive protocol eligibility criteria, are not allowed.

9.3 SITE INSPECTIONS

Site visits will be conducted by the Sponsor or an authorized representative for inspection of study data, subjects' medical records, and eCRFs. The investigator will permit national and local health authorities; Sponsor monitors, representatives, and collaborators; and the IRBs/ECs to inspect facilities and records relevant to this study.

9.4 ADMINISTRATIVE STRUCTURE

This trial is sponsored by Genentech. This pilot study will be conducted at study sites experienced in conducting clinical trials in CSU. Data will be recorded via an EDC system from Medidata Solutions (New York, NY) using eCRFs (see Section 7.2). The contract research organization will be responsible for submission to IRB/ECs for approval of the study protocol, patient recruitment, data collection, and reporting. An IxRS will be used to assign patients to treatment groups and to manage ongoing investigational product requests and shipments.

9.5 PUBLICATION OF DATA AND PROTECTION OF TRADE SECRETS

Regardless of the outcome of a trial, the Sponsor is dedicated to openly providing information on the trial to healthcare professionals and to the public, both at scientific congresses and in peer-reviewed journals. The Sponsor will comply with all requirements for publication of study results. For more information, refer to the Roche Global Policy on Sharing of Clinical Trials Data at the following Web site:

www.roche.com/roche_global_policy_on_sharing_of_clinical_study_information.pdf

The results of this study may be published or presented at scientific congresses. For all clinical trials in patients involving an IMP for which a marketing authorization application has been filed or approved in any country, the Sponsor aims to submit a journal manuscript reporting primary clinical trial results within 6 months after the availability of the respective Clinical Study Report. In addition, for all clinical trials in patients involving an IMP for which a marketing authorization application has been filed or approved in any country, the Sponsor aims to publish results from analyses of additional endpoints and exploratory data that are clinically meaningful and statistically sound.

The investigator must agree to submit all manuscripts or abstracts to the Sponsor prior to submission for publication or presentation. This allows the Sponsor to protect proprietary information and to provide comments based on information from other studies that may not yet be available to the investigator.

In accordance with standard editorial and ethical practice, the Sponsor will generally support publication of multicenter trials only in their entirety and not as individual center data. In this case, a coordinating investigator will be designated by mutual agreement.

Authorship will be determined by mutual agreement and in line with International Committee of Medical Journal Editors authorship requirements. Any formal publication of the study in which contribution of Sponsor personnel exceeded that of conventional monitoring will be considered as a joint publication by the investigator and the appropriate Sponsor personnel.

Any inventions and resulting patents, improvements, and/or know-how originating from the use of data from this study will become and remain the exclusive and unburdened property of the Sponsor, except where agreed otherwise.

9.6 PROTOCOL AMENDMENTS

Any protocol amendments will be prepared by the Sponsor. Protocol amendments will be submitted to the IRB/EC and to regulatory authorities in accordance with local regulatory requirements.

Approval must be obtained from the IRB/EC and regulatory authorities (as locally required) before implementation of any changes, except for changes necessary to eliminate an immediate hazard to patients or changes that involve logistical or administrative aspects only (e.g., change in Medical Monitor or contact information).

10. REFERENCES

Altrichter S, Peter HJ, Pisarevskaja D, et al. IgE mediated autoallergy against thyroid peroxidase–a novel pathomechanism of chronic spontaneous urticaria? PLoS ONE 2011;6:e14794.

Broides A, Yang W, Conley ME. Genotype/phenotype correlations in X-linked agammaglobulinemia. Clin Immunol 2006;118:195–200.

Bruton OC. Agammaglobulinemia. Pediatrics 1952;9:722–8.

Bugatti S, Vitolo B, Caporali R, et al. B cells in rheumatoid arthritis: from pathogenic players to disease biomarkers. Biomed Res Int 2014;2014:681678. doi: 10.1155/2014/681678

Conley ME, Broides A, Hernandez-Trujillo V, et al. Genetic anaylis of patients with defects in early B-cell development. Immunol Rev 2005;203:216–34.

Di Paolo JA, Huang T, Balazs M, et al. Specific BTK Inhibition suppresses B cell and myeloid cell-mediated arthritis. Nat Chem Biol 2011;7:41–50.

[FDA] U.S. Food and Drug Administration. Pharmacology review of Incivek (telaprevir). 2011. Available from: http://www.accessdata.fda.gov/drugsatfda_docs/nda/2011/201917Orig1s000TOC.cfm

Ferrer M. Immunological events in chronic spontaneous urticaria. Clin Transl Allergy 2015;5:360. doi: 10.1186/s13601-015-0074-7

Fiebiger E, Maurer D, Holub H, et al. Serum IgG autoantibodies directed against the α chain of FcεRI: a selective marker and pathogenetic factor for a distinct subset of chronic urticaria patients? J Clin Invest 1995;96:2606–12. doi: 10.1172/JCI118325

Greaves M. Chronic urticaria. J Allergy Clin Immunol 2000;105:664–72.

Greaves M. Chronic idiopathic urticaria. Curr Opin Allergy Clin Immunol 2003;3:363–8.

Hata D, Kawakami Y, Inagaki N, et al. Involvement of Bruton's tyrosine kinase in FcεRI-dependent mast cell degranulation and cytokine production. J Exp Med. 1998 Apr 20;187:1235–47.

Inman WH, Vessey MP, Westerholm B, et al. Thromboembolic disease and the steroidal content of oral contraceptives. A report to the Committee on Safety of Drugs. Br Med J 1970;2:203–9.

Iyer AS, Morales JL, Huang W, et al. Absence of Tec family kinases interleukin-2 inducible T cell kinase (Itk) and Bruton's tyrosine kinase (Btk) severely impairs FcεRI-dependent mast cell responses. J Bio Chem 2011;286:9503–13. doi: 10.1074/jbc.M110.1656131

Kaplan AP. Chronic urticaria and angioedema. N Engl J Med 2002;346:175–9.

Kaveri SV, Maddur MS, Hegde P, et al. Intravenous immunoglobulins in immunodeficiences: more that mere replacement therapy. Clin Exp Immunol 2011;164(Suppl 2):2–5. doi: 10.1111/j.1365-2249.2011.04387.x

Kay AB, Ying S, Ardelean E, et al. Elevations in vascular markers and eosinophils in chronic spontaneous urticarial weals with low-level persistence in uninvolved skin. Br J Dermatol 2014;171:505–11. doi: 10.1111/bjd.12991

Kellick KA, Bottorff M, Toth PP, et al. A clinician's guide to statin drug-drug interactions. J Clin Lipidol 2014 May-Jun;8(3 Suppl):S30-46.

Kern F, Lichtenstein LM. Defective histamine release in chronic urticaria. J Clin Invest 1976;57:1369–77.

Kozel MA, Sabroe RA. Chronic urticaria, aetiology, management and current and future treatment options. Drugs 2004;64:2515–36.

[redacted]

Lederman HM, Winkelstein JA. X-linked agammaglobulinema: an analysis of 96 patients. Medicine (Baltimore) 1985;64:145–56.

Liu L, Di Paolo J, Barbosa J, et al. Antiarthritis effect of a novel Bruton's tyrosine kinase (BTK) inhibitor in rat collagen-induced arthritis and mechanism-based pharmacokinetic/pharmacodynamic modeling: relationships between inhibition of BTK phosphorylation and efficacy. J Pharmacol Exp Ther 2011;338:154–63.

Luquin E, Kaplan AP, Ferrer M. Increased responsiveness of basophils of patients with chronic urticaria to sera but hypo-responsiveness to other stimuli. Clin Exp Allergy 2005;35:456–60. doi: 10.1111/j.1365-2222.2005.02212.x

Mathias SD, Crosby RD, Zazzali JL, et al. Evaluating the minimally important difference of the urticaria activity score and other measures of disease activity in patients with chronic idiopathic urticaria. Ann Allergy Asthma Immunol 2012;108:20–4. doi: 10.1016/j.anai.2011.09.008

Maurer M, Weller K, Bindslev-Jensen C, et al. Unmet clinical needs in chronic spontaneous urticaria. A GA2LEN task force report. Allergy 2011;66:317–30.

McGirt LY, Vasagar K, Gober LM, et al. Successful treatment of recalcitrant chronic idiopathic urticaria with sulfasalazine. Arch Dermatol 2006;142:1337–42.

Medicinal Products Act in the version published on 12 December 2005 (Federal Law Gazette [BGBl.]) Part I p. 3394, last amended by Article 2a of the Law of 27 March 2014 (Federal Law Gazette I p. 261).

Misbah SA, Spickett GP, Ryba PC, et al. Chronic enteroviral meningoencephalitis in agammaglobulinemia: case report and literature review. J Clin Immunol 1992;12:266–70.

Niiro H, Clark EA. Regulation of B-cell fate by antigen-receptor signals. Nat Rev Immunol 2002;2:945–56.

O'Kelly M, Ratitch B. Clinical trials with missing data: a guide for practitioners. Chichester, UK: John Wiley & Sons, 2014.

Powell RJ, Leech SC, Till S, et al. BSACI guideline for the management of chronic urticaria and angioedema. Clin Exp Allergy 2015;45:547–65. doi: 10.1111/cea.12494

Puri K, Di Paolo J, Gold M. B-cell receptor signaling inhibitors for treatment of autoimmune inflammatory diseases and B-cell malignancies. Int Rev Immunol 2013;32:397–427.

Reth M, Nielsen P. Signaling circuits in early B-cell development. Adv Immunol 2014;122:129–75. doi: 10.1016/B978-0-12-800267-4.00004-3

Saini SS. Chronic spontaneuos urticaria: etiology and pathogensis. Immunol Allergy Clin North Am 2014;34:33–52. doi: 10.1016/j.iac.2013.09.012

Sochorová K, Horvath R, Rozkova D, et al. Impaired toll-like receptor 8-mediated IL-6 and TNF-α production in antigen-presenting cells from patients with X-linked agammaglobulinemia. Blood 2007;109:2553–6.

Snyder PW, Kazacos EA, Scott-Moncrieff JC, et al. Pathologic features of naturally occurring juvenile polyarteritis in beagle dogs. Vet Pathol 1995;32:337–45.

Tilles SA. Approach to therapy in chronic urticaria: when Benadryl is not enough. Allergy Asthma Proc 2005;26:9–12.

Tong LJ, Balakrishnan G, Kochan J. et al. Assessment of autoimmunity in patients with chronic urticaria. J Allergy Clin Immunol 1997;99:461–5.

Tsukada S, Saffran DC, Rawlings DJ, et al. Deficient expression of a B cell cytoplasmic tyrosine kinase in human X-linked agammaglobulinemia. Cell 1993;72:279–90. doi:10.1016/0092-8674(93)90667-F

van den Bruele T, Mourad-Baars PE, Claas EC, et al. Campylobacter jejuni bacteremia and Helicobacter pylori in a patient with X-linked agammaglobulinemia. Eur J Clin Microbiol Infect Dis 2010;29:1315–9.

Vetrie D, Vořechovský I, Sideras P, et al. The gene involved in X-linked agammaglobulinaemia is a member of the src family of protein-tyrosine kinases. Nature 1993;361:226–33. doi:10.1038/361226a0

Wang B, Sanchez RI, Franklin RB, et al. The involvement of CYP3A4 and CYP2C9 in the metabolism of 17 alpha-ethinylestradiol. Drug Metab Dispos 2004;32:1209–12.

Whang JA, Chang BY. Bruton's tyrosine kinase inhibitors for the treatment of rheumatoid arthritis. Drug Discov Today 2014;19:1200–4. doi: 10.1016/j.drudis.2014.03.028

Winkelstein JA, Marino MC, Lederman HM, et al. X-linked agammaglobulinema: report on a United States registry of 201 patients. Medicine (Baltimore) 2006;85:193–202.

Ying S, Kikuchi Y, Meng Q, et al. TH1/TH2 cytokines and inflammatory cells in skin biopsy specimens from patients with chronic idiopathic urticaria: comparison with the allergen-induced late-phase cutaneous reaction. J Allergy Clin Immunol 2002;109:694–700.

Zhang H, Cui D, Wang B, et al. Pharmacokinetic drug interactions involving 17alpha-ethinylestradiol: a new look at an old drug. Clin Pharmacokinet 2007;46:133–57.

Ziegner UH, Kobayashi RH, Cunningham-Rundles C, et al. Progressive neurodegeneration in patients with primary immunodeficiency disease on IVIG treatment. Clin Immunol 2002;102:19–24.

Zweiman B, Valenzano M, Atkins PC. Modulation of serum histamine releasing activity in chronic idiopathic urticaria. Immunopharmacology 1998;39:225–34.

Appendix 1
Schedule of Activities (Cohorts 1 and 2)

									Safety Follow Up [b]		
			Treatment Period								
Week	Screening [a]		0	1	2	4	6 [c]	8	12		
Day (± days)	–14 (–4/+2)	–7	1 [d]	8 [d] (±1)	15 (±1)	29 [d] (±2)	43 (±2)	57 [d, e] (±2)	85 [d] (±2)	Early Term.	Unscheduled Visit
Informed consent	x										
Demographic data	x										
General medical history and baseline conditions	x										
Inclusion/exclusion criteria	x	x	x								
Randomization			x								
Train patient to complete Urticaria Patient Daily eDiary (including UAS) [f]	x	x									
Distribute eDiary to patient [g]	x										
Urticaria Patient Daily eDiary [g]		x	x	x	x	x	x	x	x	x	
[redacted]			x			x		x	x		
Concomitant medications [i]	x	x	x	x	x	x	x	x	x	x	x
Adverse events			x	x	x	x	x	x	x	x	x
Vital signs [j]	x	x	x	x	x	x		x	x	x	x [k]
Height	x										
Weight	x		x			x		x	x	x	x [k]
Complete physical examination [l]	x								x		
Limited physical examination [m]		x	x	x	x	x		x		x	x [k]
ECG [n]	x		x	x					x	x	x [k]
Hepatitis Screening [o]	x										
QFT (PPD if QFT not available)	x										

Appendix 1
Schedule of Activities (Cohorts 1 and 2) (cont.)

	Screening [a]		Treatment Period						Safety Follow Up [b]		
Week			0	1	2	4	6 [c]	8	12		
Day (± days)	−14 (−4/+2)	−7	1 [d]	8 [d] (±1)	15 (±1)	29[d] (±2)	43 (±2)	57[d, e] (±2)	85 [d] (±2)	Early Term.	Unscheduled Visit
Chest X-ray [p]	x										
Fenebrutinib/placebo administration in clinic[q, r]			x	x	x	x					
Drug Dispensing			x			x					
Hematology[s]	x	x	x	x	x	x		x	x	x	x[k]
Chemistry[t]	x	x	x	x	x	x		x	x	x	x[k]
Fasting Lipid Panel			x			x		x	x		
Coagulation studies [u]	x									x	x[k]
Pregnancy test[v]	x		x	x	x	x		x	x	x	
Urinalysis[w]	x		x					x	x	x	
[redacted]	x								x	x	
[redacted]	x								x	x	
FricTest (for patients with dermographism only)	x		x					x	x	x	
Plasma PK assessment[y]			x	x				x		x	x[k]
[redacted]			x								
[redacted]	x		x					x	x	x	
[redacted]	x		x					x	x	x	
[redacted]			x					x			
Phone call [bb]							x				

BID = twice a day; eCRF = electronic case report form; eDiary = electronic diary (patient reported outcomes); HBcAb = hepatitis B core antibody; HBsAb = hepatitis B surface antibody; HBsAg = hepatitis B surface antigen; HCV Ab = hepatitis C antibody; PD = pharmacodynamic; PK = pharmacokinetic; PPD = purified protein derivative; PPI = proton pump inhibitor; QFT = QuantiFERON-TB Gold®; QTcF = QT interval corrected using Fridericia's formula; TB = tuberculosis; [redacted] Term. = Termination; UAS = Urticaria Activity Score; UAS7 = Urticaria Activity Score over 7 days [redacted].

[a] Laboratory tests prior to randomization and dosing may be performed locally on Day 0, if central laboratory tests are not available due to sampling handling problems, breakage, or lab error.

[b] Safety follow-up visit 4 weeks after the last dose of study drug if the patient discontinues the study and/or study treatment (per Sections 4.6.1 and 4.6.2) prior to Day 85 visit.

[c] Phone call instead of a clinic visit.

[d] Morning clinic visit is required for visits on Days 1, 8, 29, 57, and 85; for other study visits, morning visits are recommended. For mandatory morning visits, the patient should be fasting (overnight, > 8 hours) prior to the first PK blood draw and/or fasting lipid panel.

[e] Day 57 visit is the last day of the study treatment period; however, no study drug will be taken at the Day 57 visit. The last dose of blinded study drug will be the p.m. dose on Day 56 or the day before the Day 57 visit if it does not occur on Day 57.

[f] Patients should be trained to use the eDiary at Day –14. At Day –7, staff should query patients for any questions they may have concerning the use of the eDiary and ensure patients understand correct usage before randomization.

[g] Patient is to complete the eDiary twice daily, approximately every 12 hours (a.m./p.m.), every day for the duration of the study. The eDiary includes the UAS7 (itch score, number of hives) and other patient reported outcomes.

[redacted]

[i] Includes any medication (e.g., prescription drugs, over-the-counter drugs, vaccines, herbal or homeopathic remedies, vitamins, and nutritional or dietary supplements) used by a patient from 12 weeks prior to initiation of study drug until 4 weeks after the last dose of study drug. In addition, at each clinic visit, any use of PPIs, H2 receptor antagonists, and/or other antacids (e.g., Pepto-Bismol®, Rolaids®) should be recorded as concomitant medications, including the date, dose, and time of last administration.

[j] Includes respiratory rate, pulse rate, temperature, and systolic and diastolic blood pressures while the patient is in a seated position for at least 5 minutes.

[k] This procedure is optional per the investigator's discretion.

[l] A complete physical examination should include an evaluation of the head, eyes, ears, nose, and throat and the cardiovascular, dermatological, musculoskeletal, respiratory, gastrointestinal, and neurological systems. Patients should be screened for dermographism. Any abnormality identified at baseline should be recorded on the General Medical History and Baseline Condition eCRF.

[m] Perform a limited, symptom-directed examination at specified timepoints or as clinically indicated. Record new or worsened clinically significant abnormalities on the Adverse Event eCRF.

[n] Interpretable digital ECG recording (e.g., without artifacts) will be obtained. The ECG intervals (e.g., PR, QRS, QT, QTcF, and RR) and heart rate from the ECGs will be entered into the eCRF; ECGs for each patient should be obtained from the same machine whenever possible. ECGs can be performed without specific restrictions (e.g., can be any time of day, before or after dosing, fasting or fed) but are to be obtained prior to other procedures scheduled at that same time (e.g., vital sign measurements, blood draws). ECGs must be performed after the patient has been resting in a supine position for at least 10 minutes prior to beginning the ECG recording. Environmental distractions (e.g., television, radio, conversation) should be avoided during the pre-ECG resting period and during ECG recording.

[o] HBsAg, HBsAb, HBcAb, and HCV Ab.

[p] Performed only if required by local guidelines to rule out active TB infection.

[q] On mandatory morning clinic visit days during the treatment period (Days 1, 8, 29, and 57), patients should be instructed that the morning dose of study drug will be taken in the clinic. On other clinic visit days, if the visit occurs in the morning, the patient should be instructed that the morning dose of study drug will be taken in the clinic. The morning dose should be taken after all pre-dose assessments are complete (i.e., ECG, questionnaires, and PK and biomarker sample collection).

[e] Patients will take fenebrutinib/placebo BID approximately every 12 hours starting on Day 1 and ending on Day 56 (pm) or the day before (pm) the Day 57 visit if it does not occur on Day 57. One dose (a total of 4 tablets) of fenebrutinib/placebo should be taken with water by mouth BID (a total of 8 tablets each day). The dates and times of the most recent prior meal, last dose of oral study drug (prior to clinic visit), and timing of study drug administration in clinic should be recorded at each clinic visit.

[s] Includes WBC count, RBC count, hemoglobin, hematocrit, platelet count, and WBC differential (i.e., neutrophils, eosinophils, basophils, monocytes, lymphocytes, and other cells if present).

[t] Includes sodium, potassium, chloride, bicarbonate, glucose, BUN or urea, creatinine, total protein, albumin, phosphorus, calcium, total and direct bilirubin, alkaline phosphatase, ALT, AST, uric acid, LDH, CRP, and lipase.

[u] Includes PT, PTT, INR, and fibrinogen.

[v] All women of childbearing potential, including those who have had a tubal ligation, will have a serum pregnancy test at screening. Urine pregnancy tests will be performed locally at specified subsequent visits. If a urine pregnancy test result is positive, it must be confirmed by a serum pregnancy test (performed locally).

[w] Includes dipstick, including pH, specific gravity, glucose, protein, ketones, blood, and microscopic examination (e.g., sediment, RBCs, WBCs, casts, crystals, epithelial cells, bacteria if present).

[redacted]

[y] Collect PK samples prior to drug administration, and the patient should be fasting overnight for $>$8 hours.

[redacted]

[redacted]

[bb] Site staff to remind patients to take the study drug and to complete their eDiaries.

Appendix 2
Childbearing Potential, Pregnancy Testing, and Contraception

For Women

All women of childbearing potential (including those who have had a tubal ligation) will have a serum pregnancy test at screening and a urine pregnancy test on Study Day 1 prior to administration of study drug and monthly at appropriate clinic visits. If a urine pregnancy test result is positive, study drug will not be administered until pregnancy is ruled out. The result must be confirmed by a serum pregnancy test (conducted by the local laboratory). Refer to Section 5.4.3 of the protocol for management of a patient with a confirmed pregnancy.

All female patients are considered to be of childbearing potential unless they meet one of the following criteria:

- The patient has been postmenopausal (non−therapy-induced amenorrhea) for at least 12 continuous months with no other identified cause.
- The patient had a surgical bilateral oophorectomy (with or without hysterectomy) more than 6 weeks prior to enrollment.
- The patient had a hysterectomy.

Female patients of reproductive or childbearing potential who are unwilling to use a method of contraception that results in a failure rate of < 1 % per year or remain abstinent (refrain from heterosexual intercourse), and refrain from donating eggs during the treatment period and for at least 4 weeks after the last dose of study drug will be excluded from study participation.

Abstinence is acceptable only if it is in line with the preferred and usual lifestyle of the patient. Periodic abstinence (e.g., calendar, ovulation, symptothermal, or post-ovulation methods) and withdrawal are not acceptable methods of contraception.

Examples of contraceptive methods with a failure rate of < 1% per year include the following:

- Sterilization, bilateral surgical tubal ligation
- Intrauterine device
- Combined oral contraceptive pill [1]
- Contraceptive transdermal patch (estrogen and progestin containing)[1]
- Hormonal vaginal device
- Progestogen-only hormonal contraception associated with inhibition of ovulation
- Implants for contraception
- Injections for contraception (with prolonged release)

- Sole sexual partner consisting of surgically sterilized male partner with appropriate postsurgical verification of the absence of spermatozoa in the ejaculate. Patients may provide verbal confirmation that the partner completed appropriate follow-up after vasectomy. Sites are not required to obtain partner medical records.

[1] Women using estrogen-containing hormonal contraceptives as a method of contraception must also use a barrier such as a male condom in conjunction with the hormonal contraceptives.

- **For Men**:
- All men must agree to remain abstinent (refrain from heterosexual intercourse) or use contraceptive measures, and agreement to refrain from donating sperm, as defined below:
- With female partners of childbearing potential or pregnant female partners, men must remain abstinent or use a condom during the treatment period and for at least 4 weeks after the last dose of fenebrutinib to avoid exposing the embryo. Men must refrain from donating sperm during this same period.

For Men and Women

The reliability of sexual abstinence should be evaluated in relation to the duration of the clinical trial and the preferred and usual lifestyle of the patient. Periodic abstinence (e.g., calendar, ovulation, symptothermal, and post-ovulation methods) and withdrawal are not acceptable methods of contraception.

Appendix 3
Urticaria Patient Daily eDiary

<u>General Instructions</u>

Please answer each question to the best of your ability.

There are no right or wrong answers.

For each question, please choose the response that describes your experience.

Please pay close attention to the timeframe of interest. Some questions ask about the **<u>past 12 hours</u>**, while others ask about the **<u>past 24 hours</u>**.

<u>Instructions for Counting the Number of Hives and Measuring the Size of the Largest Hive</u>

Count each hive separately even if you have more than one hive grouped together with other hives.

Please use the ruler that you have been given to measure the size of your largest hive. If you need help, please have someone else take this measurement for you. **Please do not measure a group of hives as one hive.**

Today's Date

		-				-				

Day **Month** **Year**

Please complete this Section <u>every morning</u> throughout the duration of the study. (Please circle only one response.)

1. Thinking about the **<u>past 12 hours</u>**, please record the severity of itch and the number of hives you may have had associated with your skin condition. **<u>Please count each hive separately</u>** even if you have more than one hive grouped together with other hives.

Itch (severity)	Hives (number)
0 = none<br>1 = mild<br>2 = moderate<br>3 = severe	0 = none<br>1 = between 1 and 6 hives<br>2 = between 7 and 12 hives<br>3 = greater than 12 hives

This next question asks you to estimate the size of your largest hive in centimeters (cm). Please use the ruler that you have been provided with to make this measurement. If your largest hive is located on your back or in a place that is hard to reach, please have someone else take this measurement for you. When measuring the largest hive size, **<u>please do not measure a group of hives as one hive.</u>**

Largest Hive (size)
0 = none<br>1 = less than 1.25 centimeter (cm)<br>2 = between 1.25 centimeter (cm) and 2.5 centimeters (cm)<br>3 = greater than 2.5 centimeters (cm)

Appendix 3
Urticaria Patient Daily eDiary (cont.)

Today's Date

		-				-				

Day **Month** **Year**

Please complete this Section <u>every evening</u> throughout the duration of the study. (Please circle only one response.)

2. Thinking about the **<u>past 12 hours</u>**, please record the severity of itch and the number of hives you may have had associated with your skin condition. **<u>Please count each hive separately</u>** even if you have more one than one hive grouped together with other hives.

Itch (severity)	Hives (number)
0 = none<br>1 = mild<br>2 = moderate<br>3 = severe	0 = none<br>1 = between 1 and 6 hives<br>2 = between 7 and 12 hives<br>3 = greater than 12 hives

This next question asks you to estimate the size of your largest hive in centimeters (cm). Please use the ruler that you have been provided with to make this measurement. If your largest hive is located on your back or in a place that is hard to reach, please have someone else take this measurement for you. When measuring the largest hive size, **<u>please do not measure a group of hives as one hive.</u>**

Largest Hive (size)
0 = none<br>1 = less than 1.25 centimeter (cm)<br>2 = between 1.25 centimeter (cm) and 2.5 centimeters (cm)<br>3 = greater than 2.5 centimeters (cm)

Appendix 3
Urticaria Patient Daily eDiary (cont.)

Today's Date

		–				–				

Day **Month** **Year**

Please complete this Section *once* ***each day throughout the duration of the study (preferably at the same time each day).***

(Please circle only one response.)

3. Please rate how much your hives or itch interfered with your sleep during the **past 24 hours**.

   0 No interference

   1 Mild, little interference with sleep

   2 Moderate, awoke occasionally, some interference with sleep

   3 Substantial, woke up often, severe interference with sleep

4. Please rate how much your hives or itch interfered with your daily activities during **the past 24 hours**. This could include work, school, sports, hobbies, and activities with friends and family.

   0 No interference

   1 Mild, little interference with daily activities

   2 Moderate, some interference with daily activities

   3 Substantial, severe interference with daily activities

These next questions are about your symptoms and how you managed them during the past 24 hours.

5. During the **past 24 hours**, did you use loratadine or cetirizine in order to control symptoms of your skin condition such as itch or hives?

   0 = No

   1 = Yes

6a. During the **past 24 hours**, did you have any rapid swelling on your face, (especially your eyelids or lips), inside your mouth (including your throat or tongue), or elsewhere on your body? This rapid swelling, also called angioedema, is at a deeper level under your skin than hives.

   0 = No **(GO TO Question 7)**   1 = Yes

6b. If Yes, how did you treat this rapid swelling? **(Circle all that apply.)**

   0 Did nothing (GO TO Question 7)

   1 Took some prescription or non-prescription medication

   2 Called my doctor, nurse or nurse practitioner

   3 Went to see my doctor, nurse, or nurse practitioner

   4 Went to the emergency room at the hospital

   5 Was hospitalized

7. During the **past 24 hours**, did you or someone else call your doctor, nurse or nurse practitioner because of your skin condition?

   0 = No   1 = Yes

[redacted]

[redacted]

[redacted]

[redacted]

[redacted]

[redacted]

[redacted] [redacted] [redacted] [redacted] [redacted]

[redacted]

[redacted] [redacted] [redacted] [redacted] [redacted]

[redacted]

[redacted] [redacted] [redacted] [redacted] [redacted]

[redacted]

[redacted] [redacted] [redacted] [redacted] [redacted]

Appendix 5
Concomitant Medications (Including Foods and Herbal Products)

Class	Expected Interaction	Recommendation	Examples of Drugs in this Class [a]
Antacids	Decreased fenebrutinib absorption due to increased gastric pH	Take fenebrutinib 2 hours before or 2 hours after antacid	• Pepto-Bismol, Rolaids
Moderate or strong CYP3A inhibitors	Increased fenebrutinib plasma concentrations due to inhibition of metabolism	Avoid for 7 days or 5 half-lives (whichever is longer) prior to first dose of study drug and during the treatment period	• Antimicrobials (clarithromycin, erythromycin, itraconazole, ketoconazole, telithromycin, troleandomycin, voriconazole, posaconazole)<br>• Antidepressants (nefazodone)<br>• Antihypertensive/cardiac (verapamil, diltiazem)<br>• Other (grapefruit juice, Seville orange juice, pomegranate, star fruit)
CYP3A inducers	Decreased fenebrutinib plasma concentrations due to increased metabolism	Avoid for 7 days or 5 half-lives (whichever is longer) prior to first dose of study drug and during the treatment period	• Antimicrobials (rifampin, rifapentine, rifabutin)<br>• Antidepressants (St. John's wort, hyperforin)<br>• Antiepileptics (carbamazepine, phenytoin, phenobarbital, hyperforin)<br>• Diabetes (pioglitazone, troglitazone)<br>• Other (modafinil, bosentan)

Class	Expected Interaction	Recommendation	Examples of Drugs in this Class [a]
Sensitive and narrow therapeutic window CYP3A substrates	Potential for increased plasma concentrations of CYP3A substrates due to inhibition of metabolism by fenebrutinib	Use with caution and monitor for adverse events related to CYP3A substrates as directed by product labeling; consult with the Medical Monitor as needed	• Antiemetic/prokinetic (aprepitant, cisapride)<br>• Antihistamine (astemizole, terfenadine)<br>• Antihypertensive/cardiac (dronedarone, eplerenone, felodipine, nisoldipine, quinidine, ticagrelor, vardenafil)<br>• Benzodiazepines (alprazolam, diazepam, midazolam)<br>• Lipid-lowering (simvastatin [recommended maximum dose: 10 mg/day], lovastatin [recommended maximum dose: 20 mg/day])<br>• Migraine (eletriptan, ergotamine)<br>• Steroids (budesonide, fluticasone)<br>• Other (alfentanil, buspirone, conivaptan, darifenacin, dasatinib, dihydroergotamine, fentanyl, lurasidone, pimozide, quetiapine, sildenafil, tolvaptan, triazolam)
BCRP substrates with a narrow therapeutic index	Potential for increased plasma concentrations of BCRP substrates due to inhibition of transport by fenebrutinib	Use with caution and monitor for adverse events related to BCRP substrates as directed by product labeling; consult with the Medical Monitor as needed	• Antihypertensive (prazosin)<br>• Anti-inflammatory (sulfasalazine)<br>• Lipid-lowering (rosuvastatin [recommended maximum dose: 10 mg/day], atorvastatin [recommended maximum dose: 20 mg/day]<br>• Muscle relaxants (dantrolene)<br>• Steroids (estrone-3-sulfate)

[a] The following list is not comprehensive. Please refer to the following websites for additional information and consult the Medical Monitor if necessary:

U.S. FDA Table of Substrates, Inhibitors, and Inducers (Tables 3-1, 3-2, 3-3, and 5-1) (http://www.fda.gov/Drugs/DevelopmentApprovalProcess/DevelopmentResources/DrugInteractionsLabeling/ucm093664.htm)

Indiana University Department of Medicine P450 Interaction Table (http://medicine.iupui.edu/clinpharm/ddis/clinical-table)

Summary of Protocol Amendments
(including changes to Statistical Considerations and Analysis Plan)

<table>
<tr><th>Version</th><th>Amendment</th></tr>
<tr><td>V2
(Germany)
11-Apr-2017</td><td>Protocol Amendment, Version 2
Rationale
The GS39684 protocol has been amended for German sites to reflect the input from the German health authority, the Paul-Ehrlich-Institut (PEI), as follows:
• To ensure that the maximum dose of cetirizine or loratadine permitted by the treatment guidelines will not be exceeded by adding these as rescue medications
• To exclude patients with moderately or severely impaired renal function
• To exclude patients with moderate anemia
• To clarify retesting and re-screening procedures
• To exclude patients who receive specific drugs metabolized by the CYP3A4/5 pathway (astemizole, terfenadine, and ebastine)

In addition, minor changes were made in Section 5.3.5, Procedures for Recording Adverse Events, in accordance with recent updates to the Sponsor’s protocol model document. This amendment represents cumulative changes to the original protocol.</td></tr>
<tr><td>V3
19-Dec-2017</td><td>PROTOCOL AMENDMENT, VERSION 3: RATIONALE

Protocol GS39684 was amended in April 2017 (Version 2) for German sites to reflect the input from the German health authority, the Bundesinstitut für Arzneimittel und Medizinprodukte (BfArM), as follows:
• To ensure that the maximum dose of cetirizine or loratadine permitted by the treatment guidelines will not be exceeded by adding these as rescue medications (Section 3.1)
• To exclude patients with moderately or severely impaired renal function (Section 4.1.2)
• To exclude patients with moderate anemia (Section 4.1.2)
• To clarify retesting and re-screening procedures (Sections 4.5.2.1 and 4.5.2.2)
• To exclude patients who receive specific drugs metabolized by the CYP3A4/5 pathway (astemizole, terfenadine, and ebastine) (Section 4.1.2)

In this global version (GS39684, Version 3), the above changes have been incorporated. Additionally, the following new changes have been incorporated:

• A dose-ranging cohort (Cohort 2) gated on the basis of results from an interim analysis of Cohort 1 has been added (Sections 3.1, 4.1). Language has been updated throughout the protocol to include the additional cohort.
    • An internal monitoring committee for Cohort 2 has been added (Section 3.1.1). This committee will conduct periodic safety reviews and any optional interim analyses.
    • The length of study has been revised to accommodate Cohort 2 (Section 3.2).
    • The dose rationale section has been updated to reflect the current study design (Section 3.3.1).
    • Randomization and Blinding had been updated to include</td></tr>
</table>

	Cohort 2 (Section 4.2.1).<br>• Study Treatment sections have been updated to include current study design (Sections 4.3.1 and 4.3.2.1).<br>• Instructions regarding the use of blister wallets and the recording of medication use into the eDiary for Cohort 2 have been added (Section 4.3.2.2).<br>• The statistics sections have been updated to reflect the current study design (throughout Section 6).<br>• Interim analysis language to ungate Cohort 2 has been added (Section 6.8.1).<br>• Optional interim analysis language for Cohort 2 has been added (Section 6.8.2).<br>• Background information on GDC-0853 has been updated (Section 1.2.3).<br>• Method of randomization has been clarified (Section 4.2.1).<br>[redacted]<br>• It has been clarified that laboratory tests prior to randomization and dosing may be performed locally on Day 0, if central laboratory tests are not available due to sampling handling problems, breakage, or lab error (Section 4.5.7).<br>• Preliminary results from the ongoing clinical drug-drug interaction study GP39616 indicate that GDC-0853 is a mild CYP3A inhibitor and a breast cancer resistance protein (BCRP) inhibitor as well as a moderately sensitive substrate of CYP3A. Section 4.4.2.2 has been updated to include this information, and recommendations with respect to certain classes of concomitant medications have been revised accordingly. An Appendix with a list of Concomitant Medications has been added (Appendix 5).<br>• The reporting of the term “sudden death” has been updated to also require the presumed cause of death (Section 5.3.5.8).<br>• Event reporting for hospitalization has been clarified (Section 5.3.5.11).<br>• Content from previous protocol clarification letters has been added, which includes the following:<br>– The inclusion criterion for assessment of tuberculosis has been clarified (Section 4.1.1).<br>– Guidelines for patient discontinuation and treatment discontinuation have been clarified (Section 4.6.1 and 4.6.2).<br>– Guidelines for study and site discontinuation have been clarified (Section 4.6.3).<br>– Laboratory requirements were corrected to be consistent throughout the protocol (Section 4.5.7, Appendix 1).<br>– The days for urticaria control testing has been corrected in the

	Schedule of Assessments (Appendix 1).<br>– The days for drawing plasma PK samples has been corrected to align with the Schedule of Assessments (Section 4.4.4).<br>– The prohibited medication washout duration has been clarified (Section 4.4.2).<br>– The requirement for Urticaria Patient Daily eDiary entries has been clarified (Section 4.1.1).<br>• The Informed Consent Forms have been updated to reflect changes to the protocol.<br><br>Additional minor changes have been made to improve clarity and consistency. Substantive new information appears in italics. This amendment represents cumulative changes to the original protocol.
V4<br>09-Aug-2018	**PROTOCOL AMENDMENT, VERSION 4: RATIONALE**<br><br>Protocol GS39684 has been amended to allow patients the option to enter an open-label extension (OLE) Study GS40868 after completing the 8-week treatment period. Language has been updated throughout the protocol to include the OLE (Sections 3.1, 3.1.2, and 4.3.2.1 and Appendix 1).<br><br>The following additional changes have been made:<br>• The name of the study drug has been updated from GDC-0853 to fenebrutinib throughout the document.<br>• The Medical Monitor has changed.<br>• General enrollment update for subjects who received fenebrutinib or placebo (Section 1.2.3).<br>• Safety data regarding hepatotoxicity has been updated (Section 1.2.3, 1.3.4, and 5.1.1.6).<br>• Clarification regarding the use of rescue medication (Section 3.1).<br>• Clarification of contradictory language regarding tubal ligation since bilateral tubal ligation is an example of a contraceptive method with a failure rate of $<1\%$ per year (Section 4.1.1).<br>• The inclusion criterion that women must refrain from donating eggs during the treatment period and for at least 4 weeks after the last dose of study drug has been added (Section 4.1.1)<br>• Clarification of fasting before morning clinic visits (Section 4.3.2.1)<br>• Bilastine dose range has been updated based on daily-recommended dose of 20 mg (Section 4.3.2.3).<br>• Recommendations have been updated regarding the maximum dose of several lipid-lowering agents (statins) that are metabolized by CYP3A (simvastatin, lovastatin) and/or transported by BCRP (rosuvastatin, atorvastatin), and thus may be affected by drug-drug interaction with fenebrutinib (Section 4.4.2.2 and Appendix 5).<br>• Clarification regarding the effect of ethinyl estradiol with fenebrutinib has been added (Section 4.4.2.3).

	• Clarification regarding the use of prohibited therapies and corticosteroids for exacerbations has been added (Section 4.4.3).<br>• Eligibility criteria for re-screening have been clarified (Section 4.5.2.2).<br>• Added language to clarify use of samples after withdrawal of patient consent (Section 4.5.7).<br>• Contradictory language between Section 4.5.8 and the Schedule of Activities regarding electrocardiograms has been corrected.<br>• Clarification of the UAS7 definition (Section 4.5.9.2).<br>[redacted]<br>[redacted]<br>• Additional information around infections has been added (Section 5.1.1.1).<br>• Additional information around bleeding has been added (Section 5.1.1.3).<br>• Additional information around gastrointestinal effects has been added (Section 5.1.1.5).<br>• Language has been revised to account for the fact that some sites may not allow follow-up on partner pregnancies (Section 5.4.3.2).<br>• Language has been added for consistency with Roche's current data retention policy and to accommodate more stringent local requirements (if applicable) (Section 7.6).<br>• The process for reviewing and handling protocol deviations has been updated per internal standard operating procedures (Section 9.2).<br>Additional minor changes have been made to improve clarity and consistency. Substantive new information appears in italics. This amendment represents cumulative changes to the original protocol.
V5 (Canada)<br>01-Feb-2019	**PROTOCOL AMENDMENT, VERSION 5 (CANADA): RATIONALE**<br>Protocol GS39684 has been amended to remove the option for patients to enter the open-label extension (OLE) Study GS40868 after completing the 8-week treatment period. Study GS40868 has not been approved by the Canadian health authority so the OLE study is not available to patients.<br>The following changes have been made:<br>• OLE language has been removed throughout the protocol. Figure 1, "Study Schema," has been updated. Section 3.1.2 has been deleted and the appropriate sections have been revised (Sections 3.1, 3.1.2, and 4.3.2.1 and Appendix 1).<br>• Exclusion criteria specific to German sites has been deleted (Section 4.1.2).<br>• Post-trial access to fenebrutinib has been updated for the situation in which continued access to the study drug is unlikely per the Roche Global Policy on

	Continued Access to Investigation Medicinal Product and there are no plans for an extension study (Section 4.3.4).<br>• An administrative error for the Urticaria Patient Daily eDiary has been corrected (Appendix 3).<br><br>Additional minor changes have been made to improve clarity and consistency. Substantive new information appears in italics. This amendment represents cumulative changes to the original protocol.
V5 (Germany) 01-Feb-2019	**PROTOCOL AMENDMENT, VERSION 5 (GERMANY): RATIONALE**<br><br>Protocol GS39684 has been amended to remove the option for patients to enter the open-label extension (OLE) Study GS40868 after completing the 8-week treatment period. Study GS40868 has been withdrawn in Germany so the option for patients to roll over in the OLE study is not available.<br>The following changes have been made:<br>• OLE language has been removed throughout the protocol. Figure 1, "Study Schema," has been updated. Section 3.1.2 has been deleted and the appropriate sections have been revised (Sections3.1, 3.1.2, and 4.3.2.1 and Appendix 1).<br>• Post-trial access to fenebrutinib has been updated for the situation in which continued access to the study drug is unlikely per the Roche Global Policy on Continued Access to Investigation Medicinal Product and there are no plans for an extension study (Section 4.3.4).<br>• An administrative error for the Urticaria Patient Daily eDiary has been corrected (Appendix 3).<br><br>Additional minor changes have been made to improve clarity and consistency. Substantive new information appears in italics. This amendment represents cumulative changes to the original protocol.
