# Supplementary material for: Fenebrutinib in H1 antihistamine-refractory chronic spontaneous urticaria: a randomized phase 2 trial
Source: Nat Med. 2021 Nov 8;27(11):1961–9. doi: 10.1038/s41591-021-01537-w (PMC8604722; doi:10.1038/s41591-021-01537-w)
Supplement: Supplementary file 2 — Reporting Summary [file 41591_2021_1537_MOESM2_ESM.pdf]

Corresponding author(s): Marcus Maurer

Last updated by author(s): Aug 26, 2021

Reporting Summary

Nature Portfolio wishes to improve the reproducibility of the work that we publish. This form provides structure for consistency and transparency in reporting. For further information on Nature Portfolio policies, see our Editorial Policies and the Editorial Policy Checklist.

Statistics

For all statistical analyses, confirm that the following items are present in the figure legend, table legend, main text, or Methods section.

n/a	Confirmed	
☐	☒	The exact sample size (*n*) for each experimental group/condition, given as a discrete number and unit of measurement
☐	☒	A statement on whether measurements were taken from distinct samples or whether the same sample was measured repeatedly
☒	☐	The statistical test(s) used AND whether they are one- or two-sided<br>*Only common tests should be described solely by name; describe more complex techniques in the Methods section.*
☐	☒	A description of all covariates tested
☐	☒	A description of any assumptions or corrections, such as tests of normality and adjustment for multiple comparisons
☐	☒	A full description of the statistical parameters including central tendency (e.g. means) or other basic estimates (e.g. regression coefficient) AND variation (e.g. standard deviation) or associated estimates of uncertainty (e.g. confidence intervals)
☒	☐	For null hypothesis testing, the test statistic (e.g. *F*, *t*, *r*) with confidence intervals, effect sizes, degrees of freedom and *P* value noted<br>*Give P values as exact values whenever suitable.*
☒	☐	For Bayesian analysis, information on the choice of priors and Markov chain Monte Carlo settings
☒	☐	For hierarchical and complex designs, identification of the appropriate level for tests and full reporting of outcomes
☒	☐	Estimates of effect sizes (e.g. Cohen's *d*, Pearson's *r*), indicating how they were calculated

Our web collection on statistics for biologists contains articles on many of the points above.

Software and code

Policy information about availability of computer code

Data collection	No software was used.
Data analysis	SAS, version 9.4 (SAS Institute, Cary, NC) and R, version 3.6.1, Graphpad Prism version 6

For manuscripts utilizing custom algorithms or software that are central to the research but not yet described in published literature, software must be made available to editors and reviewers. We strongly encourage code deposition in a community repository (e.g. GitHub). See the Nature Portfolio guidelines for submitting code & software for further information.

Data

Policy information about availability of data

All manuscripts must include a data availability statement. This statement should provide the following information, where applicable:

- Accession codes, unique identifiers, or web links for publicly available datasets
- A description of any restrictions on data availability
- For clinical datasets or third party data, please ensure that the statement adheres to our policy

The Study Protocol and the Biomarker Analysis Plan are provided in the Supplementary Material. As per the Roche Global Policy on Sharing of Clinical Study Information, Roche supports data sharing with qualified investigators engaged in rigorous, independent scientific research. The data for this study was available as of October 2020. Access to Roche's de-identified patient-level data is facilitated through the cross-industry request site https://vivli.org. Requests for access to Roche data are made through the Vivli process and supported by a research proposal that is assessed by an Independent Review Panel managed by the Wellcome Trust. The panel considers the scientific merit of each application. This independent group then decides whether or not the data should be provided. On average it takes a few months to access data in the Vivli platform, but the timeline will vary depending on the number of data contributors, the number of studies, and your availability to respond to comments. Analyses performed on the data must be in line with the purpose outlined in the research proposal and be approved by the

Independent Review Panel. The mechanisms for how data will be made available on the platform are outlined on the Vivli website page, "Platform Process at a Glance" (https://vivli.org/about/data-request-review-process/). The Vivli secure research environment allows research teams to access data and conduct analyses in a shared workspace that is equipped with analytical tools and may be flexibly configured. The download of Roche anonymized patient-level data from the secure environment is not permitted. For further restrictions and information, please visit https://vivli.org. Further details on Roche's criteria for eligible studies are available here (https://vivli.org/members/ourmembers/). For further details on Roche's Global Policy on the Sharing of Clinical Information and how to request access to related clinical study documents, see here (https://www.roche.com/research_and_development/who_we_are_how_we_work/clinical_trials/our_commitment_to_data_sharing.htm).

Field-specific reporting

Please select the one below that is the best fit for your research. If you are not sure, read the appropriate sections before making your selection.

☒ Life sciences ☐ Behavioural & social sciences ☐ Ecological, evolutionary & environmental sciences

For a reference copy of the document with all sections, see nature.com/documents/nr-reporting-summary-flat.pdf

Life sciences study design

All studies must disclose on these points even when the disclosure is negative.

Sample size

The purpose of this phase 2 study was initial efficacy estimation and hypothesis generatio n. Assuming a standard deviation (SD) of 13, a two-sid ed alpha level of 0.10, and a 10% dropout rate at Week 8, we estimat ed that a sample size of 30 patients per arm provided approximately 90% power to detect an 11-point difference in the UAS7 change from baseline at Week 8 between treatment groups.

Data exclusions

Analyses included data from all patients who received at least one dose of study treatment; CONSORT diagrams are included. No subjects were excluded from analysis.

Replication

Phase 2 clinical study; for 200 mg BID dose arm in cohort 2 supports findings from cohort 1; but no replication for 150 mg and 50 mg dose arms. The study was originally designed as a pilot study to enable initial assessment of clinical efficacy in CSU. The 200 mg dose was selected because it was expected to be well-tolerated and to substantially inhibit BTK activity, based on results from the phase 1 studies. On the basis of the PK and PK/PD models constructed using data from relative bioavailability, and the phase 1 studies, the 200 mg BID dose was expected to provide a steady-state exposure achieving 90% maximal inhibitory concentration over the entire dosing interval in greater than 75% of patients. The extent of target engagement required for clinical efficacy was unknown. Based on results from an interim analysis of the pilot study (Cohort 1), a dose-ranging cohort (Cohort 2) was initiated. Because the extent of target engagement required for clinical efficacy was unknown, doses for Cohort 2 were selected to evaluate a range of target engagement and to characterize the dose- and exposure-response relationships for safety and efficacy in order to select the optimal dose. As a result, replication was not performed for the 50 mg and 150 mg doses.

Randomization

Genentech, Inc. provided the specifications for an interactive voice/web-based response system (IxRS) with a stratified permuted blocks randomization scheme with stratification by country. The IxRS randomly allocated patients to each of four treatment arms (~1:1:1:1; Cohort 2) or to 200 mg oral BID fenebrutinib or placebo (~2:1; Cohort 1). Genentech, Inc. provided 50 mg fenebrutinib and matching placebo tablets.

Blinding

Patients and study site personnel were blinded to individual treatment assignments throughout the study. Results of assessments that might have unblinded investigators to patient treatments, other than local standard and safety laboratory data, were not provided to site staff. During the trial, Genentech personnel, except for members of the IMC, monitored blinded clinical and safety data and had access to unblinded data if needed for safety evaluations. The IMC was also responsible for reviewing results of pre-planned interim analyses. All patients took fenebrutinib and/or placebo tablets orally twice daily (every 12 hours) to maintain blinding.

Reporting for specific materials, systems and methods

We require information from authors about some types of materials, experimental systems and methods used in many studies. Here, indicate whether each material, system or method listed is relevant to your study. If you are not sure if a list item applies to your research, read the appropriate section before selecting a response.

Materials & experimental systems

n/a	Involved in the study	
☐	☒	Antibodies
☒	☐	Eukaryotic cell lines
☒	☐	Palaeontology and archaeology
☒	☐	Animals and other organisms
☐	☒	Human research participants
☐	☒	Clinical data
☒	☐	Dual use research of concern

Methods

n/a	Involved in the study	
☒	☐	ChIP-seq
☒	☐	Flow cytometry
☒	☐	MRI-based neuroimaging

Antibodies

Antibodies used	Antibody name in Methods: Mouse IgG x FCER1A<br>Supplier: Abcam<br>Supplier name: Anti-Fc epsilon RI/FCER1A antibody [9El]<br>Supplier cat#: ab54411<br>Link to datasheet: https://www.abcam.com/fc-epsilon-rifcer1a-antibody-9e1-ab54411.html<br>Lot#s used: GR3223330-6, GR3264138-4<br><br>Antibody name in Methods: Goat x Mouse IgG (minimal cross-reactivity), HRP<br>Supplier: Biolegend<br>Supplier name: HRP Goat anti-mouse IgG (minimal x-reactivity) Antibody<br>Supplier Cat# : 405306<br>Link to datasheet: https://www.biolegend.com/en-us/products/hrp-goat-anti-mouse-igg-minimal-x-reactivity-1395<br>Lot#s used: B257228, B269637<br><br>Antibody name in Methods: Affinipure Goat anti Human IgG-Fc (minimum cross-reactivity)<br>Supplier: Jackson Immunolabs<br>Supplier name: Peroxidase AffiniPure Goat Anti-Human IgG, Fcy fragment specific<br>Supplier Cat#: 109-035-098<br>Link to datasheet: https://www.jacksonimmuno.com/catalog/products/109-035-098<br>Lot#s used: 140585, 144464<br><br>Dilution range: 6-point, 3-fold dilution series.
Validation	See manufacturer datasheets for vendor-specific validation and references.<br>The IgG anti-FceR1 ELISA protocol is described in detail in the Supplementary Methods section. This ELISA used recombinant human FceR1a as a capture protein. Anti-Fe epsilon RI/FCER1A antibody [9El] was used as the standard curve (for relative quantification). HRP Goat ant i-mouse IgG (minimal x-reactivity antibody) was the detection antibody for the standard curve & Peroxidase AffiniPure Goat Ant i-Hum an IgG, Fey fragment specific antibody was the detection antibody for the patient serum samples. Validation of the antibodies used in the anti-FceR1 ELISA included titrations to establish the concentrat ion yielding minimal background and maximum signal/background ratio. This assay was also validated and performed as a "competitive inhibition" assay, to determine the specific reactivity of IgG anti FceR1a in the patient samples.

Human research participants

Policy information about studies involving human research participants

Population characteristics	The study was designed to enroll male and female adults between the age of 18 -75 years with a diagnosis of CSU for >6 months and refractory to treatment with a combination of H1 antihistamines consistent with standard of care. While H1 anti histamines are the mainstay of therapy for CSU, some patients do not respond or respond only partially to these therapies, and these patients tend to experience more severe disease. This patient population was selected for this study because of the unmet medical need for more effective oral treatments.
Recruitment	Standard site outreach and physician referrals were utilized for patient recruitment. Patients were identified from 21 dermatology, allergy, and clinical centers in Germany, Canada, and the US. Potentially eligible patients were invited to take part in the study. There is no potentially self-selection bias or any other bias that may be present that are likely to impact results.
Ethics oversight	The study protocol was approved by central institutional review boards for the US/Canada and Germany: Advarra, Columbia, MD (Canada and US); Landesamt für Gesundheit und Soziales Geschaftsstelle der Ethik-Kommission des Landes, Berlin, Germany (Germany).

Note that full information on the approval of the study protocol must also be provided in the manuscript.

Clinical data

Policy information about clinical studies

All manuscripts should comply with the ICMJE guidelines for publication of clinical research and a completed CONSORT checklist must be included with all submissions.

Clinical trial registration	EudraCT: 2016-004624-35; ClinicalTrials.gov: NCT03137069
Study protocol	See Supplementary Information
Data collection	This phase 2, multicenter, randomized, double-blind, placebo-controlled study was conducted at 21 centers in Canada, Germany, and the United States. Screening started 01 May 2017 in Canada, the first patient was enrolled on 26 May 2017, the last patient last visit was 25 October 2019, and the database was locked on 13 December 2019.<br><br>Study sites:<br>Sussman Clinical Research, 202 St. Clair Avenue W, M4V 1R2, Toronto, Ontario, CANADA<br>Centre de Recherche Applique En Allergie de Quebec, 2590 Boulevard Laurier, 2e Étage, Bureau 225, G1V 4M6, Quebec City, Quebec, CANADA

Charite Mitte; Klinik fur Dermatologie, Charite Platz 1, Allergiezentrum Charite, 10117, Berlin, GERMANY
Universitätsmedizin Johannes Gutenberg Universität, Langenbeckstrasse 1, 55131, Mainz, GERMANY
Cheema Research, Inc, 110 - 470 Hensall Circle, L5A 3V4, Mississauga, Ontario, CANADA
Universitätsklinikum Carl Gustav Carus, Klinik und Poliklinik für Augenheilkunde, Fetscherstrasse 74, Carl Gustav Carus University Clinic, 01307, Dresden, GERMANY
Klinik für Haut- und Geschlechtskrankheiten, Universitätsklinikum Münster, Von- Esmarch-Strasse 58, 48149, Muenster, GERMANY
Kern Allergy Med Clinic, Inc., 2121 17th Street, Bakersfield, CA, 93301, UNITED STATES
Ottawa Allergy Research Corp, 1081 Carling Avenue, Suite 707, K1Y 4G2, Ottawa, Ontario, CANADA
Integrated Research of Inland, 440 N Mountain Ave, Suite 301, Upland, CA, 91786, UNITED STATES
Licca Clinical Research Institute, Hofackerstrasse 19, 86179, Augsburg, GERMANY
Hautarztpraxis Mahlow, Am Bahnhof 1, 15831, Mahlow, GERMANY
Lynderm Research, 25 Main Street Markham North, L3P 1X2, Markham, Ontario, CANADA
Allergy & Asthma Consultants, 369 Main Street, Suite 200, Redwood City, CA, 94063, UNITED STATES
Timber Lane Allergy and Asthma Research, LLC, 54 Timber Lane, South Burlington, VT, 05403, UNITED STATES
Clinical Research Center of Alabama, LLC, 504 Brookwood Boulevard, Suite 250, Birmingham, AL, 35209, UNITED STATES
Asthma, Nasal Disease, and Allergy Research Center of New England, 95 Pitman Street, Providence, RI, 02906, UNITED STATES
Vital Prospects Clinical Research Institute PC - CRN, 7307 S. Yale Ave., Suite 200, Tulsa, OK, 74136, UNITED STATES
ALLERGY & ASTHMA IMMUNOLOGY ASSOCIATES, 7514 EAST MONTEREY WAY, SUITE 1, SCOTTSDALE, AZ, 85251, UNITED STATES
RENSTAR MEDICAL RESEARCH, 21 Northeast First Avenue, OCALA, FL, 34470, UNITED STATES
SOUTHERN CALIFORNIA RESEARCH CENTER, 11190 Warner Avenue, Suite 401, Fountain Valley, CA, UNITED STATES

Outcomes

The primary endpoint was change from baseline in the UAS7 at Week 8. Secondary endpoint s were change from baseline in UAS7 at Week 4 and proportion of patients well-controlled (UAS7≤6) at Week 8. Other efficacy endpoints included proportion of patients well-controlled at Week 4, change from baseline in weekly itch score at Weeks 4 and 8, change from baseline in weekly hive score at Weeks 4 and 8, proportion of patients who achieved complete response (UAS7=0) at Weeks 4 and 8, proportion of patients achieving the MID in UAS7 (reduction from baseline UAS7≥11) at Weeks 4 and 8, and time to achieving MID in UAS7. Safety outcomes were the incidence and severity of AEs, and changes in vital signs, physical examination findings, electrocardiograms (ECGs), and clinical laboratory results.
